# Doubly stereoconvergent construction of vicinal all-carbon quaternary and tertiary stereocenters by Cu/Mg-catalyzed propargylic substitution

**DOI:** 10.1038/s41467-022-29986-y

**Published:** 2022-05-04

**Authors:** Xiang Pu, Qiu-Di Dang, Lei Yang, Xia Zhang, Dawen Niu

**Affiliations:** Department of Emergency, State Key Laboratory of Biotherapy and Cancer Center, West China Hospital and School of Chemical Engineering, Sichuan University, Chengdu, 610041 China

**Keywords:** Organic chemistry, Asymmetric catalysis

## Abstract

The construction of vicinal, congested stereocenters with high selectivities is of general utility in chemistry. To build two such stereocenters in one step from readily available starting materials is very desirable, but remains challenging. We report here a doubly stereoconvergent, Cu/Mg-catalyzed asymmetric propargylic substitution reaction to convert simple starting materials to products with vicinal tertiary and all-carbon quaternary stereocenters in high yields and excellent diastereo- and enantioselectivities. Both the nucleophiles and the electrophiles employed in this transformation are racemic. This reaction uses earth abundant metal catalysts, operates under ambient conditions, and demonstrates broad substrate scope. The products of this reaction are functional group rich and synthetically versatile. Key to the success of this development is the devise of a Cu/Mg dual catalytic system and the identification of a bulky tridentate pyridinebisimidazoline (PyBim) ligand.

## Abstract (web summary)

The construction of vicinal, congested stereocenters with high selectivities is of general utility in chemistry. Here the authors report a doubly-stereoconvergent, Cu/Mg-catalyzed asymmetric propargylic substitution reaction to convert simple starting materials to products with vicinal tertiary and all-carbon quaternary stereocenters in high yields and excellent diastereo- and enantioselectivities

## Introduction

**Fig. 1:**
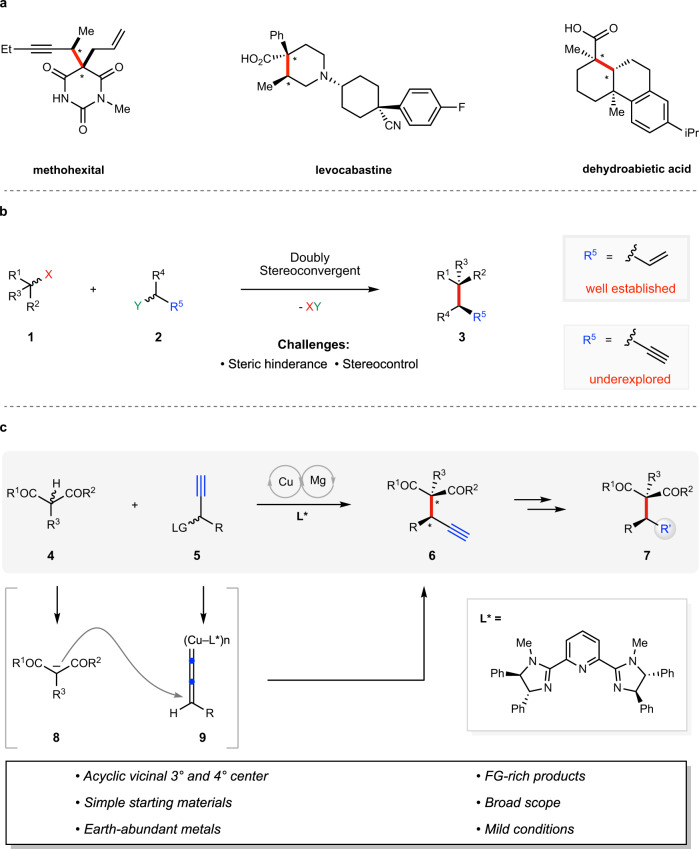
Enantioselective construction of vicinal tertiary and all-carbon quaternary stereocenters. **a** Vicinal 3° and all-carbon 4° stereocenters in pharmaceutical agents. **b** Doubly stereoconvergent approaches to make vicinal stereocenters. **c** Cu/Mg-dual catalytic APS reaction to make vicinal 3° and 4° stereocenters (this work).

Vicinal stereocenters are prevalent in naturally occurring products and pharmaceutical agents (Fig. 1a), and therefore, the stereoselective construction of these structural units represents a subject of a longstanding interest in chemistry^1^. Traditional approaches usually establish the two stereocenters sequentially, which thus require multistep manipulations. A conceptually straightforward and efficient approach to access vicinal stereocenters is by doubly stereoconvergent^2–4^ C–C bond-forming reactions, such as the ones depicted in Fig. 1b. In this case, racemic compounds **1** and **2** are each converted to prochiral intermediates, whose subsequent combination affords the stereochemically defined product **3** with two stereocenters. In practice, however, the requirement to simultaneously control the relative and absolute configurations during the key C–C bond-forming event presents significant difficulties. This task becomes more challenging if an all-carbon quaternary center is involved, as a result of the increased steric repulsion. The challenge further escalates if none of the two stereocenters is embedded in a cycle, due to the enhanced conformational flexibility^5–7^.

In spite of these challenges, extensive efforts have been devoted to developing reactions shown in Fig. 1b, with remarkable progress achieved^8^. Among the methods reported to access these stereodiads, the transition metal-catalyzed asymmetric allylic substitution (AAS) reaction^9–15^ (**3**, R^5^ = vinyl, Fig. 1b) has attracted the most attention and shown tremendous power. For instance, the Trost group developed a Mo-catalyzed AAS of cyanoesters using cinnamyl carbonates, affording products with vicinal tertiary and quaternary carbons^16^. The Stoltz group employed the Ir-catalyzed AAS reaction in accessing this type of stereodiads^17,18^. The Carreira group reported an elegant allylic alkylation of aldehydes/ketones by their Ir/organocatalyst dual catalytic system^19,20^. These methods are flexible since they employ readily available starting materials and generate synthetically versatile, terminal-alkene-containing products (cf. **3**). Notably, however, for most of the products **3** accessed by the above AAS-based methods, the R^4^ substituent (carried from electrophile **2**) is limited to aryl or alkenyl groups. These advancements notwithstanding, general methods for building vicinal acyclic, congested stereodiads remain highly needed in synthesis. Ideally, a reaction may employ economical and simple catalysts and at the same time accommodate the broad scope of nucleophiles and electrophiles.

Our group^21–24^ has been interested in the copper-catalyzed asymmetric propargylic substitution (APS) reactions^25–38^. In terms of bond disconnection, the metal-catalyzed APS reactions are analogous to the AAS reactions. In terms of synthetic utility, the terminal alkyne group installed by the APS reaction is arguably the most versatile^39,40^ functionality in chemistry. However, the scope of nucleophiles that can be employed in the Cu-catalyzed APS reactions remains limited compared with the AAS reactions. To understand such a limitation, we have shown through kinetic studies an important mechanistic distinction of the Cu-catalyzed APS reactions: the generation of electrophilic intermediate (see **9** in Fig. 1c) is likely the rate-limiting step, and this intermediate would undergo polymerization in the absence of a potent nucleophile^24^. This feature may have rendered expanding the scope of nucleophiles a difficult task. Regardless, through developing new catalytic systems, remarkable progress has been achieved in this field. For example, we^21,22,24^ and the Nishibayashi group^41^ have realized asymmetric propargylation of aliphatic alcohols, a class of nucleophiles previously considered inactive in this transformation.

In continuation of our effort in this field, we wonder if we could employ the simple, linear 1,3-dicarbonyl based nucleophiles (e.g., **4**) in the Cu-catalyzed APS reactions (cf. Fig. 1c, 4 + **5** to **6**, **via 8** and **9**). Once realized, this would represent a general tool to construct acyclic, vicinal tertiary, and all-carbon quaternary centers. Furthermore, by facile functional group interconversions of the terminal alkyne group installed, a broad scope of derivatives could be readily accessed. We reasoned that the skinny nature of the alkyne substituent in **5** (and **9**) could, to some extent, ameliorate the steric repulsion between both reactants^42^. If **4** could be deprotonated under mild conditions, the resulting soft nucleophile **8** should be able to trap **9** effectively. However, a preeminent challenge lies in how to control the configuration of the acyclic, all-carbon quaternary center in **6**: the prochiral nucleophilic center in **8** is quite distant from the chiral environment exerted by the ligands in **9**. Previous related studies had to employ cyclic nucleophiles such as 2-indolones to ensure high diastereoselectivities^43^.

In this work, we develop a Cu/Mg-dual catalytic system for a highly diastereoselective and enantioselective propargylation of simple, linear 1,3-dicarbonyl compounds (Fig. 1c). We establish that the use of a bulky ligand for Cu-catalyst and Mg(O^*t*^Bu)_2_ as a base is vital for the efficiency and selectivity of this process. As key advantages of this method, it employs non-precious metal catalysts, and readily available reagents/substrates, and demonstrates significant scope with respect to both reaction partners. Importantly, the R group in electrophile **5** could be either aryl or alkyl in nature. The terminal alkyne-containing products **6** could be smoothly converted to other classes of compounds (**6**–**7**), further adding to its synthetic utility.

## Results

### Reaction optimization

**Fig. 2:**
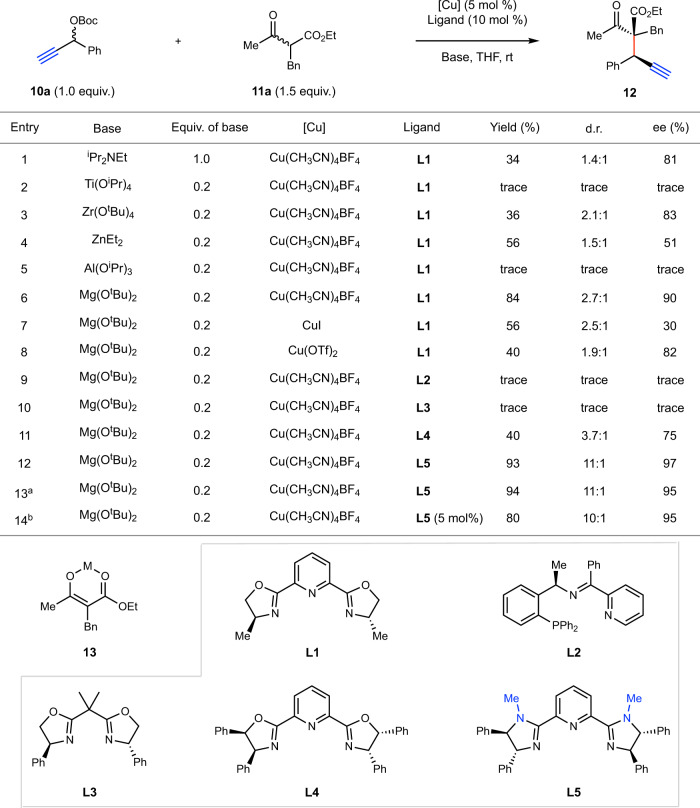
Condition optimization. Reactions in this table were performed on a 0.1 mmol scale. Yields and d.r. values were determined by ^1^H NMR spectroscopy of the crude reaction mixture with 1,1,2,2-tetrachloroethane as an internal standard. The ee values were determined by HPLC analysis. ^a^**10a** (1.5 equiv.) and **11a** (1.0 equiv.) were used. ^b^The ratio of ligand to Cu is 1:1.

To commence our investigation, we used propargyl carbonate **10a** and β-keto ester **11a** as model substrates (Fig. 2). Not surprisingly, under the “classical” Cu-catalyzed APS reaction conditions [Cu(CH_3_CN)_4_BF_4_ as the catalyst precursor, **L1** as ligand, and ^*i*^Pr_2_NEt as base], product **12** was formed, but with low yield and poor diastereoselectivity (entry 1). We then focused our efforts to improve the efficiency and selectivity of this process. We first considered strategies to restrict the rotational flexibility of the nucleophile **11a**. Along this line, we investigated the use of various Brønsted bases that contain Lewis acidic metal counterions^44–46^, with the expectation that they would deprotonate **11a** to form cyclic metal enolate intermediates such as **13**. Among the bases screened (entries 2–6), Mg(O^*t*^Bu)_2_ afforded **12** in best yield (84%) but still with moderate diastereoselectivity (entry 6). The use of other Cu sources (entries 7–8) instead of Cu(CH_3_CN)_4_BF_4_ led to inferior selectivities and efficiency. We next examined the impact of ligand structures on the reaction performance (entries 9–12). The use of Hu’s tridentate *P, N, N*- ligand^28^ or bis-oxazoline ligand^47^ **L3** gave no detectable amount of product. Interestingly, the use of a bulkier Pybox-type ligand **L4** slightly improved the diastereoselectivity. Along this line, we synthesized various sterically more demanding ligands, and identified pyridinebisimidazoline^48^ (PyBim) **L5** to give **12** in excellent enantio- and diastereoselectivities (entry 12). These results suggest that using large ligands may be conducive to acyclic stereocontrol for this transformation. Under the above conditions, we further showed either **10a** or **11a** could serve as the limiting reagent, giving almost identical results (entry 13). Lastly, when the ratio of ligand to Cu was reduced to 1:1, the product was obtained in almost identical stereoselectivitities (entry 14). We used a slightly excess amount of ligand in the following studies to ensure efficient ligation of Cu salts and to minimize background nonstereoselective propargylation processes.

### Reaction scope

**Fig. 3:**
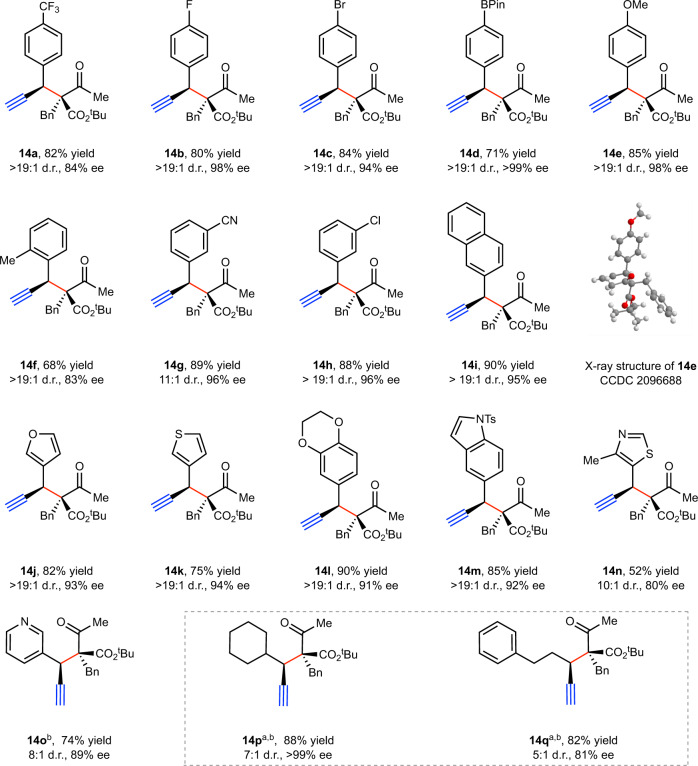
Substrate scope with respect to electrophiles. Reactions were performed on a 0.2 mmol scale in THF (2 mL) with 5 mol% Cu(I), 10 mol% **L5**, and 20 mol% Mg(O^*t*^Bu)_2_. Yield is that of an isolated product. The ee values were determined by HPLC analysis, the d.r. values were determined by ^1^H NMR analysis of the crude reaction mixture. ^a^The pentafluorobenzoate group was used as leaving group. ^b^Cu(CH_3_CN)_4_BF_4_ (20 mol%), **L5** (40 mol%), and Mg(O^*t*^Bu)_2_ (80 mol%) were used. See SI for experimental details.

With the optimized reaction conditions established, we next examined the substrate scope of this transformation. As shown in Fig. 3, a variety of propargyl carbonates could be employed as electrophiles. For example, the aryl group at the propargylic position could be either electron-rich (**14e, 14l**) or electron-deficient (**14a**, **14g**). Those bearing a substituent at the *ortho* position (**14f**) or containing bicyclic ring systems (**14i**, **14l–m**) partook in the reaction smoothly. Functional groups such as aryl halides (**14b–c**, **14h**), boronic acid esters (**14d**), and cyano (**14g**) are compatible with the reaction conditions. These functional groups in products are useful handles for further derivatizations. Importantly, pharmaceutically relevant heterocycles, including furans (**14j**), thiophenes (**14k**), thiazoles (**14n**), benzodioxanes (**14l**), pyridines (**14o**), and indoles (**14m**) could also be incorporated. Lastly, we show that the substituent at the propargylic center is not limited to aryls, and those bearing alkyl groups can be employed (**14p–q**) under slightly modified conditions.

**Fig. 4:**
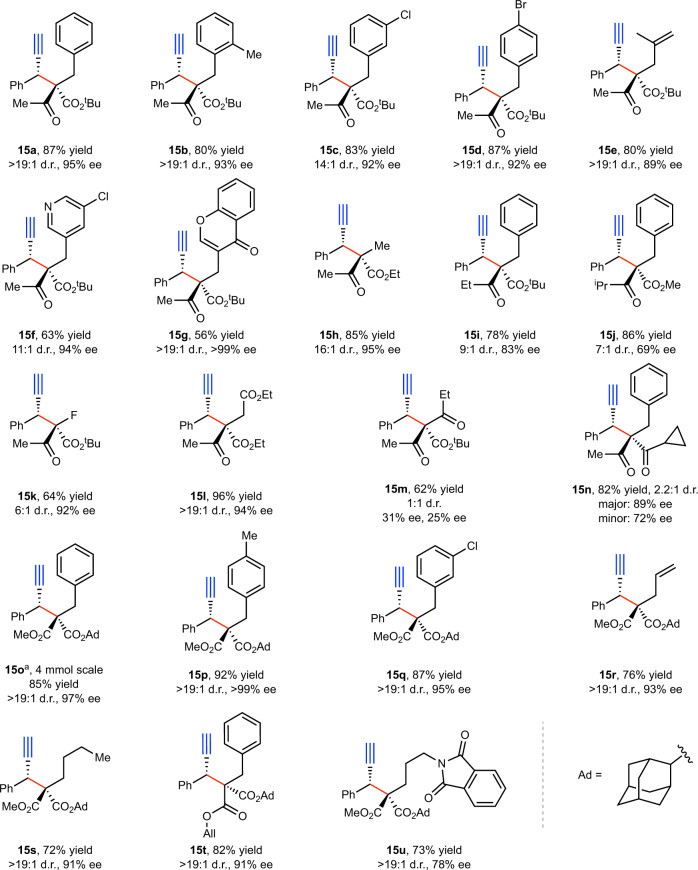
Substrate scope with respect to nucleophiles. Reactions were performed on a 0.2 mmol scale in THF (2 mL) with 5 mol% Cu(I), 10 mol% **L5**, and 20 mol% Mg(O^*t*^Bu)_2_. Yield is that of an isolated product. The ee values were determined by HPLC analysis, the d.r. values were determined by ^1^H NMR analysis of the crude reaction mixture. ^a^Cu(CH_3_CN)_4_BF_4_ (15 mol%), **L5** (30 mol%), and Mg(O^*t*^Bu)_2_ (50 mol%) were used. See SI for experimental details.

We then examined the scope with respect to the nucleophiles (Fig. 4). To our delight, both β-ketoesters (**15a–m**) and malonate esters (**15o–u**) are tolerated by this reaction. Again, various (hetero)aryl groups of different electronic properties and substitution patterns can be incorporated. Notably, pyridine rings (**15f**) and chromones (**15g**) are well accommodated. Moreover, various alkyl substituents are allowed. In addition, alkene groups (**15e**, **15r**) are also compatible with our conditions. Even the allyl ester group in **15t**, a potent reacting functionality in the TM-catalyzed AAS reaction, is completely inert under our conditions, highlighting the mechanistic distinction between the AAS and the APS reactions. Particularly noteworthy is the selective formation of product **15k**, which contains a tertiary fluoride: methods to prepare fluorine-containing stereocenters remain underdeveloped but highly needed^49^. Nucleophiles bearing three different carbonyl groups **(15l**, **15m)** were also examined. Interestingly, while product **15l** was obtained with excellent diastereo- and enantioselectivities, **15m** was formed in poor selectivities. Reactions employing 1,3-diketone nucleophiles proceeded with good efficiency but low diastereoselectivites (**15n**). Regardless, we show this reaction could be carried out on a gram-scale under slightly modified conditions, with no notable erosion of yield and stereoselectivities (**15o**).

### Product derivatization

**Fig. 5:**
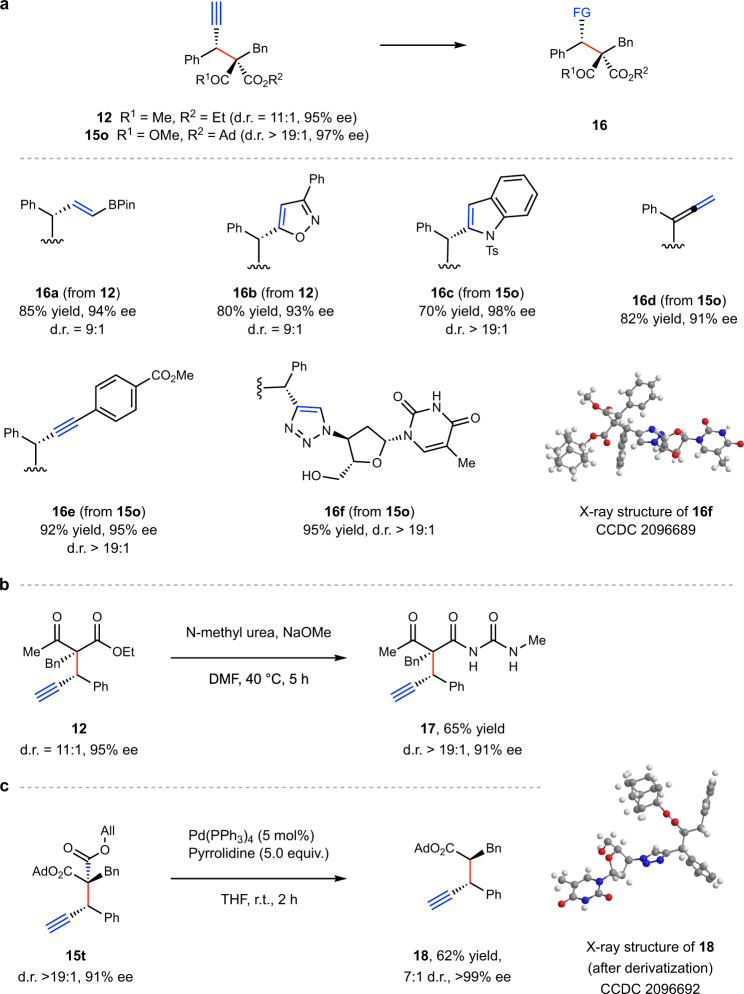
Product derivatization. **a** Derivatization of **12** and **15o**. **b** Derivatization of **12**. **c** Derivatization of **15t**. Reactions were performed on a 0.1 mmol scale. Yield is that of an isolated product. The ee values were determined by HPLC analysis. The d.r. values were determined by ^1^H NMR analysis of the crude reaction mixture. See SI for experimental details.

To further demonstrate the synthetic utility of this method, we performed product derivatization reactions shown in Fig. 5. First, treatment of **12** with pinacolborane (H-BPin) in the presence of HZrCp_2_Cl led to vinyl boronic acid ester **16a**^50^. Alkyne **12** could also undergo [3 + 2] cycloadditions with the in situ generated nitrile oxides, to afford 1,2-oxazoles **16b**^51^. Further, alkyne **15o** partook in an annulation reaction to give the indole **16c**^52^. Treating **15o** with basic conditions generated allene **16d**^53^. Moreover, alkyne **15o** could participate in a Sonagashira coupling with methyl 4-iodobenzoate, leading to **16e**^54^. The CuAAC reaction^40^ of **15o** with anti-HIV drug Zidovudine resulted in **16f** with excellent efficiency. Notably, selective amination^55^ of **12** could be achieved using methylurea with NaOMe employed as a base. Finally, treatment of **15t** with pyrrolidine in the presence of Pd(PPh_3_)_4_ triggered a deallylation and a decarboxylation cascade^56^, yielding product **18** that bear contiguous tertiary stereocenters in good diastereo- and enantioselectivities. The synthetic versatility of these products adds to the utility of our current method.

### Mechanistic studies

**Fig. 6:**
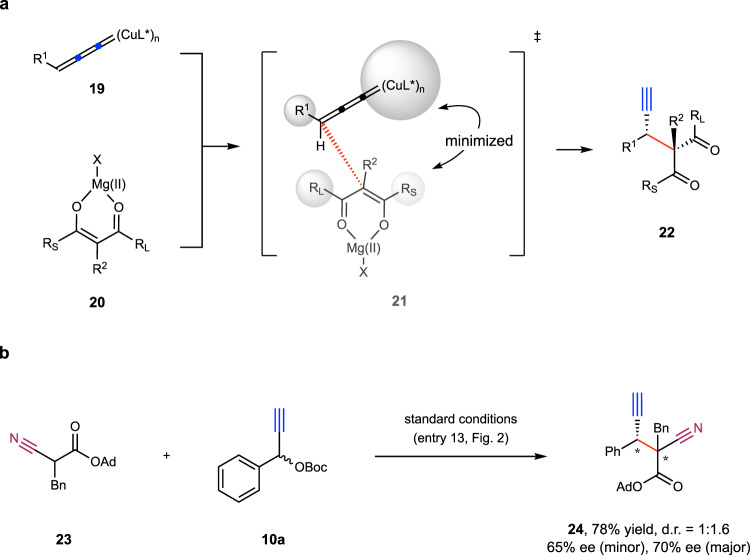
Mechanistic studies. **a** Proposed stereochemical model. **b** Reaction of substrate **23**. The ee value was determined by HPLC analysis. The d.r. value was determined by ^1^H NMR analysis of the crude reaction mixture. See SI for experimental details.

To explain the observed diastereoselectivities of our reaction, we proposed the stereochemical model in Fig. 6a based on experimental results and literature precedence^25,26^. We reason that Mg(O^*t*^Bu)_2_ activates nucleophiles by deprotonating β-ketoesters to form the cyclic intermediate **20**. On the other hand, the copper complex activates electrophiles by forming the copper allenylidene intermediate **19**. Attack of **20** to **19** may adopt a geometry shown in Fig. 6 (see **21**), in order to minimize the repulsion between the ligated copper moiety and the large substituent of **20**. The use of bulky ligand to complex with Cu(I) helped to better differentiate the two substituents (R_L_ vs. R_S_) in the nucleophiles, leading to higher diastereoselectivities. As shown in Fig. 6b, the reaction employing cyanoester **23** as nucleophile under our standard conditions gave the corresponding product **24** with poor diastereomeric ratio. This is likely because cyanoester **23** could hardly form a cyclic metallic complex (cf. **18)** with Mg(O^*t*^Bu)_2_, due to the conformational constraints imposed by the linear cyano group. These results provide support for our proposed stereochemical model.

## Discussion

In conclusion, we have established a general, mild, enantio-, and diastereoselective method to construct vicinal tertiary and all-carbon quaternary stereocenters from simple starting materials. The method, which exploits the copper-catalyzed asymmetric propargylic substitution reaction, builds two stereocenters simultaneously by a doubly stereoconvergent process and furnishes a broad array of functional group-rich, terminal alkyne-containing products. Critical to the success of this development are the use of a Cu/Mg-dual catalytic system and the use of a bulky, tridentate PyBim-type ligand. The synthetic versatility of products further adds to the power of this method. We anticipate this method will be rapidly adopted to make the challenging stereodiads that are frequently encountered in natural products and drug candidates, and inspire developments of novel Cu-catalyzed APS reactions.

## Methods

### General procedures for the Cu/Mg-catalyzed propargylation

In an N_2_-filled glovebox, Cu(CH_3_CN)_4_BF_4_ (3.2 mg, 0.01 mmol, 5 mol%), **L5** (11.0 mg, 0.02 mmol, 10 mol%), and THF (2 mL) were sequentially added to an 8 mL screw cap vial containing a magnetic stirring bar. The mixture was stirred at r.t. for 1 h. Then, propargyl carbonate **10** (0.30 mmol, 1.5 equiv.), 1,3-dicarbonyl compound **11** (0.20 mmol, 1.0 equiv.), and Mg(O^*t*^Bu)_2_ (0.04 mmol, 0.2 equiv.) were added sequentially, and the resulting mixture was allowed to stir at r.t. for 24 h. After completion, the reaction mixture was quenched by the addition of water. The mixture was extracted with ethyl acetate three times, and the combined organic layers were washed with brine, dried over anhydrous Na_2_SO_4_, and concentrated. The crude product was purified by flash chromatography, dried under vacuum, weighed, and sampled for HPLC analysis.

## Supplementary information

Supplementary Information

## Data Availability

Additional data supporting the findings described in this paper are available in the Supplementary Information. The X-ray crystallographic data for structures reported in this study have been deposited at the Cambridge Crystallographic Data Centre (CCDC), under deposition numbers CDCC 2096688 (**14e**), CDCC 2096689 (**16f**), and CDCC 2096692 (a derivative of **18**). These data can be obtained free of charge from the Cambridge Crystallographic Data Centre (www.ccdc.cam.ac.uk/data_request/cif).
