# Supplementary material for: Doubly stereoconvergent construction of vicinal all-carbon quaternary and tertiary stereocenters by Cu/Mg-catalyzed propargylic substitution
Source: Nat Commun. 2022 May 4;13:2457. doi: 10.1038/s41467-022-29986-y (PMC9068607; doi:10.1038/s41467-022-29986-y)
Supplement: Supplementary file 1 — Supplementary Information [file 41467_2022_29986_MOESM1_ESM.pdf]

Supplementary Information

Doubly Stereoconvergent Construction of Vicinal All-Carbon Quaternary and Tertiary Stereocenters by Cu/Mg-Catalyzed Propargylic Substitution

Xiang Pu[1‡], Qiu-Di Dang[1‡], Lei Yang[1], Xia Zhang[1*], and Dawen Niu[1*]

[1]Department of Emergency, State Key Laboratory of Biotherapy and Cancer Center, and School of Chemical Engineering, Sichuan University, Chengdu 610041, China.

*Correspondence to: niudawen@scu.edu.cn; zhang-xia@scu.edu.cn.

Contents of Supplementary Information

1 **Supplementary Methods** 3

General Information 3

General Procedure for the Preparation of (*4R,5R*)-PyBim-Me 4

Procedures for Substrate Synthesis 5

Characterization Data of Substrates 9

Optimization Tables 17

General Procedures for the Cu/Mg-catalyzed Propargylation 22

Gram-Scale Propargylation (4 mmol) 23

Characterization Data for Propargylation Products 24

Derivatization Reactions 48

Determination of Non-Linear Effect 58

2 **HPLC Traces** 60

3 **NMR Spectra** 111

4 **Supplementary References** 249

Supplementary Methods

General Information

Flash column chromatography was performed using silica gel purchased from Qindao Haiyang. $Cu(CH_3CN)_4BF_4$ was purchased from TCI, used as received, and stored in a N_2-filled glovebox. $Mg(O^tBu)_2$ was purchased from Adamas, used as received, and stored in a N_2-filled glovebox. Anhydrous THF (stabilized with BHT, water ≤50 ppm) were purchased from Energy Chemical and used as received. 1,3-dicarbonyl compounds and Ligands were synthesized according to reported methods or modified version. Propargyl carbonates were prepared following the literature methods. NMR yields were determined by using 1,1,2,2-tetrachchloroethane as an internal standard. Unless otherwise noted, all reported yields of the Cu/Mg co-catalyzed propargylation reactions are isolated yields.

All new compounds were characterized by NMR spectroscopy, IR spectroscopy, high resolution mass spectroscopy (HRMS), and melting point (if solids). NMR spectra were recorded on a Bruker AMX 400 spectrometer and were calibrated using TMS (tetramethylsilane) or residual deuterated solvent as an internal reference ($CDCl_3$: 7.26 ppm for ^1H NMR and 77.16 ppm for ^{13}C NMR), and the tabulated data were reported in ppm. All IR spectra were taken on a Thermo Scientific Nicolet iS5 spectrometer (iD5 ATR, diamond), and the tabulated data were reported in cm-^{1}. HRMS spectra were recorded on a Waters Q-TOF Premier. Melting points (M.p.) were recorded on an INESA SGW X-4 melting point apparatus. Diastereomeric ratio (d.r.) values were determined by ^1H NMR analysis of crude reaction mixtures. The enantiomeric excesses (*ee*) of the products were determined by high-performance liquid chromatography (HPLC) analysis performed on Agilent 1100, 1260 and Ultimate 3000 Series chromatographs using a Daicel® chiral column (25 cm). Optical rotations were measured on a Rudolph Research Analytical Autopol VI polarimeter with $[\alpha]_D$ values in degrees; concentration (c) is in g/100 mL.

General Procedure for the Preparation of (*4R,5R*)-PyBim-Me

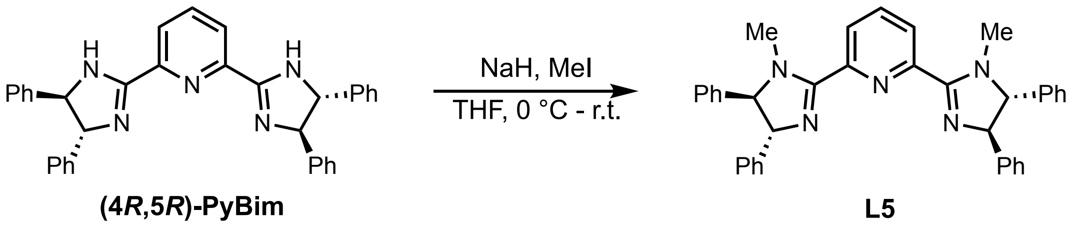

L5 was prepared following reported procedures.[1] To a solution of **(4*R*, 5*R*)-PyBim** (519 mg, 1.0 mmol, 1.0 equiv.) in anhydrous THF (10 mL) cooled at 0 °C was slowly added NaH (60% suspension in mineral oil, 160 mg, 4.0 mmol, 4.0 equiv.) with stirring. After stirred at 0 °C for 15 min, MeI (3.0 mmol, 187 uL, 3.0 equiv.) was added slowly. The resulting mixture was allowed to warm to room temperature and stirred overnight. After completion, the reaction mixture was quenched with water, extracted with EtOAc, dried over anhydrous Na_2SO_4 and evaporated under vacuum. The residue was then purified by column chromatography (CH_2Cl_2: MeOH = 20:1) to yield the title product as a white solid.

2,6-bis((4*R*,5*R*)-1-methyl-4,5-uydiphenyl-4,5-dihydro-1H-imidazol-2-yl)pyridine (L5)

^{1}H NMR (400 MHz, $CDCl_3$) δ: 8.26 (d, *J* = 7.8 Hz, 2H), 7.93 (t, *J* = 7.9 Hz, 1H), 7.42 – 7.27 (m, 20H), 4.98 (d, *J* = 10.1 Hz, 2H), 4.41 (d, *J* = 10.1 Hz, 2H), 3.10 (s, 6H).

^{13}C NMR (101 MHz, $CDCl_3$) δ: 163.77, 149.47, 143.54, 141.26, 137.47, 128.95, 128.61, 127.95, 127.35, 127.17, 127.07, 125.97, 78.95, 77.62, 34.48.

IR (thin film, cm^{-1}): 3027, 3007, 2862, 1561, 1451, 1275, 1261, 1068, 750 and 698.

HRMS (DART-TOF) calculated for $C_{37}H_{34}N_5^+$ $[M+H]^+$ m/z 548.2814, found 548.2809.

M.p.: 143.7-145.2 °C.

$[\alpha]_D^{22}$= 8.9 (c = 0.22, $CHCl_3$).

Procedures for Substrate Synthesis

General Procedure for the preparation of 1,3-dicarbonyl compounds.[2]

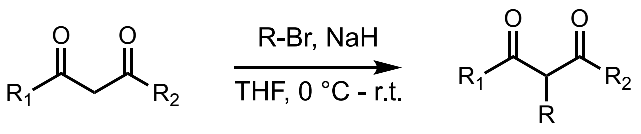

SI-1 **SI-2**

NaH (60% suspension in mineral oil, 6.0 mmol, 1.2 equiv.) was suspended in 20 mL anhydrous THF in a round bottle flask which was allowed to stir at 0 °C. A solution of **SI-1** (5.0 mmol, 1.0 equiv.) in 10 mL anhydrous THF was added slowly. After stirring for 15 min, bromides (6.0 mmol, 1.2 equiv.) was added and the reaction mixture was warmed to room temperature and stirred overnight. After completion, the mixture was quenched with water, extracted with EtOAc, dried over anhydrous Na_2SO_4 and concentrated under vacuum. The residue was then purified by column chromatography to give the product **SI-2**.

Preparation of adamantan-2-yl methyl malonate.[3]

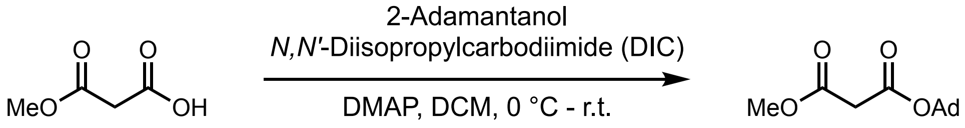

SI-3 **SI-4**

To a solution of **SI-3** (767 mg, 6.5 mmol, 1.3 equiv.) in 20 mL DCM at 0 °C was slowly added 2-adamantanol (760mg, 5.0 mmol, 1.0 equiv.), *N,N'*-Diisopropylcarbodiimide (DIC, 850 uL, 5.5 mol, 1.1 equiv.) and DMAP (61 mg, 0.5 mmol, 0.1 equiv.). Then, the resulting solution was warmed to room temperature and stirred overnight. After completion, the reaction was quenched with water, extracted with EtOAc. The organic phase was then dried over anhydrous Na_2SO_4 and evaporated under vacuum. The residue was then purified by column chromatography to give the product **SI-4**.

Preparation of adamantan-2-yl 2-cyanoacetate.[4]

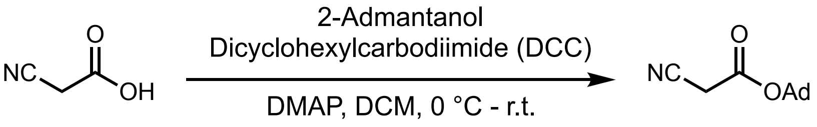

Cyanoacetic acid **SI-5** (510mg, 6.0 mmol, 1.2 equiv.) and 2-Admantanol (760 mg, 5.0 mmol, 1.0 equiv.) are dissolved in 20 mL DCM and cooled to 0° C. DCC (1.0 mL, 6.0 mmol, 1.2 equiv.) and DMAP (cat.) are added to the solution. The resulting solution was warmed to room temperature and stirred overnight. After completion, the reaction mixture is filtered, then concentrated under reduced pressure. The residue was then purified by column chromatography to give the product **SI-6**.

Preparation of adamantan-2-yl allyl malonate.[3],[5]

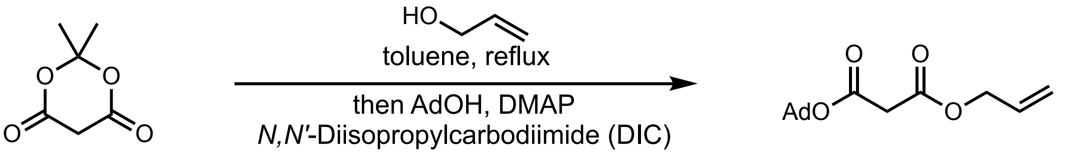

To a stirred solution of Meldrum's acid **SI-7** (1440 mg, 10.0 mmol, 1.0 equiv.) in toluene (50 mL) was added prop-2-en-1-ol (870 mg, 15.0 mmol, 1.5 equiv) and the mixture was heated under refluxed at 110 °C for indicated time. Upon completion of the reaction (as judged by TLC), the reaction mixture was cooled to room temperature. Most of the volatile components were evaporated under reduced pressure. Then DCM (50 mL) was added to the reaction mixture, followed by the addition of 2-adamantanol (1520 mg, 10.0 mmol, 1.0 equiv.), *N,N'*-Diisopropylcarbodiimide (DIC, 1.7 mL, 11.0 mmol, 1.1 equiv.) and DMAP (122 mg, 1.0 mmol, 0.1 equiv.) at 0 °C. The resulting solution was warmed to room temperature and stirred overnight. After completion, the reaction was quenched with water, extracted with EtOAc, dried over anhydrous Na_2SO_4 and evaporated under vacuum. The residue was then purified by column chromatography to give the product **SI-8**.

Preparation of *tert*-butyl 3-oxopentanoate and *tert*-butyl 4-methyl-3-oxopentanoate.[6]

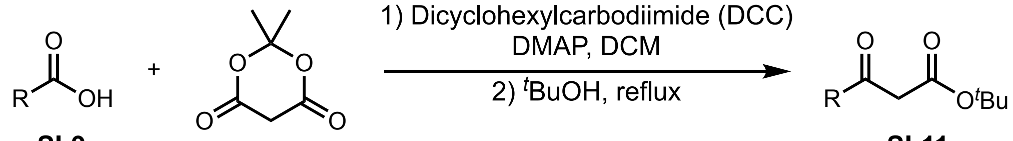

SI-9 R = Et, ^{i}Pr **SI-10** **SI-11** R = Et, ^{i}Pr

To a stirred solution of Meldrum's acid **SI-10** (10.0 mmol, 1.0 equiv.) and the appropriate acid **SI-9** in DCM (50 mL) was slowly added DMAP (11.0 mmol, 1.1 equiv) and Dicyclohexylcarbodiimide (DCC, 11.0 mmol, 1.1 equiv) at 0 °C. The reaction mixture stirred at 0 °C over night and the precipitated solid was removed by filtration and washed with DCM. The filtrate was washed subsequently with 1 M aq $NaHSO_4$ and brine, dried with anhydrous Na_2SO_4, filtered, and the filtrate was evaporated in vacuo. Then, the residue was dissolved in *tert*-butanol (4 mL/mmol) and the solution was refluxed for 5 h. Volatile components were evaporated in vacuo and the residue was purified by column chromatography.

Preparation of bromides.[7]

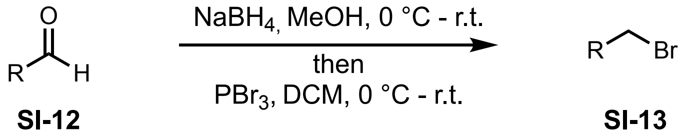

SI-12 **SI-13**

To a stirred solution of aldehydes **SI-12** (5.0 mmol, 1.0 equiv.) in methanol (20 mL) was slowly added sodium borohydride (10.0 mmol, 2.0 equiv.) at 0 °C. After stirring at 0 °C for 10 min, the reaction gradually warm to room temperature and stirred for another 2 hours. After completion, the mixture was quenched by acetone at 0°C and the solvent was removed under reduced pressure. The resulting organic layer was extracted with EtOAc, dried over anhydrous Na_2SO_4 and concentrated under vacuum. Then DCM (20 mL) was added to the reaction mixture, and PBr_3 (10.0 mmol, 2.0 equiv.) was added slowly at 0 °C. After stirring at 0 °C for 10 min, the mixture was warmed to room temperature and stirred for another 2h. After completion, the mixture was

quenched with saturated $NaHCO_3$, extracted with EtOAc and dried over anhydrous Na_2SO_4 and concentrated under vacuum. The resulting crude material was used for next step without further purification.

Preparation of SI-15.[8]

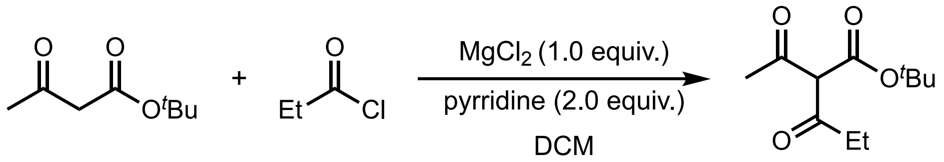

SI-14 **SI-15**

$MgCl_2$ (10.0 mmol, 1.0 equiv.), **SI-14** (10.0 mmol, 1.0 equiv.) and 20 mL DCM were added to a 100 mL round bottom flask. After cooling to 0°C, pyridine (20.0 mmol, 2.0 equiv.) was added. After the solution was stirred for 15 min at 0 °C, Propionyl chloride (10.0 mmol, 1.0 equiv.) was added and the resulting solution was warmed to room temperature and stirred overnight. After completion, the reaction was quenched with water, extracted with DCM. The organic phase was then dried over anhydrous Na_2SO_4 and evaporated under vacuum. The residue was then purified by column chromatography to give the product **SI-15**.

Characterization Data of Substrates

***tert*-butyl 2-(2-methylbenzyl)-3-oxobutanoate (11b)**

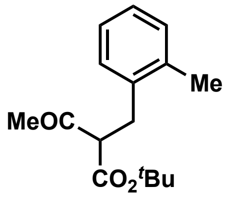

Physical state: colorless oil.

^{1}H NMR (400 MHz, $CDCl_3$) δ: 7.07 – 7.14 (m, 4H), 3.68 (t, *J* = 7.1 Hz, 1H), 3.07 – 3.17 (m, 2H), 2.32 (s, 3H), 2.19 (s, 3H), 1.39 (s, 9H). **^{13}C NMR (101 MHz, $CDCl_3$)** δ: 202.93, 168.58, 136.59, 136.28, 130.49, 129.39, 126.77, 126.05, 82.13, 60.79, 31.13, 29.51, 27.92, 19.54. **IR (thin film, cm^{-1})**: 3011, 2980, 1714, 1459, 1368, 1275, 1260, 1141 and 750. **HRMS (DART-TOF)** calculated for $C_{16}H_{22}NaO_3^+$ [M+Na]$^+$ m/z 285.1467, found 285.1466.

***tert*-butyl 2-(3-chlorobenzyl)-3-oxobutanoate (11c)**

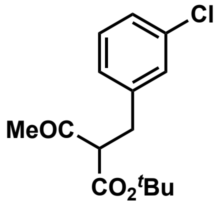

Physical state: colorless oil.

^{1}H NMR (400 MHz, $CDCl_3$) δ: 7.16 – 7.22 (m, 3H), 7.04 – 7.09 (m, 1H), 3.65 (t, *J* = 7.4 Hz, 1H), 3.04 – 3.13 (m, 2H), 2.21 (s, 3H), 1.40 (s, 9H). **^{13}C NMR (101 MHz, $CDCl_3$)** δ: 202.13, 168.03, 140.58, 134.23, 129.78, 129.07, 127.18, 126.81, 82.40, 62.10, 33.43, 29.40, 27.87. **IR (thin film, cm^{-1})**: 3007, 2983, 1714, 1596, 1476, 1368, 1275, 1260, 1140 and 749. **HRMS (DART-TOF)** calculated for $C_{15}H_{19}ClNaO_3^+$ [M+Na]$^+$ m/z 305.0920, found 305.0913.

tert-butyl 2-(4-bromobenzyl)-3-oxobutanoate (11d)

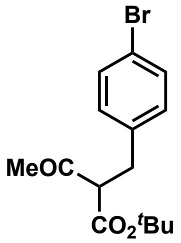

Physical state: white solid. **M.p.** = 41.2-43.0 °C.

^{1}H NMR (400 MHz, $CDCl_3$) δ: 7.39 (d, *J* = 8.4 Hz, 2H), 7.06 (d, *J* = 8.4 Hz, 2H), 3.63 (t, *J* = 7.6 Hz, 1H), 3.00 – 3.12 (m, 2H), 2.19 (s, 3H), 1.39 (s, 9H). **^{13}C NMR (101 MHz, $CDCl_3$)** δ: 202.40, 168.13, 137.58, 131.67, 130.77, 120.56, 82.44, 62.21, 33.31, 29.56, 27.97. **IR (thin film, cm^{-1})**: 3011, 2976, 1714, 1490, 1368, 1275, 1260, 1140, 1011 and 750. **HRMS (DART-TOF)** calculated for $C_{15}H_{19}BrNaO_3^+$ $[M+Na]^+$ m/z 349.0415, found 349.0417.

tert-butyl 2-((5-chloropyridin-3-yl)methyl)-3-oxobutanoate (11f)

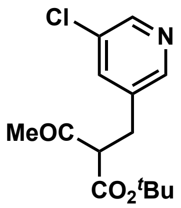

Physical state: pale yellow oil.

^{1}H NMR (400 MHz, $CDCl_3$) δ: 8.32 - 8.40 (m, 1H), 7.19 - 7.24 (m, 1H), 7.09 - 7.14 (m, 1H), 4.17 (t, *J* = 7.4 Hz, 1H), 3.17 - 3.38 (m, 2H), 2.32 (s, 3H), 1.43 (s, 9H). **^{13}C NMR (101 MHz, $CDCl_3$)** δ: 202.87, 168.18, 160.25, 149.92, 144.23, 123.97, 121.96, 82.23, 58.86, 35.36, 29.96, 27.95. **IR (thin film, cm^{-1})**: 2976, 2929, 1714, 1577, 1555, 1368, 1141, 846 and 763. **HRMS (DART-TOF)** calculated for $C_{14}H_{18}ClNNaO_3^+$ $[M+Na]^+$ m/z 306.0873, found 306.0872.

tert-butyl 3-oxo-2-((4-oxo-4H-chromen-3-yl)methyl)butanoate (11g)

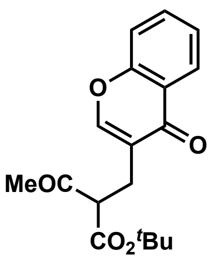

Physical state: white solid, **M.p.** = 87.5-89.1 °C.

^{1}H NMR (400 MHz, CDCl$_3$) δ: 8.17 – 8.25 (m, 1H), 7.89 (s, 1H), 7.71 – 7.61 (m, 1H), 7.37-7.47 (m, 2H), 4.05 (t, *J* = 7.3 Hz, 1H), 2.78-3.02 (m, 2H), 2.27 (s, 3H), 1.41 (s, 9H). **^{13}C NMR (101 MHz, CDCl$_3$)** δ: 203.13, 177.81, 168.24, 156.59, 154.27, 133.70, 125.81, 125.24, 123.89, 121.02, 118.29, 82.26, 58.16, 30.02, 28.03, 24.88. **IR (thin film, cm^{-1})**: 3005, 2992, 1718, 1645, 1466, 1275, 1260 and 750. **HRMS (DART-TOF)** calculated for $C_{18}H_{20}NaO_5^+$ $[M+Na]^+$ m/z 339.1208, found 339.1208.

tert-butyl 2-benzyl-3-oxopentanoate (11i)

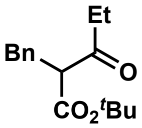

Physical state: colorless oil.

^{1}H NMR (400 MHz, CDCl$_3$) δ: 7.23 - 7.29 (m, 2H), 7.14 - 7.22 (m, 3H), 3.70 (t, *J* = 7.7 Hz, 1H), 3.05 - 3.17 (m, 2H), 2.48 - 2.61 (m, 1H), 2.29 - 2.39 (m, 1H), 1.38 (s, 9H), 1.01 (t, *J* = 7.2 Hz, 3H). **^{13}C NMR (101 MHz, CDCl$_3$)** δ: 205.80, 168.52, 138.64, 128.98, 128.56, 126.61, 82.07, 61.41, 36.05, 34.17, 27.96, 7.72. **IR (thin film, cm^{-1})**: 2978, 2936, 1712, 1455, 1368, 1255, 1145, 844 and 699. **HRMS (DART-TOF)** calculated for $C_{16}H_{22}NaO_3^+$ $[M+Na]^+$ m/z 285.1467, found 285.1464.

methyl 2-benzyl-4-methyl-3-oxopentanoate (11j)

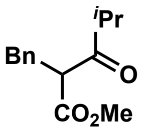

Physical state: colorless oil.

^{1}H NMR (400 MHz, $CDCl_3$) δ: 7.13 - 7.29 (m, 5H), 3.95 (t, *J* = 7.5 Hz, 1H), 3.68 (s, 3H), 3.16 (d, *J* = 7.6 Hz, 2H), 2.54 - 2.65 (m. 1H), 1.05 (d, *J* = 6.8 Hz, 3H), 0.87 (d, *J* = 7.0 Hz, 3H). **^{13}C NMR (101 MHz, $CDCl_3$)** δ: 208.46, 169.64, 138.48, 129.01, 128.65, 126.77, 58.63, 52.50, 41.47, 34.60, 17.86, 17.75. **IR (thin film, cm^{-1})**: 2972, 2875, 1744, 1711, 1454, 1436, 1207, 1163, 750 and 700. **HRMS (DART-TOF)** calculated for $C_{14}H_{18}NaO_3^+$ $[M+Na]^+$ m/z 257.1154, found 257.1161.

***tert*-butyl 2-acetyl-3-oxopentanoate (11m)**

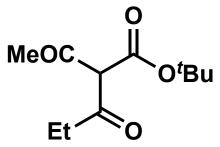

Physical state: colorless oil.

^{1}H NMR (400 MHz, $CDCl_3$) δ 2.66 (q, *J* = 7.4 Hz, 2H), 2.30 (d, *J* = 1.2 Hz, 3H), 1.55 (s, 9H), 1.17 (t, *J* = 7.4 Hz, 3H). **^{13}C NMR (101 MHz, $CDCl_3$)** δ 199.17, 193.92, 166.74, 110.26, 110.25, 81.55, 31.19, 28.27, 25.10, 9.73.

2-benzyl-1-cyclopropylbutane-1,3-dione (11n)

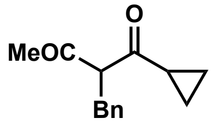

Physical state: white solid. **M.p.** = 54.2-57.0 °C.

^{1}H NMR (400 MHz, Chloroform-*d*) δ 7.30-7.24 (m, 2H), 7.23 – 7.14 (m, 3H), 4.11 (t, *J* = 7.4 Hz, 1H), 3.20 (d, *J* = 7.4 Hz, 2H), 2.15 (s, 3H), 2.04 – 1.89 (m, 1H), 1.07 – 0.97 (m, 2H), 0.96 – 0.82 (m, 2H). **^{13}C NMR (101 MHz, $CDCl_3$)** δ 205.86, 203.52, 138.42, 128.86, 128.72, 126.74, 70.43, 34.19, 29.62, 20.71, 12.37, 12.04. **HRMS (DART-TOF)** calculated for $C_{14}H_{16}NaO_2^+$ $[M + Na]^+$ m/z 239.1048, found 239.1039.

1-((1*r*,3*r*,5*r*,7*r*)-adamantan-2-yl) 3-methyl 2-benzylmalonate (11o)

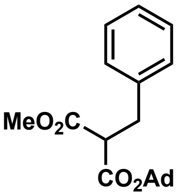

Physical state: colorless oil.

^{1}H NMR (400 MHz, CDCl$_3$) δ: 7.18 - 7.29 (m, 5H), 4.93 (br t, *J* = 3 Hz, 1H), 3.67 - 3.73 (m, 4H), 3.24 (d, *J* = 7.8 Hz, 2H), 1.69 - 2.02(m, 12H), 1.43 - 1.54(m, 2H). **^{13}C NMR (101 MHz, CDCl$_3$)** δ: 169.34, 167.93, 137.87, 128.68, 128.40, 126.60, 78.19, 53.90, 52.19, 37.21, 36.15, 36.14, 34.66, 31.64,31.63, 31.55, 31.48, 27.07, 26.86. **IR (thin film, cm^{-1})**: 3035, 2905, 2855, 1723, 1451, 1251, 1170, 1086, 738 and 698. **HRMS (DART-TOF)** calculated for $C_{21}H_{26}NaO_4^+$ [M+Na]$^+$ m/z 365.1729, found 365.1731.

1-((1*r*,3*r*,5*r*,7*r*)-adamantan-2-yl) 3-methyl 2-(4-methylbenzyl)malonate (11p)

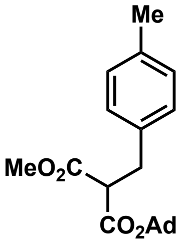

Physical state: white solid, **M.p.** = 38.2-40.1 °C.

^{1}H NMR (400 MHz, CDCl$_3$) δ: 7.04 - 7.12 (m, 4H), 4.94 (br t, *J* = 3 Hz, 1H), 3.64 - 3.72 (m, 4H), 3.20 (d, *J* = 7.8 Hz, 2H), 2.30 (s, 3H), 1.69 - 2.00 (m, 12H), 1.43 - 1.55 (m, 2H). **^{13}C NMR (101 MHz, CDCl$_3$)** δ: 169.64, 168.23, 136.28, 134.92, 129.26, 128.71, 78.37, 54.22, 52.40, 37.38, 36.33, 36.32 34.42, 31.80, 31.79, 31.72, 31.64, 27.22, 27.00, 21.10. **IR (thin film, cm^{-1})**: 2906, 2855, 1729, 1450, 1348, 1230, 1150, 1042, 763 and 751. **HRMS (DART-TOF)** calculated for $C_{22}H_{28}NaO_4^+$ [M+Na]$^+$ m/z 379.1885, found 379.1883.

1-((1*r*,3*r*,5*r*,7*r*)-adamantan-2-yl) 3-methyl 2-(3-chlorobenzyl)malonate (11q)

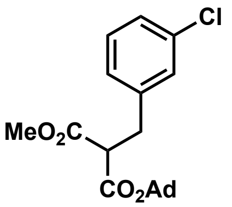

Physical state: colorless oil.

^{1}H NMR (400 MHz, $CDCl_3$) δ: 7.17 - 7.23 (m, 3H), 7.08 - 7.12 (m, 1H), 4.95 (br t, *J* = 3 Hz, 1H), 3.72 (s, 3H), 3.67 (t, *J* = 7.8 Hz, 1H), 3.21 (d, *J* = 7.8 Hz, 2H), 1.99 – 1.70 (m, 12H), 1.55 – 1.45 (m, 2H). **^{13}C NMR (101 MHz, $CDCl_3$)** δ: 169.10, 167.71, 139.95, 134.19, 129.72, 128.91, 126.97, 126.89, 78.46, 53.59, 52.37, 37.20, 36.16, 36.15, 34.25, 31.68, 31.65, 31.57, 31.47, 27.07, 26.85. **IR (thin film, cm^{-1})**: 2907, 2858, 1750, 1730, 1451, 1353, 1275, 1152 and 750. **HRMS (DART-TOF)** calculated for $C_{21}H_{25}ClNaO_4^+$ $[M+Na]^+$ m/z 399.1339, found 399.1334.

1-((1*r*,3*r*,5*r*,7*r*)-adamantan-2-yl) 3-methyl 2-allylmalonate (11r)

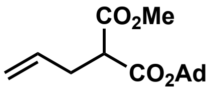

Physical state: colorless oil.

^{1}H NMR (400 MHz, $CDCl_3$) δ: 5.73 - 5.86 (m, 1H), 5.09 - 5.17 (m, 1H), 5.03 - 5.09 (m, 1H), 4.98 (br t, *J* = 3 Hz, 1H), 3.74 (s, 3H), 3.48 (t, *J* = 7.5 Hz, 1H), 2.63 - 2.71 (m, 2H), 1.72 - 2.02 (m, 12H), 1.49 – 1.56 (m, 2H). **^{13}C NMR (101 MHz, $CDCl_3$)** δ: 170.27, 168.85, 78.09, 52.31, 37.39, 36.34, 36.31, 31.88, 31.80, 31.76, 31.73, 29.56, 28.56, 27.25, 27.02, 22.40, 13.85. **IR (thin film, cm^{-1})**: 2907, 2856, 1730, 1451, 1228, 1156, 1100, 767 and 751. **HRMS (DART-TOF)** calculated for $C_{17}H_{24}NaO_4^+$ $[M+Na]^+$ m/z 315.1572, found 315.1569.

1-((1*r*,3*r*,5*r*,7*r*)-adamantan-2-yl) 3-methyl 2-butylmalonate (11s)

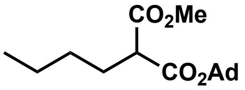

Physical state: colorless oil.

^{1}H NMR (400 MHz, $CDCl_3$) δ: 4.97 (br t, *J* = 3 Hz, 1H), 3.73 (s, 3H), 3.37 (t, *J* = 7.5 Hz, 1H), 1.71 - 2.03 (m, 14H), 1.52 – 1.62 (m, 2H), 1.28 - 1.40 (m, 4H), 0.90 (t, *J* = 6.8 Hz,

3H). **^{13}C NMR (101 MHz, $CDCl_3$)** δ: 170.22, 168.80, 78.05, 52.27, 52.21, 37.36, 36.31, 36.28, 31.85, 31.78, 31.73, 31.70, 29.52, 28.53, 27.22, 26.99, 22.37, 13.82. **IR (thin film, cm^{-1})**: 2906, 2856, 1729, 1451, 1275, 1260, 1155, 1100, 1042 and 751. **HRMS (DART-TOF)** calculated for $C_{18}H_{28}NaO_4^+$ $[M+Na]^+$ m/z 331.1885, found 331.1878.

1-((1*r*,3*r*,5*r*,7*r*)-adamantan-2-yl) 3-allyl 2-benzylmalonate (11t)

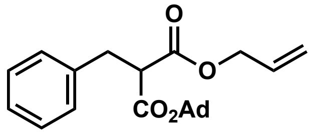

Physical state: colorless oil.

^{1}H NMR (400 MHz, $CDCl_3$) δ: 7.16 – 7.30 (m, 5H), 5.76 – 5.91 (m, 1H), 5.22 - 5.29 (m, 1H), 5.17 - 5.22 (m, 1H), 4.94 (br t, *J* = 3 Hz, 1H), 4.56 - 4.62 (m, 2H), 3.73 (t, *J* = 7.8 Hz, 1H), 3.25 (d, *J* = 7.8 Hz, 2H), 2.01 – 1.70 (m, 12H), 1.55 – 1.44 (m, 2H). **^{13}C NMR (101 MHz, $CDCl_3$)** δ: 168.83, 168.16, 138.03, 131.66, 128.93, 128.62, 126.82, 118.74, 78.50, 66.09, 54.22, 37.41, 36.36, 36.36, 34.85, 31.83, 31.81, 31.79, 31.71, 27.24, 27.03. **IR (thin film, cm^{-1})**: 3035, 2906, 2855, 1729, 1452, 1148, 980 and 699. **HRMS (DART-TOF)** calculated for $C_{23}H_{28}NaO_4^+$ $[M+Na]^+$ m/z 391.1885, found 391.1877.

1-((1*r*,3*r*,5*r*,7*r*)-adamantan-2-yl) 3-methyl 2-(3-(1,3-dioxoisoindolin-2-yl)propyl)malonate (11u)

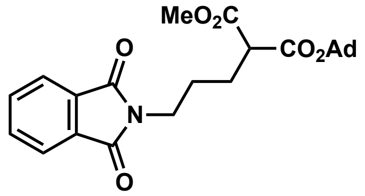

Physical state: colorless oil.

^{1}H NMR (400 MHz, $CDCl_3$) δ: 7.81 - 7.87 (m, 2H), 7.68 - 7.74 (m, 2H), 4.96 (br t, *J* = 3 Hz, 1H), 3.69 - 3.77 (m, 5H), 3.44 (t, *J* = 7.5 Hz, 1H), 1.72 – 2.02 (m, 16H), 1.47 – 1.55 (m, 2H). **^{13}C NMR (101 MHz, $CDCl_3$)** δ: 169.73, 168.27, 168.25, 133.94, 132.07, 123.22, 78.26, 52.36, 51.63, 37.32, 37.29, 36.25, 36.22, 31.76, 31.69, 31.67, 31.64, 27.14, 26.92, 26.32, 25.97. **IR (thin film, cm^{-1})**: 2907, 2854, 1709, 1396, 1260, 1040, 748 and 720. **HRMS (DART-TOF)** calculated for $C_{25}H_{29}NNaO_6^+$ $[M+Na]^+$ m/z 462.1893, found 462.1891.

adamantan-2-yl 2-cyano-3-phenylpropanoate (23)

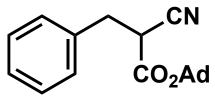

Physical state: colorless oil.

^{1}H NMR (400 MHz, $CDCl_3$) δ: 7.26 – 7.34 (m, 5H), 4.99 (br t, *J* = 3 Hz, 1H), 3.75 (dd, *J* = 8.1, 5.8 Hz, 1H), 3.16 – 3.34 (m, 2H), 1.69 – 2.01 (m, 12H), 1.48-1.56 (m, 2H). **^{13}C NMR (101 MHz, $CDCl_3$)** δ: 164.97, 135.44, 130.12, 129.15, 128.95, 128.61, 127.85, 116.44, 80.26, 40.02, 37.26, 36.30, 36.28, 35.97, 31.88, 31.84, 31.63, 31.58, 27.09, 26.88. **HRMS (DART-TOF)** calculated for $C_{20}H_{23}NNaO_2^+$ $[M+Na]^+$ m/z 332.1626, found 332.1633.

Optimization Tables

Supplementary Table 1. Screening of bases.

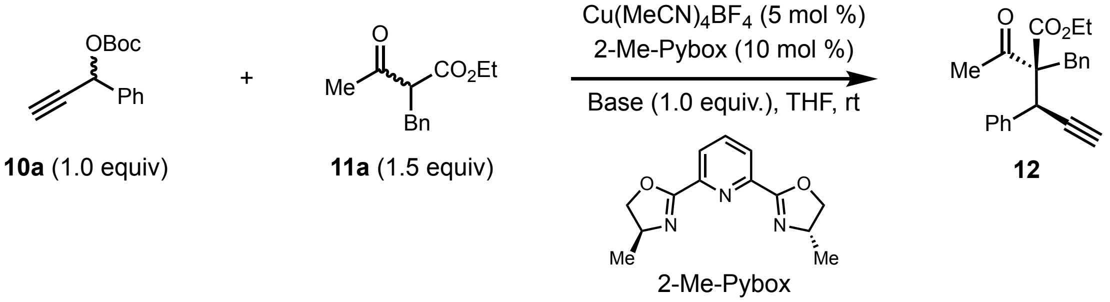

Entry	Base	Yield%	*dr*	*ee%*
1	K_2CO_3	15	1:1	5
2	K_3PO_4	18	1:1	4
3	DBU	N.D.	N.D.	N.D.
4	$CsCO_3$	28	1:1	3
5	TMG	45	1:1	0
6	$^{i}Pr_2NEt$	34	1.4:1	81

Reactions in this table were performed on a 0.1 mmol scale. **10a** (0.10 mmol), **11a** (0.15 mmol), $Cu(MeCN)_4BF_4$ (0.005 mmol), 2-Me-Pybox (0.01 mmol), base (0.10 mmol), THF (1 mL). Yields and *d.r.* values were determined by ^1H NMR spectroscopy of the crude reaction mixture with 1,1,2,2-tetrachloroethane as an internal standard. The *ee* values were determined by HPLC analysis.

Supplementary Table 2. Screening of Lewis acids.

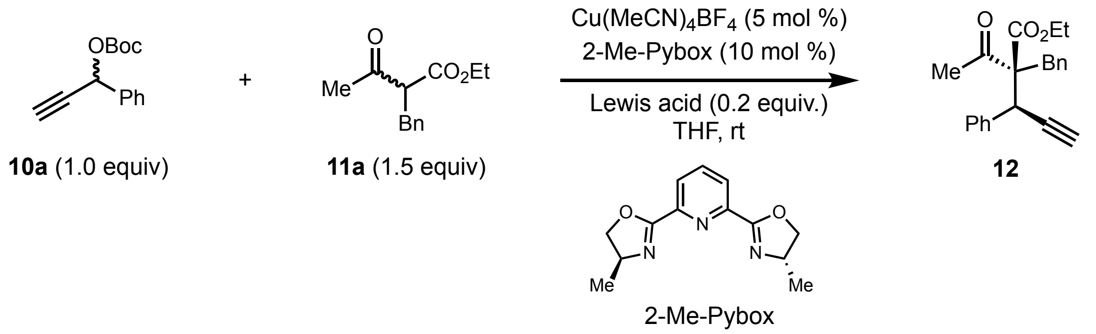

Entry	Base	Yield%	*dr*	*ee*%
1	$Mg(OEt)_2$	92	1.8:1	93
2	$Mg(^{n}Bu)_2$	trace	trace	trace
3	$Mg(O^{t}Bu)_2$	84	2.7:1	90
4	$Bi(OTf)_3$	N.D.	N.D.	N.D.
5	$Ti(O^{i}Pr)_4$	trace	trace	trace
6	$Sc(OTf)_2$	N.D.	N.D.	N.D.
7	$Mg(OTf)_2$	N.D.	N.D.	N.D.
8	$Fe(OTf)_3$	N.D.	N.D.	N.D.
9	$Zr(O^{t}Bu)_4$	36	2.1:1	83
10	$ZnEt_2$	56	1.5:1	51
11	$Al(O^{i}Pr)_3$	trace	trace	trace

Reactions in this table were performed on a 0.1 mmol scale. **10a** (0.10 mmol), **11a** (0.15 mmol), $Cu(MeCN)_4BF_4$ (0.005 mmol), 2-Me-Pybox (0.01 mmol), Lewis acid (0.02 mmol), THF (1 mL). Yields and *d.r.* values were determined by ^{1}H NMR spectroscopy of the crude reaction mixture with 1,1,2,2-tetrachloroethane as an internal standard. The *ee* values were determined by HPLC analysis.

Supplementary Table 3. Screening of Cu resources.

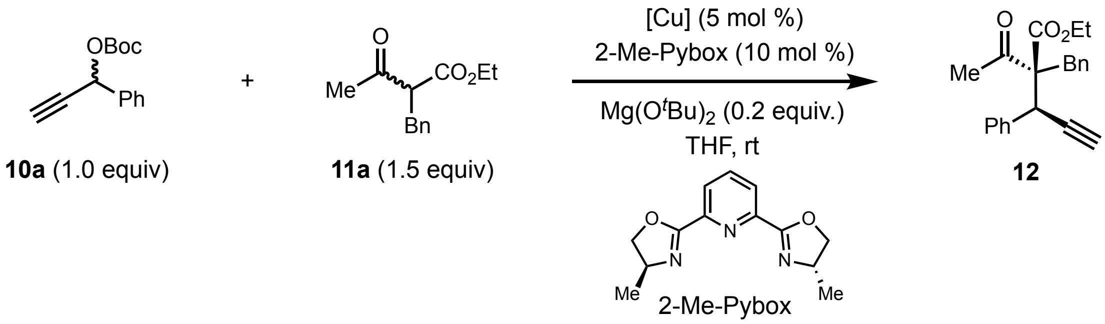

Entry	[Cu]	Yield%	*dr*	*ee*%
1	$CuSO_4$	40	1.5:1	47
2	$CuBr_2$	76	2.3:1	39
3	$Cu(OAc)_2$	40	1.4:1	22
4	$Cu(OTf)_2$	40	1.9:1	82
5	$Cu(MeCN)_4BF_4$	84	2.7:1	90
6	$Cu(MeCN)_4PF_6$	86	1.2:1	75
7	CuOTf	93	1.7:1	87
8	CuI	56	2.5:1	30

Reactions in this table were performed on a 0.1 mmol scale. **10a** (0.10 mmol), **11a** (0.15 mmol), [Cu] (0.005 mmol), 2-Me-Pybox (0.01 mmol), $Mg(O^tBu)_2$ (0.02 mmol), THF (1 mL). Yields and *d.r.* values were determined by ^1H NMR spectroscopy of the crude reaction mixture with 1,1,2,2-tetrachloroethane as an internal standard. The *ee* values were determined by HPLC analysis.

Supplementary Table 4. Screening of Ligands.

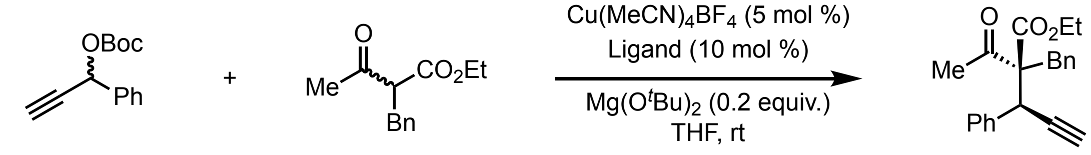

10a (1.0 equiv) **11a** (1.5 equiv) **12**

Entry	Ligand	Yield%	dr	ee%
1	**SI-L1**	84	2.7:1	90
2	**SI-L2**	trace	trace	trace
3	**SI-L3**	trace	trace	trace
4	**SI-L4**	28	3.8:1	53
5	**SI-L5**	65	2.6:1	95
6	**SI-L6**	trace	trace	trace
7	**SI-L7**	40	2.7:1	84
8	**SI-L8**	28	1.5:1	31
9	**SI-L9**	trace	trace	trace
10	**SI-L10**	24	2.3:1	10
11	**SI-L11**	trace	trace	trace
12	**SI-L12**	40	3.7:1	75
13	**SI-L13**	62	1.7:1	93
14	**SI-L14**	N.D.	N.D.	N.D.
15	**SI-L15**	35	6.0:1	38
16	**SI-L16**	93	11.0:1	97

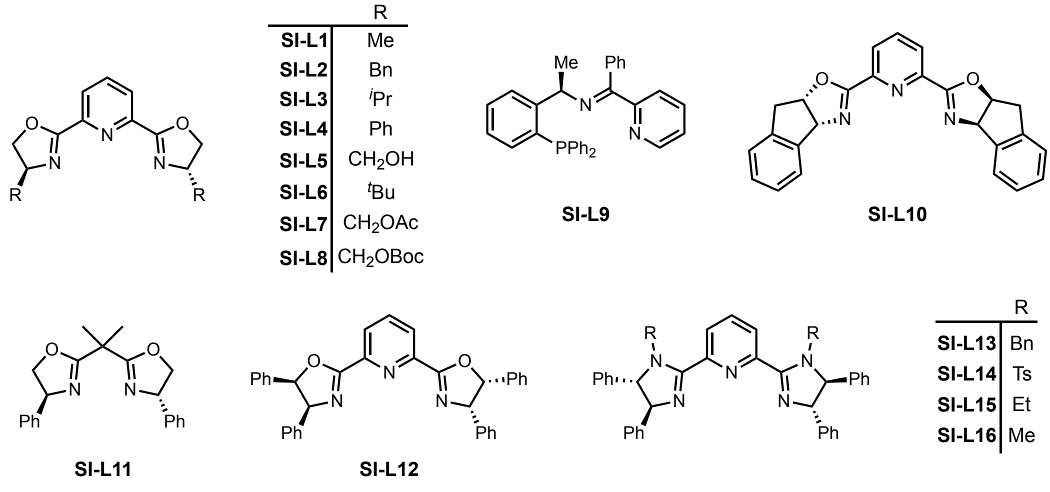

Reactions in this table were performed on a 0.1 mmol scale. **10a** (0.10 mmol), **11a** (0.15 mmol), $Cu(MeCN)_4BF_4$ (0.005 mmol), Ligand (0.01 mmol), $Mg(O^tBu)_2$ (0.02 mmol), THF (1 mL). Yields and *d.r.* values were determined by ^1H NMR spectroscopy of the crude reaction mixture with 1,1,2,2-tetrachloroethane as an internal standard. The *ee* values were determined by HPLC analysis.

Supplementary Table 5. Screening of solvents.

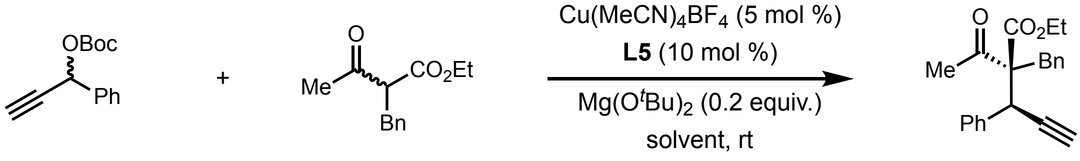

10a (1.0 equiv) **11a** (1.5 equiv) **12**

Entry	solvent	Yield%	*dr*	*ee*%
1	MeCN	96	11.0:1	93
2	1,4-dioxane	95	10.0:1	87
3	DCM	92	7.0:1	85
4	MTBE	91	6.1:1	55
5	DMF	90	3.8:1	93
6	CF_3Ph	70	7.8:1	77

Reactions in this table were performed on a 0.1 mmol scale. **10a** (0.10 mmol), **11a** (0.15 mmol), $Cu(MeCN)_4BF_4$ (0.005 mmol), **L5** (0.01 mmol), $Mg(O^tBu)_2$ (0.02 mmol), solvent (1 mL). Yields and *d.r.* values were determined by ^1H NMR spectroscopy of the crude reaction mixture with 1,1,2,2-tetrachloroethane as an internal standard. The *ee* values were determined by HPLC analysis.

General Procedures for the Cu/Mg-catalyzed Propargylation

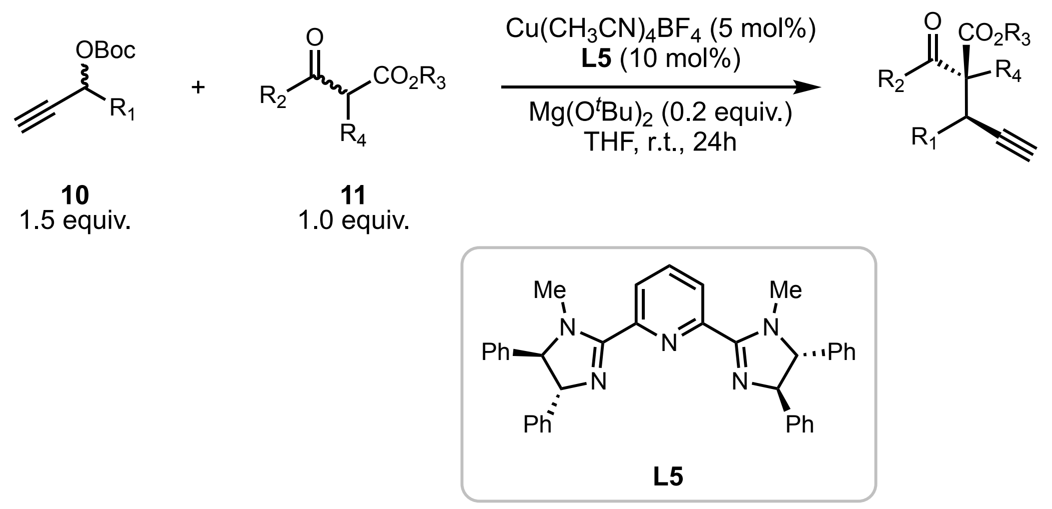

In a N_2-filled glovebox, $Cu(CH_3CN)_4BF_4$ (3.2 mg, 0.01 mmol, 5.0 mol%), **L5** (11.0 mg, 0.02 mmol, 10 mol%), and THF (2 mL) were sequentially added to a 8 mL screw cap vial containing a magnetic stirring bar which was stirring at room temperature for 1 h. Then, propargyl carbonate **10** (0.30 mmol, 1.5 equiv), 1,3-dicarbonyl compounds **11** (0.20 mmol, 1.0 equiv) and $Mg(O^tBu)_2$ (6.8 mg, 0.04 mmol, 0.2 equiv) were added sequentially, then the vial was tightly capped, transferred out of the glovebox, and stirred at room temperature for 24 h. After completion, the reaction mixture was quenched with water, extracted with EtOAc, dried over anhydrous Na_2SO_4 and concentrated under vacuum. Then, the reaction mixture was charged with 1,1,2,2-tetrachloroethane (22 uL, 1.0 equiv.) as an internal standard and the crude mixture was subjected to ^{1}H NMR analysis to determine the NMR yield as well as the *dr* values. Finally, the combined crude products were purified by flash chromatography, and the obtained title product was dried under vacuum, weighed, and sampled for HPLC analysis.

Gram-Scale Propargylation (4 mmol)

In a N_2-filled glovebox, $Cu(CH_3CN)_4BF_4$ (188 mg, 0.6 mmol, 15 mol%)*,* **L5** (657 mg, 1.2 mmol, 30 mol%), and THF (40 mL) were sequentially added to a 50 mL round bottle flask containing a magnetic stirring bar which was stirring at room temperature for 1 h. Then, *tert*-butyl (1-phenylprop-2-yn-1-yl) carbonate (1393 mg, 6.0 mmol, 1.5 equiv), 1,3-dicarbonyl compound **11o** (1370 mg, 4.0 mmol, 1.0 equiv) and $Mg(O^tBu)_2$ (341 mg, 2.0 mmol, 0.5 equiv) were added sequentially, then the vial was tightly capped, transferred out of the glovebox, and stirred at room temperature for 24 h. After completion, the reaction mixture was quenched with water, extracted with EtOAc, dried over anhydrous Na_2SO_4 and concentrated under vacuum. Then, the reaction mixture was charged with 1,1,2,2-tetrachloroethane (423 uL, 1.0 equiv.) as an internal standard and the crude mixture was subjected to ^1H NMR analysis to determine the NMR yield as well as the d.r. values. Finally, the combined crude products were purified by flash chromatography (silica gel, PE: EtOAc = 30:1) as a colorless oil (78mg, 85% yield, d.r. >19:1, 97% *ee*).

1-(adamantan-2-yl) 3-methyl (*S*)-2-benzyl-2-((*S*)-1-phenylprop-2-yn-1-yl)malonate

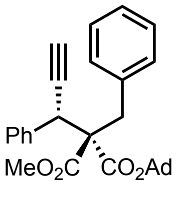

15o

^{1}H NMR (400 MHz, $CDCl_3$) δ: 7.40 - 7.45 (m, 2H), 7.20 - 7.30 (m, 8H), 4.85 (br t, *J* = 3 Hz, 1H), 4.48 (d, *J* = 2.7 Hz, 1H), 3.67 (d, *J* = 13.7 Hz, 1H), 3.61 (s, 3H), 3.29 (d, *J* = 13.8 Hz, 1H), 2.44 (d, *J* = 2.7 Hz, 1H), 1.58 - 1.83 (m, 12H), 1.33 - 1.43 (m, 2H). **^{13}C NMR (101 MHz, $CDCl_3$)** δ: 169.72, 168.53, 136.67, 136.27, 130.31, 130.02, 128.21, 128.01, 127.80, 127.11, 83.44, 78.82, 73.87, 64.08, 51.87, 42.57, 40.48, 37.36, 36.38, 36.28, 31.72, 31.66, 31.63, 31.59, 27.16, 27.00. **HPLC** analysis (AD-H, Hexanes: ^{i}PrOH = 98:2, 1 mL/min) indicated 97% *ee*: t_R (major) = 11.3 min, t_R (minor) = 10.5 min. **IR (thin film, cm^{-1})**: 3294, 3005, 2992, 2905, 1724, 1451, 1275, 1260 and 752. **HRMS (DART-TOF)** calculated for $C_{30}H_{32}NaO_4^+$ $[M+Na]^+$ m/z 479.2198, found 479.2195. **$[\alpha]_D^{20}$**= -4.5 (c = 0.22, $CHCl_3$).

Characterization Data for Propargylation Products

ethyl (2*S*,3*S*)-2-acetyl-2-benzyl-3-phenylpent-4-ynoate

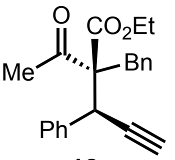

12

Product **12** was prepared following **General Procedure** from ethyl 2-benzyl-3-oxobutanoate (44.9 mg, 0.20 mmol, 1.0 equiv), *tert*-butyl (1-phenylprop-2-yn-1-yl) carbonate (68.8mg, 0.30 mmol, 1.5 equiv), $Cu(CH_3CN)_4BF_4$ (3.2 mg, 0.01 mmol, 5.0 mol%), **L5** (5.4 mg, 0.02 mmol, 10 mol%), $Mg(O^tBu)_2$ (6.8 mg, 0.04 mmol, 0.2 equiv.) and isolated by flash chromatography (silica gel, PE: EtOAc = 30:1) as a pale yellow oil (63 mg, 94%, d.r. = 11:1).

^{1}H NMR (400 MHz, $CDCl_3$) δ: 7.34 – 7.16 (m, 10H), 4.61 (d, *J* = 2.7 Hz, 1H), 4.12 – 3.92 (m, 2H), 3.43 (d, *J* = 13.6 Hz, 1H), 3.31 (d, *J* = 13.7 Hz, 1H), 2.47 (d, *J* = 2.7 Hz, 1H), 2.04 (s, 3H), 1.05 (t, *J* = 7.2 Hz, 3H). **^{13}C NMR (101 MHz, $CDCl_3$)** δ: 204.01, 169.99, 136.57, 136.30, 130.33, 129.71, 128.35, 128.11, 127.83, 127.11, 83.40, 74.30, 69.52, 61.37, 42.70, 40.60, 31.30, 13.71. **HPLC analysis** (OJ-H, Hexanes: ^{i}PrOH = 99:1, 1 mL/min) indicated 95% *ee*: t_R (major) = 18.4 min, t_R (minor) = 24.6 min. **IR (thin film, cm^{-1})**: 3005, 2984, 1706, 1455, 1275, 1260 and 749. **HRMS (DART-TOF)** calculated for $C_{22}H_{22}NaO_3^+$ $[M+Na]^+$ m/z 357.1467, found 357.1465. **$[\alpha]_D^{21}$**= -35.5 (c = 0.31, $CHCl_3$).

***tert*-butyl (2*S*,3*S*)-2-acetyl-2-benzyl-3-(4-(trifluoromethyl)phenyl)pent-4-ynoate**

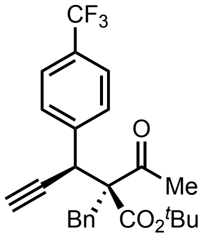

14a

Product **14a** was prepared following **General Procedure** from *tert*-butyl 2-benzyl-3-oxobutanoate (50.3 mg, 0.20 mmol, 1.0 equiv), *tert*-butyl (1-(4-(trifluoromethyl)phenyl)prop-2-yn-1-yl) carbonate (90.8mg, 0.30 mmol, 1.5 equiv), $Cu(CH_3CN)_4BF_4$ (3.2 mg, 0.01 mmol, 5.0 mol%), **L5** (5.4 mg, 0.02 mmol, 10 mol%), $Mg(O^tBu)_2$ (6.8 mg, 0.04 mmol, 0.2 equiv.) and isolated by flash chromatography (silica gel, PE: EtOAc = 30:1) as a pale yellow oil (70 mg, 82%, d.r. >19:1).

^{1}H NMR (400 MHz, $CDCl_3$) δ: 7.49 - 7.54 (m, 4H), 7.18 - 7.26 (m, 5H), 4.64 (d, *J* = 2.7 Hz, 1H), 3.44 (d, *J* = 13.9 Hz, 1H), 3.32 (d, *J* = 13.9 Hz, 1H), 2.53 (d, *J* = 2.7 Hz, 1H), 2.04 (s, 3H), 1.32 (s, 9H). **^{13}C NMR (101 MHz, $CDCl_3$)** δ: 203.98, 168.63, 140.89, 135.94, 130.77, 130.47, 129.85 (q, *J* = 32.5 Hz), 128.41, 127.26, 124.80 (q, *J* = 3.8

Hz), 124.19 (q, J = 272.7 Hz), 83.39, 82.68, 75.07, 69.56, 41.95, 40.84, 30.99, 27.82. **^{19}F NMR (376 MHz, $CDCl_3$)** δ: -62.64. **HPLC analysis** (AD-H, Hexanes: ^{i}PrOH = 99:1, 1 mL/min) indicated 84% *ee*: t_R (major) =9.3 min, t_R (minor) = 12.3 min. **IR (thin film, cm^{-1}):** 3286, 3005, 2989, 1710, 1459, 1325, 1275, 1260, 1069 and 751. **HRMS (DART-TOF)** calculated for $C_{25}H_{25}F_3NaO_3^+$ [M + Na]$^+$ m/z 453.1653, found 453.1657. **$[\alpha]_D^{25}$**= -16.2 (c = 0.20, $CHCl_3$).

***tert*-butyl (2*S*,3*S*)-2-acetyl-2-benzyl-3-(4-fluorophenyl)pent-4-ynoate**

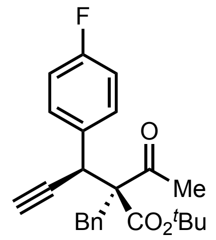

14b

Product **14b** was prepared following **General Procedure** from *tert*-butyl 2-benzyl-3-oxobutanoate (49.3 mg, 0.20 mmol, 1.0 equiv), *tert*-butyl (1-(4-fluorophenyl)prop-2-yn-1-yl) carbonate (74.6 mg, 0.30 mmol, 1.5 equiv), $Cu(CH_3CN)_4BF_4$ (3.2 mg, 0.01 mmol, 5.0 mol%), **L5** (5.4 mg, 0.02 mmol, 10 mol%), $Mg(O^tBu)_2$ (6.8 mg, 0.04 mmol, 0.2 equiv.) and isolated by flash chromatography (silica gel, PE: EtOAc = 30:1) as a colorless oil (61 mg, 80 %, d.r. >19:1).

^{1}H NMR (400 MHz, $CDCl_3$) δ: 7.30 - 7.35 (m, 2H), 7.17 - 7.25 (m, 5H), 6.92 - 6.98 (m, 2H), 4.56 (d, J = 2.7 Hz, 1H), 3.41 (d, J = 13.8 Hz, 1H), 3.26 (d, J = 13.8 Hz, 1H), 2.49 (d, J = 2.7 Hz, 1H), 2.06 (s, 3H), 1.33 (s, 9H). **^{13}C NMR ($CDCl_3$, 101 MHz)** δ: 204.32, 168.88, 162.30 (d, J = 246.6 Hz), 136.27, 132.37(d, J = 3.3 Hz), 131.86 (d, J = 8.1 Hz), 130.51, 128.33, 127.12, 114.82 (21.4 Hz), 83.38, 83.16, 74.57, 69.63, 41.83, 40.81, 31.24, 27.87. **^{19}F NMR (376 MHz, $CDCl_3$)** δ: -114.91. **HRMS (DART-TOF)** calculated for $C_{24}H_{25}$ $FNaO_3^+$ [M + Na]$^+$ m/z 403.1685, found 403.1681. **HPLC analysis** (OJ-H, Hexanes: ^{i}PrOH = 99:1, 1 mL/min) indicated 98% *ee*: t_R (major) = 11.3 min, t_R (minor) = 9.6 min. **IR (thin film, cm^{-1}):** 3290, 3005, 2987, 1705, 1508, 1369, 1354, 1275, 1260 and 750. **$[\alpha]_D^{21}$**= -26.0 (c = 0.23, $CHCl_3$).

***tert*-butyl (2*S*,3*S*)-2-acetyl-2-benzyl-3-(4-bromophenyl)pent-4-ynoate**

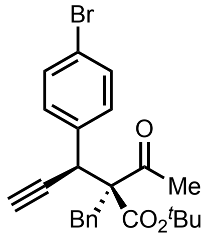

14c

Product **14c** was prepared following **General Procedure** from *tert*-butyl 2-benzyl-3-oxobutanoate (49.1 mg, 0.20 mmol, 1.0 equiv), 1-(4-bromophenyl)prop-2-yn-1-yl tert-butyl carbonate (93.0mg, 0.30 mmol, 1.5 equiv), $Cu(CH_3CN)_4BF_4$ (3.2 mg, 0.01 mmol, 5.0 mol%), **L5** (5.4 mg, 0.02 mmol, 10 mol%), $Mg(O^tBu)_2$ (6.8 mg, 0.04 mmol, 0.2 equiv.) and isolated by flash chromatography (silica gel, PE: EtOAc = 30:1) as a pale yellow oil (74 mg, 84%, d.r. >19:1).

^{1}H NMR (400 MHz, $CDCl_3$) δ: 7.36 - 7.41 (m, 2H), 7.18 - 7.26 (m, 7H), 4.53 (d, *J* = 2.7 Hz, 1H), 3.41 (d, *J* = 13.8 Hz, 1H), 3.26 (d, *J* = 13.8 Hz, 1H), 2.49 (d, *J* = 2.7 Hz, 1H), 2.05 (s, 3H), 1.33 (s, 9H). **^{13}C NMR (101 MHz, $CDCl_3$)** δ: 204.15, 168.76, 136.14, 135.72, 131.97, 131.05, 130.49, 128.34, 127.15, 121.80, 83.26, 83.01, 74.70, 69.50, 41.92, 40.77, 31.15, 27.88. **HPLC analysis** (AD-H, Hexanes: ^{i}PrOH = 99:1, 1 mL/min) indicated 94% *ee*: t_R (major) = 19.8 min, t_R (minor) =30.5 min. **IR (thin film, cm^{-1}):** 3292, 2979, 2929, 1704, 1488, 1368, 1275, 1260, 1148, 1012 and 750. **HRMS (DART-TOF)** calculated for $C_{24}H_{25}BrNaO_3^+$ [M + Na]$^+$ m/z 463.0885, found 463.0883. **$[\alpha]_D^{21}$**= -19.3 (c = 0.28, $CHCl_3$).

***tert*-butyl (2*S*,3*S*)-2-acetyl-2-benzyl-3-(4-(4,4,5,5-tetramethyl-1,3,2-dioxaborolan-2-yl)phenyl)pent-4-ynoate**

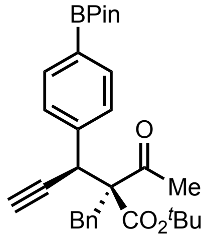

14d

Product **14d** was prepared following **General Procedure** from *tert*-butyl 2-benzyl-3-oxobutanoate (49.0 mg, 0.20 mmol, 1.0 equiv), *tert*-butyl (1-(4-(4,4,5,5-tetramethyl-1,3,2-dioxaborolan-2-yl)phenyl)prop-2-yn-1-yl) carbonate (107.9 mg, 0.30 mmol, 1.5 equiv), $Cu(CH_3CN)_4BF_4$ (3.2 mg, 0.01 mmol, 5.0 mol%), **L5** (5.4 mg, 0.02 mmol, 10 mol%), $Mg(O^tBu)_2$ (6.8 mg, 0.04 mmol, 0.2 equiv.) and isolated by flash chromatography (silica gel, PE: EtOAc = 30:1) as a colorless oil (69 mg, 71%, d.r. >19:1).

^{1}H NMR (400 MHz, $CDCl_3$) δ: 7.69 - 7.74 (m, 2H), 7.32 - 7.37 (m, 2H), 7.16 -7.23 (m, 5H), 4.54 (d, *J* = 2.7 Hz, 1H), 3.41 (d, *J* = 13.7 Hz, 1H), 3.19 (d, *J* = 13.7 Hz, 1H), 2.46 (d, *J* = 2.7 Hz, 1H), 2.11 (s, 3H), 1.32 (s, 21H). **^{13}C NMR (101 MHz, $CDCl_3$)** δ: 204.20, 168.94, 139.47, 136.51, 134.51, 130.59, 129.41, 128.20, 126.96, 83.85, 83.44, 83.05,

74.27, 69.52, 43.16, 40.67, 31.38, 27.86, 24.97, 24.94. **HPLC analysis** (AD-H, Hexanes: ^{i}PrOH = 99:1, 1 mL/min) indicated >99% *ee*: t_R (major) = 10.6 min, t_R (minor) = 19.6 min. **IR (thin film, cm^{-1}):** 3300, 2979, 2931, 1703, 1611, 1397, 1358, 1275, 1260, 1142, 1089 and 749. **HRMS (DART-TOF)** calculated for $C_{30}H_{37}BNaO_5^+$ [M + Na]+ m/z 511.2632, found 511.2633. **[α]$_D^{21}$**= -19.1 (c = 0.34, $CHCl_3$).

***tert*-butyl (2*S*,3*S*)-2-acetyl-2-benzyl-3-(4-methoxyphenyl)pent-4-ynoate**

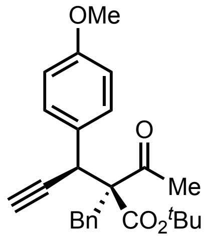

14e

Product **14e** was prepared following **General Procedure** from *tert*-butyl 2-benzyl-3-oxobutanoate (49.3 mg, 0.20 mmol, 1.0 equiv), *tert*-butyl (1-(4-methoxyphenyl)prop-2-yn-1-yl) carbonate (78.5mg, 0.30 mmol, 1.5 equiv), $Cu(CH_3CN)_4BF_4$ (3.2 mg, 0.01 mmol, 5.0 mol%), **L5** (5.4 mg, 0.02 mmol, 10 mol%), $Mg(O^tBu)_2$ (6.8 mg, 0.04 mmol, 0.2 equiv.) and isolated by flash chromatography (silica gel, PE: EtOAc = 30:1) as a white solid (67 mg, 85%, d.r. >19:1).

^{1}H NMR (400MHz, $CDCl_3$) δ: 7.15 - 7.26 (m, 7H), 6.77 - 6.83 (m, 2H), 4.50 (d, *J* = 2.7 Hz, 1H), 3.78 (s, 3H), 3.40 (d, *J* = 13.7 Hz, 1H), 3.18 (d, *J* = 13.7 Hz, 1H), 2.46 (d, *J* = 2.7 Hz, 1H), 2.12 (s, 3H), 1.32 (s, 9H). **^{13}C NMR (101 MHz, $CDCl_3$)** δ: 204.49, 169.11, 159.13, 136.64, 131.11, 130.58, 128.39, 128.20, 126.93, 113.45, 83.93, 82.92, 74.02, 69.67, 55.32, 42.27, 40.69, 31.43, 27.87. **HPLC analysis** (AD-H, Hexanes: ^{i}PrOH = 99:1, 1 mL/min) indicated 98% *ee*: t_R (major) = 31.9 min, t_R (minor) = 48.2 min. **IR (thin film, cm^{-1}):** 3285, 2987, 2932, 1703, 1512, 1455, 1369, 1275, 1261, 1149 and 750. **HRMS (DART-TOF)** calculated for $C_{25}H_{28}NaO_4^+$ [M + Na]$^+$ m/z 415.1885, found 415.1885. **[α]$_D^{21}$**= -27.0 (c = 0.23, $CHCl_3$). **M.p.:** 101.2-103.4 °C.

***tert*-butyl (2*S*,3*S*)-2-acetyl-2-benzyl-3-(*o*-tolyl)pent-4-ynoate**

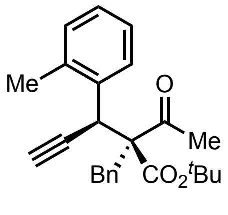

14f

Product **14f** was prepared following **General Procedure** from *tert*-butyl 2-benzyl-3-oxobutanoate (49.5 mg, 0.20 mmol, 1.0 equiv), *tert*-butyl (1-(o-tolyl)prop-2-yn-1-yl) carbonate (73.4 mg, 0.30 mmol, 1.5 equiv), $Cu(CH_3CN)_4BF_4$ (3.2 mg, 0.01 mmol, 5.0 mol%), **L5** (5.4 mg, 0.02 mmol, 10 mol%), $Mg(O^tBu)_2$ (6.8 mg, 0.04 mmol, 0.2 equiv.) and isolated by flash chromatography (silica gel, PE: EtOAc = 30:1) as a colorless oil (51 mg, 68%, d.r >19:1).

^{1}H NMR (400 MHz, $CDCl_3$) δ: 7.24 - 7.30 (m, 1H), 7.12 - 7.19 (m, 6H), 7.05 - 7.09 (m, 2H), 4.65 (d, *J* = 2.7 Hz, 1H), 3.45 (d, *J* = 13.7 Hz, 1 H), 3.09 (d, *J* = 13.7 Hz, 1H), 2.34 (d, *J* = 2.9 Hz, 1H), 2.30 (s, 3H), 2.28 (s, 3H), 1.32 (s, 9H). **^{13}C NMR (101 MHz, $CDCl_3$)** δ: 204.76, 169.54, 136.74, 136.70, 135.53, 130.89, 130.77, 128.98, 128.09, 127.75, 126.87, 126.26, 84.40, 83.20, 73.08, 69.07, 39.09, 37.97, 31.48, 27.78, 20.19. **HPLC analysis** (AD-H, Hexanes: ^{i}PrOH = 99:1, 1 mL/min) indicated 83% *ee*: t_R (major) = 11.1 min, t_R (minor) = 12.5 min. **IR (thin film, cm^{-1})**: 3011, 2984, 1702, 1459, 1275, 1260, 1149 and 749. **HRMS (DART-TOF)** calculated for $C_{25}H_{28}NaO_3^+$ [M + Na]+ m/z 399.1936, found 399.1939. **$[\alpha]_D^{21}$**= -9.1 (c = 0.23, $CHCl_3$).

***tert*-butyl (2*S*,3*S*)-2-acetyl-2-benzyl-3-(3-cyanophenyl)pent-4-ynoate**

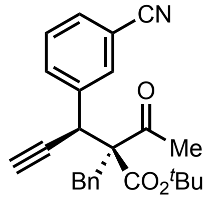

14g

Product **14g** was prepared following **General Procedure** from *tert*-butyl 2-benzyl-3-oxobutanoate (49.7 mg, 0.20 mmol, 1.0 equiv), *tert*-butyl (1-(3-cyanophenyl)prop-2-yn-1-yl) carbonate (77.0mg, 0.30 mmol, 1.5 equiv), $Cu(CH_3CN)_4BF_4$ (3.2 mg, 0.01 mmol, 5.0 mol%), **L5** (5.4 mg, 0.02 mmol, 10 mol%), $Mg(O^tBu)_2$ (6.8 mg, 0.04 mmol, 0.2 equiv.) and isolated by flash chromatography (silica gel, PE: EtOAc = 30:1) as a colorless oil (69 mg, 89%, d.r. 11:1).

^{1}H NMR (400 MHz, $CDCl_3$) δ: 7.70 - 7.74 (m, 1H), 7.59 - 7.64 (m, 1H), 7.50 - 7.55 (m, 1H), 7.32 - 7.38 (m, 1H), 7.16 - 7.26 (m, 5H), 4.59 (d, *J* = 2.7 Hz, 1H), 3.47 (d, *J* = 14.0 Hz, 1H), 3.36 (d, *J* = 14.0 Hz, 1H), 2.56 (d, *J* = 2.6 Hz, 1H), 2.01 (s, 3 H), 1.36 (s, 9H). **^{13}C NMR (101 MHz, $CDCl_3$)** δ: 203.73, 168.44, 138.58, 135.58, 135.08, 134.21, 131.17, 130.38, 128.59, 128.48, 127.39, 118.79, 111.99, 83.60, 82.19, 75.48, 69.56, 41.28, 40.73, 30.64, 27.90. **HPLC analysis** (AD-H, Hexanes: ^{i}PrOH = 99:1, 1 mL/min) indicated 96% *ee*: t_R (major) = 36.1 min, t_R (minor) = 45.3 min. **IR (thin film, cm^{-1}):** 3289, 3005, 2989, 1708, 1457, 1369, 1275, 1260 1148 and 750. **HRMS (DART-TOF)** calculated for $C_{25}H_{25}NNaO_3^+$ [M + Na]$^+$ m/z 410.1732, found 410.1736. **$[\alpha]_D^{20}$** = -19.2 (c = 0.39, $CHCl_3$).

tert-butyl (2_S_,3_S_)-2-acetyl-2-benzyl-3-(3-chlorophenyl)pent-4-ynoate

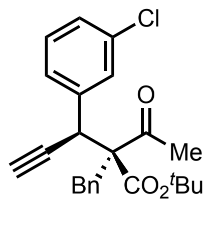

14h

Product **14h** was prepared following **General Procedure** from *tert*-butyl 2-benzyl-3-oxobutanoate (48.5 mg, 0.20 mmol, 1.0 equiv), *tert*-butyl (1-(3-chlorophenyl)prop-2-yn-1-yl) carbonate (79.8 mg, 0.30 mmol, 1.5 equiv), $Cu(CH_3CN)_4BF_4$ (3.2 mg, 0.01 mmol, 5.0 mol%), **L5** (5.4 mg, 0.02 mmol, 10 mol%), $Mg(O^tBu)_2$ (6.8 mg, 0.04 mmol, 0.2 equiv.) and isolated by flash chromatography (silica gel, PE: EtOAc = 30:1) as a colorless oil (70 mg, 88%, d.r. >19:1).

^{1}H NMR (400 MHz, $CDCl_3$) δ: 7.38 - 7.41 (m, 1H), 7.15 - 7.26 (m, 8H), 4.56 (d, *J* = 2.7 Hz, 1H), 3.43 (d, *J* = 13.8 Hz, 1H), 3.33 (d, *J* = 13.8 Hz, 1H), 2.52 (d, *J* = 2.7 Hz, 1H), 2.01 (s, 3H), 1.33 (s, 9H). **^{13}C NMR (101 MHz, $CDCl_3$)** δ: 204.04, 168.63, 138.80, 136.06, 133.70, 130.49, 130.37, 129.14, 128.52, 128.36, 127.80, 127.19, 83.33, 82.78, 74.94, 69.57, 41.87, 40.84, 31.03, 27.84. **HPLC analysis** (AD-H, Hexanes: ^{i}PrOH = 99:1, 1 mL/min) indicated 96% *ee*: t_R (major) = 16.3 min, t_R (minor) = 10.3 min. **IR (thin film, cm^{-1}):** 3293, 3005, 2987, 1707, 1476, 1369, 1275, 1260, 1148 and 750. **HRMS (DART-TOF)** calculated for $C_{24}H_{25}ClNaO_3^+$ $[M + Na]^+$m/z 419.1390, found 419.1388. **$[\alpha]_D^{21}$**= -32.0 (c = 0.29, $CHCl_3$).

tert-butyl (2_S_,3_S_)-2-acetyl-2-benzyl-3-(naphthalen-2-yl)pent-4-ynoate

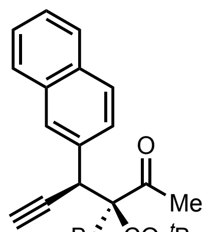

14i

Product **14i** was prepared following **General Procedure** from *tert*-butyl 2-benzyl-3-oxobutanoate (49.9 mg, 0.20 mmol, 1.0 equiv), *tert*-butyl (1-(naphthalen-2-yl)prop-2-yn-1-yl) carbonate (84.1mg, 0.30 mmol, 1.5 equiv), $Cu(CH_3CN)_4BF_4$ (3.2 mg, 0.01 mmol, 5.0 mol%), **L5** (5.4 mg, 0.02 mmol, 10 mol%), $Mg(O^tBu)_2$ (6.8 mg, 0.04 mmol, 0.2 equiv.) and isolated by flash chromatography (silica gel, PE: EtOAc = 30:1) as a white solid (74 mg, 90%, d.r. >19:1).

^{1}H NMR (400 MHz, $CDCl_3$) δ: 7.72 - 7.84 (m, 4H), 7.40 - 7.49 (m, 3H), 7.17 - 7.25 (m, 5H), 4.74 (d, *J* = 2.7 Hz, 1H), 3.47 (d, *J* = 13.8 Hz, 1H), 3.32 (d, *J* = 13.8 Hz, 1H), 2.53 (d, *J* = 2.7 Hz, 1H). 2.10 (s, 3H), 1.27 (s, 9H). **^{13}C NMR (101 MHz, $CDCl_3$)** δ: 204.43, 169.03, 136.50, 133.99, 133.02, 132.85, 130.61, 129.38, 128.28, 128.10, 127.77, 127.64, 127.55, 127.04, 126.17, 126.16, 83.63, 83.07, 74.53, 69.70, 42.92, 40.87, 31.38, 27.82. **HPLC analysis** (AD-H, Hexanes: ^{i}PrOH = 99:1, 1 mL/min) indicated 95%

ee: t_R (major) = 35.8 min, t_R (minor) = 27.6 min. **IR (thin film, cm^{-1})**: 3285, 3005, 2987, 1706, 1455, 1368, 1275, 1260, 1149, and 750. **HRMS (DART-TOF)** calculated for $C_{28}H_{28}NaO_3^+$ $[M + Na]^+$ m/z 435.1936, found 435.1932. $[\alpha]_D^{21}$= -23.5 (c = 0.20, $CHCl_3$). **M.p.:** 71.5-73.1 °C.

***tert*-butyl (2*S*,3*S*)-2-acetyl-2-benzyl-3-(furan-3-yl)pent-4-ynoate**

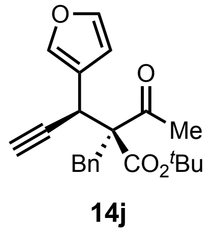

14j

Product **14j** was prepared following **General Procedure** from *tert*-butyl 2-benzyl-3-oxobutanoate (49.0 mg, 0.20 mmol, 1.0 equiv), *tert*-butyl (1-(furan-3-yl)prop-2-yn-1-yl) carbonate (66.1 mg, 0.30 mmol, 1.5 equiv), $Cu(CH_3CN)_4BF_4$ (3.2 mg, 0.01 mmol, 5.0 mol%), **L5** (5.4 mg, 0.02 mmol, 10 mol%), $Mg(O^tBu)_2$ (6.8 mg, 0.04 mmol, 0.2 equiv.) and isolated by flash chromatography (silica gel, PE: EtOAc = 30:1) as a colorless oil (58 mg, 82%, d.r. >19:1).

^{1}H NMR (400 MHz, $CDCl_3$) δ: 7.34 - 7.36 (m, 1H), 7.27 - 7.30 (m, 1H), 7.20 - 7.26 (m, 5H), 6.29 - 6.31 (m, 1H), 4.41 (d, *J* = 2.6 Hz, 1H), 3.48 (d, *J* = 14.0 Hz, 1H), 3.31 (d, *J* = 14.1 Hz, 1H), 2.45 (d, *J* = 2.6 Hz, 1H), 2.09 (s, 3H), 1.34 (s, 9H). **^{13}C NMR (101 MHz, $CDCl_3$)** δ: 204.12, 168.73, 142.28, 142.17, 136.03, 130.45, 128.37, 127.18, 121.39, 111.95, 82.94, 82.57, 73.65, 68.91, 40.17, 33.18, 30.26, 27.83. **HPLC analysis** (AD-H, Hexanes: ^{i}PrOH = 99:1, 1 mL/min) indicated 93% *ee*: t_R (major) = 14.1 min, t_R (minor) = 18.1 min. **IR (thin film, cm^{-1})**: 3289, 2981, 2931, 1706, 1455, 1368, 1275, 1260, 1147, and 749. **HRMS (DART-TOF)** calculated for $C_{22}H_{24}$ NaO_4^+ $[M + Na]^+$ m/z 375.1572, found 375.1571. $[\alpha]_D^{21}$= -20.4 (c = 0.25, $CHCl_3$).

***tert*-butyl (2*S*,3*R*)-2-acetyl-2-benzyl-3-(thiophen-3-yl)pent-4-ynoate**

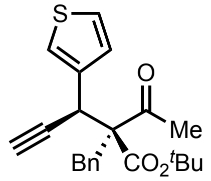

14k

Product **14k** was prepared following **General Procedure** from *tert*-butyl 2-benzyl-3-oxobutanoate (50.7 mg, 0.20 mmol, 1.0 equiv), *tert*-butyl (1-(thiophen-3-yl)prop-2-yn-1-yl) carbonate (71.4 mg, 0.30 mmol, 1.5 equiv), $Cu(CH_3CN)_4BF_4$ (3.2 mg, 0.01 mmol, 5.0 mol%), **L5** (5.4 mg, 0.02 mmol, 10 mol%), $Mg(O^tBu)_2$ (6.8 mg, 0.04 mmol, 0.2 equiv.) and isolated by flash chromatography (silica gel, PE: EtOAc = 30:1) as a colorless oil (55 mg, 75%, d.r. >19:1).

^{1}H NMR (400 MHz, $CDCl_3$) δ: 7.16 - 7.25 (m, 7H), 7.00 - 7.03 (m, 1H), 4.65 (d, *J* = 2.7 Hz, 1H), 3.45 (d, *J* = 13.9 Hz, 1H), 3.33 (d, *J* = 13.9 Hz, 1H), 2.49 (d, *J* = 2.7 Hz, 1H), 2.06 (s, 3 H), 1.30 (s, 9 H). **^{13}C NMR (101 MHz, $CDCl_3$)** δ: 204.40, 168.79, 136.88, 136.25, 130.48, 129.17, 128.32, 127.10, 124.72, 124.69, 83.19, 82.85, 74.14, 69.35, 40.61, 37.67, 30.75 and 27.78. **HPLC analysis** (AD-H, Hexanes: ^{i}PrOH = 99:1, 1 mL/min) indicated 94% *ee*: t_R (major) = 14.1 min, t_R (minor) = 29.2 min. **IR (thin film, cm^{-1})**: 3284, 3005, 2987, 1706, 1455, 1368, 1275, 1260, 1149 and 749. **HRMS (DART-TOF)** calculated for $C_{22}H_{24}NaO_3S^+$ [M + Na]$^+$ m/z 391.1344, found 391.1348. **$[\alpha]_D^{21}$**= -23.1 (c = 0.28, $CHCl_3$).

tert-butyl (2*S*,3*S*)-2-acetyl-2-benzyl-3-(2,3-dihydrobenzo[*b*][1,4]dioxin-5-yl)pent-4-ynoate

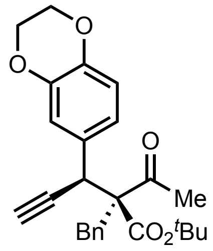

14l

Product **14l** was prepared following **General Procedure** from *tert*-butyl 2-benzyl-3-oxobutanoate (49.3 mg, 0.20 mmol, 1.0 equiv), *tert*-butyl (1-(2,3-dihydrobenzo[*b*][1,4]dioxin-6-yl)prop-2-yn-1-yl) carbonate (87.8 mg, 0.30 mmol, 1.5 equiv), $Cu(CH_3CN)_4BF_4$ (3.2 mg, 0.01 mmol, 5.0 mol%), **L5** (5.4 mg, 0.02 mmol, 10 mol%), $Mg(O^tBu)_2$ (6.8 mg, 0.04 mmol, 0.2 equiv.) and isolated by flash chromatography (silica gel, PE: EtOAc = 30:1) as a white solid (75 mg, 90%, d.r. >19:1).

^{1}H NMR (400 MHz, $CDCl_3$) δ: 7.16 - 7.25 (m, 5H), 6.87 - 6.90 (m, 1H), 6.73 - 6.82 (m, 2H), 4.46 (d, *J* = 2.7 Hz, 1H), 4.22 (s, 4H), 3.40 (d, *J* = 13.8 Hz, 1H), 3.18 (d, *J* = 13.7 Hz, 1H), 2.46 (d, *J* = 2.7 Hz, 1H), 2.11 (s, 3H), 1.32 (s, 9H). **^{13}C NMR (101 MHz, $CDCl_3$)** δ: 204.34, 169.01, 143.16, 142.90, 136.63, 130.58, 129.44, 128.18, 126.90, 123.04, 118.94, 116.71, 83.73, 82.93, 74.15, 69.62, 64.44, 64.36, 42.34, 40.68, 31.39, 27.84. **HPLC analysis** (IA, Hexanes: ^{i}PrOH = 95:5, 1 mL/min) indicated 91% *ee*: t_R (major) = 18.8 min, t_R (minor) = 13.5 min. **IR (thin film, cm^{-1}):** 3283, 2987, 2928, 1701, 1506, 1456, 1368, 1275, 1260, 1147, 1068 and 750. **HRMS (DART-TOF)** calculated for $C_{26}H_{28}NaO_5^+$ [M + Na]$^+$ m/z 443.1834, found 443.1833. **$[\alpha]_D^{21}$**= -19.3 (c = 0.16, $CHCl_3$). **M.p.**: 66.7-68.3 °C.

tert-butyl (2_S_,3_S_)-2-acetyl-2-benzyl-3-(1-tosyl-1_H_-indol-5-yl)pent-4-ynoate

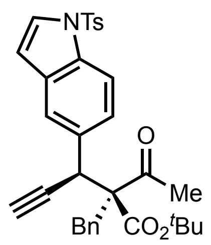

14m

Product **14m** was prepared following **General Procedure** from _tert_-butyl 2-benzyl-3-oxobutanoate (49.4 mg, 0.20 mmol, 1.0 equiv), _tert_-butyl (1-(1-tosyl-1H-indol-7-yl)prop-2-yn-1-yl) carbonate (126.1mg, 0.30 mmol, 1.5 equiv), $Cu(CH_3CN)_4BF_4$ (3.2 mg, 0.01 mmol, 5.0 mol%), **L5** (5.4 mg, 0.02 mmol, 10 mol%), $Mg(O^tBu)_2$ (6.8 mg, 0.04 mmol, 0.2 equiv.) and isolated by flash chromatography (silica gel, PE: EtOAc = 10:1) as a white solid (94 mg, 85%, d.r. >19:1).

^{1}H NMR (400 MHz, $CDCl_3$) δ: 7.81 - 7.88 (m, 1H), 7.70 - 7.76 (m, 2H), 7.49 - 7.55 (m, 2H), 7.27 - 7.31 (m, 1H), 7.14 - 7.26 (m, 7H), 6.56 - 6.61 (m, 1H), 4.65 (d, _J_ = 2.7 Hz, 1H), 3.40 (d, _J_ = 13.8 Hz, 1H), 3.27 (d, _J_ = 13.8 Hz, 1H), 2.49 (d, _J_ = 2.7 Hz, 1H), 2.33 (s, 3H), 2.03 (s, 3H), 1.20 (s, 9H). **^{13}C NMR (101 MHz, $CDCl_3$)** δ: 204.37, 168.91, 145.00, 136.43, 135.36, 134.20, 131.68, 130.54, 130.50, 129.95, 128.24, 127.00, 126.90, 126.81, 126.69, 123.01, 112.89, 109.12, 83.81, 82.92, 74.38, 69.74, 42.47, 40.83, 31.30, 27.70, 21.60. **HPLC analysis** (AD-H, Hexanes: ^{i}PrOH = 90:10, 1 mL/min) indicated 92% _ee_: t_R (major) = 32.2 min, t_R (minor) = 43.7 min. **IR (thin film, cm^{-1})**: 3289, 3005, 2987, 1702, 1457, 1369, 1275, 1260, 1171, 1143 and 750. **HRMS (DART-TOF)** calculated for $C_{33}H_{33}NNaO_5S^+$ [M + Na]$^+$ m/z 578.1977, found 578.1970. **$[\alpha]_D^{21}$**= -20.3 (c = 0.31, $CHCl_3$). **M.p.**: 105.2-107.5 °C.

tert-butyl (2_S_,3_R_)-2-acetyl-2-benzyl-3-(4-methylthiazol-5-yl)pent-4-ynoate

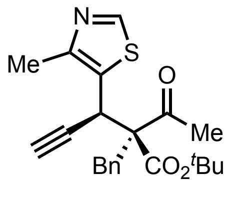

14n

Product **14n** was prepared following **General Procedure** from _tert_-butyl 2-benzyl-3-oxobutanoate (48.9 mg, 0.20 mmol, 1.0 equiv), _tert_-butyl (1-(4-methylthiazol-5-yl)prop-2-yn-1-yl) carbonate (75.7 mg, 0.30 mmol, 1.5 equiv), $Cu(CH_3CN)_4BF_4$ (3.2 mg, 0.01 mmol, 5.0 mol%), **L5** (5.4 mg, 0.02 mmol, 10 mol%), $Mg(O^tBu)_2$ (6.8 mg, 0.04 mmol, 0.2 equiv.) and isolated by flash chromatography (silica gel, PE: EtOAc = 10:1) as a pale yellow oil (40 mg, 52%, d.r. = 10:1).

^{1}H NMR (400 MHz, $CDCl_3$) δ: 8.62 - 8.70 (m, 1H), 7.10 - 7.26 (m, 5H), 4.69 (d, _J_ = 2.7 Hz, 1H), 3.47 (d, _J_ = 13.8 Hz, 1H), 3.22 (d, _J_ = 13.8 Hz, 1H), 2.45 (d, _J_ = 2.7 Hz, 1H), 2.37 (s, 3H), 2.12 (s, 3H), 1.40 (s, 9H). **^{13}C NMR (101 MHz, $CDCl_3$)** δ: 203.93, 169.06, 151.85, 135.98, 130.58, 130.52, 128.41, 128.31, 127.20, 83.75, 82.49, 74.10, 68.82,

39.61, 35.40, 30.91, 27.86, 15.88. **HPLC analysis** (IA, Hexanes: ^{i}PrOH = 95:5, 1 mL/min) indicated 80% *ee*: t_R (major) =14.2 min, t_R (minor) = 18.2 min. **IR (thin film, cm^{-1}):** 3278, 3003, 2982, 1706, 1454, 1368, 1275, 1260, 1150 and 749. **HRMS (DART-TOF)** calculated for $C_{22}H_{25}$ $NNaO_3S^+$ [M + H]$^+$m/z 384.1633, found 384.1632. **[α]$_D$^{21}**= -12.6 (c = 0.27, $CHCl_3$).

***tert*-butyl (2*S*,3*S*)-2-acetyl-2-benzyl-3-(pyridin-3-yl)pent-4-ynoate**

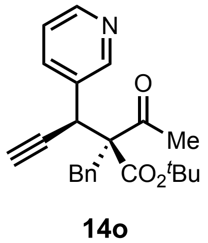

14o

Product **14o** was prepared following **General Procedure** from *tert*-butyl 2-benzyl-3-oxobutanoate (49.9 mg, 0.20 mmol, 1.0 equiv), *tert*-butyl (1-(pyridin-3-yl)prop-2-yn-1-yl) carbonate (69.1mg, 0.30 mmol, 1.5 equiv), $CuBF_4(CH_3CN)_4$ (12.6 mg, 0.04 mmol, 0.2 equiv), **L5** (43.2 mg, 0.08 mmol, 0.4 equiv), Mg(O^{t}Bu)$_2$ (27.6 mg, 0.16 mmol, 0.8 equiv) and isolated by flash chromatography (silica gel, PE: EtOAc = 5:1) as a colorless oil (50 mg, 74%, d.r. = 8:1).

^{1}H NMR (400 MHz, $CDCl_3$) δ: 8.58 - 8.70 (m, 1H), 8.41 - 8.53 (m, 1H), 7.63 - 7.73 (m, 1H), 7.15 - 7.29 (m, 6H), 4.56 (d, *J* = 2.7 Hz, 1H), 3.48 (d, *J* = 13.9 Hz, 1H), 3.34 (d, *J* = 13.9 Hz, 1H), 2.54 (d, *J* = 2.7 Hz, 1H), 2.03 (s, 3H), 1.35 (s, 9H). **^{13}C NMR (101 MHz, $CDCl_3$)** δ: 203.76, 168.55, 151.56, 148.63, 137.91, 135.72, 132.77, 130.42, 128.44, 127.32, 122.74, 83.57, 82.23, 75.14, 69.48, 40.57, 39.58, 30.64, 27.85. **HPLC analysis** (AD-H, Hexanes: ^{i}PrOH = 90:10, 1 mL/min) indicated 89% *ee*: t_R (major) = 11.9 min, t_R (minor) = 12.8 min. **IR (thin film, cm^{-1})**: 3280, 2977, 2928, 1704, 1368, 1277, 1146, 842, 751 and 755. **HRMS (DART-TOF)** calculated for $C_{23}H_{26}NO^+$ [M + Na]$^+$ m/z 364.1913, found 364.1908. **[α]$_D$^{17}**= -17.4 (c = 0.21, $CHCl_3$).

***tert*-butyl (2*S*,3*R*)-2-acetyl-2-benzyl-3-cyclohexylpent-4-ynoate**

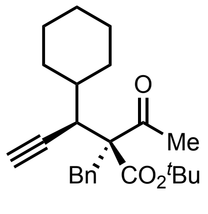

14p

Product **14p** was prepared following **General Procedure** from *tert*-butyl 2-benzyl-3-oxobutanoate (49.7 mg, 0.20 mmol, 1.0 equiv), 1-cyclohexylprop-2-yn-1-yl 2,3,4,5,6-pentafluorobenzoate (71.7 mg, 0.30 mmol, 1.5 equiv), $CuBF_4(CH_3CN)_4$ (12.6 mg, 0.04 mmol, 0.2 equiv), **L5** (43.2 mg, 0.08 mmol, 0.4 equiv), Mg(O^{t}Bu)$_2$ (27.6 mg, 0.16 mmol, 0.8 equiv) and isolated by flash chromatography (silica gel, PE: EtOAc = 30:1) as a white solid (65 mg, 88%, d.r. = 7:1).

^{1}H NMR (400 MHz, $CDCl_3$) δ: 7.15 - 7.26 (m, 5H), 3.38 (d, *J* = 13.9 Hz, 1H), 3.22 (d, *J* = 13.9 Hz, 1H), 3.00 (br t, *J* = 2.8 Hz, 1H), 2.29 (d, *J* = 2.7 Hz, 1H), 2.17 (s, 3H), 1.50-1.76 (m, 6H), 1.44 (s, 9H), 1.10 - 1.32 (m, 5H). **^{13}C NMR (101 MHz, $CDCl_3$)** δ: 205.27, 169.74, 136.49, 130.55, 128.24, 126.99, 83.26, 82.88, 74.09, 67.56, 42.98, 40.36, 37.52, 33.99, 30.83, 30.04, 28.00, 26.99, 26.45, 26.14. **HPLC analysis** (OJ-H, Hexanes: ^{i}PrOH = 99:1, 1 mL/min) indicated >99% *ee*: t_R (major) = 11.5 min, t_R (minor) = 14.0 min. **IR (thin film, cm^{-1})**: 2933, 1707, 1274, 1262, 1151, 763 and 751. **HRMS (DART-TOF)** calculated for $C_{24}H_{32}NaO_3^+$ [M + Na]$^+$ m/z 391.2249, found 391.2239. **$[\alpha]_D^{21}$**= -16.2 (c = 0.20, $CHCl_3$). **M.p.**: 55.4-57.8 °C.

***tert*-butyl (2*S*,3*R*)-2-acetyl-2-benzyl-3-phenethylpent-4-ynoate**

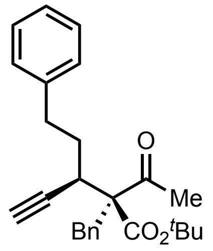

14q

Product **14q** was prepared following **General Procedure** from *tert*-butyl 2-benzyl-3-oxobutanoate (49.9 mg, 0.20 mmol, 1.0 equiv), 5-phenylpent-1-yn-3-yl 2,3,4,5,6-pentafluorobenzoate (78.0 mg, 0.30 mmol, 1.5 equiv), $CuBF_4(CH_3CN)_4$ (12.8 mg, 0.04 mmol, 0.2 equiv), **L5** (43.7 mg, 0.08 mmol, 0.4 equiv), $Mg(O^tBu)_2$ (27.3 mg, 0.16 mmol, 0.8 equiv) and isolated by flash chromatography (silica gel, PE: EtOAc = 30:1) as a pale yellow oil (64 mg, 82%, d.r. = 5:1).

^{1}H NMR (400 MHz, $CDCl_3$) δ: 7.22 – 7.28 (m, 2H), 7.14 – 7.22 (m, 6H), 7.07 – 7.13 (m, 2H), 3.39 (d, *J* = 14.2 Hz, 1H), 3.17 (d, *J* = 14.2 Hz, 1H), 2.88 - 2.99 (m, 2H), 2.58 - 2.68 (m, 1H), 2.34 (d, *J* = 2.3 Hz, 1H), 2.16 (s, 3H), 1.89 - 1.80 (m, 1H), 1.56 - 1.65 (m, 1H), 1.44 (s, 9H). **^{13}C NMR (101 MHz, $CDCl_3$)** δ: 204.01, 169.35, 141.53, 135.99, 130.39, 128.58, 128.45, 128.27, 127.07, 126.01, 84.34, 82.87, 73.38, 67.64, 39.25, 35.41, 34.43, 32.83, 29.72, 28.06. **HPLC analysis** (OJ-H, Hexanes: ^{i}PrOH = 99:1, 1 mL/min) indicated 81% *ee*: t_R (major) = 10.9 min, t_R (minor) = 8.3 min. **IR (thin film, cm^{-1})**: 3284, 2977, 2929, 1708, 1495, 1454, 1368, 1277, 1083, 845, 755 and 751. **HRMS (DART-TOF)** calculated for $C_{26}H_{30}NaO_3^+$ [M + Na]$^+$ m/z 413.2093, found 413.2086. **$[\alpha]_D^{17}$**= -15.7 (c = 0.18, $CHCl_3$).

***tert*-butyl (2*S*,3*S*)-2-acetyl-2-benzyl-3-phenylpent-4-ynoate**

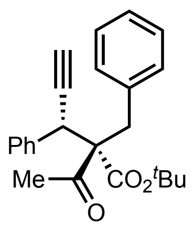

15a

Product **15a** was prepared following **General Procedure** from *tert*-butyl 2-benzyl-3-oxobutanoate (49.1 mg, 0.20 mmol, 1.0 equiv), *tert*-butyl (1-phenylprop-2-yn-1-yl) carbonate (69.0mg, 0.30 mmol, 1.5 equiv), $Cu(CH_3CN)_4BF_4$ (3.2 mg, 0.01 mmol, 5.0 mol%), **L5** (5.4 mg, 0.02 mmol, 10 mol%), $Mg(O^tBu)_2$ (6.8 mg, 0.04 mmol, 0.2 equiv.) and isolated by flash chromatography (silica gel, PE: EtOAc = 30:1) as a colorless oil (63 mg, 87%, d.r. >19:1).

^{1}H NMR (400 MHz, $CDCl_3$) δ: 7.18 - 7.37 (m, 10H), 4.57 (d, *J* = 2.7 Hz, 1H), 3.41 (d, *J* = 13.7 Hz, 1H), 3.23 (d, *J* = 13.7 Hz, 1H), 2.47 (d, *J* = 2.7 Hz, 1H), 2.10 (s, 3H), 1.30 (s, 9H). **^{13}C NMR (101 MHz, $CDCl_3$)** δ: 204.35, 168.96, 136.54, 136.44, 130.57, 130.04, 128.22, 128.04, 127.73, 126.96, 83.63, 82.97, 74.24, 69.58, 42.91, 40.76, 31.39, 27.80. **HPLC** analysis (AD-H, Hexanes: ^{i}PrOH = 99:1, 1 mL/min) indicated 95% *ee*: t_R (major) = 15.5 min, t_R (minor) = 21.1 min. **IR (thin film, cm^{-1})**: 3005, 2992, 1494, 1463, 1275, 1260 and 749. **HRMS (DART-TOF)** calculated for $C_{24}H_{26}NaO_3^+$ $[M + Na]^+$ m/z 385.1780, found 385.1780. **$[\alpha]_D^{21}$**= -35.5 (c = 0.14, $CHCl_3$).

***tert*-butyl (2*S*,3*S*)-2-acetyl-2-(2-methylbenzyl)-3-phenylpent-4-ynoate**

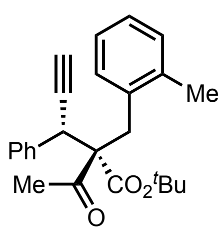

15b

Product **15b** was prepared following **General Procedure** from **11b** (52.8 mg, 0.20 mmol, 1.0 equiv), *tert*-butyl (1-phenylprop-2-yn-1-yl) carbonate (68.7mg, 0.30 mmol, 1.5 equiv), $Cu(CH_3CN)_4BF_4$ (3.2 mg, 0.01 mmol, 5.0 mol%), **L5** (5.4 mg, 0.02 mmol, 10 mol%), $Mg(O^tBu)_2$ (6.8 mg, 0.04 mmol, 0.2 equiv.) and isolated by flash chromatography (silica gel, PE: EtOAc = 30:1) as a white solid (60 mg, 80%, d.r. >19:1).

^{1}H NMR (400 MHz, $CDCl_3$) δ: 7.31 - 7.38 (m, 2H), 7.26 - 7.30 (m, 3H), 7.20 - 7.24 (m, 1H), 7.02 - 7.09 (m, 3H), 4.64 (d, *J* = 2.7 Hz, 1H). 3.42 (d, *J* = 14.4 Hz, 1H), 3.36 (d, *J* = 14.5 Hz, 1H), 2.45 (d, *J* = 2.7 Hz, 1H), 2.22 (s, 3H), 2.02 (s, 3H), 1.23 (s, 9H). **^{13}C NMR (101 MHz, $CDCl_3$)** δ: 204.54, 169.25, 137.35, 136.53, 135.80, 130.55, 130.19, 129.97, 128.16, 127.84, 126.75, 125.86, 83.68, 82.92, 73.92, 68.86, 43.60, 35.46, 31.05, 27.61 and 20.19. **HPLC** analysis (OD-H, Hexanes: ^{i}PrOH = 99:1, 1 mL/min) indicated 93% *ee*: t_R (major) = 6.0 min, t_R (minor) = 6.5 min. **IR (thin film, cm^{-1}):** 3302,

2979, 2931, 1700, 1454, 1368, 1260, 1146 and 749. **HRMS (DART-TOF)** calculated for $C_{25}H_{28}NaO_3^+$ [M + Na]$^+$m/z 399.1936, found 399.1937. **$[\alpha]_D^{21}$**= -33.5 (c = 0.35, $CHCl_3$). **M.p.:** 47.8-50.3 °C.

***tert*-butyl (2*S*,3*S*)-2-acetyl-2-(3-chlorobenzyl)-3-phenylpent-4-ynoate**

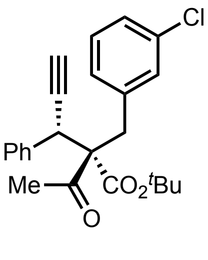

15c

Product **15c** was prepared following **General Procedure** from **11c** (56.6 mg, 0.20 mmol, 1.0 equiv), *tert*-butyl (1-phenylprop-2-yn-1-yl) carbonate (69.7mg, 0.30 mmol, 1.5 equiv), $Cu(CH_3CN)_4BF_4$ (3.2 mg, 0.01 mmol, 5.0 mol%), **L5** (5.4 mg, 0.02 mmol, 10 mol%), $Mg(O^tBu)_2$ (6.8 mg, 0.04 mmol, 0.2 equiv.) and isolated by flash chromatography (silica gel, PE: EtOAc = 30:1) as a colorless oil (66 mg, 83%, d.r. =14:1).

^{1}H NMR (400 MHz, $CDCl_3$) δ: 7.26 - 7.34 (m, 5H), 7.12 - 7.21 (m, 3H), 7.02 - 7.07 (m, 1H), 4.52 (d, *J* = 2.7 Hz, 1H), 3.35 (d, *J* = 13.6 Hz, 1H), 3.09 (d, *J* = 13.7 Hz, 1H), 2.47 (d, *J* = 2.7 Hz, 1H), 2.19 (s, 3H), 1.33 (s, 9H). **^{13}C NMR (101 MHz, $CDCl_3$)** δ: 204.07, 168.85, 138.77, 136.01, 133.90, 130.75, 129.94, 129.40, 128.85, 128.20, 127.95, 127.10, 83.43, 83.37, 74.38, 69.47, 43.32, 40.17, 31.50, 27.84. **HPLC analysis** (IA, Hexanes: ^{i}PrOH = 99:1, 1 mL/min) indicated 92% *ee*: t_R (major) =16.7 min, t_R (minor) = 19.5 min. **IR (thin film, cm^{-1})**: 3273, 3005, 2988, 1711, 1577, 1556, 1455, 1368, 1275, 1260, 1152 and 751. **HRMS (DART-TOF)** calculated for $C_{24}H_{25}ClNaO_3^+$ [M + Na]$^+$ m/z 419.1390, found 419.1391. **$[\alpha]_D^{25}$**= -22.8 (c = 0.21, $CHCl_3$).

***tert*-butyl (2*S*,3*S*)-2-acetyl-2-(4-bromobenzyl)-3-phenylpent-4-ynoate**

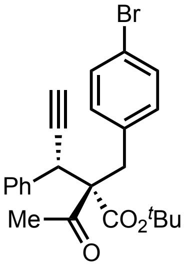

15d

Product **15d** was prepared following **General Procedure** from **11d** (65.4 mg, 0.20 mmol, 1.0 equiv), *tert*-butyl (1-phenylprop-2-yn-1-yl) carbonate (69.7mg, 0.30 mmol, 1.5 equiv), $Cu(CH_3CN)_4BF_4$ (3.2 mg, 0.01 mmol, 5.0 mol%), **L5** (5.4 mg, 0.02 mmol, 10 mol%), $Mg(O^tBu)_2$ (6.8 mg, 0.04 mmol, 0.2 equiv.) and isolated by flash chromatography (silica gel, PE: EtOAc = 30:1) as a white solid (76 mg, 87%, d.r. >19:1).

^{1}H NMR (400 MHz, $CDCl_3$) δ: 7.23 - 7.36 (m, 7H), 7.03 - 7.09 (m, 2H), 4.52 (d, *J* = 2.7 Hz, 1H), 3.34 (d, *J* = 13.7 Hz, 1H), 3.11 (d, *J* = 13.8 Hz, 1H), 2.47 (d, *J* = 2.7 Hz, 1H),

2.16 (s, 3H), 1.31 (s, 9H). **^{13}C NMR (101 MHz, $CDCl_3$)** δ: 204.12, 168.83, 136.11, 135.64, 132.39, 131.25, 129.96, 128.14, 127.87, 121.00, 83.42, 83.25, 74.42, 69.47, 43.12, 40.00, 31.45, 27.83. **HPLC analysis** (AD-H, Hexanes:^{i}PrOH = 99:1, 1 mL/min) indicated 92% *ee*: t_R (major) =26.0 min, t_R (minor) = 23.6 min. **IR (thin film, cm^{-1})**: 3291, 3005, 2988, 1704, 1454, 1360, 1275, 1260, 1149 and 754. **HRMS (DART-TOF)** calculated for $C_{24}H_{25}BrNaO_3^+$ [M + Na]$^+$m/z 463.0885, found 463.0883. **$[\alpha]_D^{21}$**= -35.4 (c = 0.22, $CHCl_3$). **M.p.:** 48.2-49.9 °C.

tert-butyl (_S_)-2-acetyl-4-methyl-2-((_S_)-1-phenylprop-2-yn-1-yl)pent-4-enoate

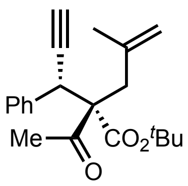

15e

Product **15e** was prepared following **General Procedure** from *tert*-butyl 2-acetyl-4-methylpent-4-enoate (68.5 mg, 0.20 mmol, 1.0 equiv), *tert*-butyl (1-phenylprop-2-yn-1-yl) carbonate (69.7mg, 0.30 mmol, 1.5 equiv), $Cu(CH_3CN)_4BF_4$ (3.2 mg, 0.01 mmol, 5.0 mol%), **L5** (5.4 mg, 0.02 mmol, 10 mol%), $Mg(O^tBu)_2$ (6.8 mg, 0.04 mmol, 0.2 equiv.) and isolated by flash chromatography (silica gel, PE: EtOAc = 30:1) as a pale yellow oil (52 mg, 80%, d.r. >19:1).

^{1}H NMR (400 MHz, $CDCl_3$) δ: 7.28 – 7.14 (m, 5H), 4.74(br s, 1H), 4.68(br s, 1H), 4.46 (d, *J* = 2.7 Hz, 1H), 2.76 (d, *J* = 14.6 Hz, 1H), 2.46 (d, *J* = 14.6 Hz, 1H), 2.34 (d, *J* = 2.8 Hz, 1H), 2.29 (s, 3H), 1.57 (s, 3H), 1.34 (s, 9H). **^{13}C NMR (101 MHz, $CDCl_3$)** δ: 204.03, 169.36, 141.49, 136.32, 129.95, 127.98, 127.73, 115.16, 83.72, 82.82, 73.80, 67.67, 42.59, 42.40, 31.07, 27.90, 24.07. **HPLC analysis** (AD-H, Hexanes: ^{i}PrOH = 99:1, 1 mL/min) indicated 89% *ee*: t_R (major) = 9.5 min, t_R (minor) = 6.8 min. **IR (thin film, cm^{-1})**: 3286, 3007, 2987, 1706, 1455, 1368, 1275, 1260, 1150, and 748. **HRMS (DART-TOF)** calculated for $C_{21}H_{26}NaO_3^+$ [M + Na]$^+$ m/z 349.1780, found 349.1772. **$[\alpha]_D^{21}$**= -16.8 (c = 0.31, $CHCl_3$).

***tert*-butyl (2*S*,3*S*)-2-acetyl-2-((5-chloropyridin-3-yl)methyl)-3-phenylpent-4-ynoate**

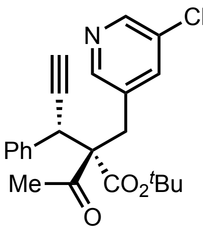

15f

Product **15f** was prepared following **General Procedure** from **11f** (56.8 mg, 0.20 mmol, 1.0 equiv), *tert*-butyl (1-phenylprop-2-yn-1-yl) carbonate (69.7mg, 0.30 mmol, 1.5 equiv), $Cu(CH_3CN)_4BF_4$ (3.1 mg, 0.01 mmol, 5.0 mol%), **L5** (5.4 mg, 0.02 mmol, 10 mol%), $Mg(O^tBu)_2$ (6.8 mg, 0.04 mmol, 0.2 equiv.) and isolated by flash chromatography (silica gel, PE: EtOAc = 10:1) as a yellow oil (50 mg, 63%, d.r. = 11:1).

^{1}H NMR (400 MHz, $CDCl_3$) δ: 8.26 - 8.30 (m, 1H), 7.32 - 7.37 (m, 2H), 7.24 - 7.28 (m, 3H), 7.16 - 7.20 (m, 1H), 7.06 - 7.10 (m, 1H), 4.59 (d, *J* = 2.7 Hz, 1H), 3.57 (d, *J* = 15.6 Hz, 1H), 3.41 (d, *J* = 15.6 Hz, 1H), 2.38 (d, *J* = 2.9 Hz, 1H), 2.37 (s, 3H), 1.29 (s, 9H). **^{13}C NMR (101 MHz, $CDCl_3$)** δ: 204.51, 168.96, 159.91, 149.13, 143.86, 136.17, 130.14, 128.06, 127.86, 124.19, 121.71, 83.38, 82.42, 73.83, 67.01, 42.37, 41.29, 30.78, 27.75. **HPLC** analysis (AD-H, Hexanes: ^{i}PrOH = 99:1, 1 mL/min) indicated 94% *ee*: t_R (major) = 22.3 min, t_R (minor) = 29.1 min. **IR (thin film, cm^{-1})**: 3423, 2979, 2932, 1712, 1576, 1555, 1368, 1356, 1275, 1260, 1139 and 749. **HRMS (DART-TOF)** calculated for $C_{23}H_{24}ClNNaO_3^+$ [M + H]+ m/z 398.1523, found 398.1514. **$[\alpha]_D^{21}$**= -9.4 (c = 0.26, $CHCl_3$).

***tert*-butyl (2*S*,3*S*)-2-acetyl-2-((4-oxo-4*H*-chromen-3-yl)methyl)-3-phenylpent-4-ynoate**

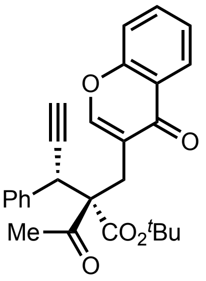

15g

Product **15g** was prepared following **General Procedure** from **11g** (56.8 mg, 0.20 mmol, 1.0 equiv), *tert*-butyl (1-phenylprop-2-yn-1-yl) carbonate (69.7mg, 0.30 mmol, 1.5 equiv), $Cu(CH_3CN)_4BF_4$ (3.2 mg, 0.01 mmol, 5.0 mol%), **L5** (5.4 mg, 0.02 mmol, 10 mol%), $Mg(O^tBu)_2$ (6.8 mg, 0.04 mmol, 0.2 equiv.) and isolated by flash chromatography (silica gel, PE: EtOAc = 10:1) as a white solid (48 mg, 56%, d.r. >19:1).

^{1}H NMR (400 MHz, $CDCl_3$) δ: 8.14 - 8.18 (m, 1H), 8.08 (s, 1H), 7.66 – 7.60 (m, 1H), 7.43 – 7.34 (m, 4H), 7.30 – 7.22 (m, 3H), 4.62 (d, *J* = 2.8 Hz, 1H), 3.30 (d, *J* = 14.3 Hz,

1H), 3.11 (d, *J* = 14.3 Hz, 1H), 2.50 (d, *J* = 2.7 Hz, 1H), 2.40 (s, 3H), 1.33 (s, 9H). **^{13}C NMR (101 MHz, $CDCl_3$)** δ: 203.63, 177.60, 168.93, 156.31, 155.28, 136.29, 133.53, 130.38, 128.04, 127.77, 126.13, 125.14, 123.74, 119.84, 118.16, 84.06, 83.22, 74.60, 67.98, 41.65, 30.33, 28.21, 27.75. **HPLC analysis** (OJ-H, Hexanes: ^{i}PrOH = 99:1, 1 mL/min) indicated >99 % *ee*: t_R (major) = 25.3 min, t_R (minor) = 44.5 min. **HRMS (DART-TOF)** calculated for $C_{27}H_{26}NaO_5^+$ [M + Na]$^+$ m/z 453.1678, found 453.1674. **IR (thin film, cm^{-1})**: 3005, 2988, 2929, 2850, 1706, 1647, 1466, 1275, 1260 and 747. **$[\alpha]_D^{25}$**= 58.09 (c = 0.21, $CHCl_3$). **M.p.:** 80.8-82.4 °C.

ethyl (2*S*,3*S*)-2-acetyl-2-methyl-3-phenylpent-4-ynoate

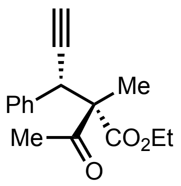

15h

The product **15h** was prepared following **General Procedure** from ethyl 2-methyl-3-oxobutanoate (29.0 mg, 0.20 mmol, 1.0 equiv), *tert*-butyl (1-phenylprop-2-yn-1-yl) carbonate (69.5mg, 0.30 mmol, 1.5 equiv), $Cu(CH_3CN)_4BF_4$ (3.2 mg, 0.01 mmol, 5.0 mol%), **L5** (5.4 mg, 0.02 mmol, 10 mol%), $Mg(O^tBu)_2$ (6.8 mg, 0.04 mmol, 0.2 equiv.) and isolated by flash chromatography (silica gel, PE: EtOAc = 30:1) as a pale yellow oil (44 mg, 85%, d.r. = 16:1).

^{1}H NMR (400 MHz, $CDCl_3$) δ: 7.34 - 7.39 (m, 2H), 7.26 - 7.32 (m, 3H), 4.77 (d, *J* = 2.6 Hz, 1H), 4.05 (t, *J* = 7.2 Hz, 2H), 2.33 (s, 3H), 2.31 (d, *J* = 2.6 Hz, 1H), 1.44 (s, 3H), 1.16 (t, *J* = 7.1 Hz, 3H). **^{13}C NMR (101 MHz, $CDCl_3$)** δ: 203.08, 170.02, 136.32, 129.55, 128.28, 127.89, 82.77, 73.34, 64.94, 61.85, 41.69, 26.78, 15.19, 13.96. **HPLC** analysis (OJ-H, Hexanes: ^{i}PrOH = 99:1, 1 mL/min) indicated 95% *ee*: t_R (major) = 13.8 min, t_R (minor) = 25.7 min. **IR (thin film, cm^{-1}):** 3285, 3004, 2988, 1712, 1453, 1356, 1260, 1107 and 750. **HRMS (DART-TOF)** calculated for $C_{16}H_{18}NaO_3^+$ [M+Na]$^+$ m/z 281.1154, found 281.1154. **$[\alpha]_D^{21}$**= -63.2 (c = 0.25, $CHCl_3$).

***tert*-butyl (2*S*,3*S*)-2-benzyl-3-phenyl-2-propionylpent-4-ynoate**

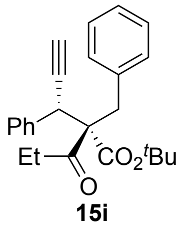

15i

Product **15i** was prepared following **General Procedure** from **11i** (52.4 mg, 0.20 mmol, 1.0 equiv), *tert*-butyl (1-phenylprop-2-yn-1-yl) carbonate (69.5mg, 0.30 mmol, 1.5 equiv), $Cu(CH_3CN)_4BF_4$ (3.2 mg, 0.01 mmol, 5.0 mol%), **L5** (5.5 mg, 0.02 mmol, 10 mol%), $Mg(O^tBu)_2$

(6.7 mg, 0.04 mmol, 0.2 equiv.) and isolated by flash chromatography (silica gel, PE: EtOAc = 30:1) as a colorless oil (59 mg, 78%, d.r. = 9:1).

^{1}H NMR (400 MHz, CDCl$_3$) δ: 7.35 - 7.30 (m, 2H), 7.28 - 7.23 (m, 3H), 7.21 - 7.15 (m, 5H), 4.62 (d, *J* = 2.7 Hz, 1H), 3.37 (d, *J* = 13.8 Hz, 1H), 3.32 (d, *J* = 13.7 Hz, 1H), 2.48 – 2.57 (m, 1H), 2.46 (d, *J* = 2.7 Hz, 1H), 2.09 – 2.20 (m, 1H), 1.32 (s, 9H), 0.90 (t, *J* = 7.1 Hz, 3H). **^{13}C NMR (101 MHz, CDCl$_3$)** δ: 207.58, 169.10, 136.72, 136.63, 130.58, 130.11, 128.18, 127.98, 127.65, 126.92, 83.51, 82.88, 74.18, 69.51, 43.21, 41.12, 36.67, 27.86, 8.34. **HPLC analysis** (AD-H, Hexanes: ^{i}PrOH = 99:1, 1 mL/min) indicated 83 % *ee*: t_R (major) = 9.9 min, t_R (minor) = 7.8 min. **IR (thin film, cm^{-1})**: 3284, 2977, 2935, 1703, 1454, 1368, 1154, 751 and 699. **HRMS (DART-TOF)** calculated for $C_{25}H_{28}NaO_3^+$ [M + Na]$^+$ m/z 399.1936, found 399.1937. **[α]$_D^{17}$**= -35.7 (c = 0.19, CHCl$_3$).

methyl (2*S*,3*S*)-2-benzyl-2-isobutyryl-3-phenylpent-4-ynoate

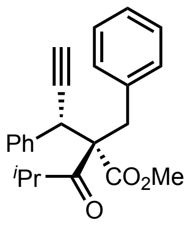

15j

Product **15j** was prepared following **General Procedure** from **11j** (46.8 mg, 0.20 mmol, 1.0 equiv), *tert*-butyl (1-phenylprop-2-yn-1-yl) carbonate (69.9 mg, 0.30 mmol, 1.5 equiv), Cu(CH$_3$CN)$_4$BF$_4$ (3.1 mg, 0.01 mmol, 5.0 mol%), **L5** (5.5 mg, 0.02 mmol, 10 mol%), Mg(O^{t}Bu)$_2$ (6.8 mg, 0.04 mmol, 0.2 equiv.) and isolated isolated by flash chromatography (silica gel, PE: EtOAc = 30:1) as a colorless oil (60 mg, 86%, d.r. = 7:1).

^{1}H NMR (400 MHz, CDCl$_3$) δ: 7.23 - 7.27 (m, 5H), 7.17 - 7.21 (m, 3H), 7.08 - 7.13 (m, 2H), 4.62 (d, *J* = 2.7 Hz, 1H), 3.64 (s, 3H), 3.42 (d, *J* = 13.8 Hz, 1H), 3.34 (d, *J* = 13.8 Hz, 1H), 2.64 - 2.73 (m, 1H), 2.41 (d, *J* = 2.7 Hz, 1H), 0.85 (d, *J* = 6.6 Hz, 3H), 0.65 (d, *J* = 6.6 Hz, 3H). **^{13}C NMR (101 MHz, CDCl$_3$)** δ: 211.03, 170.90, 136.52, 136.46, 130.60, 130.15, 128.22, 127.91, 127.81, 126.99, 82.85, 74.12, 70.25, 51.69, 43.83, 41.08, 40.40, 19.71, 19.42. **HPLC analysis** (AD-H, Hexanes: ^{i}PrOH = 99:1, 1 mL/min) indicated 69% *ee*: t_R (major) = 9.9 min, t_R (minor) = 7.8 min. **IR (thin film, cm^{-1})**: 3282, 3012, 2932, 1739, 1710, 1277, 1265, 763 and 751. **HRMS (DART-TOF)** calculated for $C_{23}H_{24}NaO_3^+$ [M + Na]$^+$ m/z 371.1623, found 371.1623. **[α]$_D^{17}$**= -21.7 (c = 0.14, CHCl$_3$).

tert-butyl (2*S*,3*S*)-2-acetyl-2-fluoro-3-phenylpent-4-ynoate

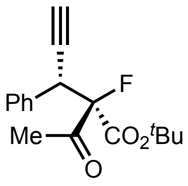

15k

Product **15k** was prepared following **General Procedure** from *tert*-butyl 2-fluoro-3-oxobutanoate (35.2 mg, 0.20 mmol, 1.0 equiv), *tert*-butyl (1-phenylprop-2-yn-1-yl) carbonate (69.7mg, 0.30 mmol, 1.5 equiv), $Cu(CH_3CN)_4BF_4$ (3.2 mg, 0.01 mmol, 5.0 mol%), **L5** (5.5 mg, 0.02 mmol, 10 mol%), $Mg(O^tBu)_2$ (6.8 mg, 0.04 mmol, 0.2 equiv.) and isolated by flash chromatography (silica gel, PE: EtOAc = 30:1) as a pale yellow oil (37 mg, 64%, d.r. = 6:1).

^{1}H NMR (400 MHz, $CDCl_3$) δ: 7.48 – 7.41 (m, 2H), 7.35 - 7.28 (m, 3H), 4.70 (dd, *J* = 30.0, 2.6 Hz, 1H), 2.41 (d, *J* = 5.3 Hz, 3H), 2.29 (d, *J* = 2.6 Hz, 1H), 1.27 (s, 9H). **^{13}C NMR (101 MHz, $CDCl_3$)** δ: 200.93 (d, *J* = 29.6 Hz), 162.56 (d, *J* = 25.2 Hz), 134.21, 129.73 (d, *J* = 2.0 Hz), 128.52, 128.40, 100.94 (d, *J* = 210.7 Hz), 84.74, 80.01(d, *J* = 3.1 Hz), 73.24 (d, *J* = 2.7 Hz), 42.06 (d, *J* = 19.2 Hz), 27.65, 26.63. **^{19}F NMR (376 MHz, $CDCl_3$)** δ: -172.13. **HPLC analysis** (OJ-H, Hexanes: ^{i}PrOH = 99:1, 1 mL/min) indicated 92 % *ee*: t_R (major) = 9.2 min, t_R (minor) = 11.3 min. **IR (thin film, cm^{-1})**: 3291, 2985, 2929, 1749, 1370,1296, 1155, 1081,838 and 751. **HRMS (DART-TOF)** calculated for $C_{17}H_{19}FNaO_3^+$ [M+Na]+ m/z 313.1216, found 313.1208. **$[\alpha]_D^{17}$**= -73.4 (c = 0.14, $CHCl_3$).

diethyl (*S*)-2-acetyl-2-((*S*)-1-phenylprop-2-yn-1-yl)succinate

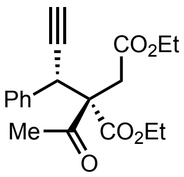

15l

Product **15l** was prepared following **General Procedure** from diethyl 2-acetylsuccinate (43.8 mg, 0.20 mmol, 1.0 equiv), *tert*-butyl (1-phenylprop-2-yn-1-yl) carbonate (69.3mg, 0.30 mmol, 1.5 equiv), $Cu(CH_3CN)_4BF_4$ (3.2 mg, 0.01 mmol, 5.0 mol%), **L5** (5.5 mg, 0.02 mmol, 10 mol%), $Mg(O^tBu)_2$ (6.8 mg, 0.04 mmol, 0.2 equiv.) and isolated by flash chromatography (silica gel, PE: EtOAc = 20:1) as a colorless oil (63 mg, 96%, d.r. >19:1).

^{1}H NMR (400 MHz, $CDCl_3$) δ: 7.26 - 7.36 (m, 5H), 4.50 (d, *J* = 2.7 Hz, 1H), 4.15 – 4.05 (m, 2H), 4.00 (q, *J* = 7.2 Hz, 2H), 3.18 (d, *J* = 17.0 Hz, 1H), 2.93 (d, *J* = 17.0 Hz, 1H), 2.40 (s, 4H), 1.19 (t, *J* = 7.1 Hz, 3H), 1.13 (t, *J* = 7.1 Hz, 3H). **^{13}C NMR (101 MHz, $CDCl_3$)** δ: 202.93, 171.10, 169.58, 135.19, 129.67, 128.22, 128.16, 81.28, 74.71, 65.30, 61.62, 60.73, 42.67, 37.02, 28.99, 14.09, 13.81. **HPLC** analysis (AD-H, Hexanes: ^{i}PrOH = 99:1, 1 mL/min) indicated 94% *ee*: t_R (major) = 12.1 min, t_R (minor) = 10.1 min.

HRMS (DART-TOF) calculated for $C_{19}H_{22}NaO_5^+$ $[M + Na]^+$ m/z 353.1365, found 353.1363. **$[\alpha]_D^{10}$**= -25.4 (c = 0.37, $CHCl_3$).

tert-butyl (2*S*,3*S*)-2-acetyl-3-phenyl-2-propionylpent-4-ynoate

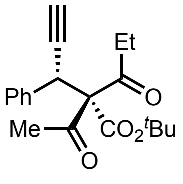

15m

Product **15m** was prepared following **General Procedure** from **11m** (42.8 mg, 0.20 mmol, 1.0 equiv), *tert*-butyl (1-phenylprop-2-yn-1-yl) carbonate (69.3mg, 0.30 mmol, 1.5 equiv), $Cu(CH_3CN)_4BF_4$ (3.1 mg, 0.01 mmol, 5.0 mol%), **L5** (5.5 mg, 0.02 mmol, 10 mol%), $Mg(O^tBu)_2$ (6.8 mg, 0.04 mmol, 0.2 equiv.) and isolated by flash chromatography (silica gel, PE: EtOAc = 20:1) as a colorless oil (41 mg, 62%, d.r. =1:1).

^{1}H NMR (400 MHz, CDCl$_3$) δ: 7.53 – 7.41 (m, 2H), 7.30 – 7.20 (m, 3H), 5.00 (d, *J* = 2.7 Hz, 0.5H), 4.96 (d, *J* = 2.7 Hz, 0.5H), 2.74 – 2.43 (m, 2H), 2.40 (d, *J* = 2.8 Hz, 1H), 2.29 (s, 1.5H), 2.18 (s, 1.5H), 1.46 (d, *J* = 4.4 Hz, 9H), 1.04 (t, *J* = 7.2 Hz, 1.5H), 0.95 (t, *J* = 7.1 Hz, 1.5H). **^{13}C NMR (101 MHz, CDCl$_3$)** δ: 204.43, 202.86, 201.64, 199.84, 165.90, 135.35, 135.27, 130.06, 130.01, 128.15, 128.15, 127.95, 127.94, 84.19, 84.16, 82.99, 82.90, 81.77, 81.60, 73.70, 73.68, 39.31, 39.11, 35.91, 35.07, 30.57, 29.61, 27.83, 27.81, 8.44, 8.09. **P-1 :HPLC** analysis (OD-H, Hexanes: ^{i}PrOH = 99:1, 1 mL/min) indicated 31% *ee*: t_R (major) = 5.9 min, t_R (minor) = 6.2 min. **P-2 : HPLC** analysis (OD-H, Hexanes: ^{i}PrOH = 99:1, 1 mL/min) indicated 25% *ee*: t_R (major) = 5.5 min, t_R (minor) = 7.0 min. **HRMS (DART-TOF)** calculated for $C_{20}H_{24}NaO_4^+$ $[M + Na]^+$ m/z 351.1572, found 351.1563. **$[\alpha]_D^{12}$**= -16.0 (c = 0.13, $CHCl_3$).

(*S*)-2-benzyl-1-cyclopropyl-2-((*S*)-1-phenylprop-2-yn-1-yl)butane-1,3-dione

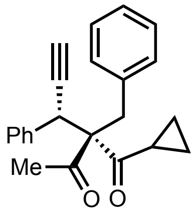

15n

Product **15n** was prepared following **General Procedure** from **11n** (43.8 mg, 0.20 mmol, 1.0 equiv), *tert*-butyl (1-phenylprop-2-yn-1-yl) carbonate (69.3mg, 0.30 mmol, 1.5 equiv), $Cu(CH_3CN)_4BF_4$ (3.1 mg, 0.01 mmol, 5.0 mol%), **L5** (5.5 mg, 0.02 mmol, 10 mol%), $Mg(O^tBu)_2$ (6.8 mg, 0.04 mmol, 0.2 equiv.) and isolated by flash chromatography (silica gel, PE: EtOAc = 20:1) as a colorless oil (53 mg, 82%, d.r. =2.2:1).

^{1}H NMR (400 MHz, Chloroform-*d*) δ 7.44 – 7.33 (m, 2H), 7.32 – 7.07 (m, 8H), 4.67 (d, *J* = 2.8 Hz, 1H), 3.63 (d, *J* = 14.3 Hz, 2H), 3.23 (d, *J* = 14.3 Hz, 1H), 2.47 (d, *J* = 2.8 Hz, 2H), 2.17 (s, 3H), 1.52-1.39 (m, 1H), 1.17 – 1.02 (m, 2H), 0.87-0.69 (m, 2H).

^{13}C NMR (101 MHz, $CDCl_3$) δ 207.21, 204.82, 136.37, 136.08, 130.38, 130.26, 128.34, 128.02, 127.76, 127.05, 83.91, 75.21, 74.59, 41.07, 39.39, 31.17, 22.75, 14.72, 14.52. **Major: HPLC** analysis (IB, Hexanes: ^{i}PrOH = 99:1, 1 mL/min) indicated 89% *ee*: t_R (major) = 11.2 min, t_R (minor) = 9.9 min. **Minor: HPLC** analysis (IB, Hexanes: ^{i}PrOH = 99:1, 1 mL/min) indicated 72% *ee*: t_R (major) = 12.2 min, t_R (minor) = 11.5 min. **HRMS (DART-TOF)** calculated for $C_{23}H_{22}NaO_2^+$ [M + Na]$^+$ m/z 353.1517, found 353.1517. **$[\alpha]_D^{21}$**= -12.5 (c = 0.15, $CHCl_3$).

1-(adamantan-2-yl) 3-methyl (*S*)-2-(4-methylbenzyl)-2-((*S*)-1-phenylprop-2-yn-1-yl)malonate

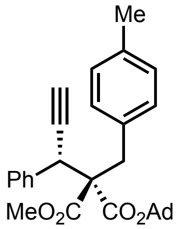

15p

Product **15p** was prepared following **General Procedure** from **11p** (71.8 mg, 0.20 mmol, 1.0 equiv), *tert*-butyl (1-phenylprop-2-yn-1-yl) carbonate (68.8mg, 0.30 mmol, 1.5 equiv), $Cu(CH_3CN)_4BF_4$ (3.1 mg, 0.01 mmol, 5.0 mol%), **L5** (5.5 mg, 0.02 mmol, 10 mol%), $Mg(O^tBu)_2$ (6.8 mg, 0.04 mmol, 0.2 equiv.) and isolated by flash chromatography (silica gel, PE: EtOAc = 30:1) as a colorless oil (86 mg, 92%, d.r. >19:1). **^{1}H NMR (400 MHz, $CDCl_3$)** δ: 7.38 - 7.45 (m, 2H), 7.22 - 7.26 (m, 3H), 7.14 - 7.19 (m, 2H), 7.02 - 7.09 (m, 2H), 4.86 (br t, *J* = 3 Hz, 1H), 4.45 (d, *J* = 2.7 Hz, 1H), 3.66 (d, *J* = 13.9 Hz, 1H), 3.63 (s, 3H), 3.24 (d, *J* = 13.9 Hz, 1H), 2.45 (d, *J* = 2.7 Hz, 1H), 2.30 (s, 3H), 1.59 - 1.88 (m, 12H), 1.32 - 1.44 (m, 2H). **^{13}C NMR (101 MHz, $CDCl_3$)** δ: 169.75, 168.56, 136.76, 136.63, 132.98, 130.13, 130.06, 128.93, 127.94, 127.71, 83.52, 78.74, 73.87, 64.04, 51.84, 42.30, 40.13, 37.35, 36.37, 36.27, 31.71, 31.65, 31.61, 27.16, 26.99, 21.16. **HPLC** analysis (AD-H, Hexanes: ^{i}PrOH = 99:1, 1 mL/min) indicated >99% *ee*: t_R (major) = 28.4 min, t_R (minor) = 25.7 min. **IR (thin film, cm^{-1})**: 3300, 2906, 2855, 1721, 1451, 1434, 1343, 1275, 1261, 1206, 1175 and 749. **HRMS (DART-TOF)** calculated for $C_{31}H_{34}NaO_4^+$ [M + Na]+ m/z 493.2355, found 493.2353. **$[\alpha]_D^{21}$**= -5.2 (c = 0.21, $CHCl_3$).

1-(adamantan-2-yl) 3-methyl (*S*)-2-(3-chlorobenzyl)-2-((*S*)-1-phenylprop-2-yn-1-yl)malonate

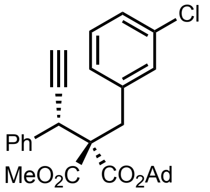

15q

Product **15q** was prepared following **General Procedure** from **11q** (75.6 mg, 0.20 mmol, 1.0 equiv), *tert*-butyl (1-phenylprop-2-yn-1-yl) carbonate (69.5mg, 0.30 mmol, 1.5 equiv), $Cu(CH_3CN)_4BF_4$ (3.2 mg, 0.01 mmol, 5.0 mol%), **L5** (5.4 mg, 0.02 mmol, 10 mol%), $Mg(O^tBu)_2$ (6.8 mg, 0.04 mmol, 0.2 equiv.) and isolated by flash chromatography (silica gel, PE: EtOAc = 30:1) as a colorless oil (85 mg, 87%, d.r. >19:1).

^{1}H NMR (400 MHz, $CDCl_3$) δ: 7.38 - 7.43 (m, 2H), 7.24 - 7.29 (m, 4H), 7.15-7.20 (m, 3H), 4.85 (br t, *J* = 3 Hz, 1H), 4.48 (d, *J* = 2.7 Hz, 1H), 3.64 (s, 3H), 3.58 (d, *J* = 13.8Hz, 1 H), 3.24 (d, *J* = 13.8 Hz, 1H), 2.45 (d, *J* = 2.6 Hz, 1H), 1.61 - 1.83 (m, 12H), 1.35 - 1.44 (m, 2H). **^{13}C NMR (101 MHz, $CDCl_3$)** δ: 169.55, 168.33, 138.46, 136.35, 133.95, 130.51, 129.94, 129.39, 128.60, 128.15, 127.98, 127.27, 83.16, 79.09, 73.97, 64.02, 52.00, 42.91, 39.97, 37.35, 36.39, 36.30, 31.74, 31.69, 31.65, 31.63, 27.16 and 26.99. **HPLC** analysis (AD-H, Hexanes: ^{i}PrOH = 99:1, 1 mL/min) indicated 95% *ee*: t_R (major) = 28.3 min, t_R (minor) = 22.2 min. **IR (thin film, cm^{-1}):** 3302, 2907, 2855, 1721, 1474, 1452, 1261, 1208 and 749. **HRMS (DART-TOF)** calculated for $C_{30}H_{31}ClNaO_4^+$ [M+Na]$^+$ m/z 513.1809, found 513.1808. **$[\alpha]_D^{25}$**= -1.5 (c = 0.21, $CHCl_3$).

1-(adamantan-2-yl) 3-methyl (*S*)-2-allyl-2-((*S*)-1-phenylprop-2-yn-1-yl)malonate

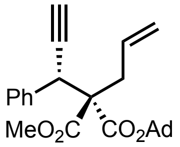

15r

Product **15r** was prepared following **General Procedure** from **11r** (58.1 mg, 0.20 mmol, 1.0 equiv), *tert*-butyl (1-phenylprop-2-yn-1-yl) carbonate (69.1mg, 0.30 mmol, 1.5 equiv), $Cu(CH_3CN)_4BF_4$ (3.1 mg, 0.01 mmol, 5.0 mol%), **L5** (5.4 mg, 0.02 mmol, 10 mol%), $Mg(O^tBu)_2$ (6.9 mg, 0.04 mmol, 0.2 equiv.) and isolated by flash chromatography (silica gel, PE: EtOAc = 30:1) as a pale yellow oil (62 mg, 76%, d.r. >19:1).

^{1}H NMR (400 MHz, $CDCl_3$) δ: 7.38 - 7.43 (m, 2H), 7.25 - 7.30 (m, 3H), 5.77 - 5.88 (m, 1H), 5.04 - 5.16 (m, 2H), 4.93 (br t, *J* = 3 Hz, 1 H), 4.49 (d, *J* = 2.7 Hz, 1H), 3.74 (s, 3H), 2.84 - 2.95 (m, 1H), 2.58 - 2.67 (m, 1H), 2.33 (d, *J* = 2.7 Hz, 1H), 1.70 - 1.95 (m, 12H), 1.45 - 1.52 (m, 2H). **^{13}C NMR (101 MHz, $CDCl_3$)** δ: 169.94, 168.52, 136.38, 133.12, 129.78, 128.19, 127.93, 118.95, 83.13, 78.69, 72.80, 62.68, 52.17, 41.88,

38.27, 37.38, 36.38, 36.33, 31.85, 31.82, 31.80, 27.22, 27.01. **HPLC** analysis (AD-H, Hexanes: ^{i}PrOH = 99:1, 1 mL/min) indicated 93% *ee*: t_R (major) = 40.9 min, t_R (minor) = 16.5 min. **IR (thin film, cm^{-1})**: 3305, 3005, 2989, 2907, 2855, 1724, 1452, 1343, 1275, 1260, 1203 and 749. **HRMS (DART-TOF)** calculated for $C_{26}H_{30}NaO_4^+$ [M + H]$^+$ m/z 407.2222, found 407.2221. **[α]$_D^{20}$**= -7.2 (c = -0.22, $CHCl_3$).

1-(adamantan-2-yl) 3-methyl (*S*)-2-butyl-2-((*S*)-1-phenylprop-2-yn-1-yl)malonate

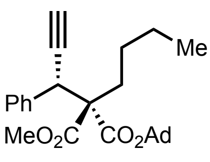

15s

Product **15s** was prepared following **General Procedure** from **11s** (61.9 mg, 0.20 mmol, 1.0 equiv), *tert*-butyl (1-phenylprop-2-yn-1-yl) carbonate (68.5 mg, 0.30 mmol, 1.5 equiv), $Cu(CH_3CN)_4BF_4$ (3.1 mg, 0.01 mmol, 5.0 mol%), **L5** (5.4 mg, 0.02 mmol, 10 mol%), $Mg(O^tBu)_2$ (6.8 mg, 0.04 mmol, 0.2 equiv.) and isolated by flash chromatography (silica gel, PE: EtOAc = 30:1) as a pale yellow oil (61 mg, 72%, d.r. >19:1).

^{1}H NMR (400 MHz, $CDCl_3$) δ: 7.36 - 7.43 (m, 2H), 7.24 - 7.30 (m, 3H), 4.93 (br t, *J* = 3 Hz, 1H), 4.50 (d, *J* = 2.7 Hz, 1H), 3.74 (s, 3H), 2.30 (d, *J* = 2.7 Hz, 1H), 1.96 - 1.69 (m, 14H), 1.25 - 1.50 (m, 6H), 0.87 (t, *J* = 7.2 Hz, 3H). **^{13}C NMR (101 MHz, $CDCl_3$)** δ: 170.49, 169.12, 136.67, 129.66, 128.14, 127.82, 83.32, 78.44, 72.40, 62.64, 52.10, 42.06, 37.39, 36.37, 36.34, 33.75, 31.88, 31.84, 31.83, 31.81, 27.24, 27.04, 27.03, 23.17, 13.94. **HPLC** analysis (AD-H, Hexanes: ^{i}PrOH = 99:1, 1 mL/min) indicated 91% *ee*: t_R (major) = 21.7 min, t_R (minor) = 14.1 min. **IR (thin film, cm^{-1})**: 3308, 2907, 2856, 1724, 1452, 1275, 1260, 1200, 928 and 750. **HRMS (DART-TOF)** calculated for $C_{27}H_{34}NaO_4^+$ [M + H]$^+$ m/z 423.2535, found 423.2535. **[α]$_D^{21}$**= -6.7 (c = 0.37, $CHCl_3$).

1-(adamantan-2-yl) 3-allyl (*S*)-2-benzyl-2-((*S*)-1-phenylprop-2-yn-1-yl)malonate

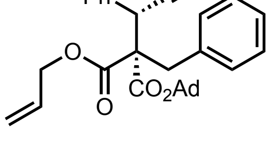

15t

Product **15t** was prepared following **General Procedure** from **11t** (73.9 mg, 0.20 mmol, 1.0 equiv), *tert*-butyl (1-phenylprop-2-yn-1-yl) carbonate (69.9mg, 0.30 mmol, 1.5 equiv), $Cu(CH_3CN)_4BF_4$ (3.2 mg, 0.01 mmol, 5.0 mol%), **L5** (5.4 mg, 0.02 mmol, 10 mol%), $Mg(O^tBu)_2$ (6.8 mg, 0.04 mmol, 0.2 equiv.) and isolated by flash chromatography (silica gel, PE: EtOAc = 30:1) as a pale yellow oil (79 mg, 82%, d.r. >19:1).

^{1}H NMR (400 MHz, CDCl$_3$) δ: 7.45 – 7.39 (m, 2H), 7.31 – 7.20 (m, 8H), 5.82 – 5.70 (m, 1H), 5.15 - 5.28 (m, 2H), 4.84 (br t, *J* = 3 Hz, 1H), 4.57 – 4.42 (m, 3H), 3.65 (d, *J* = 13.8 Hz, 1H), 3.31 (d, *J* = 13.8 Hz, 1H), 2.44 (d, *J* = 2.6 Hz, 1H), 1.81 – 1.60 (m, 12H), 1.30 – 1.40 (m, 2H). **^{13}C NMR (101 MHz, CDCl$_3$)** δ: 169.02, 168.53, 136.62, 136.31, 131.44, 130.42, 130.03, 128.17, 128.02, 127.81, 127.08, 119.01, 83.47, 78.94, 73.89, 66.34, 64.07, 42.78, 40.53, 37.34, 36.37, 36.27, 31.69, 31.66, 31.61, 31.53, 27.10, 26.99. **HPLC** analysis AD-H, Hexanes: ^{i}PrOH = 95:5, 1 mL/min) indicated 91% *ee*: t_R (major) = 10.5 min, t_R (minor) = 9.7 min. **HRMS (DART-TOF)** calculated for $C_{32}H_{34}NaO_4^+$ [M + Na]$^+$ m/z 505.2355, found 505.2350. **IR (thin film, cm^{-1})**: 3007, 2988, 2908, 2854, 1723, 1453, 1275, 1260, 1208, 927 and 751. **[α]$_D^{21}$**= -6.7 (c = 0.33, CHCl$_3$).

1-(adamantan-2-yl) 3-methyl (*S*)-2-(3-(1,3-dioxoisoindolin-2-yl)propyl)-2-((*S*)-1-phenylprop-2-yn-1-yl)malonate

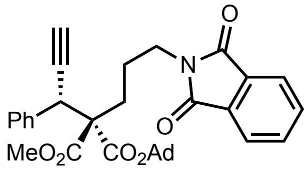

15u

Product **15u** was prepared following **General Procedure** from **11u** (87.1 mg, 0.20 mmol, 1.0 equiv), *tert*-butyl (1-phenylprop-2-yn-1-yl) carbonate (68.3mg, 0.30 mmol, 1.5 equiv), $Cu(CH_3CN)_4BF_4$ (3.1 mg, 0.01 mmol, 5.0 mol%), **L5** (5.5 mg, 0.02 mmol, 10 mol%), Mg(O^{t}Bu)$_2$ (6.7 mg, 0.04 mmol, 0.2 equiv.) and isolated by flash chromatography (silica gel, PE: EtOAc = 5:1) as a white solid (81 mg, 73%, d.r. >19:1).

^{1}H NMR (400 MHz, CDCl$_3$) δ: 7.80 - 7.86 (m, 2H), 7.67 - 7.73 (m, 2H), 7.34 - 7.39 (m, 2H), 7.17 - 7.26 (m, 3H), 4.90 (br t, *J* = 3 Hz, 1H), 4.50 (d, *J* = 2.7 Hz, 1H), 3.72 (s, 3H), 3.69 – 3.62 (m, 2H), 2.27 (d, *J* = 2.6 Hz, 1H), 1.92 – 1.63 (m, 16H), 1.43 - 1.49 (m, 2H). **^{13}C NMR (101 MHz, CDCl$_3$)** δ: 170.02, 168.63, 168.30, 136.22, 133.97, 132.21, 129.62, 128.20, 127.91, 123.24, 82.93, 78.74, 72.77, 62.24, 52.30, 42.14, 38.03, 37.34, 36.34, 36.30, 31.80, 30.87, 27.19, 26.98, 24.34. **IR (thin film, cm^{-1}):** 3283, 2989, 2910, 1713, 1452, 1396, 1275, 1260 and 750. **HPLC** analysis (IA, Hexanes: ^{i}PrOH = 95:5, 1 mL/min) indicated 78% *ee*: t_R (major) = 42.5 min, t_R (minor) = 39.6 min. **HRMS (DART-TOF)** calculated for $C_{33}H_{33}NNaO_6^+$ [M + Na]$^+$ m/z 576.2362, found 576.2353. **[α]$_D^{21}$**= -8.6 (c = 0.21, CHCl$_3$). **M.p.**: 54.2-56.1 °C.

adamantan-2-yl (2*S*,3*S*)-2-benzyl-2-cyano-3-phenylpent-4-ynoate

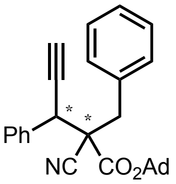

24

Product **24** was prepared following **General Procedure** from **23** (61.3 mg, 0.20 mmol, 1.0 equiv), *tert*-butyl (1-phenylprop-2-yn-1-yl) carbonate (68.7 mg, 0.30 mmol, 1.5 equiv), $Cu(CH_3CN)_4BF_4$ (3.1 mg, 0.01 mmol, 5.0 mol%), **L5** (5.5 mg, 0.02 mmol, 10 mol%), $Mg(O^tBu)_2$ (6.8 mg, 0.04 mmol, 0.2 equiv.) and isolated by flash chromatography (silica gel, PE: EtOAc = 5:1) as a yellow oil (66 mg, 78%, d.r. =1.6:1).

Major: ^{1}H NMR (400 MHz, $CDCl_3$) δ: 7.58 - 7.66 (m, 2H), 7.38 - 7.47 (m, 3H), 7.28 - 7.33 (m, 1H), 7.22 - 7.26 (m, 2H), 7.16 - 7.22 (m, 2H), 4.93 (br t, *J* = 3 Hz, 1H), 4.28 (d, *J* = 2.6 Hz, 1H), 3.12 (d, *J* = 13.2 Hz, 1H), 2.69 (d, *J* = 13.2 Hz, 1H), 2.47 (d, *J* = 2.6 Hz, 1H), 1.54 – 2.03 (m, 12H), 1.37 - 1.47 (m, 2H). **^{13}C NMR (101 MHz, $CDCl_3$)** δ: 167.01, 134.02, 133.81, 130.02, 129.79, 129.04, 128.93, 128.60, 127.96, 117.27, 80.50, 80.39, 74.76, 58.02, 44.75, 41.84, 37.30, 36.31, 36.24, 32.11, 31.78, 31.59, 31.29, 27.06, 26.91. **HPLC** analysis (AD-H, Hexanes: ^{i}PrOH = 98:2, 1 mL/min) indicated 70% *ee*: t_R (major) = 20.6 min, t_R (minor) = 29.9 min. **HRMS (DART-TOF)** calculated for $C_{29}H_{29}NNaO_2^+$ [M + Na]$^+$ m/z 446.2096, found 446.2095. **$[\alpha]_D^{20}$**= -26.1 (c = 0.42, $CHCl_3$).

Minor: ^{1}H NMR (400 MHz, $CDCl_3$) δ: 7.48 - 7.55 (m, 2H), 7.26 - 7.34 (m, 8H), 4.59 (br t, *J* = 3 Hz, 1H), 4.29 (d, *J* = 2.7 Hz, 1H), 3.65 (d, *J* = 13.3 Hz, 1H), 3.37 (d, *J* = 13.3 Hz, 1H), 2.63 (d, *J* = 2.6 Hz, 1H), 1.45 – 1.73 (m, 12H), 1.10 - 1.28 (m, 2H). **^{13}C NMR (101 MHz, $CDCl_3$)** δ: 166.30, 134.88, 134.16, 130.16, 129.21, 128.76, 128.65, 128.61, 127.93, 117.03, 80.25, 80.12, 75.60, 59.00, 45.03, 43.18, 37.18, 36.13, 36.11, 31.67, 31.61, 31.06, 31.03, 26.84, 26.77. **HPLC** analysis (AD-H, Hexanes: ^{i}PrOH = 99:1, 1 mL/min) indicated 65% *ee*: t_R (major) = 15.4 min, t_R (minor) = 30.3 min. **HRMS (DART-TOF)** calculated for $C_{29}H_{29}NNaO_2^+$ [M + Na]$^+$ m/z 446.2096, found 446.2093. **$[\alpha]_D^{20}$**= -31.7 (c = 0.37, $CHCl_3$).

Derivatization Reactions

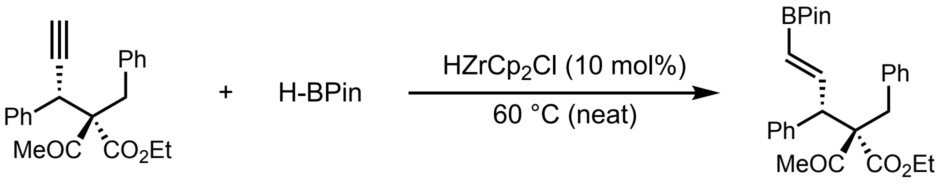

12 **16a**

Alkyne **12** (33.2 mg, 0.10 mmol, 1.0 equiv.), pinacol borane (13.1 mg, 0.11 mmol, 1.1 equiv.), and Schwartz's reagent (2.6 mg, 0.01 mmol, 10 mol%) were added to a screw-capped vial in a N_2-filled glovebox. The reaction was stirred at 60 °C for 24 h. After completed, an aliquot of the residue was dissolved in $CDCl_3$ for ^1H NMR spectroscopy, which indicated the resulting products were formed in ca. 9:1 diastereomeric ratio. The combined mixture was then purified by flash chromatography on silica gel (PE: EtOAc = 30:1), and product **16a** (39 mg, 85% yield) was obtained as a pale yellow oil.

^{1}H NMR (400 MHz, CDCl$_3$) δ: 7.04 - 7.29 (m, 11H), 5.49 (dd, *J* = 17.7, 1.2 Hz, 1H), 4.23 (d, *J* = 8.9 Hz, 1H), 3.97 - 4.09 (m, 2H), 3.15 (d, *J* = 13.6 Hz, 1H), 3.10 (d, *J* = 13.6 Hz, 1H), 1.85 (s, 3H), 1.24 (s, 12H), 1.08 (t, *J* = 7.2 Hz, 3H).

^{13}C NMR (101 MHz, CDCl$_3$) δ: 206.33, 170.97, 151.35, 138.74, 137.07, 130.25, 129.74, 128.36, 128.20, 127.25, 126.83, 83.30, 69.60, 61.15, 57.56, 41.53, 32.35, 24.95, 24.83, 13.74.

HPLC analysis (AD-H, Hexanes: ^{i}PrOH = 99:1, 1 mL/min) indicated 94% ee: t_R (major) = 12.5 min, t_R (minor) = 15.9 min.

HRMS (DART-TOF) calculated for $C_{28}H_{35}BNaO_5^+$ $[M + Na]^+$ m/z 485.2475, found 485.2472.

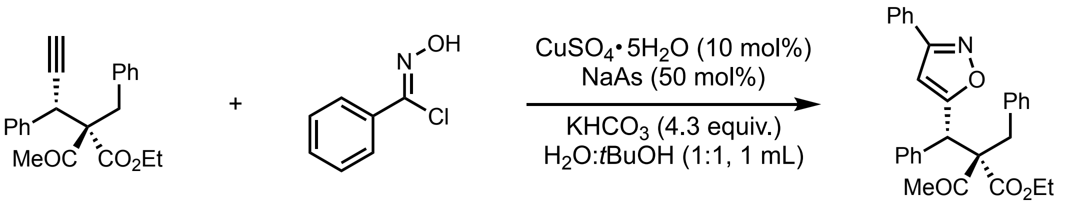

12 **16b**

To a stirred solution of alkyne **12** (33.5 mg, 0.10 mmol, 1.0 equiv.) in H_2O/*t*BuOH (1:1, 1 mL) was added chloro-oxime (16.3 mg, 0.10 mmol, 1.0 equiv.), sodium ascorbate (10.6 mg, 0.05 mmol, 50 mol%), $CuSO_4 \cdot 5H_2O$ (2.5 mg, 0.01 mmol, 10 mol%), and $KHCO_3$ (43.5 mg, 0.43 mmol, 4.3 equiv.). The reaction was stirred at room temperature for 18 h, and then diluted with water. The mixture was extracted with DCM. The organic phase was concentrated under reduced pressure and an aliquot of the residue was dissolved in $CDCl_3$ for ^1H NMR spectroscopy, which indicated the resulting products were formed in ca. 9:1 diastereomeric ratio. The combined mixture was then purified by flash chromatography on silica gel (PE: EtOAc = 30:1), and product **16b** (35 mg, 80% yield) was obtained as a pale yellow oil.

^{1}H NMR (400 MHz, CDCl$_3$) δ: 7.74 - 7.80 (m, 2H), 7.39 - 7.44 (m, 3H), 7.28 -7.36 (m, 5H), 7.14 - 7.20 (m, 3H), 7.00 - 7.07 (m, 2H), 6.50 (s, 1H), 5.20 (s, 1H), 4.08 (q, *J* = 7.2 Hz, 2H), 3.25 (d, *J* = 13.6 Hz, 1H), 3.13 (d, *J* = 13.6 Hz, 1H), 1.85 (s, 3H), 1.07 (t, *J* = 7.2 Hz, 3H).

^{13}C NMR (101 MHz, CDCl$_3$) δ: 204.63, 172.01, 170.64, 162.34, 135.99, 135.67, 130.35, 130.12, 128.96, 128.93, 128.49, 128.22, 128.08, 127.08, 126.85, 102.76, 102.74, 68.79, 61.71, 49.26, 41.03, 30.77, 13.65.

HPLC analysis (AD-H, Hexanes: ^{i}PrOH = 100:1, 1 mL/min) indicated 93% *ee*: t_R (major) = 17.1 min, t_R (minor) = 15.6 min.

HRMS (DART-TOF) calculated for $C_{29}H_{28}NO_4^+$ $[M + H]^+$ m/z 454.2018, found 454.2017.

$[\alpha]_D^{25}$= -35.1 (c = 1.16, $CHCl_3$).

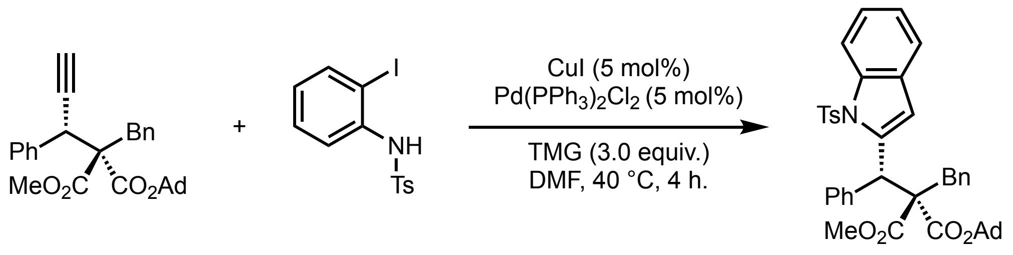

15o **16c**

To an 8mL screw cap vial with a magnetic stir bar was added **15o** (45.7 mg, 0.10 mmol, 1.0 equiv.) and *N*-tosyl-2-iodobenzene (37.3 mg, 0.10 mmol, 1.0 equiv.). The vial was loosely capped and transferred into a N_2-filled glovebox, DMF (1.0 mL) was added, followed by the addition of TMG (38 μL, 0.3 mmol, 3.0 equiv.), $Pd(PPh_3)_2Cl_2$ (3.5 mg, 0.005 mmol, 5 mol%) and CuI (1.0 mg, 0.005 mmol, 5 mol%). The vial was tightly screw-capped, transferred out of the glovebox and stirred at 40 °C for 4 h. At the end of reaction, added water and extracted with EtOAc, dried over anhydrous Na_2SO_4. An aliquot of the residue was dissolved in $CDCl_3$ for ^{1}H NMR spectroscopy, which indicated the resulting products were formed in >19:1 diastereomeric ratio. The combined mixture was then purified by flash chromatography on silica gel (PE: EtOAc = 10:1), and product **16c** (49 mg, 70% yield) was obtained as a yellow oil.

^{1}H NMR (400 MHz, $CDCl_3$) δ: 8.16 - 8.23 (m, 1H), 7.73 - 7.78 (m, 2H), 7.61 -7.68 (m, 1H), 7.36 - 7.40 (m, 2H), 7.27 - 7.32 (m, 3H), 7.19 - 7.25 (m, 7H), 7.10 - 7.15 (m, 2H), 6.90-6.96 (m, 1H), 4.99 (br t, 1H), 4.59 (s, 1H), 3.68 (s, 3H), 3.54 (d, *J* = 13.9 Hz, 1H), 3.31 (d, *J* = 13.9 Hz, 1H), 2.30 (s, 3H), 1.88 – 1.57 (m, 12H), 1.34-1.43 (m, 2H).

^{13}C NMR (101 MHz, $CDCl_3$) δ 169.85, 169.02, 143.62, 139.29, 137.07, 136.33, 135.95, 131.97, 130.26, 129.87, 129.57, 129.38, 128.34, 128.31, 128.08, 127.37, 127.22, 123.44, 118.47, 113.33, 96.28, 80.71, 79.30, 64.22, 52.28, 43.75, 40.36, 37.31, 36.38, 36.26, 31.73, 31.63, 27.14, 26.98, 21.60.

HPLC analysis (AD-H, Hexanes: ^{i}PrOH = 90:10, 1 mL/min) indicated 98% *ee*: t_R (major) = 31.3 min, t_R (minor) = 34.2 min.

IR (thin film, cm^{-1}): 3005, 2988, 2909, 1724, 1490, 1275, 1260, 1167and 749.

HRMS (DART-TOF) calculated for $C_{43}H_{43}NNaO_6S^+$ [M + Na]$^+$ m/z 724.2708, found 724.2709.

$[\alpha]_D^{20}$= -32.4 (c = 0.25, $CHCl_3$).

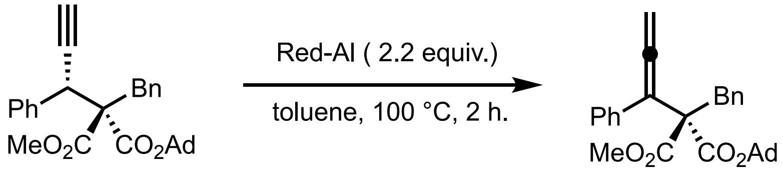

15o **16d**

An 8mL screw cap vial was charged with **15o** (45.7 mg, 0.10 mmol, 1.0 equiv.) and 1 mL toluene, Red-Al (sodium bis(2-methoxyethoxy)aluminiumhydride, 70% in toluene, 63.5 mg, 0.22 mmol, 2.2 equiv.) was added at room temperature. The mixture was stirred at 100 °C for 3 h. After completion, the reaction was quenched by addition of water, and then extracted with EtOAc, dried over anhydrous Na_2SO_4 and concentrated under vacuum. The resulting mixture was purified by flash chromatography on silica gel (PE: EtOAc = 30:1), and product **16d** (37 mg, 82% yield) was obtained as a yellow oil.

^{1}H NMR (400 MHz, $CDCl_3$) δ: 7.32 – 7.36 (m, 2H), 7.24 - 7.27 (m, 2H), 7.16-7.22 (m, 6H), 4.98 (s, 2H), 4.90 (br t, *J* = 3 Hz, 1H), 3.59 (s, 3H), 3.57 (d, *J* = 13.8 Hz, 1H), 3.53 (d, *J* = 13.8 Hz, 1H), 1.66 - 1.80 (m, 12H), 1.38 - 1.44 (m, 2H).

^{13}C NMR (101 MHz, $CDCl_3$) δ: 210.82, 170.11, 168.82, 136.68, 135.72, 130.60, 128.30, 127.97, 127.94, 127.15, 126.92, 105.27, 79.05, 78.79, 62.41, 52.34, 41.80, 37.40, 36.36, 31.82, 31.80, 31.77, 31.70.

HPLC analysis (OD-H, Hexanes:^{i}PrOH = 99:1, 1 mL/min) indicated 91% *ee*: t_R (major) = 9.9 min, t_R (minor) = 8.1 min.

IR (thin film, cm^{-1}): 3007, 2992, 2908, 2858, 1729, 1447, 1275, 1260 and 750.

HRMS (DART-TOF) calculated for $C_{30}H_{32}NaO_4^+$ $[M + Na]^+$ m/z 479.2192, found 479.2198.

$[\alpha]_D^{20}$= -4.8 (c = 0.21, $CHCl_3$).

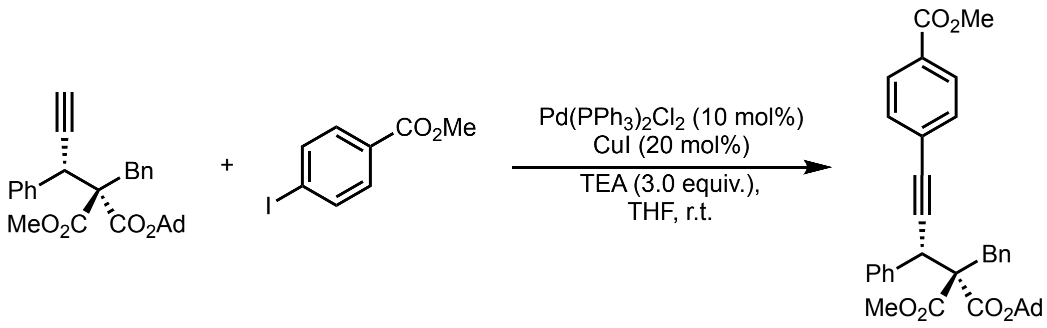

15o **16e**

An 8mL screw cap vial was charged with **15o** (45.2 mg, 0.10 mmol, 1.0 equiv.), methyl 4-iodobenzoate (26.5 mg, 0.10 mmol, 1.0 equiv.) and a magnetic stir bar. The vial was loosely screw-capped and transferred into a N_2-filled glovebox. Then, TEA (42 μL, 0.30 mmol, 3.0 equiv.), $Pd(PPh_3)_2Cl_2$ (7.0 mg, 0.01 mmol, 10 mol%), CuI (3.9 mg, 0.02 mmol, 20 mol%) and 1mL anhydrous THF were added sequentially. The vial was screw-capped, transferred out of the glovebox and stirred at room temperature overnight. After completion, the reaction was quenched with saturated aqueous NH_4Cl, extracted with EtOAc and dried over anhydrous Na_2SO_4. The resulting mixture was concentrated under vacuum and an aliquot of the residue was dissolved in $CDCl_3$ for ^{1}H NMR spectroscopy, which indicated the resulting products were formed in >19:1 diastereomeric ratio. The combined mixture was then purified by flash chromatography on silica gel (PE: EtOAc = 30:1), and product **16e** (54 mg, 92% yield) was obtained as a yellow oil.

^{1}H NMR (400 MHz, $CDCl_3$) δ: 7.94 - 8.00 (m, 2H), 7.44 – 7.53 (m, 4H), 7.35 - 7.40 (m, 1H), 7.20 – 7.32 (m, 8H), 4.83 (br t, *J* = 3 Hz, 1H), 4.71 (s, 1H), 3.91 (s, 3H), 3.69 (d, *J* = 13.8 Hz, 1H), 3.66 (s, 3H), 3.31 (d, *J* = 13.8 Hz, 1H), 1.61 – 1.82 (m, 12H), 1.33 - 1.42 (m, 2H).

^{13}C NMR (101 MHz, $CDCl_3$) δ: 169.76, 168.59, 166.70, 136.79, 136.27, 131.64, 130.33, 130.01, 129.51, 129.45, 128.31, 128.28, 128.16, 127.92, 127.18, 92.72, 85.03, 78.92, 64.57, 52.31, 52.02, 43.48, 40.75, 37.33, 36.36, 36.28, 31.74, 31.67, 31.62, 27.17, 26.99.

HPLC analysis (AD-H, Hexanes: ^{i}PrOH = 90:10, 1 mL/min) indicated 95% *ee*: t_R (major) = 16.8 min, t_R (minor) = 11.4 min.

IR (thin film, cm^{-1}): 3007, 2988, 2908, 2854, 1722, 1435, 1275, 1261, 748, and 403.

HRMS (DART-TOF) calculated for $C_{38}H_{38}NaO_6^+$ [M + Na]+ m/z 613.2565, found 613.2566.

$[\alpha]_D^{25}$= -26.8 (c = 0.30, $CHCl_3$).

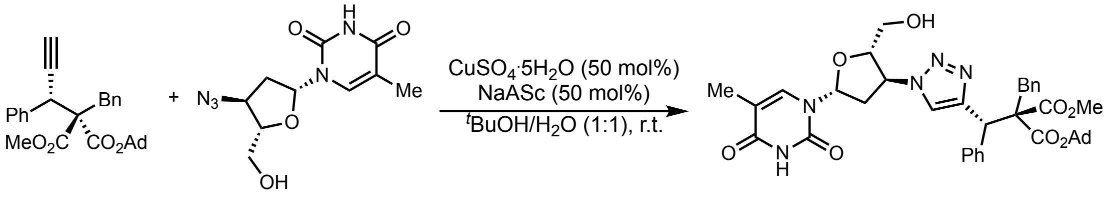

15o **ZDV** **16f**

To an 8mL screw cap vial with a magnetic stir bar was added **15o** (45.7 mg, 0.10 mmol, 1.0 equiv.) and ZDV (31.1 mg, 0.11 mmol, 1.1 equiv.). The vial was loosely capped and transferred into a N_2-filled glovebox, *t*BuOH (0.5 mL) was added. Then, A prepared solution of $CuSO_4$•$5H_2O$ (12.5 mg, 0.05 mmol, 50 mol%), and NaASc (9.6 mg, 0.05 mmol, 50 mol%) in 0.5 mL H_2O was added. The vial was tightly screw-capped, transferred out of the glovebox and stirred at room temperature overnight. The reaction was quenched by addition of water and extracted with EtOAc. The combined organic layers were dried over anhydrous Na_2SO_4 and concentrated under vacuum. An aliquot of the residue was dissolved in $CDCl_3$ for ^{1}H NMR spectroscopy, which indicated the resulting products were formed in >19:1 diastereomeric ratio. The combined mixture was then purified by flash chromatography on silica gel (PE: EtOAc = 3:7), and product **16f** (68 mg, 95% yield) was obtained as a white solid.

^{1}H NMR (400 MHz, $CDCl_3$) δ: 9.63 (s, 1H), 7.85 (d, *J* = 4.9 Hz, 1H), 7.53 (s, 1H), 7.33 – 7.41 (m, 2H), 7.20 – 7.30 (m, 3H), 7.12 – 7.18 (m, 3H), 7.05 – 7.11 (m, 2H), 6.25 (t, *J* = 6.5 Hz, 1H), 7.25 – 7.38 (m, 1H), 5.08 (s, 1H), 4.76 (br t, *J* = 3 Hz, 1H), 4.28 – 4.37 (m, 1H), 3.88 – 3.99 (m, 2H), 3.66– 3.76 (m, 1H), 3.55 (s, 3H), 3.41 (dd, *J* = 13.9, 4.9 Hz, 1H), 3.20 (dd, *J* = 14.0, 3.9 Hz, 1H), 2.77 – 2.93 (m, 2H), 1.87 (s, 3H), 1.47 - 1.81 (m, 12H), 1.28 - 1.40 (m,2H).

^{13}C NMR (101 MHz, $CDCl_3$) δ: 170.58, 170.53, 169.88, 169.83, 164.18, 150.54, 147.71, 147.67, 137.91, 137.73, 137.68, 136.47, 130.39, 130.30, 128.26, 127.97, 127.55, 126.86, 123.29, 111.10, 88.31, 85.42, 78.95, 78.91, 64.06, 63.99, 61.46, 59.00, 51.97, 49.19, 41.30, 37.67, 37.21, 36.24, 36.21, 31.68, 31.59, 31.51, 31.47, 27.02, 26.87, 12.51.

HPLC analysis (AD-H, Hexanes: ^{i}PrOH = 80:20, 1 mL/min) indicated 90% *ee*: t_R (major) = 11.9 min, t_R (minor) = 32.9 min.

IR (thin film, cm^{-1}): 3005, 2988, 1687, 1463, 1275, 1260 and 750.

HRMS (DART-TOF) calculated for $C_{40}H_{45}N_5NaO_8^+$ $[M + Na]^+$ m/z 746.3171, found 746.3166.

M.p.: 104.9-106.7 °C.

$[\alpha]_D^{21}$= -25.0 (c = 0.27, $CHCl_3$).

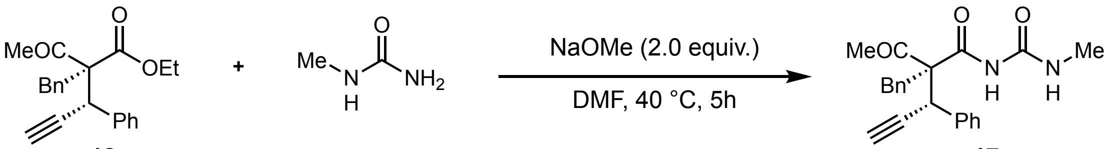

To an 8mL screw cap vial charged with a magnetic stir bar was added **12** (33.3 mg, 0.10 mmol, 1.0 equiv.) and Methylurea (14.6 mg, 0.20 mmol, 2.0 equiv.). The vial was loosely capped and transferred into a N_2-filled glovebox, NaOMe (10 mg, 0.20 mmol, 2.0 equiv.) and DMF (1.0 mL) were added sequentially. Then, the vial was tightly screw-capped, transferred out of the glovebox and stirred at 50 °C for 5 h. After completion, the reaction was quenched by addition of water and extracted with EA. The combined organic layers were dried over anhydrous Na_2SO_4 and concentrated under vacuum. An aliquot of the residue was dissolved in $CDCl_3$ for ^1H NMR spectroscopy, which indicated the resulting products were formed in ca. >19:1 diastereomeric ratio. The resulting mixture was purified by flash chromatography on silica gel (PE: EtOAc = 75:25), and product **17** (24 mg, 65% yield) was obtained as a colorless oil.

^{1}H NMR (400 MHz, Chloroform-d) δ 8.29 – 8.11 (m, 1H), 7.82 (s, 1H), 7.40-7.31 (m, 2H), 7.30-7.23 (m, 1H), 7.19-7.07 (m, 3H), 7.07-7.00 (m, 2H), 6.92-6.79 (m, 2H), 4.79 (d, J = 3.1 Hz, 1H), 4.42 (d, J = 3.1 Hz, 1H), 3.32 (d, J = 14.0 Hz, 1H), 3.23 (d, J = 13.9 Hz, 1H), 2.92 (d, J = 4.8 Hz, 3H), 1.96 (s, 3H).

^{13}C NMR (101 MHz, CDCl3) δ 173.45, 161.67, 154.08, 153.57, 135.72, 131.56, 130.57, 128.97, 127.94, 127.69, 127.24, 126.81, 112.58, 87.61, 64.57, 39.71, 26.62, 13.45.

HPLC analysis (AD-H, Hexanes: ^{i}PrOH = 70:30, 1 mL/min) indicated 91% *ee*: t_R (major) = 5.1 min, t_R (minor) = 4.2 min.

$[\alpha]_D^8$= -24.7 (c = 0.68, $CHCl_3$).

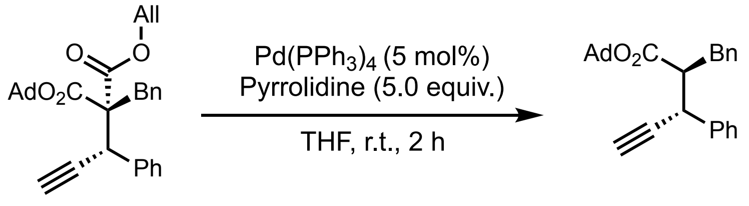

15t **18**

To an 8mL screw cap vial charged with a magnetic stir bar was added **15t** (48.3 mg, 0.10 mmol, 1.0 equiv.). The vial was loosely capped and transferred into a N_2-filled glovebox, pyrrolidine (41 uL, 0.5 mmol, 5.0 equiv.), $Pd(PPh_3)_4$ (5.8 mg, 0.005 mmol, 5 mol%) and THF (1.0 mL) were added sequentially. Then, the vial was tightly screw-capped, transferred out of the glovebox and stirred at room temperature for 2 h. After completion, the reaction was quenched by addition of water and extracted with DCM. The combined organic layers were dried over anhydrous Na_2SO_4 and concentrated under vacuum. An aliquot of the residue was dissolved in $CDCl_3$ for ^{1}H NMR spectroscopy, which indicated the resulting products were formed in ca. 7:1 diastereomeric ratio. The resulting mixture was purified by flash chromatography on silica gel (PE: EtOAc = 30:1), and product **18** (49 mg, 62% yield) was obtained as a white solid.

^{1}H NMR (400 MHz, $CDCl_3$) δ: 7.00 – 7.37 (m, 10H), 4.78 (br t, *J* = 3 Hz, 1H), 3.84 (dd, *J* = 8.5, 2.5 Hz, 1H), 2.95 - 3.04 (m, 1 H), 2.73 - 2.82 (m, 1H), 2.63 - 2.71 (m, 1H), 2.24 (d, *J* = 2.5 Hz, 1H), 1.51 – 1.80 (m, 12H), 1.29 – 1.45 (m, 2H).

^{13}C NMR (101 MHz, $CDCl_3$) δ: 172.49, 138.76, 138.49, 128.90, 128.77, 128.46, 128.31, 127.61, 126.54, 83.48, 77.29, 72.53, 54.80, 40.23, 37.51, 36.79, 36.46, 36.40, 32.05, 31.84, 31.68, 27.28, 27.09, 1.15.

HPLC analysis (OJ-H, Hexanes: ^{i}PrOH = 99:1, 1 mL/min) indicated >99% *ee*: t_R (major) = 14.3 min, t_R (minor) = 21.3 min.

IR (thin film, cm^{-1}): 3005, 2988, 1467, 1275, 1260 and 749.

HRMS (DART-TOF) calculated for $C_{28}H_{30}NaO_2^+$ $[M+Na]^+$ m/z 421.2143, found 421.2140.

$[\alpha]_D^{25}$= -1.7 (c = 0.31, $CHCl_3$).

M.p.: 54.2-55.9 °C.

Determination of Non-Linear Effect

Supplementary Table 6. Positive NLE in the APS reaction of propargyl carbonate **10a** and β-keto ester **11a (Cu:L5 = 1:2)**

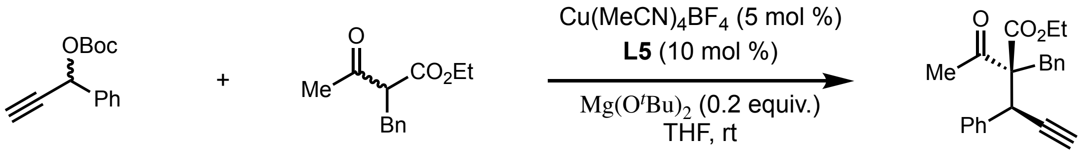

10a (1.0 equiv) **11a** (1.5 equiv) **12**

Entry	**L5** ee[a]%	**Yield%**	***dr***	***ee%***
1	0	90	11 : 1	1
2	20	87	11 : 1	30
3	40	89	11 : 1	60
4	60	92	11 : 1	85
5	80	87	11 : 1	90
6	100	93	11 : 1	96

Reactions in this table were performed on a 0.1 mmol scale. **10a** (0.10 mmol), **11a** (0.15 mmol), $Cu(MeCN)_4BF_4$ (0.005 mmol), Ligand (0.01 mmol), $Mg(O^tBu)_2$ (0.02 mmol), THF (1 mL). Yields and *d.r.* values were determined by ^1H NMR spectroscopy of the crude reaction mixture with 1,1,2,2-tetrachloroethane as an internal standard. The *ee* values were determined by HPLC analysis.

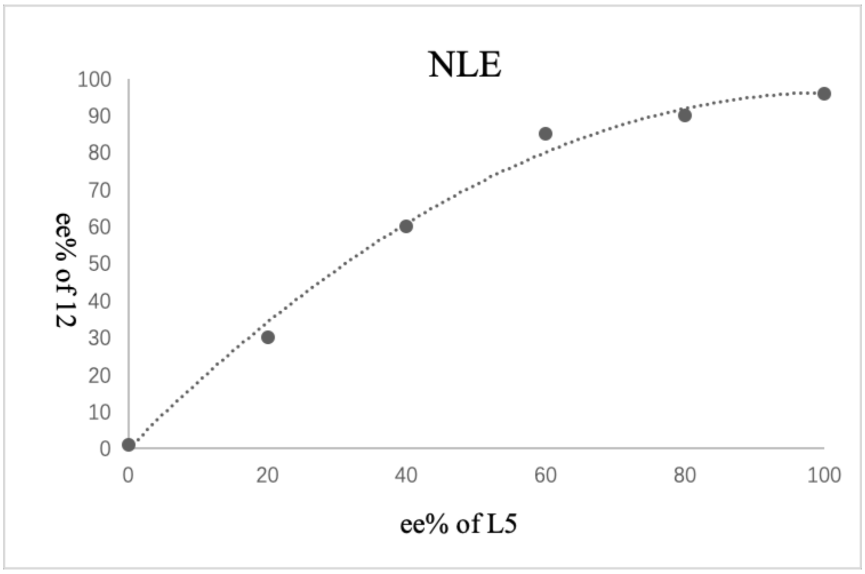

Supplementary Figure 1. Positive NLE in the APS reaction of propargyl carbonate **10a** and β-keto ester **11a (Cu:L5 = 1:2)**

As with prior studies on the APS reaction, we observed a nonlinear relationship between the *ee* value of optically active ligand **L5** and the *ee* value of product **12** as shown in Supplementary Table 6. These results are consistent with the notion that multiligand species plays a role in this transformation.

HPLC Traces

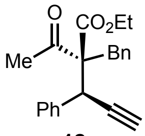

12

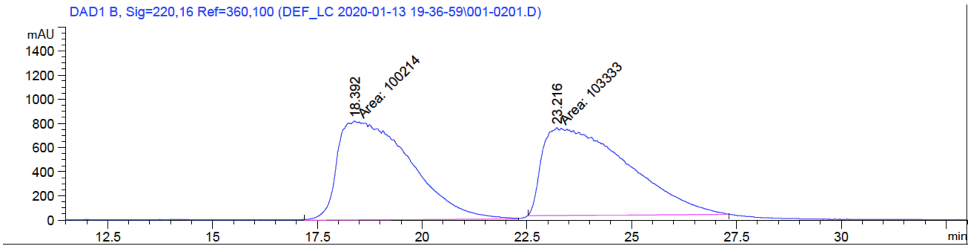

Signal 2: DAD1 B, Sig=220,16 Ref=360,100

Peak #	RetTime [min]	Type	Width [min]	Area [mAU*s]	Height [mAU]	Area %
1	18.392	MM	2.0399	1.00214e5	818.79877	49.2340
2	23.216	MM	2.3685	1.03333e5	727.12256	50.7660

Totals : 2.03547e5 1545.92133

Supplementary Figure 2. HPLC spectra for compound ***rac*-12**

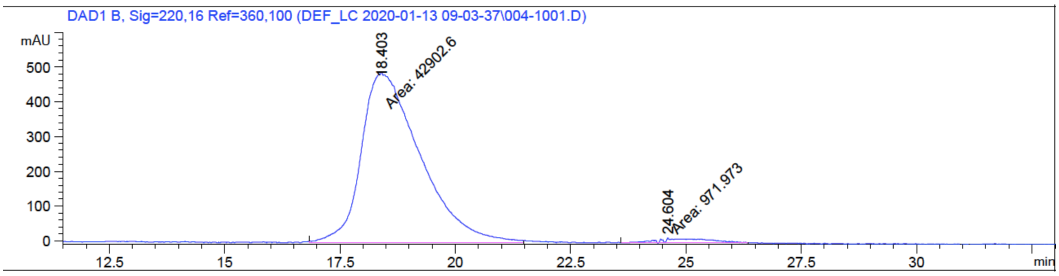

Signal 2: DAD1 B, Sig=220,16 Ref=360,100

Peak #	RetTime [min]	Type	Width [min]	Area [mAU*s]	Height [mAU]	Area %
1	18.403	MM	1.4620	4.29026e4	489.09283	97.7847
2	24.604	MM	1.2003	971.97302	13.49667	2.2153

Totals : 4.38746e4 502.58950

Supplementary Figure 3. HPLC spectra for compound **12**

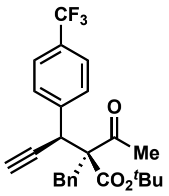

14a

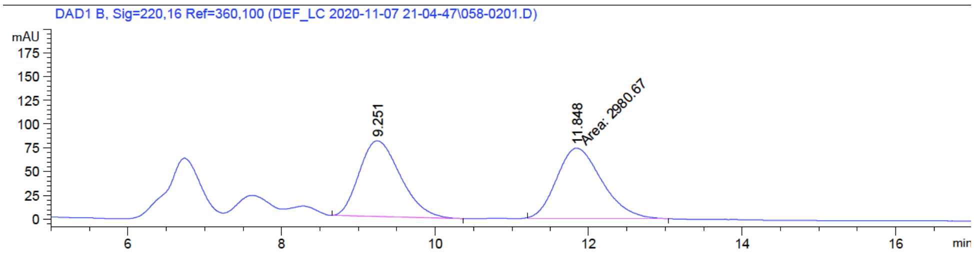

Signal 2: DAD1 B, Sig=220,16 Ref=360,100

Peak #	RetTime [min]	Type	Width [min]	Area [mAU*s]	Height [mAU]	Area %
1	9.251	BB	0.5785	2964.51660	79.60340	49.8642
2	11.848	MM	0.6767	2980.66626	73.41174	50.1358

Totals : 5945.18286 153.01514

Supplementary Figure 4. HPLC spectra for compound ***rac*-14a**

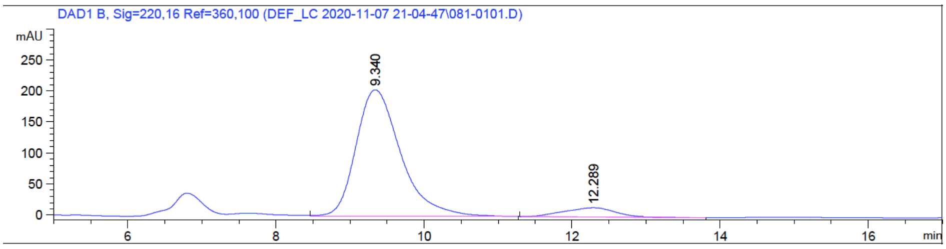

Signal 2: DAD1 B, Sig=220,16 Ref=360,100

Peak #	RetTime [min]	Type	Width [min]	Area [mAU*s]	Height [mAU]	Area %
1	9.340	VB	0.6453	8635.32715	204.21085	92.1539
2	12.289	BB	0.6618	735.22168	15.16034	7.8461

Totals : 9370.54883 219.37119

Supplementary Figure 5. HPLC spectra for compound **14a**

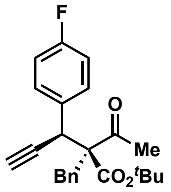

14b

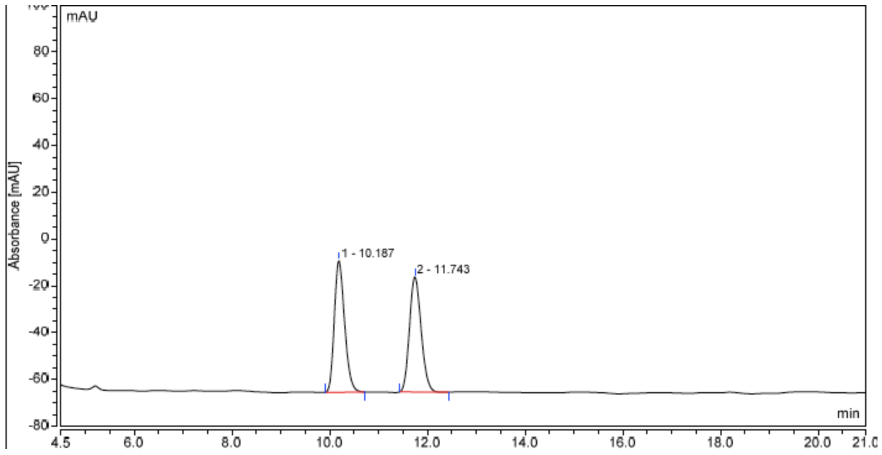

Peak	RetTime min	Area mAU*min	Height mAU	Area %	Height %
1	10.187	13.748	56.367	50.32	53.36
2	11.743	13.573	49.264	49.68	46.64
**Total**		**27.321**	**105.630**	**100.00**	**100.00**

Supplementary Figure 6. HPLC spectra for compound ***rac*-14b**

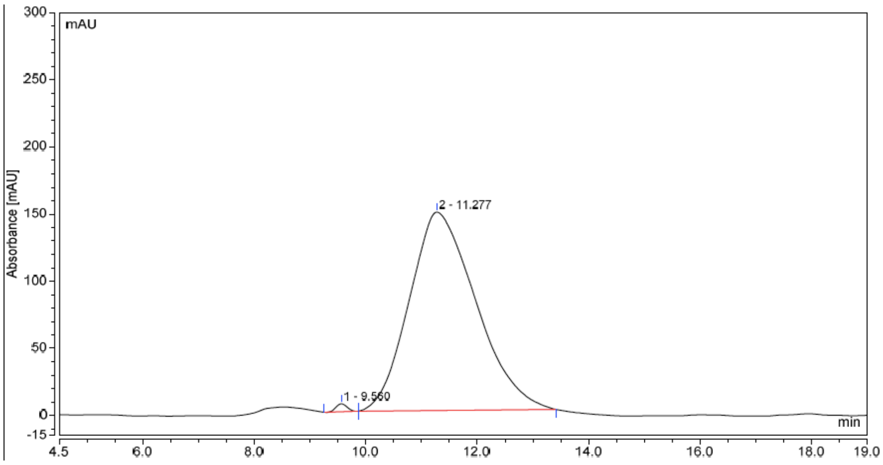

Peak	RetTime min	Area mAU*min	Height mAU	Area %	Height %
1	9.560	1.343	5.911	0.65	3.84
2	11.277	205.406	147.901	99.35	96.16
**Total**		**206.749**	**153.812**	**100.00**	**100.00**

Supplementary Figure 7. HPLC spectra for compound **14b**

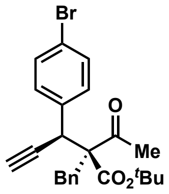

14c

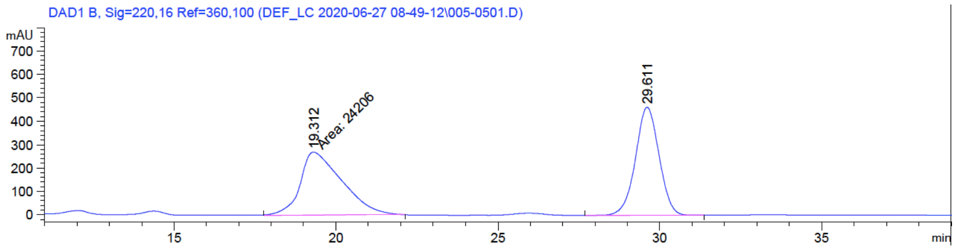

Signal 2: DAD1 B, Sig=220,16 Ref=360,100

Peak #	RetTime [min]	Type	Width [min]	Area [mAU*s]	Height [mAU]	Area %
1	19.312	MM	1.4967	2.42060e4	269.55298	50.8147
2	29.611	BB	0.7824	2.34299e4	461.41211	49.1853

Totals : 4.76359e4 730.96509

Supplementary Figure 8. HPLC spectra for compound ***rac*-14c**

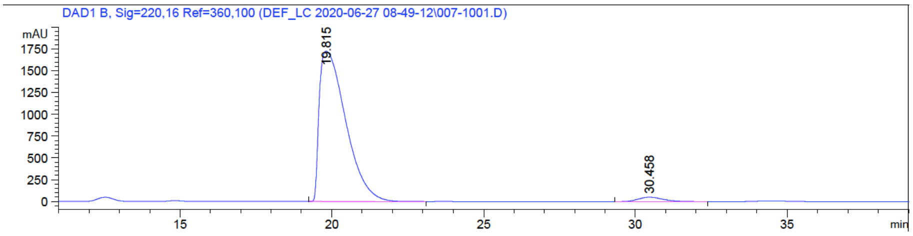

Signal 2: DAD1 B, Sig=220,16 Ref=360,100

Peak #	RetTime [min]	Type	Width [min]	Area [mAU*s]	Height [mAU]	Area %
1	19.815	BB	0.9344	1.04163e5	1720.30579	97.1901
2	30.458	BB	0.8112	3011.46851	51.12467	2.8099

Totals : 1.07174e5 1771.43045

Supplementary Figure 9. HPLC spectra for compound **14c**

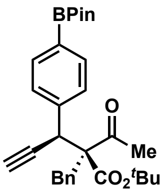

14d

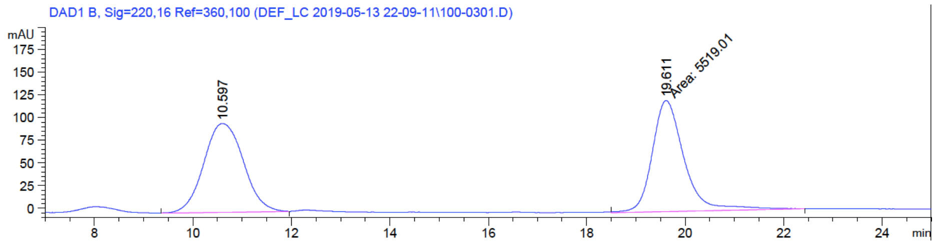

Signal 2: DAD1 B, Sig=220,16 Ref=360,100

Peak #	RetTime [min]	Type	Width [min]	Area [mAU*s]	Height [mAU]	Area %
1	10.597	BB	0.8368	5460.88379	97.90115	49.7353
2	19.611	MM	0.7504	5519.01416	122.58739	50.2647

Totals : 1.09799e4 220.48855

Supplementary Figure 10. HPLC spectra for compound ***rac*-14d**

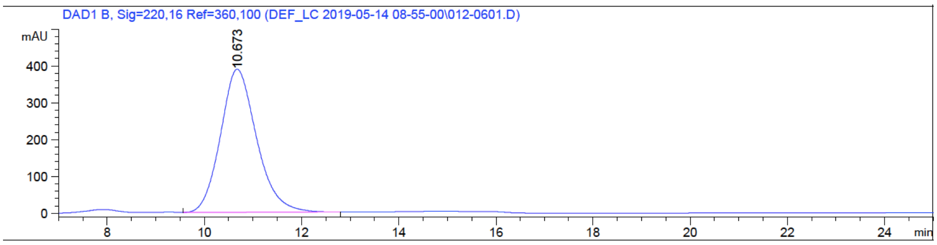

Signal 2: DAD1 B, Sig=220,16 Ref=360,100

Peak #	RetTime [min]	Type	Width [min]	Area [mAU*s]	Height [mAU]	Area %
1	10.673	BB	0.7790	2.00537e4	387.99237	100.0000

Totals : 2.00537e4 387.99237

Supplementary Figure 11. HPLC spectra for compound **14d**

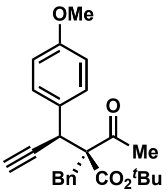

14e

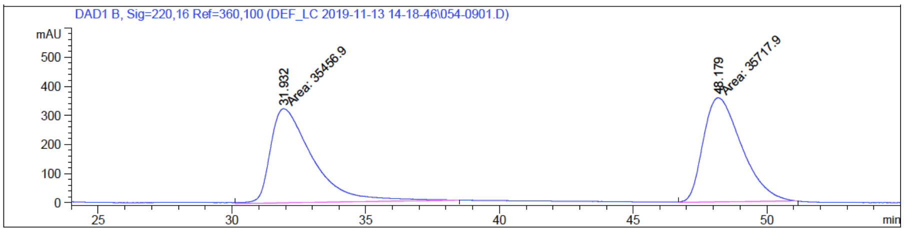

Signal 2: DAD1 B, Sig=220,16 Ref=360,100

Peak #	RetTime [min]	Type	Width [min]	Area [mAU*s]	Height [mAU]	Area %
1	31.932	MM	1.8273	3.54569e4	323.40875	49.8166
2	48.179	MM	1.6663	3.57179e4	357.25781	50.1834
Totals :				7.11749e4	680.66656	

Supplementary Figure 12. HPLC spectra for compound ***rac*-14e**

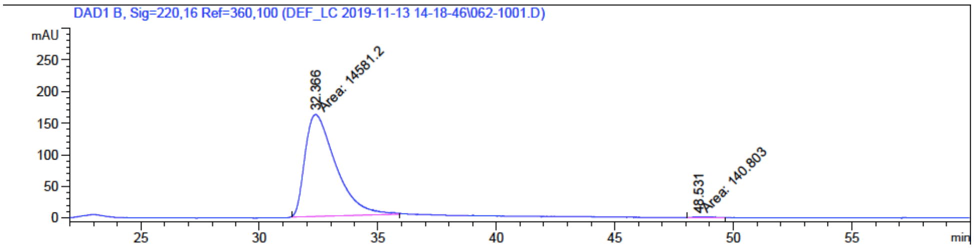

Signal 2: DAD1 B, Sig=220,16 Ref=360,100

Peak #	RetTime [min]	Type	Width [min]	Area [mAU*s]	Height [mAU]	Area %
1	32.366	MM	1.5077	1.45812e4	161.18269	99.0436
2	48.531	MM	1.2635	140.80345	1.85727	0.9564
Totals :				1.47220e4	163.03997	

Supplementary Figure 13. HPLC spectra for compound **14e**

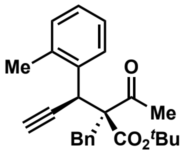

14f

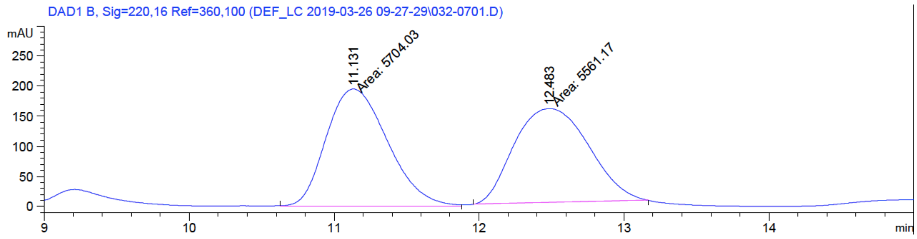

Signal 2: DAD1 B, Sig=220,16 Ref=360,100

Peak #	RetTime [min]	Type	Width [min]	Area [mAU*s]	Height [mAU]	Area %
1	11.131	MM	0.4894	5704.03467	194.26169	50.6341
2	12.483	MM	0.5958	5561.17236	155.56985	49.3659

Totals : 1.12652e4 349.83154

Supplementary Figure 14. HPLC spectra for compound ***rac*-14f**

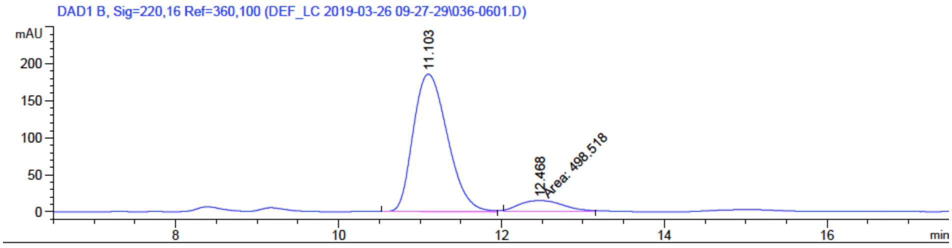

Signal 2: DAD1 B, Sig=220,16 Ref=360,100

Peak #	RetTime [min]	Type	Width [min]	Area [mAU*s]	Height [mAU]	Area %
1	11.103	BV	0.4659	5402.20068	185.40244	91.5516
2	12.468	MM	0.6074	498.51770	13.67877	8.4484

Totals : 5900.71838 199.08121

Supplementary Figure 15. HPLC spectra for compound **14f**

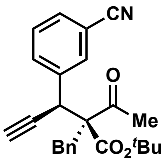

14g

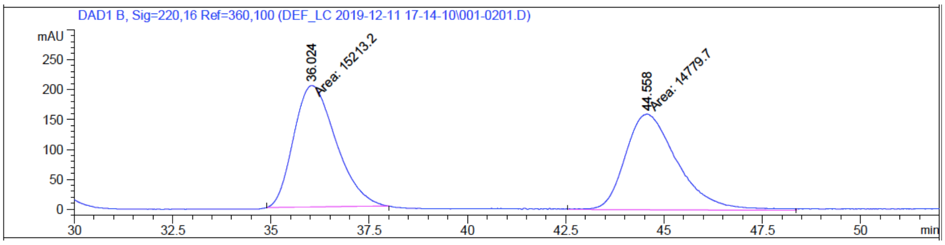

Signal 2: DAD1 B, Sig=220,16 Ref=360,100

Peak #	RetTime [min]	Type	Width [min]	Area [mAU*s]	Height [mAU]	Area %
1	36.024	MM	1.2494	1.52132e4	202.94649	50.7227
2	44.558	MM	1.5385	1.47797e4	160.10620	49.2773
Totals :				2.99929e4	363.05269	

Supplementary Figure 16. HPLC spectra for compound ***rac*-14g**

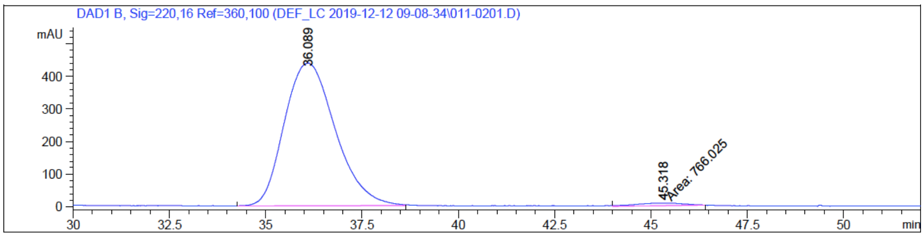

Signal 2: DAD1 B, Sig=220,16 Ref=360,100

Peak #	RetTime [min]	Type	Width [min]	Area [mAU*s]	Height [mAU]	Area %
1	36.089	BV	1.0834	4.02982e4	438.80569	98.1346
2	45.318	MM	1.5136	766.02509	8.43470	1.8654
Totals :				4.10642e4	447.24040	

Supplementary Figure 17. HPLC spectra for compound **14g**

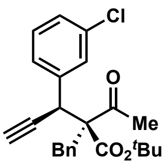

14h

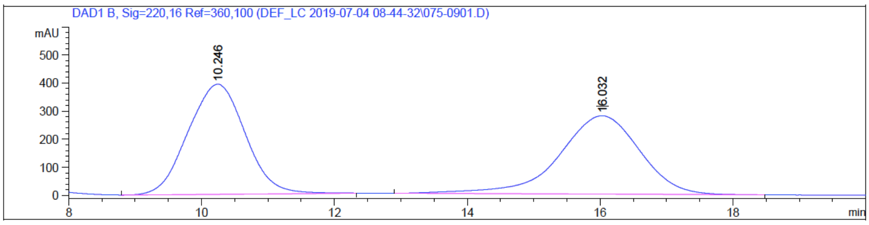

Signal 2: DAD1 B, Sig=220,16 Ref=360,100

Peak #	RetTime [min]	Type	Width [min]	Area [mAU*s]	Height [mAU]	Area %
1	10.246	BB	0.8865	2.33705e4	391.46991	49.7365
2	16.032	BB	1.0585	2.36181e4	278.75464	50.2635
Totals :				4.69886e4	670.22455	

Supplementary Figure 18. HPLC spectra for compound ***rac*-14h**

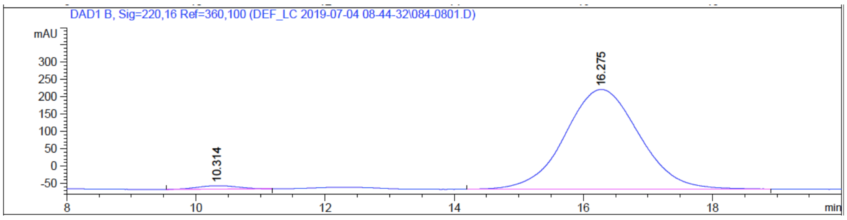

Signal 2: DAD1 B, Sig=220,16 Ref=360,100

Peak #	RetTime [min]	Type	Width [min]	Area [mAU*s]	Height [mAU]	Area %
1	10.314	BB	0.5174	414.57458	9.60882	1.7691
2	16.275	BB	1.0923	2.30202e4	287.45813	98.2309
Totals :				2.34348e4	297.06695	

Supplementary Figure 19. HPLC spectra for compound **14h**

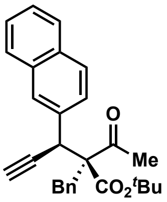

14i

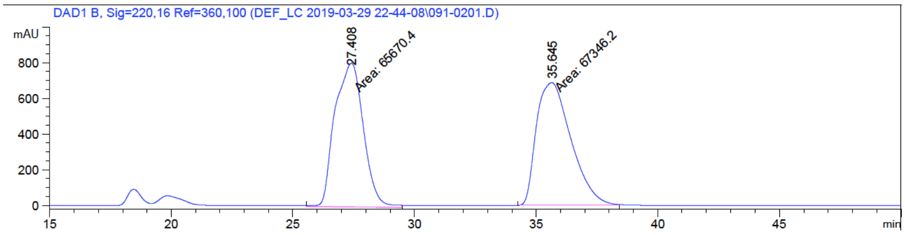

Signal 2: DAD1 B, Sig=220,16 Ref=360,100

Peak #	RetTime [min]	Type	Width [min]	Area [mAU*s]	Height [mAU]	Area %
1	27.408	MM	1.3615	6.56704e4	803.91290	49.3701
2	35.645	MM	1.6413	6.73462e4	683.85284	50.6299

Totals : 1.33017e5 1487.76575

Supplementary Figure 20. HPLC spectra for compound ***rac*-14i**

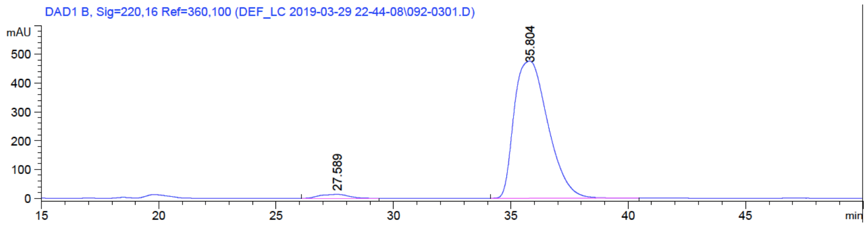

Signal 2: DAD1 B, Sig=220,16 Ref=360,100

Peak #	RetTime [min]	Type	Width [min]	Area [mAU*s]	Height [mAU]	Area %
1	27.589	BB	0.9754	1131.19592	13.58584	2.3221
2	35.804	BB	1.5655	4.75829e4	475.47284	97.6779

Totals : 4.87141e4 489.05868

Supplementary Figure 21. HPLC spectra for compound **14i**

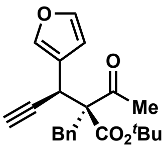

14j

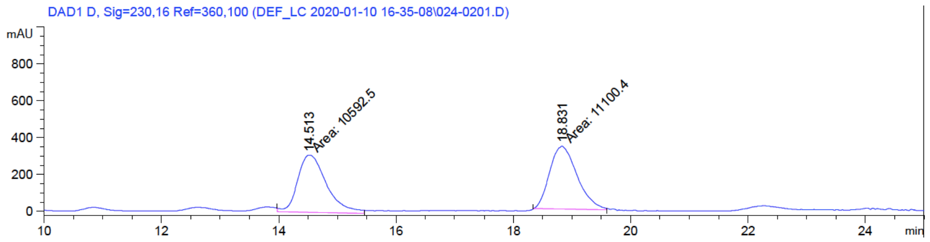

Signal 4: DAD1 D, Sig=230,16 Ref=360,100

Peak #	RetTime [min]	Type	Width [min]	Area [mAU*s]	Height [mAU]	Area %
1	14.513	MM	0.5707	1.05925e4	309.36871	48.8293
2	18.831	MM	0.5403	1.11004e4	342.41940	51.1707
Totals :				2.16930e4	651.78812	

Supplementary Figure 22. HPLC spectra for compound ***rac*-14j**

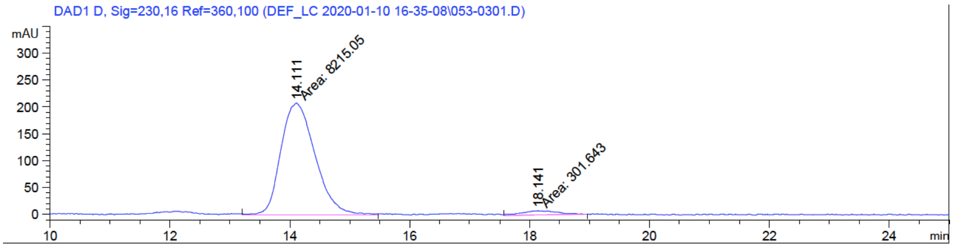

Signal 4: DAD1 D, Sig=230,16 Ref=360,100

Peak #	RetTime [min]	Type	Width [min]	Area [mAU*s]	Height [mAU]	Area %
1	14.111	MM	0.6595	8215.04883	207.59305	96.4582
2	18.141	MM	0.6595	301.64307	7.62358	3.5418
Totals :				8516.69189	215.21662	

Supplementary Figure 23. HPLC spectra for compound **14j**

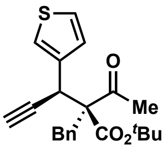

14k

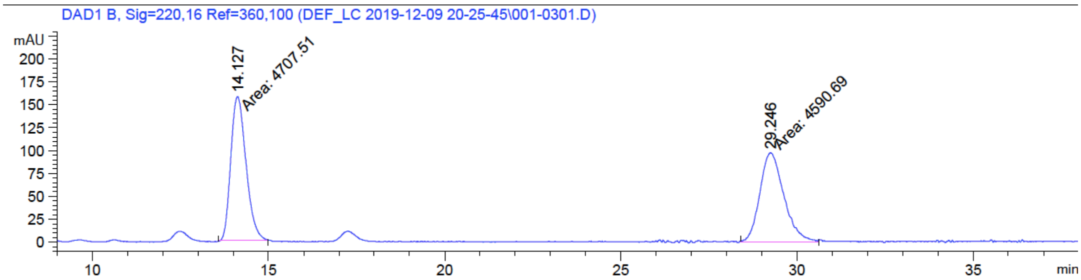

Signal 2: DAD1 B, Sig=220,16 Ref=360,100

Peak #	RetTime [min]	Type	Width [min]	Area [mAU*s]	Height [mAU]	Area %
1	14.127	MM	0.5001	4707.51123	156.88808	50.6282
2	29.246	MM	0.7878	4590.69482	97.12024	49.3718
Totals :				9298.20605	254.00832	

Supplementary Figure 24. HPLC spectra for compound ***rac*-14k**

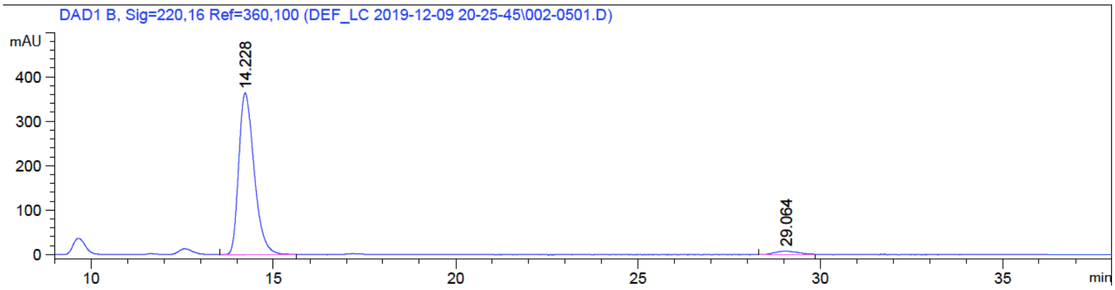

Signal 2: DAD1 B, Sig=220,16 Ref=360,100

Peak #	RetTime [min]	Type	Width [min]	Area [mAU*s]	Height [mAU]	Area %
1	14.228	BV	0.4461	1.06642e4	363.66968	96.9526
2	29.064	BV	0.5233	335.19168	7.61871	3.0474
Totals :				1.09994e4	371.28838	

Supplementary Figure 25. HPLC spectra for compound **14k**

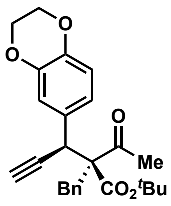

14l

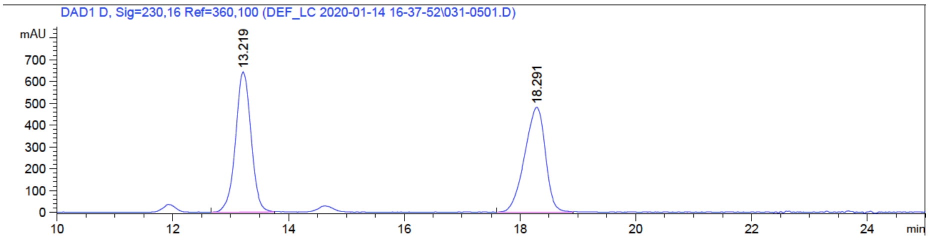

Signal 4: DAD1 D, Sig=230,16 Ref=360,100

Peak #	RetTime [min]	Type	Width [min]	Area [mAU*s]	Height [mAU]	Area %
1	13.219	BV	0.2800	1.19987e4	642.09833	49.9679
2	18.291	BV	0.3609	1.20141e4	481.83374	50.0321
Totals :				2.40128e4	1123.93207	

Supplementary Figure 26. HPLC spectra for compound ***rac*-14l**

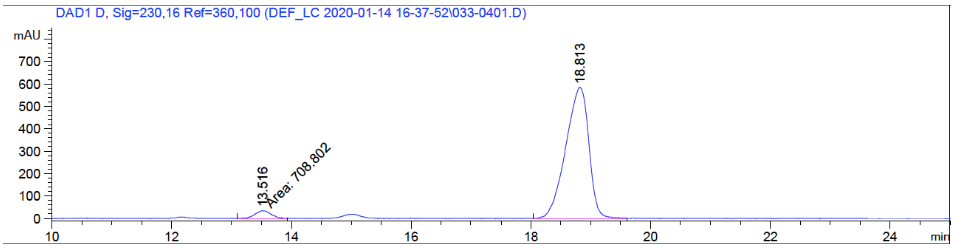

Signal 4: DAD1 D, Sig=230,16 Ref=360,100

Peak #	RetTime [min]	Type	Width [min]	Area [mAU*s]	Height [mAU]	Area %
1	13.516	MM	0.3311	708.80200	35.68075	4.3102
2	18.813	VV	0.3709	1.57359e4	586.72430	95.6898
Totals :				1.64447e4	622.40506	

Supplementary Figure 27. HPLC spectra for compound **14l**

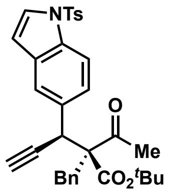

14m

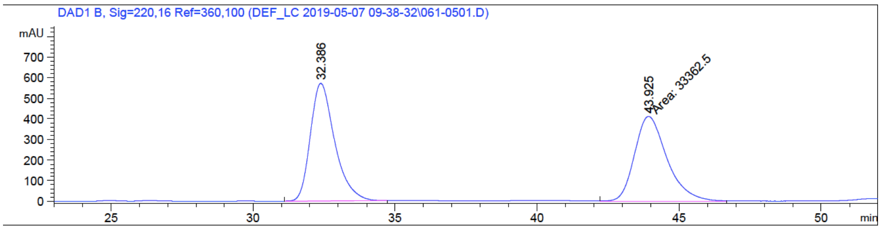

Signal 2: DAD1 B, Sig=220,16 Ref=360,100

Peak #	RetTime [min]	Type	Width [min]	Area [mAU*s]	Height [mAU]	Area %
1	32.386	BB	0.8990	3.39996e4	570.73798	50.4729
2	43.925	MM	1.3417	3.33625e4	414.43634	49.5271

Totals : 6.73621e4 985.17432

Supplementary Figure 28. HPLC spectra for compound ***rac*-14m**

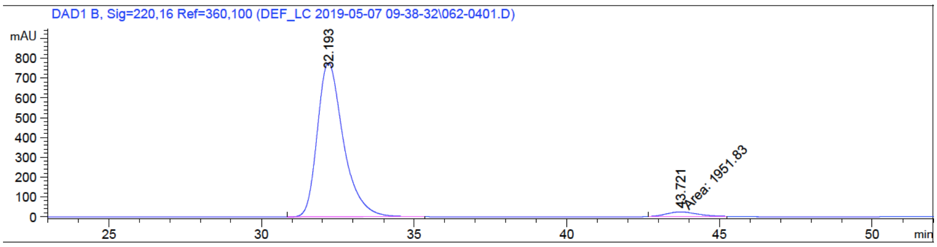

Signal 2: DAD1 B, Sig=220,16 Ref=360,100

Peak #	RetTime [min]	Type	Width [min]	Area [mAU*s]	Height [mAU]	Area %
1	32.193	BB	0.8937	4.60397e4	776.58789	95.9330
2	43.721	MM	1.3008	1951.83044	25.00811	4.0670

Totals : 4.79916e4 801.59600

Supplementary Figure 29. HPLC spectra for compound **14m**

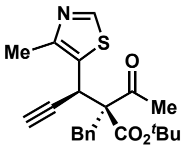

14n

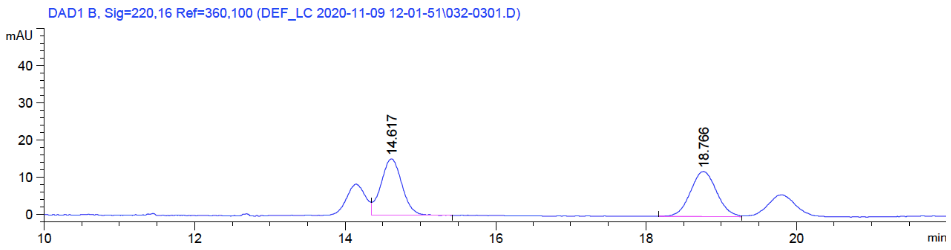

Signal 2: DAD1 B, Sig=220,16 Ref=360,100

Peak #	RetTime [min]	Type	Width [min]	Area [mAU*s]	Height [mAU]	Area %
1	14.617	VB	0.2996	295.73724	15.16040	50.3523
2	18.766	BV	0.3741	291.59882	12.05772	49.6477
Totals :				587.33606	27.21811	

Supplementary Figure 30. HPLC spectra for compound ***rac*-14n**

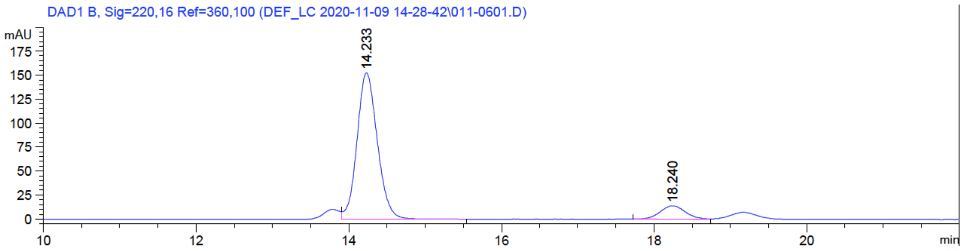

Signal 2: DAD1 B, Sig=220,16 Ref=360,100

Peak #	RetTime [min]	Type	Width [min]	Area [mAU*s]	Height [mAU]	Area %
1	14.233	VB	0.2848	2838.67651	152.74254	89.8981
2	18.240	BV	0.3630	318.98517	13.94018	10.1019
Totals :				3157.66168	166.68272	

Supplementary Figure 31. HPLC spectra for compound **14n**

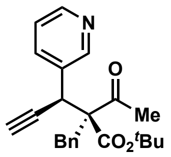

14o

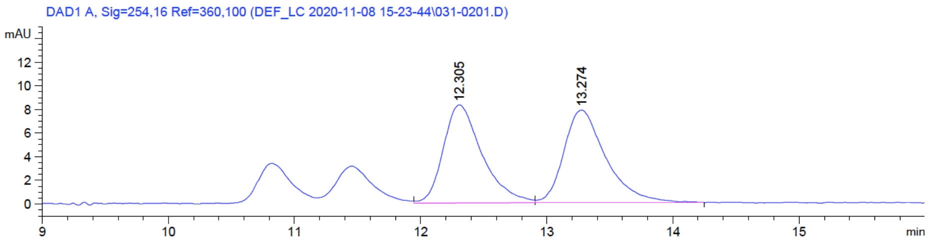

Signal 1: DAD1 A, Sig=254,16 Ref=360,100

Peak #	RetTime [min]	Type	Width [min]	Area [mAU*s]	Height [mAU]	Area %
1	12.305	VV	0.3262	178.05727	8.30385	49.4224
2	13.274	VB	0.3468	182.21948	7.85094	50.5776
Totals :				360.27675	16.15479	

Supplementary Figure 32. HPLC spectra for compound ***rac*-14o**

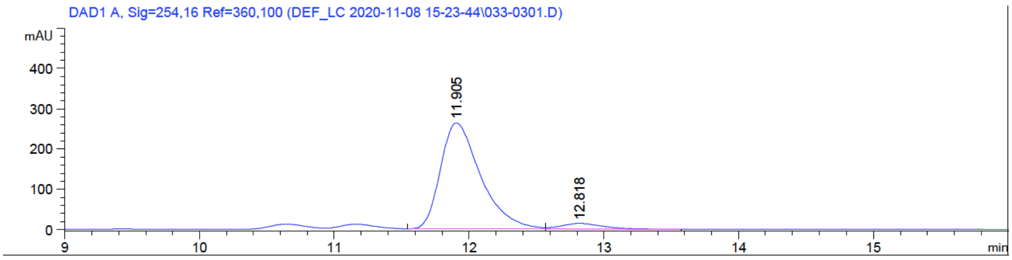

Signal 1: DAD1 A, Sig=254,16 Ref=360,100

Peak #	RetTime [min]	Type	Width [min]	Area [mAU*s]	Height [mAU]	Area %
1	11.905	BV	0.3138	5447.55273	262.84848	94.5844
2	12.818	VB	0.3219	311.91205	14.33420	5.4156
Totals :				5759.46478	277.18268	

Supplementary Figure 33. HPLC spectra for compound **14o**

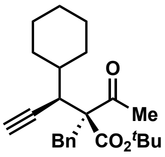

14p

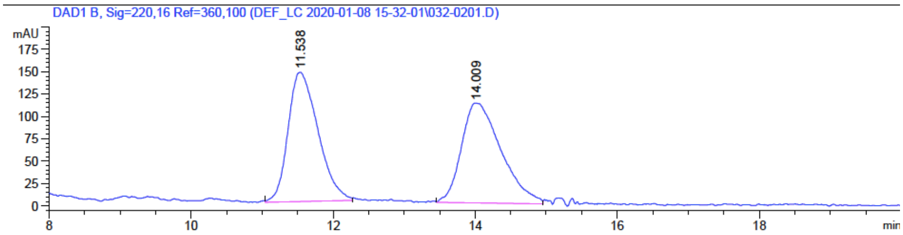


```
Signal 2: DAD1 B, Sig=220,16 Ref=360,100

Peak RetTime Type  Width     Area       Height     Area
  #   [min]        [min]   [mAU*s]      [mAU]        %
----|-------|----|-------|----------|----------|--------|
   1  11.538 VV     0.3537 4226.83252  144.31833  49.3196
   2  14.009 VV     0.4977 4343.46387  111.68843  50.6804

Totals :                   8570.29639  256.00676
```

Supplementary Figure 34. HPLC spectra for compound ***rac*-14p**

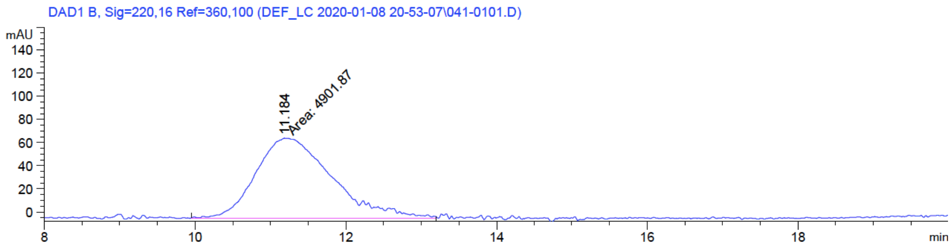


```
Signal 2: DAD1 B, Sig=220,16 Ref=360,100

Peak RetTime Type  Width     Area       Height     Area
  #   [min]        [min]   [mAU*s]      [mAU]        %
----|-------|----|-------|----------|----------|--------|
   1  11.184 MM     1.1747 4901.87305   69.55038 100.0000

Totals :                   4901.87305   69.55038
```

Supplementary Figure 35. HPLC spectra for compound **14p**

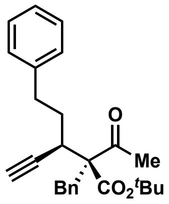

14q

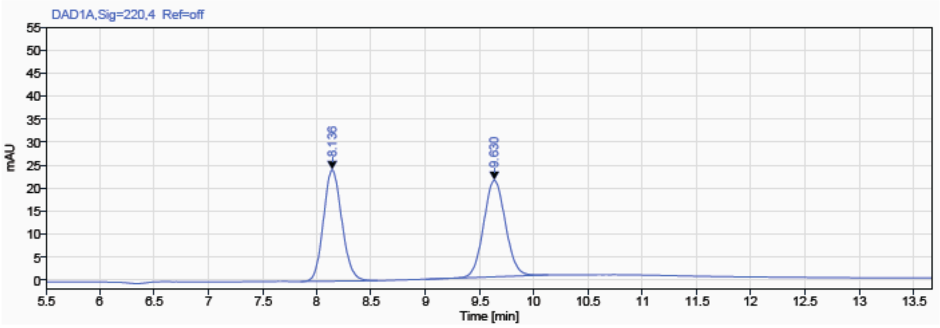

Signal:	DAD1A,Sig=220,4 Ref=off					
RT [min]	Type	Width [min]	Area	Height	Area%	Name
8.136	BB	0.71	284.18	24.09	49.27	
9.630	BB	1.12	292.61	20.88	50.73	
		Sum	576.79			

Supplementary Figure 36. HPLC spectra for compound ***rac*-14q**

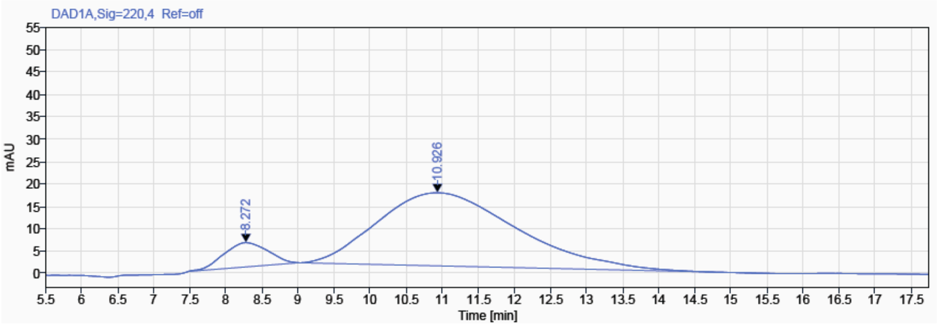

Signal:	DAD1A,Sig=220,4 Ref=off					
RT [min]	Type	Width [min]	Area	Height	Area%	Name
8.272	MM m	0.59	230.61	5.42	9.52	
10.926	MB m	1.58	2192.53	16.31	90.48	
		Sum	2423.14			

Supplementary Figure 37. HPLC spectra for compound **14q**

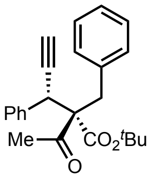

15a

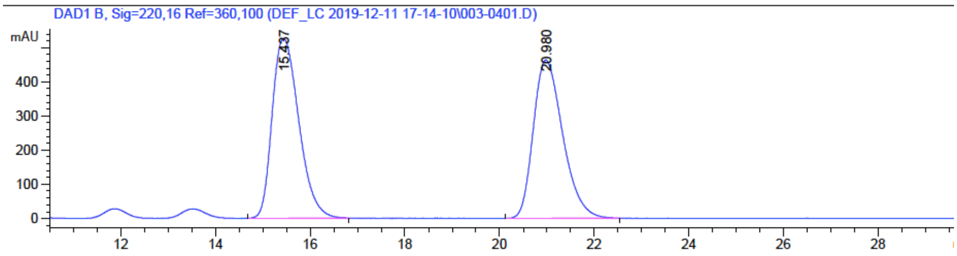

Signal 2: DAD1 B, Sig=220,16 Ref=360,100

Peak #	RetTime [min]	Type	Width [min]	Area [mAU*s]	Height [mAU]	Area %
1	15.437	BV	0.5764	2.06201e4	527.07678	50.7766
2	20.980	BB	0.6213	1.99893e4	463.39767	49.2234
Totals :				4.06094e4	990.47446	

Supplementary Figure 38. HPLC spectra for compound ***rac*-15a**

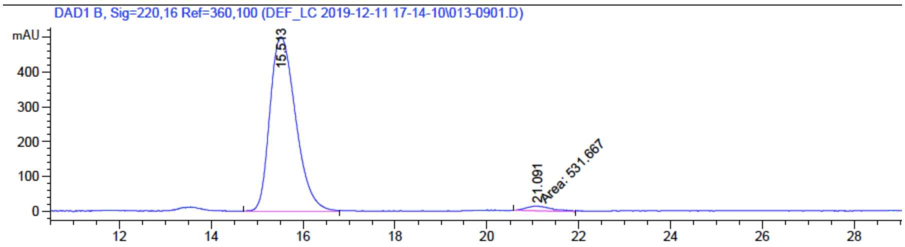

Signal 2: DAD1 B, Sig=220,16 Ref=360,100

Peak #	RetTime [min]	Type	Width [min]	Area [mAU*s]	Height [mAU]	Area %
1	15.513	BV	0.5894	1.94751e4	501.15088	97.3426
2	21.091	MM	0.6708	531.66711	13.21009	2.6574
Totals :				2.00067e4	514.36097	

Supplementary Figure 39. HPLC spectra for compound **15a**

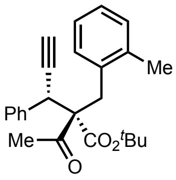

15b

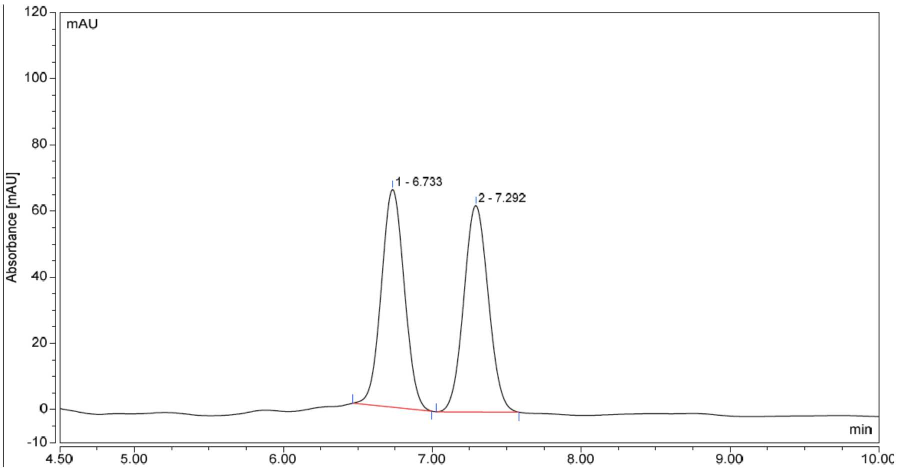

Peak	RetTime<br>min	Area<br>mAU*min	Height<br>mAU	Area<br>%	Height<br>%
1	6.733	11.776	65.819	49.68	51.33
2	7.292	11.929	62.408	50.32	48.67
**Total**		**23.705**	**128.227**	**100.00**	**100.00**

Supplementary Figure 40. HPLC spectra for compound ***rac*-15b**

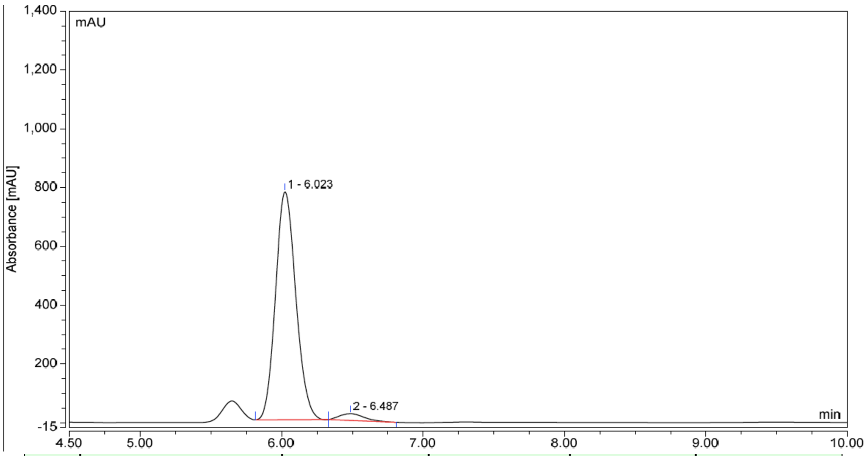

Peak	RetTime<br>min	Area<br>mAU*min	Height<br>mAU	Area<br>%	Height<br>%
1	6.023	129.117	775.745	96.51	97.11
2	6.487	4.664	23.123	3.49	2.89
**Total**		**133.781**	**798.867**	**100.00**	**100.00**

Supplementary Figure 41. HPLC spectra for compound **15b**

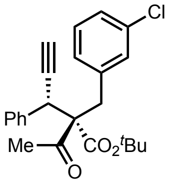

15c

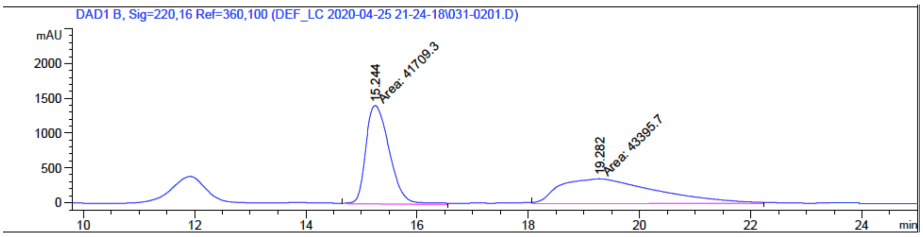

Signal 2: DAD1 B, Sig=220,16 Ref=360,100

Peak #	RetTime [min]	Type	Width [min]	Area [mAU*s]	Height [mAU]	Area %
1	15.244	MM	0.4927	4.17093e4	1410.77820	49.0092
2	19.282	MM	2.0257	4.33957e4	357.04108	50.9908
Totals :				8.51050e4	1767.81927	

Supplementary Figure 42. HPLC spectra for compound ***rac*-15c**

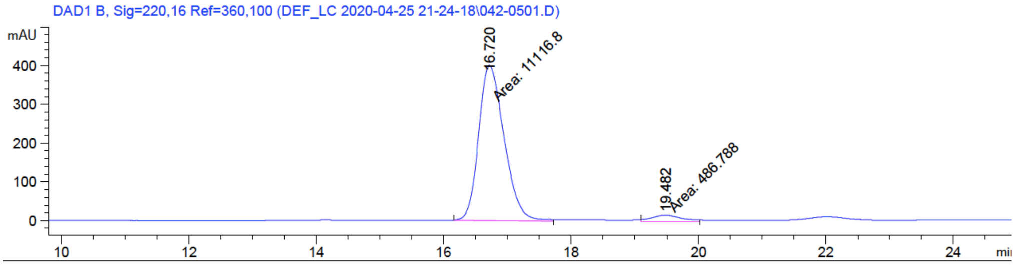

Signal 2: DAD1 B, Sig=220,16 Ref=360,100

Peak #	RetTime [min]	Type	Width [min]	Area [mAU*s]	Height [mAU]	Area %
1	16.720	MM	0.4625	1.11168e4	400.63376	95.8048
2	19.482	MM	0.5343	486.78848	15.18523	4.1952
Totals :				1.16036e4	415.81898	

Supplementary Figure 43. HPLC spectra for compound **15c**

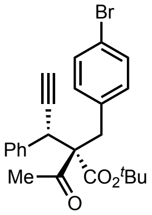

15d

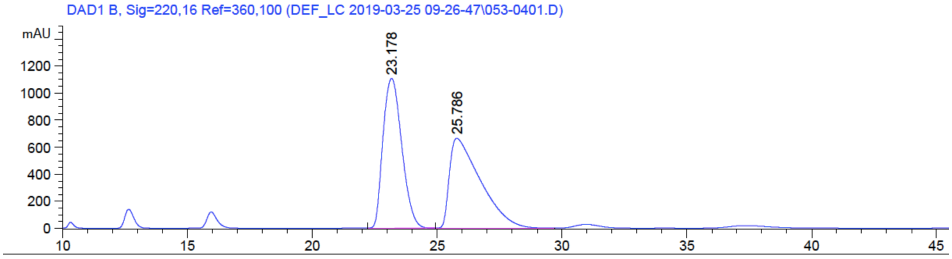

Signal 2: DAD1 B, Sig=220,16 Ref=360,100

Peak #	RetTime [min]	Type	Width [min]	Area [mAU*s]	Height [mAU]	Area %
1	23.178	VB	0.8444	5.79431e4	1107.47583	50.2607
2	25.786	BB	1.2404	5.73419e4	664.75446	49.7393
Totals :				1.15285e5	1772.23029	

Supplementary Figure 44. HPLC spectra for compound ***rac*-15d**

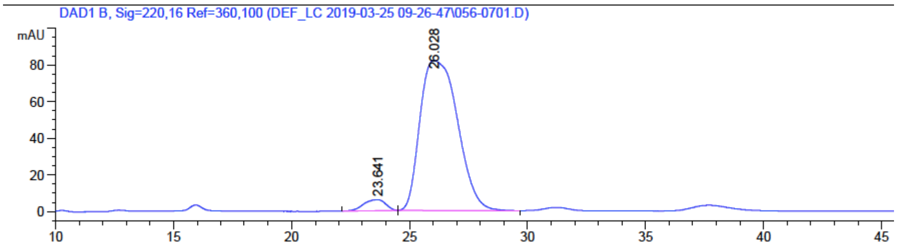

Signal 2: DAD1 B, Sig=220,16 Ref=360,100

Peak #	RetTime [min]	Type	Width [min]	Area [mAU*s]	Height [mAU]	Area %
1	23.641	BB	0.7746	381.24332	5.92605	4.0486
2	26.028	BB	1.5111	9035.41406	81.76974	95.9514
Totals :				9416.65738	87.69579	

Supplementary Figure 45. HPLC spectra for compound **15d**

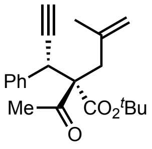

15e

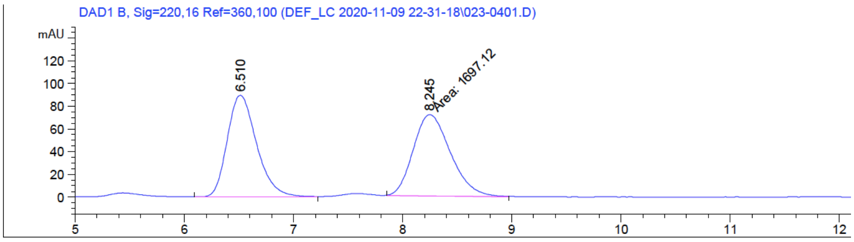

Signal 2: DAD1 B, Sig=220,16 Ref=360,100

Peak #	RetTime [min]	Type	Width [min]	Area [mAU*s]	Height [mAU]	Area %
1	6.510	BV	0.2903	1674.62646	89.49805	49.6664
2	8.245	MM	0.3950	1697.12097	71.60973	50.3336

Totals : 3371.74744 161.10778

Supplementary Figure 46. HPLC spectra for compound ***rac*-15e**

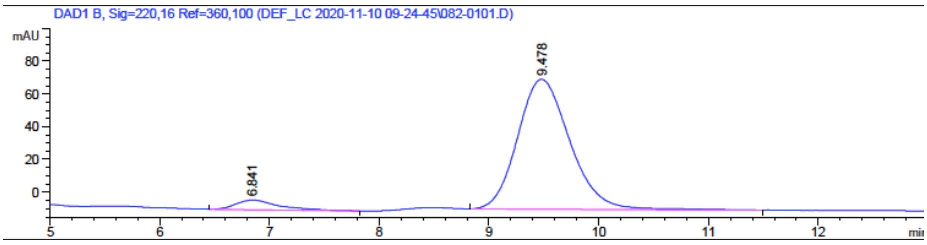

Signal 2: DAD1 B, Sig=220,16 Ref=360,100

Peak #	RetTime [min]	Type	Width [min]	Area [mAU*s]	Height [mAU]	Area %
1	6.841	BB	0.3526	158.44688	5.80159	5.6819
2	9.478	BB	0.5049	2630.20093	79.31929	94.3181

Totals : 2788.64781 85.12088

Supplementary Figure 47. HPLC spectra for compound **15e**

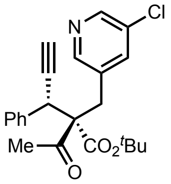

15f

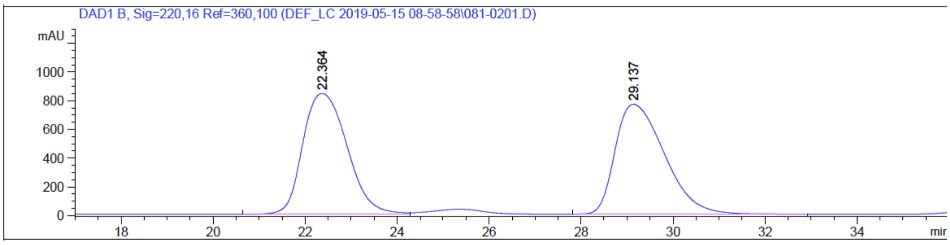

Signal 2: DAD1 B, Sig=220,16 Ref=360,100

Peak #	RetTime [min]	Type	Width [min]	Area [mAU*s]	Height [mAU]	Area %
1	22.364	BV	1.0491	5.65127e4	841.94672	49.7281
2	29.137	BB	1.0671	5.71306e4	765.13214	50.2719
Totals :				1.13643e5	1607.07886	

Supplementary Figure 48. HPLC spectra for compound ***rac*-15f**

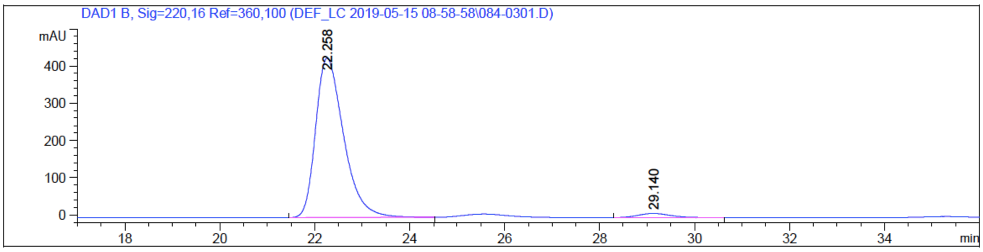

Signal 2: DAD1 B, Sig=220,16 Ref=360,100

Peak #	RetTime [min]	Type	Width [min]	Area [mAU*s]	Height [mAU]	Area %
1	22.258	BB	0.6304	1.75290e4	427.49097	97.0769
2	29.140	BB	0.5713	527.82758	11.04917	2.9231
Totals :				1.80568e4	438.54014	

Supplementary Figure 49. HPLC spectra for compound **15f**

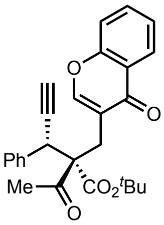

15g

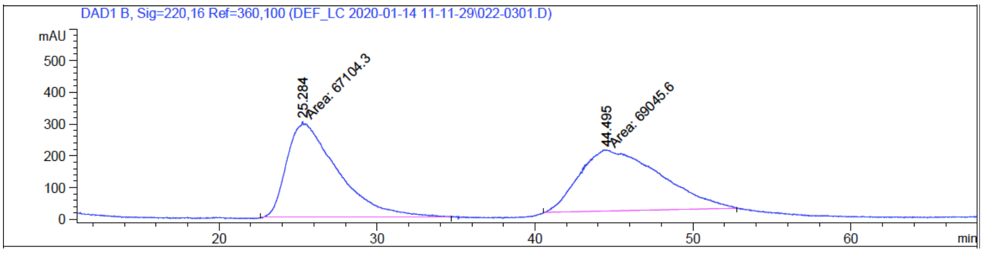

Signal 2: DAD1 B, Sig=220,16 Ref=360,100

Peak #	RetTime [min]	Type	Width [min]	Area [mAU*s]	Height [mAU]	Area %
1	25.284	MM	3.7057	6.71043e4	301.80508	49.2871
2	44.495	MM	5.9817	6.90456e4	192.38084	50.7129
Totals :				1.36150e5	494.18593	

Supplementary Figure 50. HPLC spectra for compound ***rac*-15g**

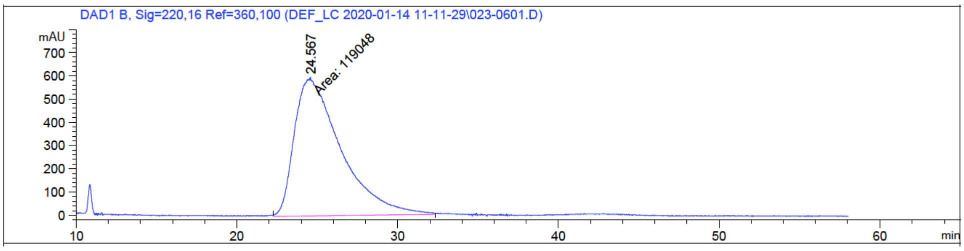

Signal 2: DAD1 B, Sig=220,16 Ref=360,100

Peak #	RetTime [min]	Type	Width [min]	Area [mAU*s]	Height [mAU]	Area %
1	24.567	MM	3.3322	1.19048e5	595.44843	100.0000
Totals :				1.19048e5	595.44843	

Supplementary Figure 51. HPLC spectra for compound **15g**

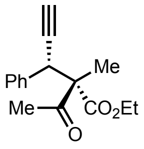

15h

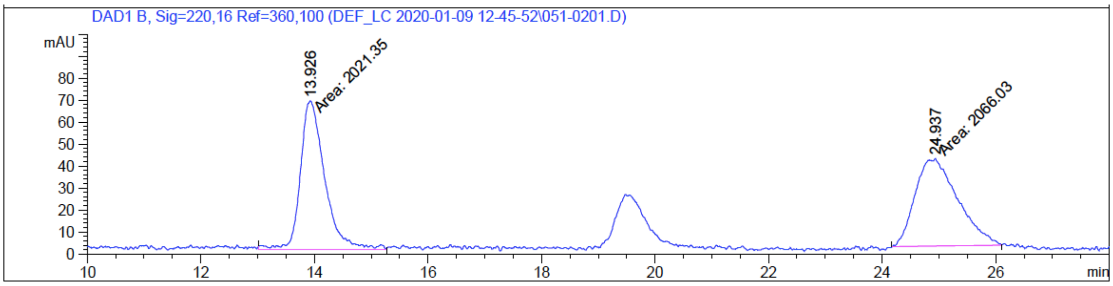

Peak #	RetTime [min]	Type	Width [min]	Area [mAU*s]	Height [mAU]	Area %
1	13.926	MM	0.4989	2021.34534	67.52643	49.4533
2	24.937	MM	0.8664	2066.03296	39.74272	50.5467
Totals :				4087.37830	107.26915	

Supplementary Figure 52. HPLC spectra for compound ***rac*-15h**

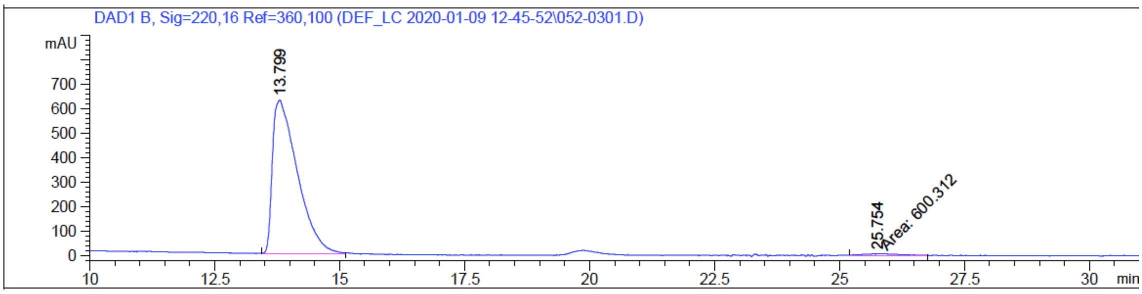

Signal 2: DAD1 B, Sig=220,16 Ref=360,100

Peak #	RetTime [min]	Type	Width [min]	Area [mAU*s]	Height [mAU]	Area %
1	13.799	VV	0.4216	2.17688e4	626.16974	97.3163
2	25.754	MM	1.0393	600.31152	9.62641	2.6837
Totals :				2.23692e4	635.79615	

Supplementary Figure 53. HPLC spectra for compound **15h**

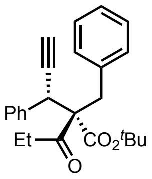

15i

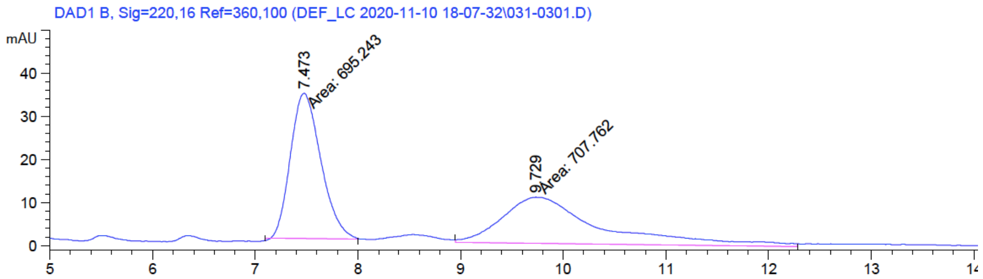

Signal 2: DAD1 B, Sig=220,16 Ref=360,100

Peak #	RetTime [min]	Type	Width [min]	Area [mAU*s]	Height [mAU]	Area %
1	7.473	MM	0.3443	695.24280	33.65096	49.5539
2	9.729	MM	1.1032	707.76178	10.69296	50.4461
Totals :				1403.00458	44.34392	

Supplementary Figure 54. HPLC spectra for compound ***rac*-15i**

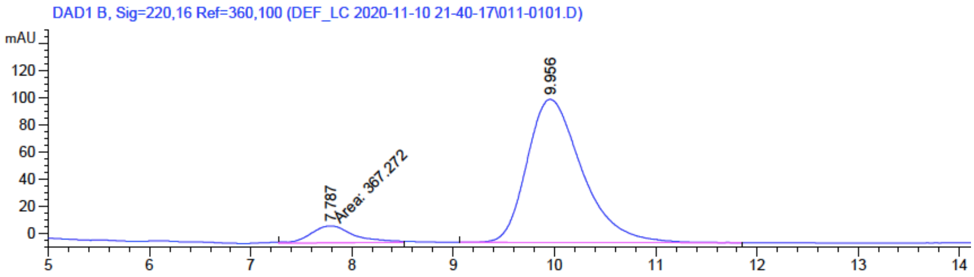

Signal 2: DAD1 B, Sig=220,16 Ref=360,100

Peak #	RetTime [min]	Type	Width [min]	Area [mAU*s]	Height [mAU]	Area %
1	7.787	MM	0.4954	367.27176	12.35485	8.4185
2	9.956	BB	0.5776	3995.39575	105.55721	91.5815
Totals :				4362.66751	117.91206	

Supplementary Figure 55. HPLC spectra for compound **15i**

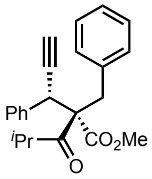

15j

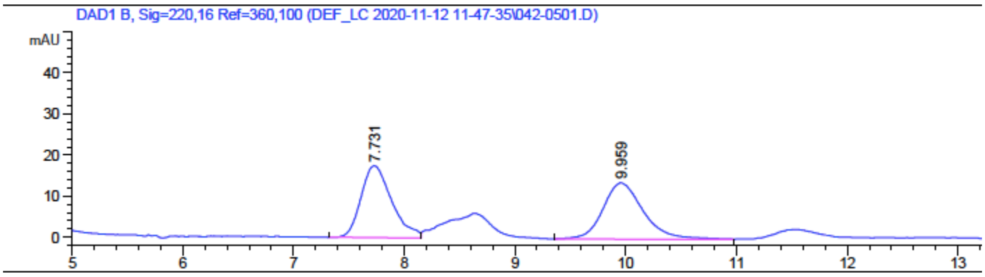

Signal 2: DAD1 B, Sig=220,16 Ref=360,100

Peak #	RetTime [min]	Type	Width [min]	Area [mAU*s]	Height [mAU]	Area %
1	7.731	BV	0.2922	335.23621	17.44101	49.3807
2	9.959	BB	0.3787	343.64511	13.60253	50.6193
Totals :				678.88132	31.04353	

Supplementary Figure 56. HPLC spectra for compound ***rac*-15j**

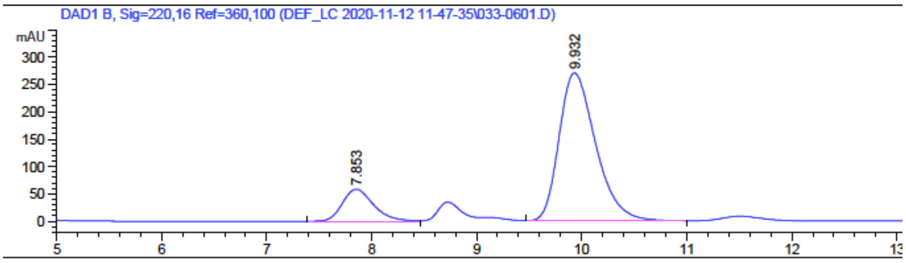

Signal 2: DAD1 B, Sig=220,16 Ref=360,100

Peak #	RetTime [min]	Type	Width [min]	Area [mAU*s]	Height [mAU]	Area %
1	7.853	BV	0.3160	1199.88489	58.31789	15.4330
2	9.932	VV	0.3759	6574.89746	270.20224	84.5670
Totals :				7774.78235	328.52013	

Supplementary Figure 57. HPLC spectra for compound **15j**

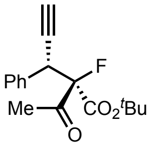

15k

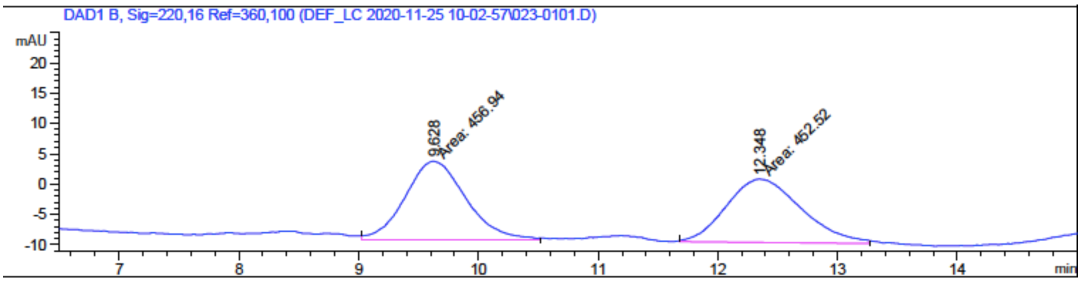

Signal 2: DAD1 B, Sig=220,16 Ref=360,100

Peak #	RetTime [min]	Type	Width [min]	Area [mAU*s]	Height [mAU]	Area %
1	9.628	MM	0.5895	456.93958	12.91800	50.2430
2	12.348	MM	0.7239	452.52029	10.41842	49.7570
Totals :				909.45987	23.33642	

Supplementary Figure 58. HPLC spectra for compound ***rac*-15k**

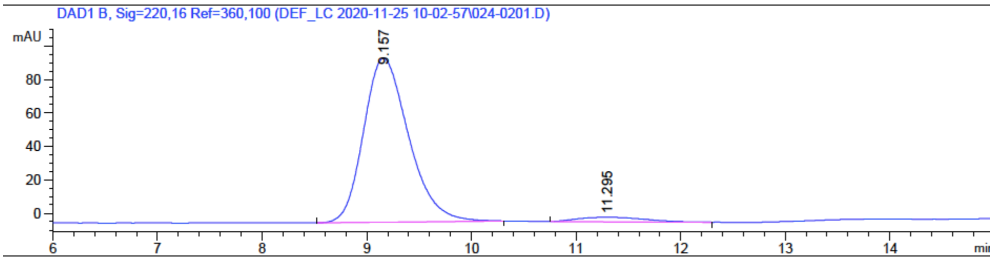

Signal 2: DAD1 B, Sig=220,16 Ref=360,100

Peak #	RetTime [min]	Type	Width [min]	Area [mAU*s]	Height [mAU]	Area %
1	9.157	BB	0.4511	2933.62451	98.03624	96.1463
2	11.295	BB	0.5328	117.58518	2.65425	3.8537
Totals :				3051.20969	100.69049	

Supplementary Figure 59. HPLC spectra for compound **15k**

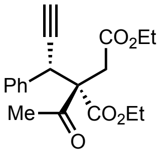

15l

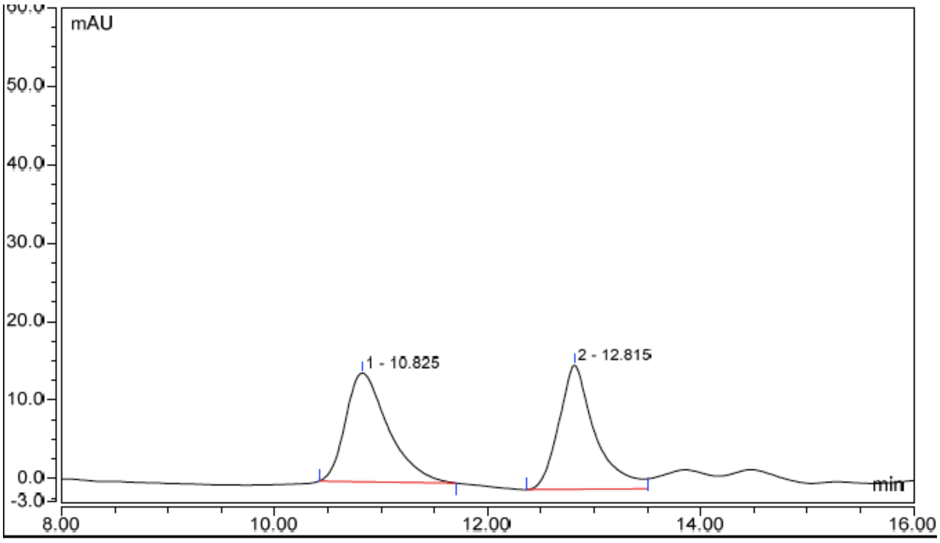

Peak	Ret.Time min	Area mAU*min	Height mAU	Area %	Height %
1	10.825	6.412	13.864	50.59	46.73
2	12.815	6.263	15.805	49.41	53.27
**Sum**	**12.675**	**29.670**	**100.00**	**100.00**	

Supplementary Figure 60. HPLC spectra for compound ***rac*-15l**

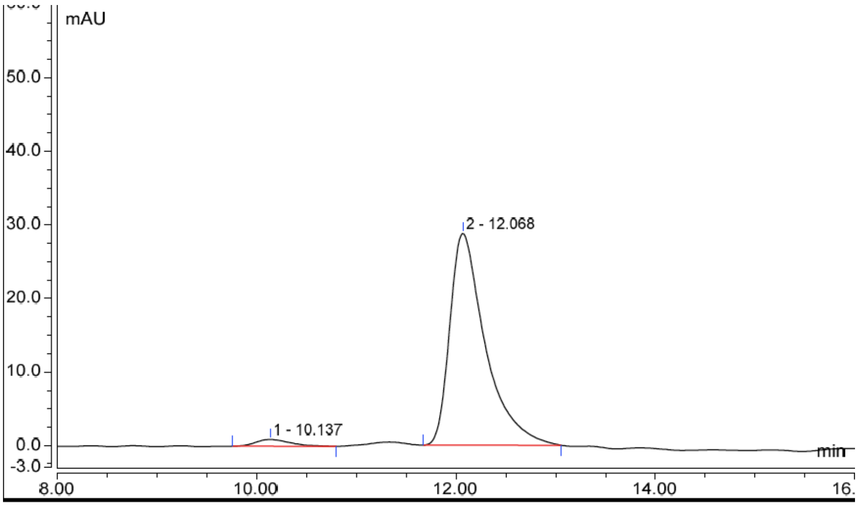

Peak	Ret.Time min	Area mAU*min	Height mAU	Area %	Height %
1	10.137	0.374	0.925	3.06	3.11
2	12.068	11.866	28.804	96.94	96.89
**Sum**	**12.241**	**29.729**	**100.00**	**100.00**	

Supplementary Figure 61. HPLC spectra for compound **15l**

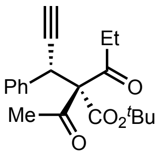

P1
15m

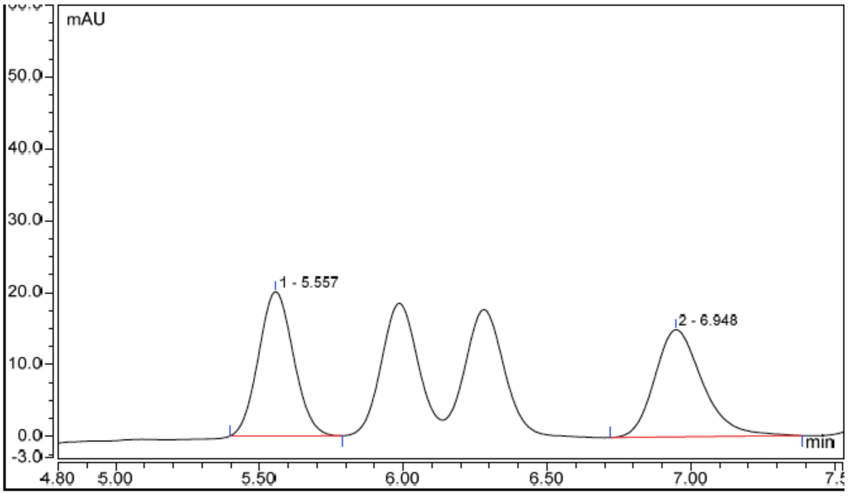

Peak	Ret.Time min	Area mAU*min	Height mAU	Area %	Height %
1	5.557	2.826	20.042	49.57	57.36
2	6.948	2.875	14.899	50.43	42.64
**Sum**	**5.701**	**34.941**	**100.00**	**100.00**	

Supplementary Figure 62. HPLC spectra for compound ***rac*-15m (P1)**

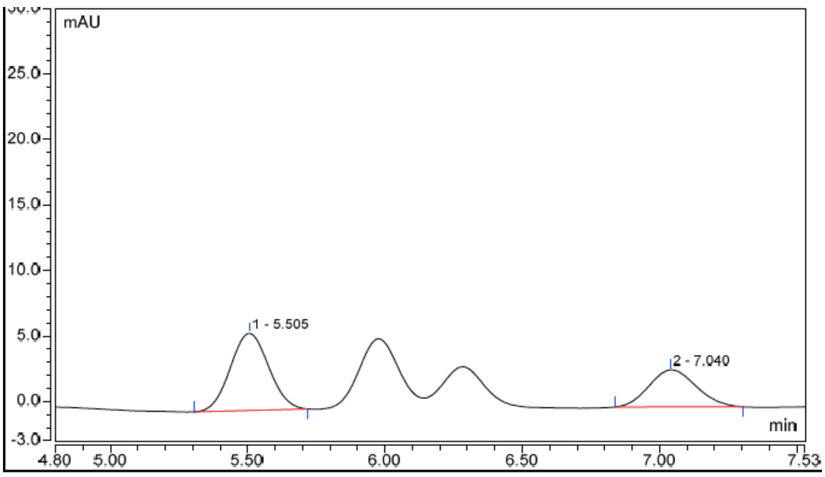

Peak	Ret.Time min	Area mAU*min	Height mAU	Area %	Height %
1	5.505	0.952	5.887	62.97	67.56
2	7.040	0.559	2.826	37.03	32.44
**Sum**	**1.511**	**8.713**	**100.00**	**100.00**	

Supplementary Figure 63. HPLC spectra for compound **15m (P1)**

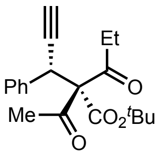

P2
15m

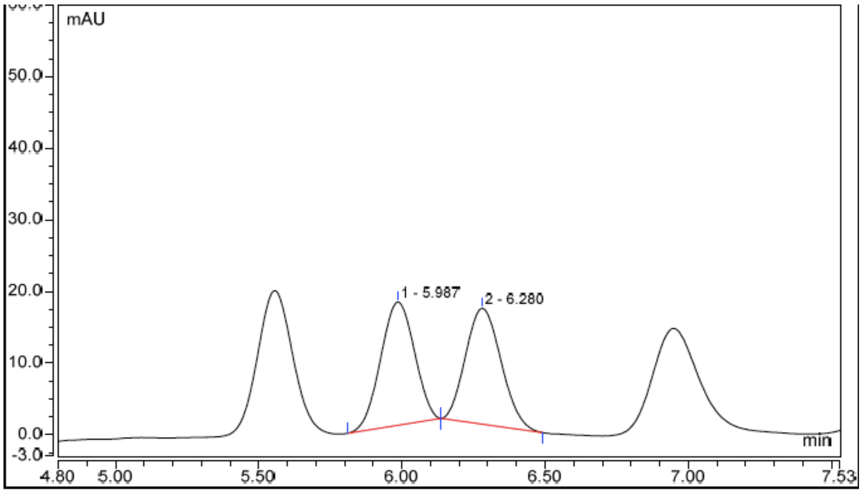

Peak	Ret.Time min	Area mAU*min	Height mAU	Area %	Height %
1	5.987	2.350	17.204	50.24	51.53
2	6.280	2.327	16.179	49.76	48.47
**Sum**	**4.678**	**33.383**	**100.00**	**100.00**	

Supplementary Figure 64. HPLC spectra for compound ***rac*-15m (P2)**

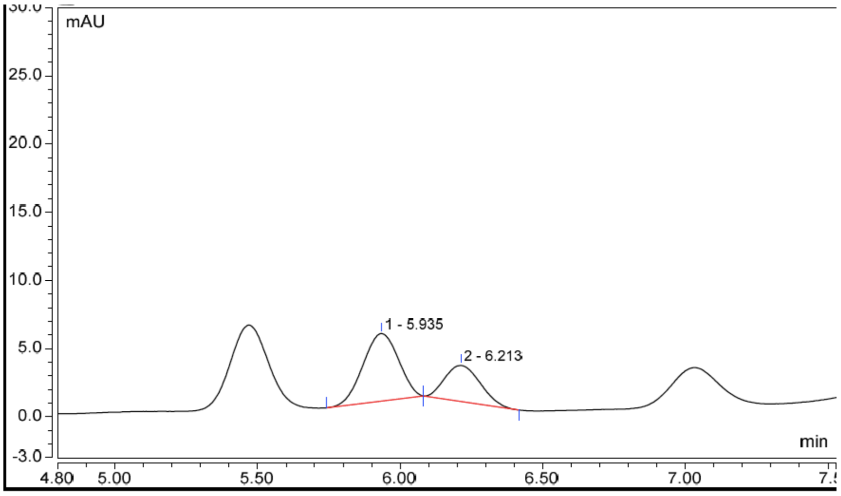

Peak	Ret.Time min	Area mAU*min	Height mAU	Area %	Height %
1	5.935	0.714	4.957	65.15	65.14
2	6.213	0.382	2.653	34.85	34.86
**Sum**	**1.097**	**7.610**	**100.00**	**100.00**	

Supplementary Figure 65. HPLC spectra for compound **15m (P2)**

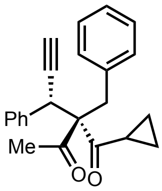

Major-15n

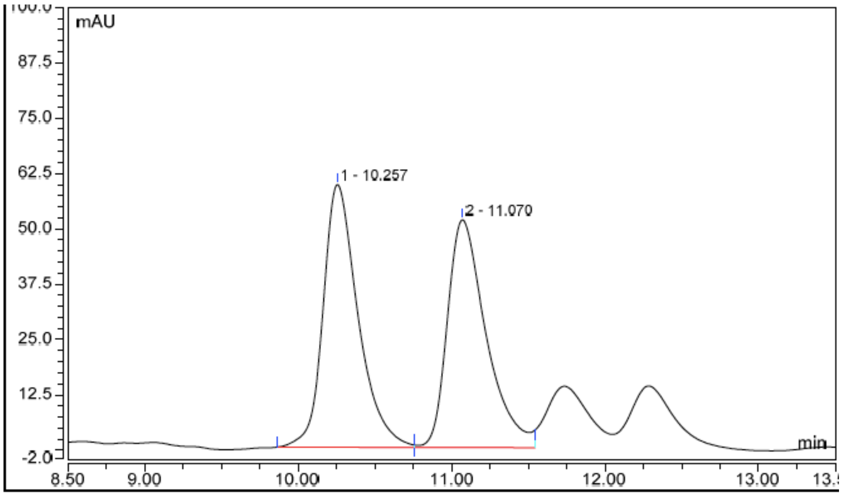

Peak	Ret.Time min	Area mAU*min	Height mAU	Area %	Height %
1	10.257	15.610	59.342	50.96	53.56
2	11.070	15.022	51.456	49.04	46.44
**Sum**	**30.632**	**110.797**	**100.00**	**100.00**	

Supplementary Figure 66. HPLC spectra for compound ***rac*-15n (Major)**

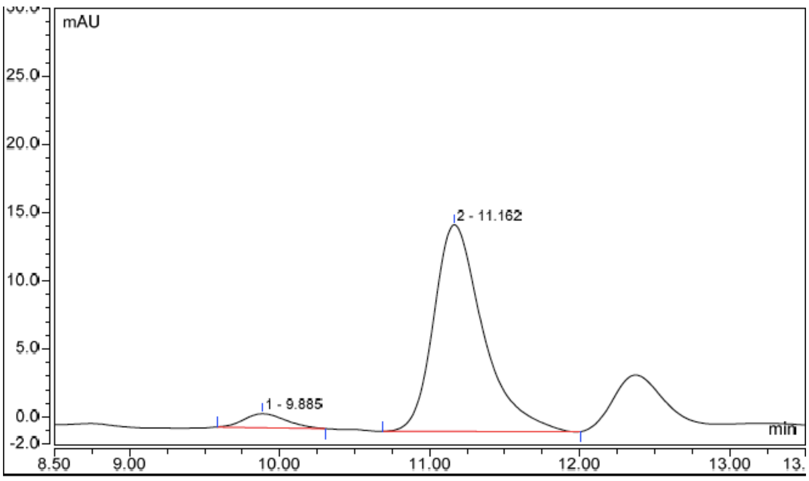

Peak	Ret.Time min	Area mAU*min	Height mAU	Area %	Height %
1	9.885	0.331	1.024	5.41	6.32
2	11.162	5.792	15.173	94.59	93.68
**Sum**	**6.123**	**16.197**	**100.00**	**100.00**	

Supplementary Figure 67. HPLC spectra for compound **15n (Major)**

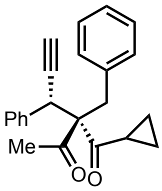

Minor-15n

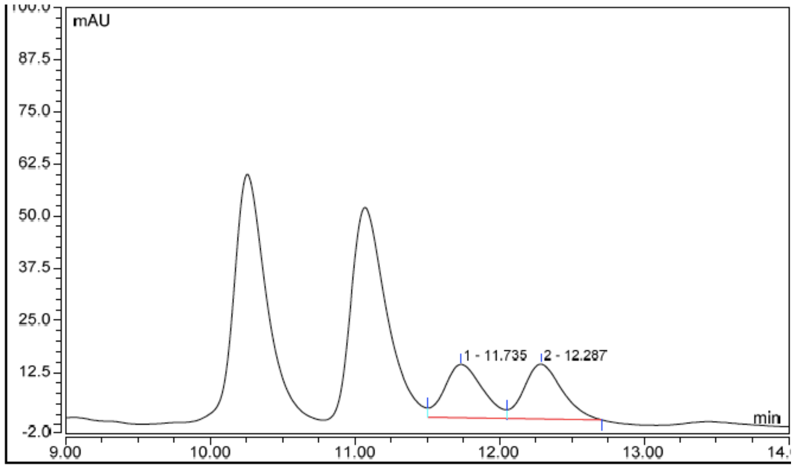

Peak	Ret.Time min	Area mAU*min	Height mAU	Area %	Height %
1	11.735	3.882	12.773	49.36	49.32
2	12.287	3.982	13.127	50.64	50.68
**Sum**	**7.864**	**25.899**	**100.00**	**100.00**	

Supplementary Figure 68. HPLC spectra for compound ***rac*-15n (Minor)**

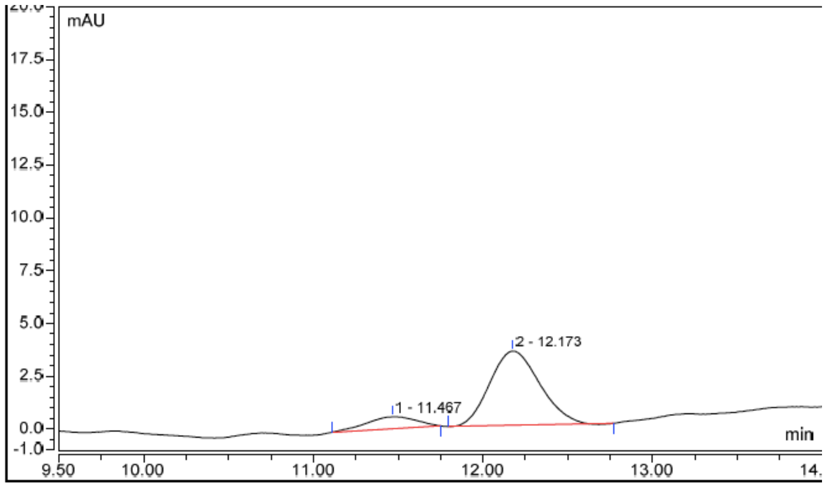

Peak	Ret.Time min	Area mAU*min	Height mAU	Area %	Height %
1	11.467	0.193	0.568	13.95	13.93
2	12.173	1.190	3.511	86.05	86.07
**Sum**	**1.383**	**4.079**	**100.00**	**100.00**	

Supplementary Figure 69. HPLC spectra for compound **15n (Minor)**

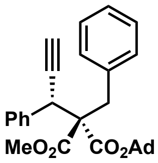

15o

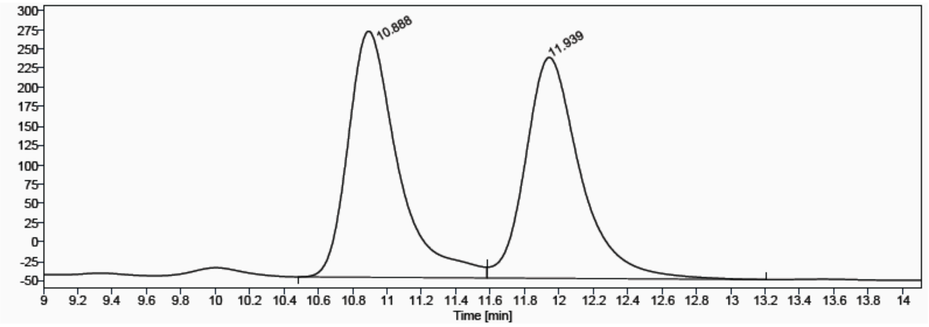

Signal: DAD1C, Sig=210, 4 Ref=off

RT [min]	Type	Width [min]	Area	Height	Area%
10.9	BV	1.1	6416.8	319	50.2
11.9	VB	1.6	6361.3	286	49.8
		Sum	12778.0		100.0

Supplementary Figure 70. HPLC spectra for compound ***rac*-15o**

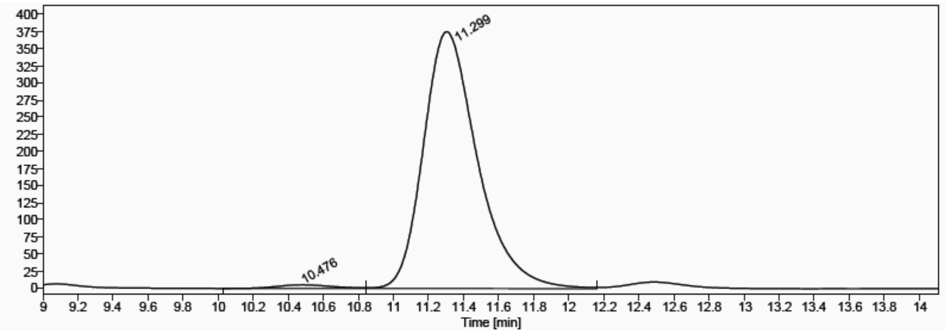

Signal: DAD1C, Sig=210, 4 Ref=off

RT [min]	Type	Width [min]	Area	Height	Area%
10.5	BV	0.8	118.9	5	1.5
11.3	VV	1.3	8082.2	376	98.5
		Sum	8201.1		100.0

Supplementary Figure 71. HPLC spectra for compound **15o**

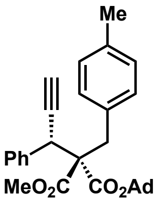

15p

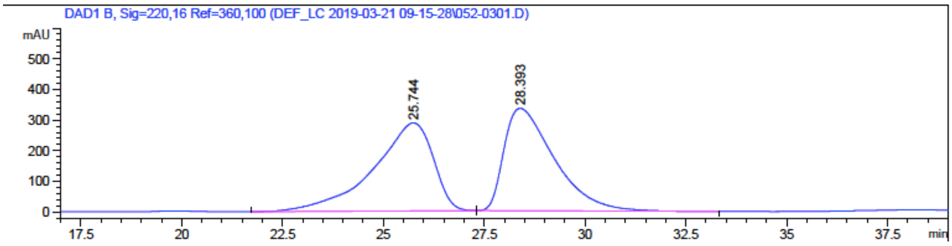

Signal 2: DAD1 B, Sig=220,16 Ref=360,100

Peak #	RetTime [min]	Type	Width [min]	Area [mAU*s]	Height [mAU]	Area %
1	25.744	BB	1.4881	2.95847e4	287.12564	50.4849
2	28.393	BB	1.2913	2.90163e4	334.07986	49.5151
Totals :				5.86010e4	621.20551	

Supplementary Figure 72. HPLC spectra for compound ***rac*-15p**

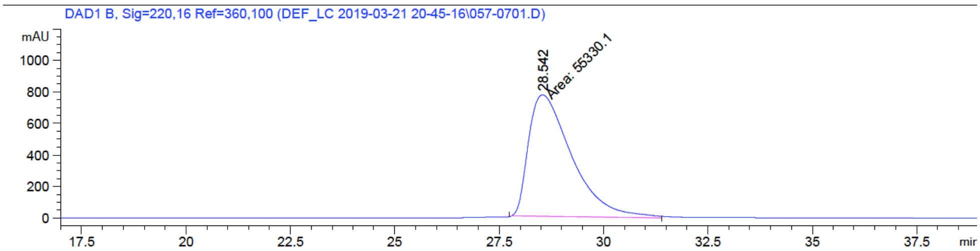

Signal 2: DAD1 B, Sig=220,16 Ref=360,100

Peak #	RetTime [min]	Type	Width [min]	Area [mAU*s]	Height [mAU]	Area %
1	28.542	MM	1.1973	5.53301e4	770.23334	100.0000
Totals :				5.53301e4	770.23334	

Supplementary Figure 73. HPLC spectra for compound **15p**

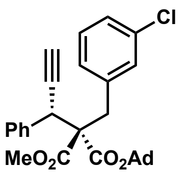

15q

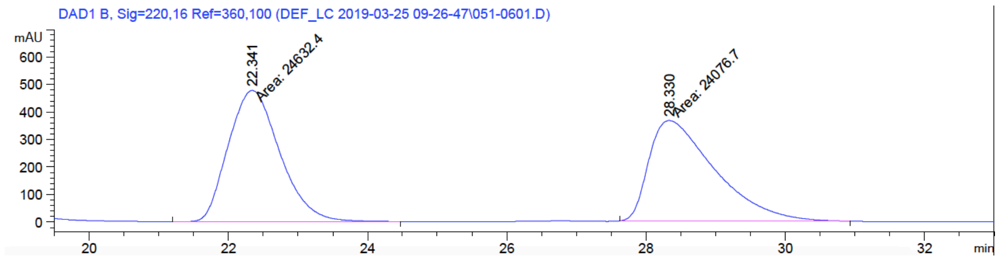

Signal 2: DAD1 B, Sig=220,16 Ref=360,100

Peak #	RetTime [min]	Type	Width [min]	Area [mAU*s]	Height [mAU]	Area %
1	22.341	MM	0.8593	2.46324e4	477.74161	50.5705
2	28.330	MM	1.0973	2.40767e4	365.68240	49.4295
Totals :				4.87091e4	843.42401	

Supplementary Figure 74. HPLC spectra for compound ***rac*-15q**

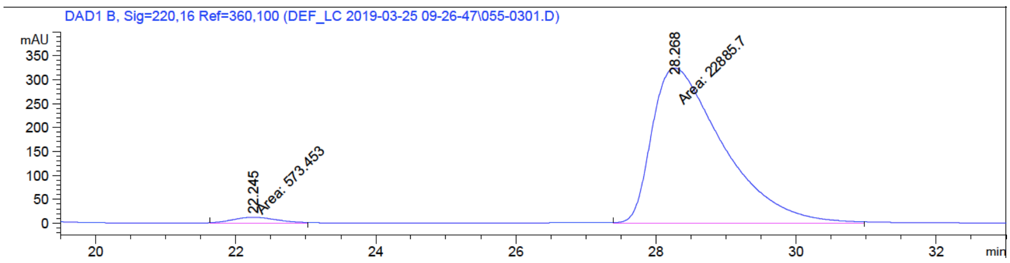

Signal 2: DAD1 B, Sig=220,16 Ref=360,100

Peak #	RetTime [min]	Type	Width [min]	Area [mAU*s]	Height [mAU]	Area %
1	22.245	MM	0.7608	573.45300	12.56320	2.4445
2	28.268	MM	1.1767	2.28857e4	324.14478	97.5555
Totals :				2.34591e4	336.70798	

Supplementary Figure 75. HPLC spectra for compound **15q**

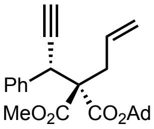

15r

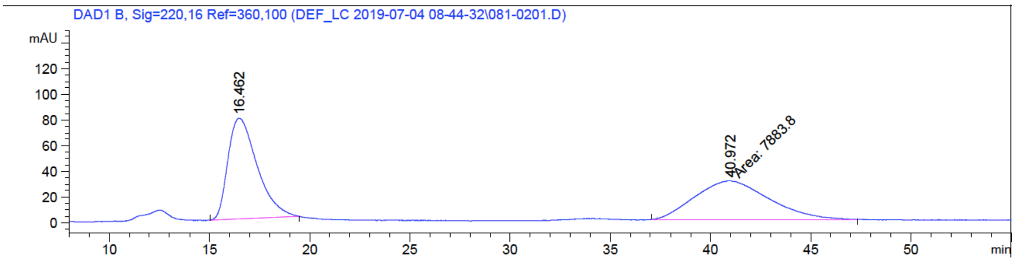


```
Signal 2: DAD1 B, Sig=220,16 Ref=360,100

Peak RetTime Type  Width     Area      Height     Area
  #   [min]         [min]   [mAU*s]     [mAU]      %
----|-------|----|-------|----------|----------|--------|
   1  16.462 BB    1.1916 7969.41553   78.42348  50.2700
   2  40.972 MM    4.3253 7883.79980   30.37896  49.7300

Totals :                  1.58532e4  108.80244
```

Supplementary Figure 76. HPLC spectra for compound ***rac*-15r**

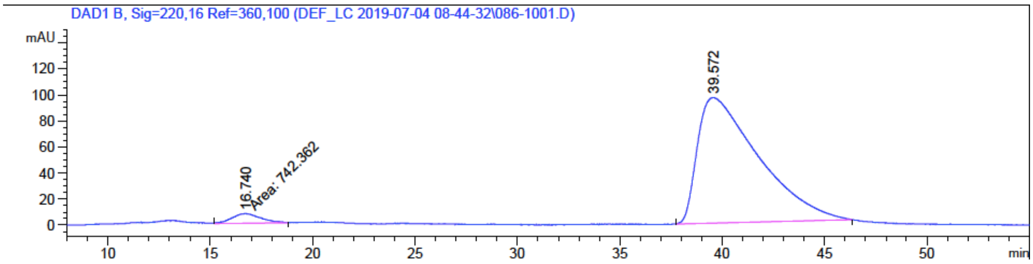


```
Signal 2: DAD1 B, Sig=220,16 Ref=360,100

Peak RetTime Type  Width     Area      Height     Area
  #   [min]         [min]   [mAU*s]     [mAU]      %
----|-------|----|-------|----------|----------|--------|
   1  16.740 MM    1.6626  742.36230    7.44167   3.5798
   2  39.572 BB    2.4340 1.99951e4   96.39645  96.4202

Totals :                  2.07374e4  103.83811
```

Supplementary Figure 77. HPLC spectra for compound **15r**

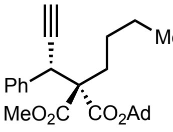

15s

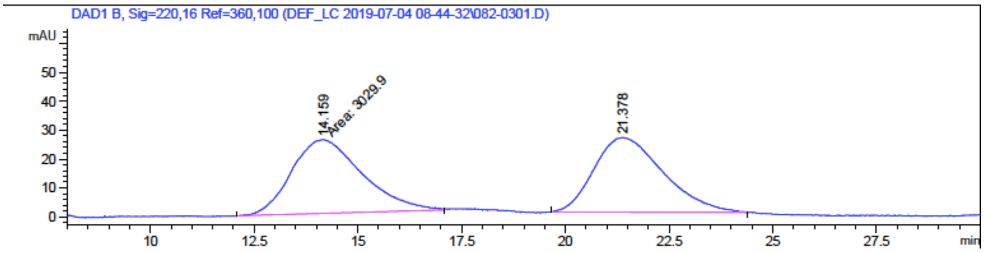

Signal 2: DAD1 B, Sig=220,16 Ref=360,100

Peak #	RetTime [min]	Type	Width [min]	Area [mAU*s]	Height [mAU]	Area %
1	14.159	MM	1.9779	3029.90454	25.53188	50.3015
2	21.378	BB	1.3715	2993.57715	25.76619	49.6985

Totals : 6023.48169 51.29807

Supplementary Figure 78. HPLC spectra for compound ***rac*-15s**

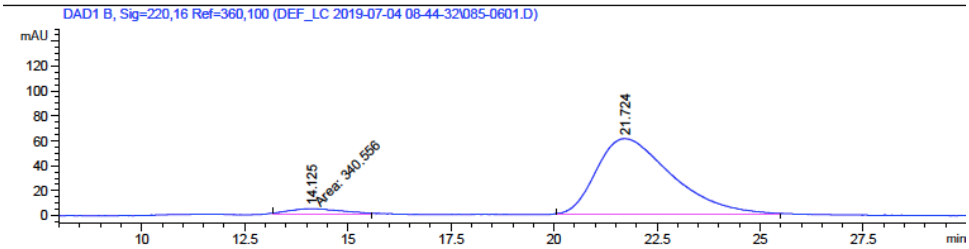

Signal 2: DAD1 B, Sig=220,16 Ref=360,100

Peak #	RetTime [min]	Type	Width [min]	Area [mAU*s]	Height [mAU]	Area %
1	14.125	MM	1.4140	340.55637	4.01409	4.2936
2	21.724	BB	1.4780	7591.10938	60.41209	95.7064

Totals : 7931.66574 64.42618

Supplementary Figure 79. HPLC spectra for compound **15s**

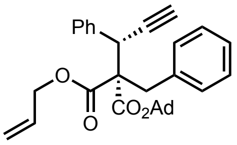

15t

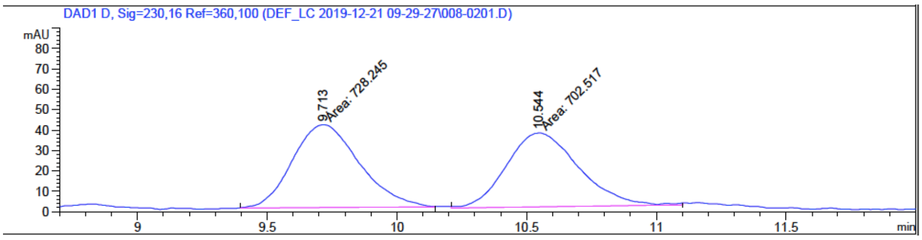

Signal 4: DAD1 D, Sig=230,16 Ref=360,100

Peak #	RetTime [min]	Type	Width [min]	Area [mAU*s]	Height [mAU]	Area %
1	9.713	MM	0.2996	728.24469	40.51402	50.8991
2	10.544	MM	0.3240	702.51746	36.13996	49.1009
Totals :				1430.76215	76.65397	

Supplementary Figure 80. HPLC spectra for compound ***rac*-15t**

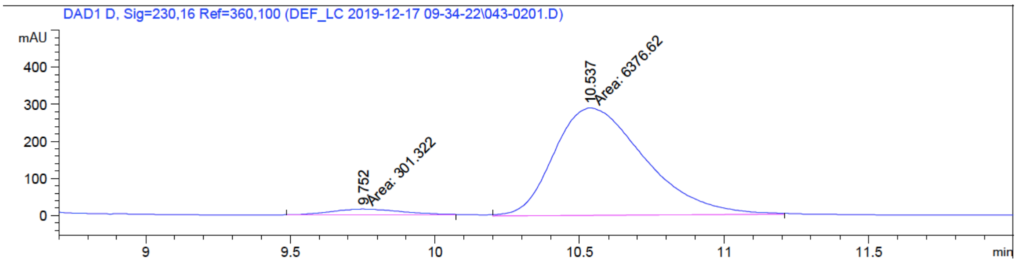

Signal 4: DAD1 D, Sig=230,16 Ref=360,100

Peak #	RetTime [min]	Type	Width [min]	Area [mAU*s]	Height [mAU]	Area %
1	9.752	MM	0.3065	301.32230	16.38713	4.5122
2	10.537	MM	0.3662	6376.61719	290.19232	95.4878
Totals :				6677.93948	306.57946	

Supplementary Figure 81. HPLC spectra for compound **15t**

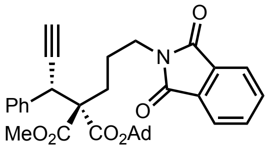

15u

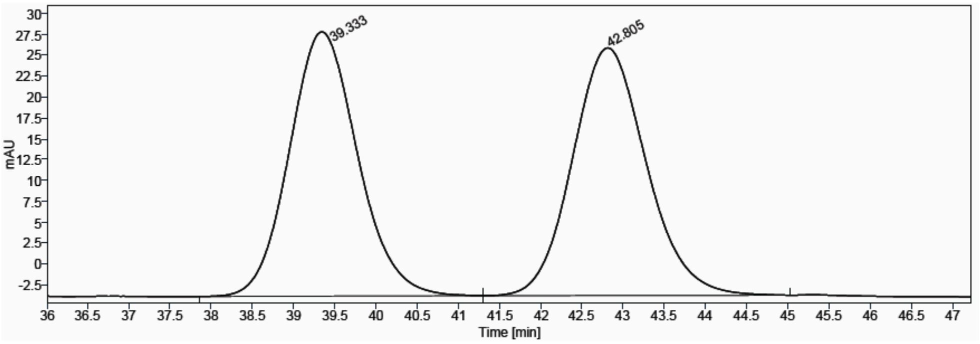

Signal: DAD1D, Sig=230, 4 Ref=off

RT [min]	Type	Width [min]	Area	Height	Area%
39.3	MM m	0.9	1825.6	32	49.6
42.8	BB	3.7	1851.5	30	50.4
		Sum	3677.0		100.0

Supplementary Figure 82. HPLC spectra for compound ***rac*-15u**

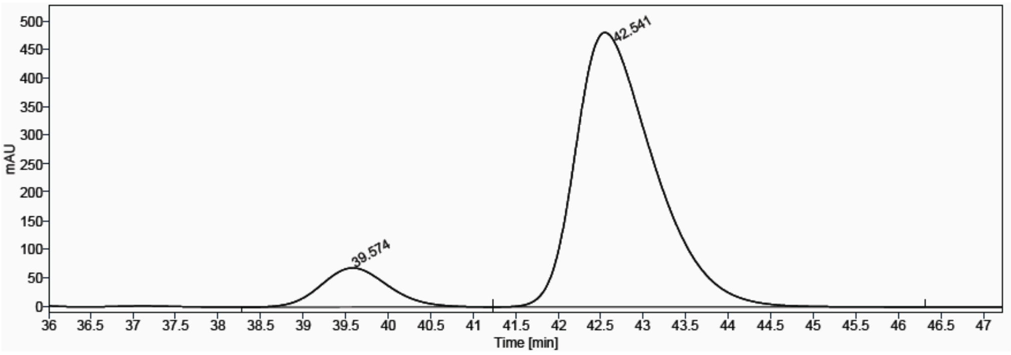

Signal: DAD1D, Sig=230, 4 Ref=off

RT [min]	Type	Width [min]	Area	Height	Area%
39.6	BB	3.0	3786.1	68	10.9
42.5	BM m	1.0	30951.9	481	89.1
		Sum	34738.0		100.0

Supplementary Figure 83. HPLC spectra for compound **15u**

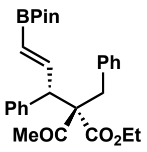

16a

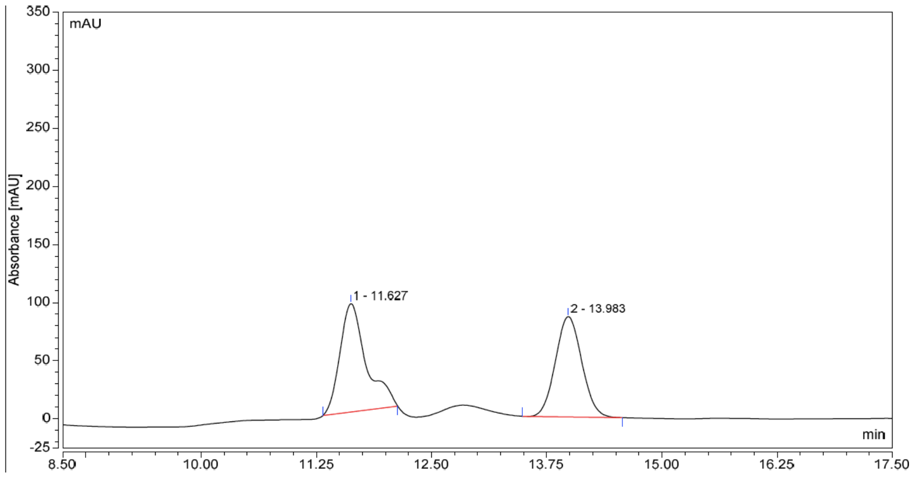

Peak	RetTime min	Area mAU*min	Height mAU	Area %	Height %
1	11.627	30.887	93.180	51.88	51.88
2	13.983	28.647	86.433	48.12	48.12
**Total**		**59.533**	**179.614**	**100.00**	**100.00**

Supplementary Figure 84. HPLC spectra for compound ***rac*-16a**

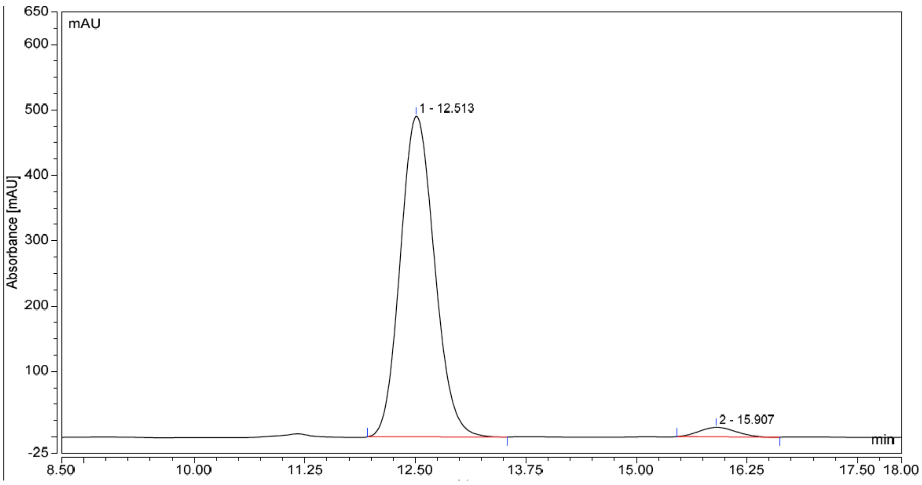

Peak	RetTime min	Area mAU*min	Height mAU	Area %	Height %
1	12.513	219.812	490.619	96.95	97.14
2	15.907	6.916	14.446	3.05	2.86
**Total**		**226.728**	**505.065**	**100.00**	**100.00**

Supplementary Figure 85. HPLC spectra for compound **16a**

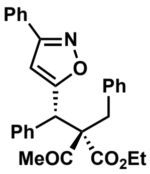

16b

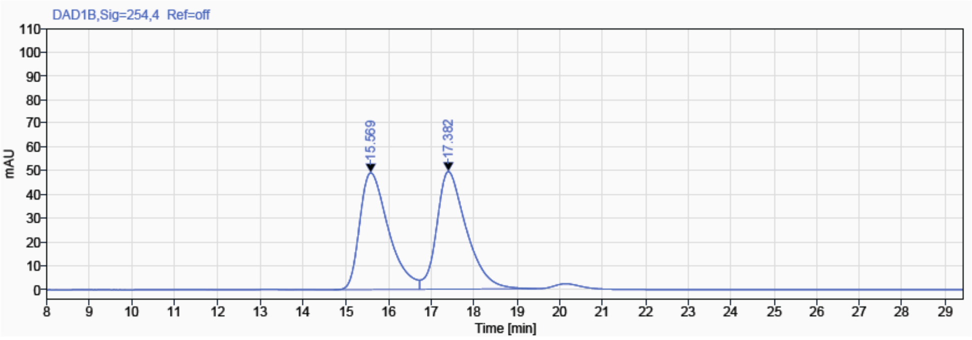

Signal: DAD1B,Sig=254,4 Ref=off

RT [min]	Type	Width [min]	Area	Height	Area%	Name
15.569	BV	2.00	2301.99	49.20	49.03	
17.382	VB	2.73	2392.85	49.59	50.97	
		Sum	4694.84			

Supplementary Figure 86. HPLC spectra for compound ***rac*-16b**

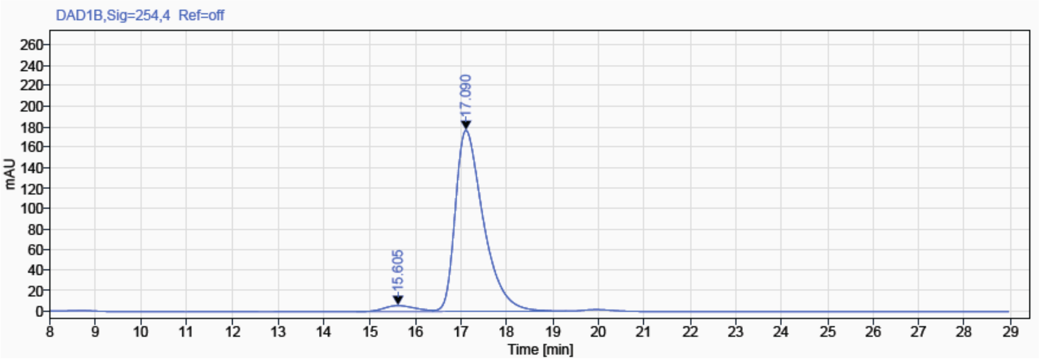

Signal: DAD1B,Sig=254,4 Ref=off

RT [min]	Type	Width [min]	Area	Height	Area%	Name
15.605	BV	1.68	275.04	5.79	3.52	
17.090	VB	2.91	7530.89	176.78	96.48	
		Sum	7805.93			

Supplementary Figure 87. HPLC spectra for compound **16b**

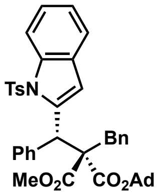

16c

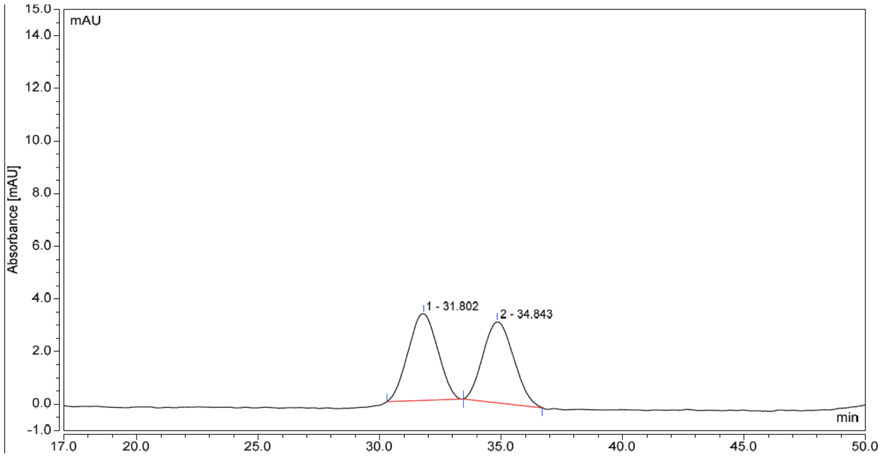

Peak	RetTime<br>min	Area<br>mAU*min	Height<br>mAU	Area<br>%	Height<br>%
1	31.802	4.644	3.293	50.74	51.68
2	34.843	4.510	3.079	49.26	48.32
**Total**		**9.154**	**6.373**	**100.00**	**100.00**

Supplementary Figure 88. HPLC spectra for compound ***rac*-16c**

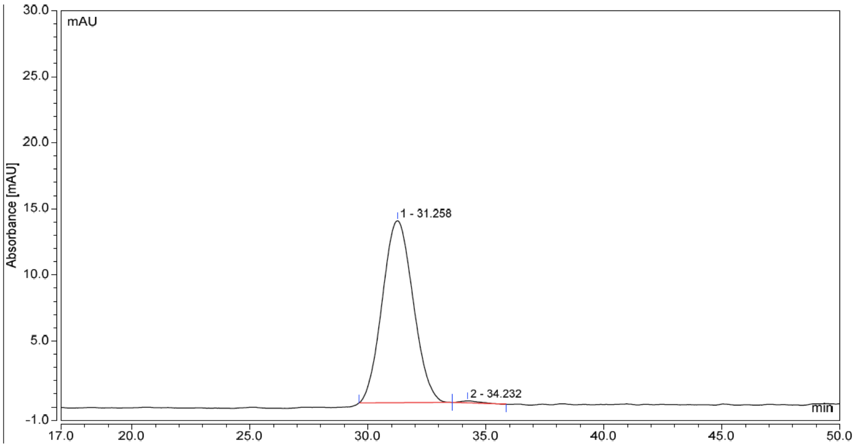

Peak	RetTime<br>min	Area<br>mAU*min	Height<br>mAU	Area<br>%	Height<br>%
1	31.258	20.854	13.769	99.37	98.91
2	34.232	0.133	0.152	0.63	1.09
**Total**		**20.987**	**13.921**	**100.00**	**100.00**

Supplementary Figure 89. HPLC spectra for compound **16c**

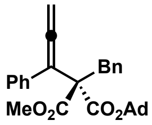

16d

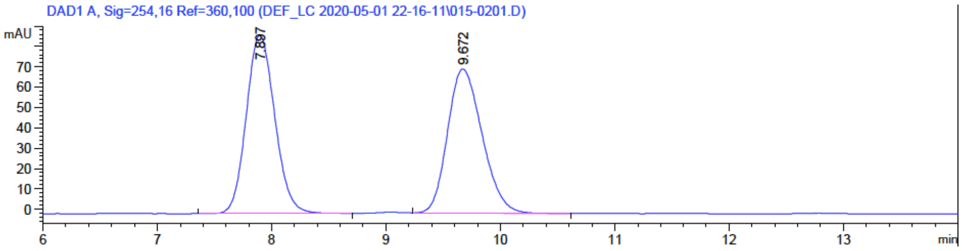

Signal 1: DAD1 A, Sig=254,16 Ref=360,100

Peak #	RetTime [min]	Type	Width [min]	Area [mAU*s]	Height [mAU]	Area %
1	7.897	BB	0.2699	1519.42004	87.81509	50.5295
2	9.672	BB	0.3270	1487.57568	70.85131	49.4705

Totals : 3006.99573 158.66640

Supplementary Figure 90. HPLC spectra for compound ***rac*-16d**

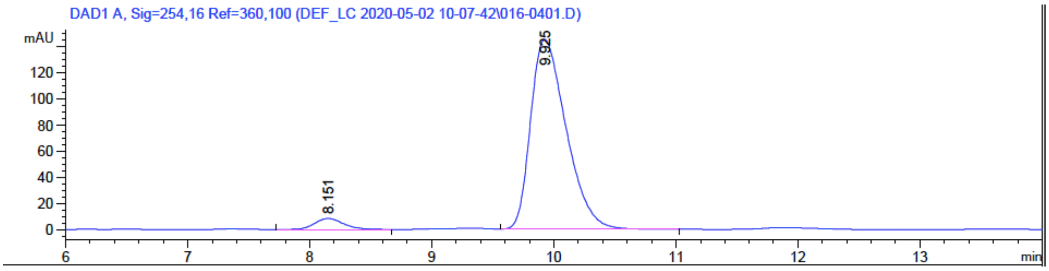

Signal 1: DAD1 A, Sig=254,16 Ref=360,100

Peak #	RetTime [min]	Type	Width [min]	Area [mAU*s]	Height [mAU]	Area %
1	8.151	BB	0.2522	138.87616	8.43143	4.5197
2	9.925	BB	0.3114	2933.80688	145.40533	95.4803

Totals : 3072.68304 153.83676

Supplementary Figure 91. HPLC spectra for compound **16d**

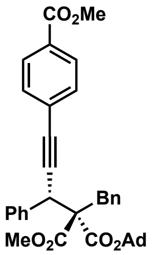

16e

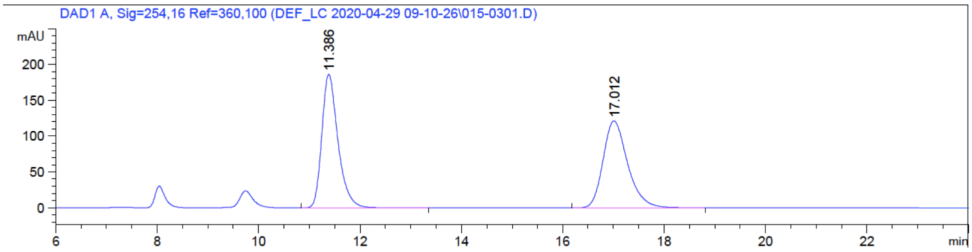

Signal 1: DAD1 A, Sig=254,16 Ref=360,100

Peak #	RetTime [min]	Type	Width [min]	Area [mAU*s]	Height [mAU]	Area %
1	11.386	BB	0.3344	4124.68506	186.24055	50.2619
2	17.012	BB	0.5147	4081.69312	121.25317	49.7381

Totals : 8206.37817 307.49372

Supplementary Figure 92. HPLC spectra for compound ***rac*-16e**

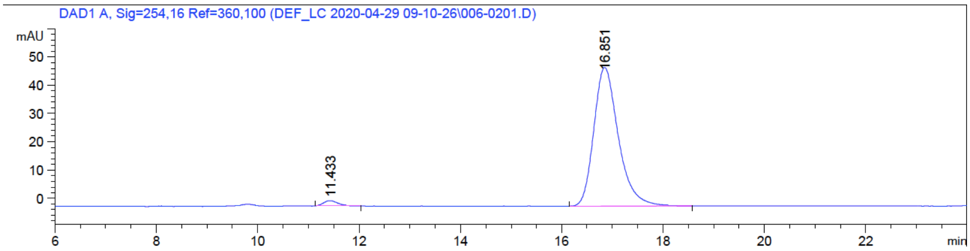

Signal 1: DAD1 A, Sig=254,16 Ref=360,100

Peak #	RetTime [min]	Type	Width [min]	Area [mAU*s]	Height [mAU]	Area %
1	11.433	BB	0.2596	38.62483	1.88083	2.2821
2	16.851	BB	0.5110	1653.90125	49.09585	97.7179

Totals : 1692.52607 50.97668

Supplementary Figure 93. HPLC spectra for compound **16e**

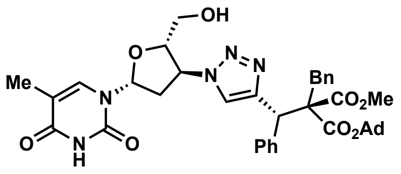

16f

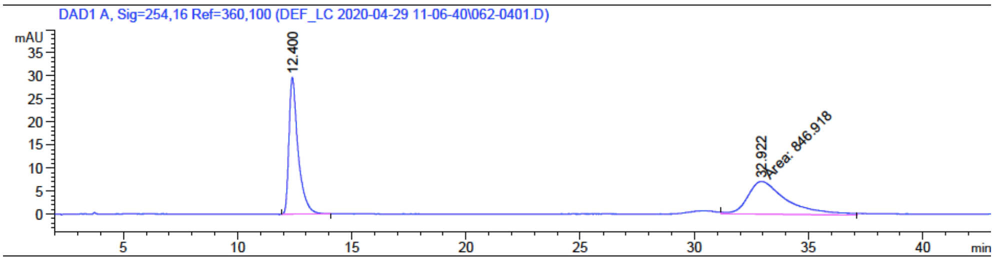

Signal 1: DAD1 A, Sig=254,16 Ref=360,100

Peak #	RetTime [min]	Type	Width [min]	Area [mAU*s]	Height [mAU]	Area %
1	12.400	BB	0.4079	823.25305	29.65197	49.2915
2	32.922	MM	1.9902	846.91840	7.09247	50.7085
Totals :				1670.17145	36.74444	

Supplementary Figure 94. HPLC spectra for compound ***rac*-16f**

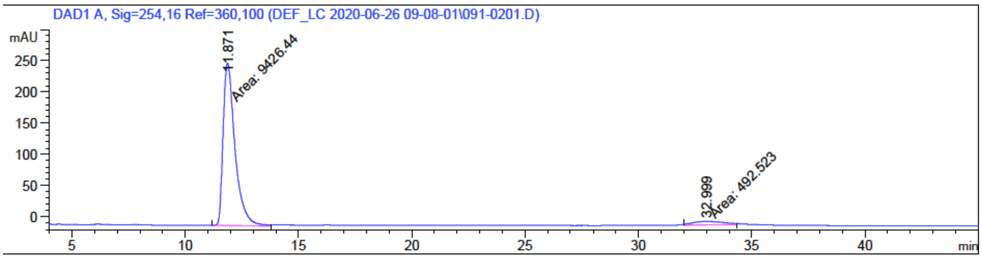

Signal 1: DAD1 A, Sig=254,16 Ref=360,100

Peak #	RetTime [min]	Type	Width [min]	Area [mAU*s]	Height [mAU]	Area %
1	11.871	MM	0.6057	9426.43555	259.37070	95.0345
2	32.999	MM	1.0988	492.52322	5.30670	4.9655
Totals :				9918.95877	264.67740	

Supplementary Figure 95. HPLC spectra for compound **16f**

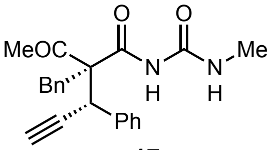

17

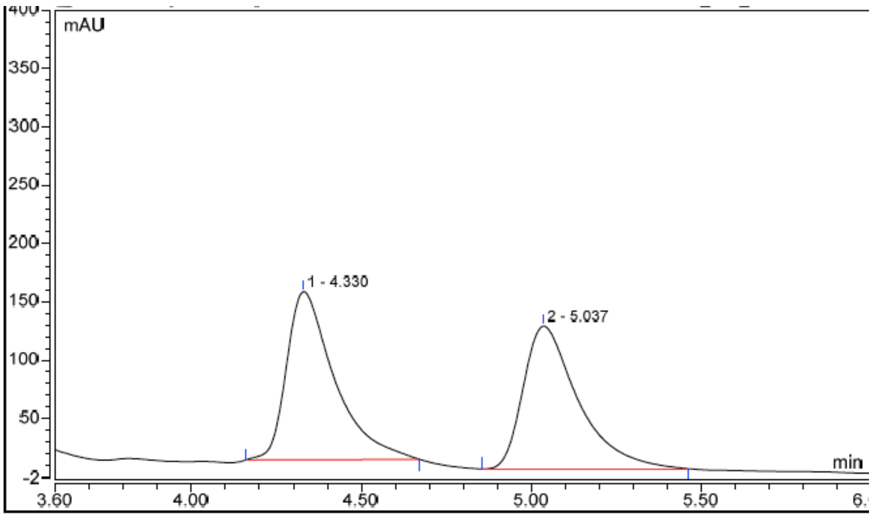

Peak	Ret.Time min	Area mAU*min	Height mAU	Area %	Height %
1	4.330	23.652	144.234	50.80	54.09
2	5.037	22.910	122.419	49.20	45.91
**Sum**	**46.562**	**266.653**	**100.00**	**100.00**	

Supplementary Figure 96. HPLC spectra for compound ***rac*-17**

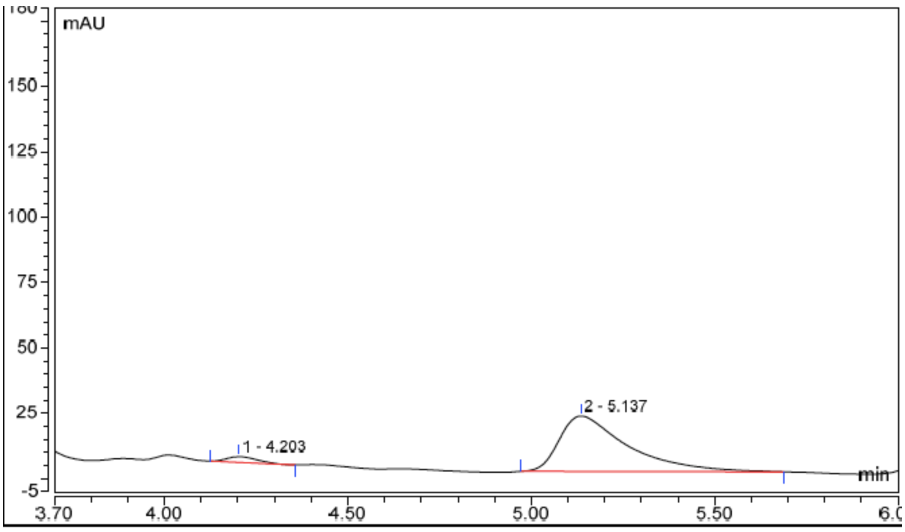

Peak	Ret.Time min	Area mAU*min	Height mAU	Area %	Height %
1	4.203	0.213	2.196	4.45	9.39
2	5.137	4.573	21.195	95.55	90.61
**Sum**	**4.786**	**23.391**	**100.00**	**100.00**	

Supplementary Figure 97. HPLC spectra for compound **17**

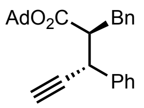

18

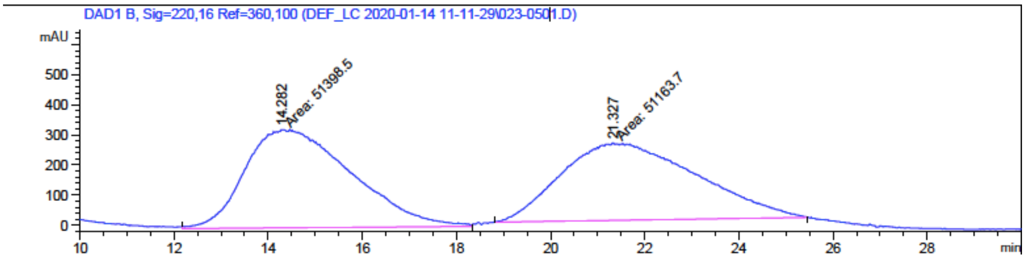

Signal 2: DAD1 B, Sig=220,16 Ref=360,100

Peak #	RetTime [min]	Type	Width [min]	Area [mAU*s]	Height [mAU]	Area %
1	14.282	MM	2.6280	5.13985e4	325.96536	50.1145
2	21.327	MM	3.3300	5.11637e4	256.07095	49.8855
Totals :				1.02562e5	582.03632	

Supplementary Figure 98. HPLC spectra for compound ***rac*-18**

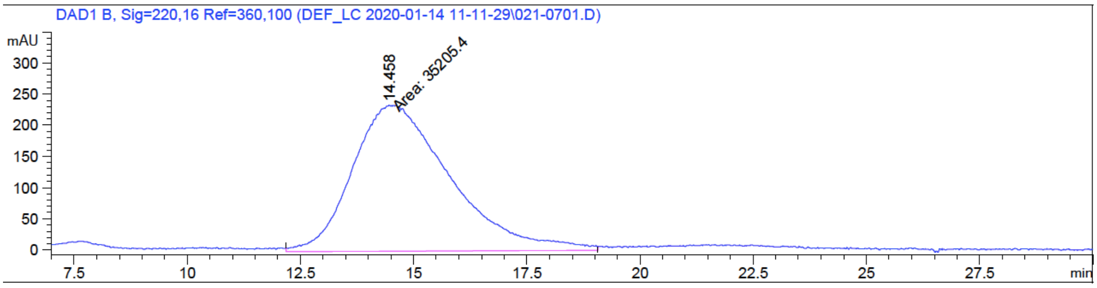

Signal 2: DAD1 B, Sig=220,16 Ref=360,100

Peak #	RetTime [min]	Type	Width [min]	Area [mAU*s]	Height [mAU]	Area %
1	14.458	MM	2.5070	3.52054e4	234.05153	100.0000
Totals :				3.52054e4	234.05153	

Supplementary Figure 99. HPLC spectra for compound **18**

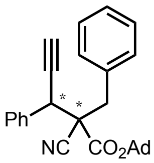

Major-24

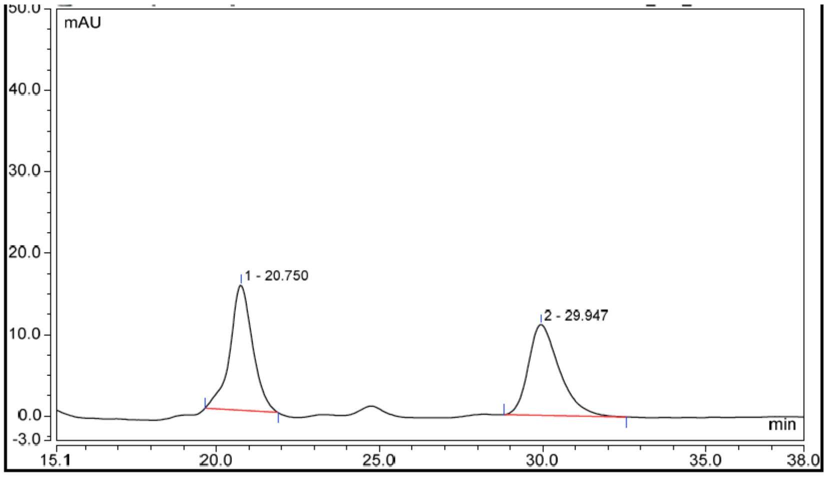

Peak	Ret.Time min	Area mAU*min	Height mAU	Area %	Height %
1	20.750	12.355	15.376	50.18	57.99
2	29.947	12.264	11.140	49.82	42.01
**Sum**	**24.619**	**26.516**	**100.00**	**100.00**	

Supplementary Figure 100. HPLC spectra for compound ***rac*-24 (Major)**

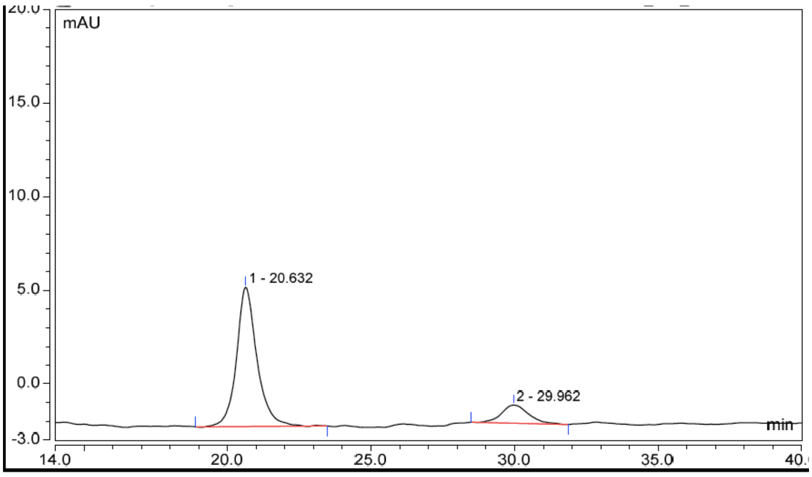

Peak	Ret.Time min	Area mAU*min	Height mAU	Area %	Height %
1	20.632	6.167	7.447	85.23	88.42
2	29.962	1.069	0.976	14.77	11.58
**Sum**	**7.236**	**8.423**	**100.00**	**100.00**	

Supplementary Figure 101. HPLC spectra for compound **24 (Major)**

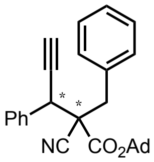

Minor-24

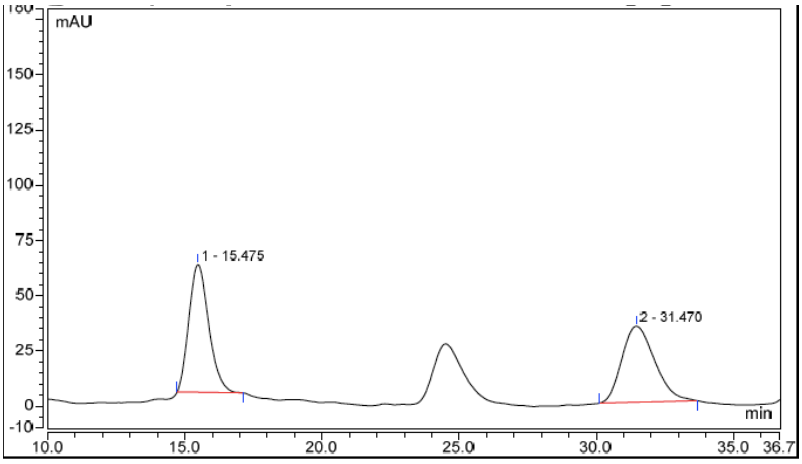

Peak	Ret.Time min	Area mAU*min	Height mAU	Area %	Height %
1	15.475	48.497	57.824	50.39	62.69
2	31.470	47.743	34.420	49.61	37.31
**Sum**	**96.239**	**92.244**	**100.00**	**100.00**	

Supplementary Figure 102. HPLC spectra for compound ***rac*-24 (Minor)**

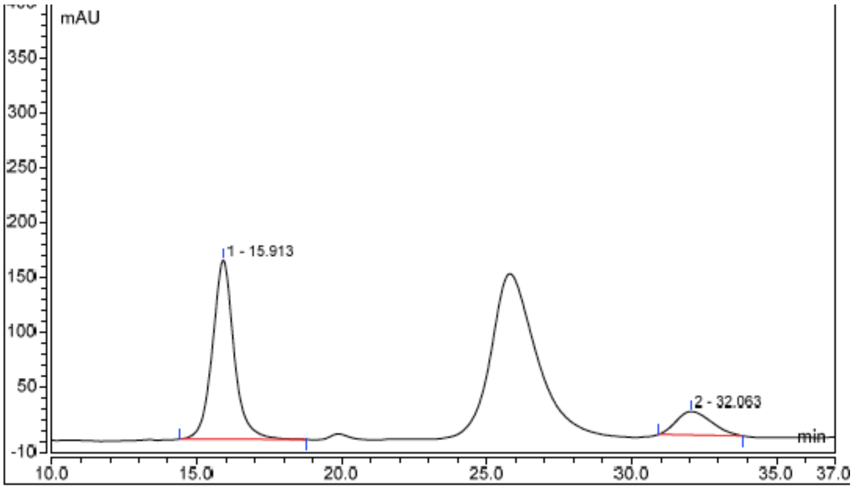

Peak	Ret.Time min	Area mAU*min	Height mAU	Area %	Height %
1	15.913	141.630	163.438	82.48	88.40
2	32.063	30.092	21.437	17.52	11.60
**Sum**	**171.722**	**184.875**	**100.00**	**100.00**	

Supplementary Figure 103. HPLC spectra for compound **24 (Minor)**

NMR Spectra

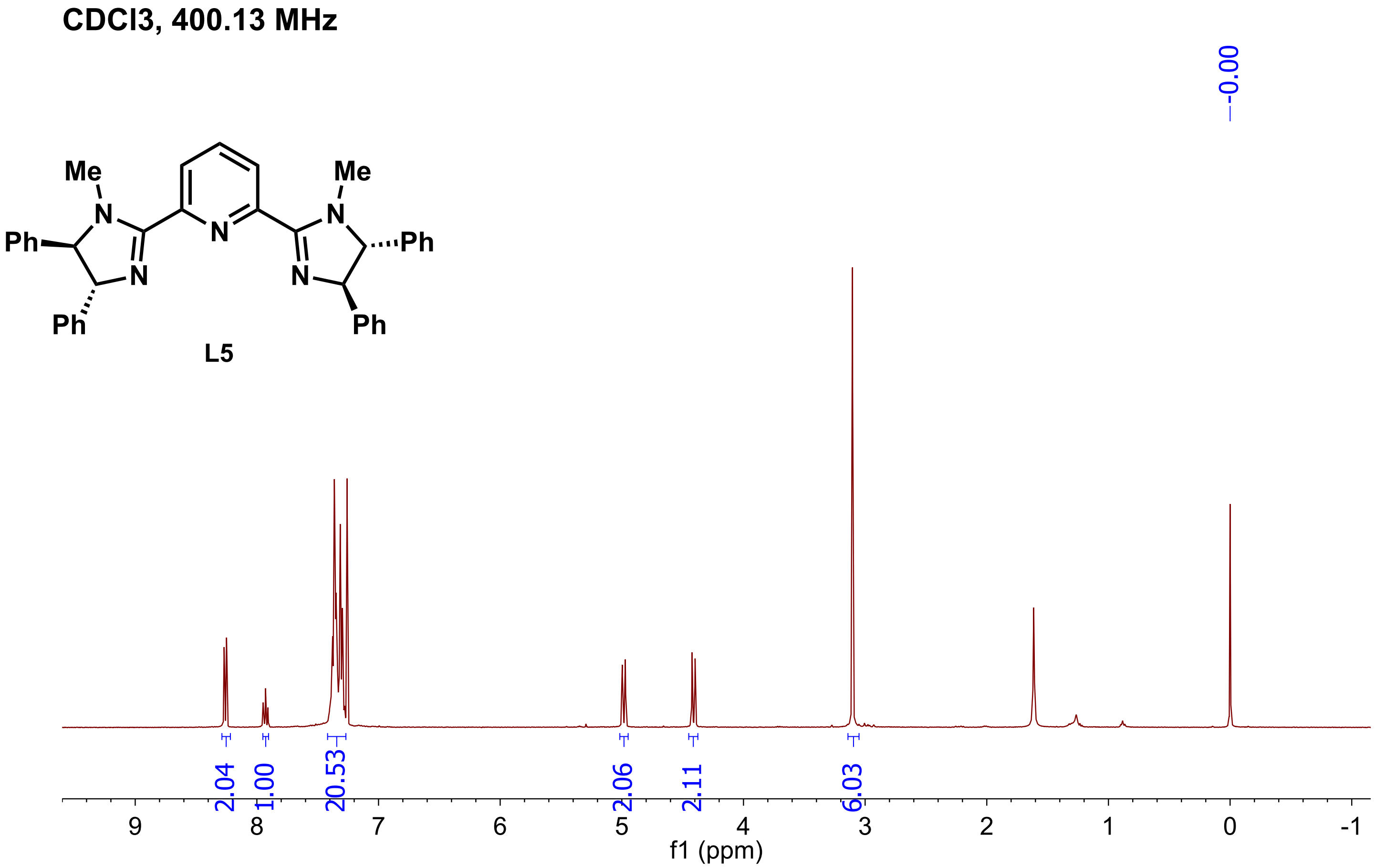

Supplementary Figure 104. ^{1}H NMR (400 MHz, $CDCl_3$) spectra for **L5**

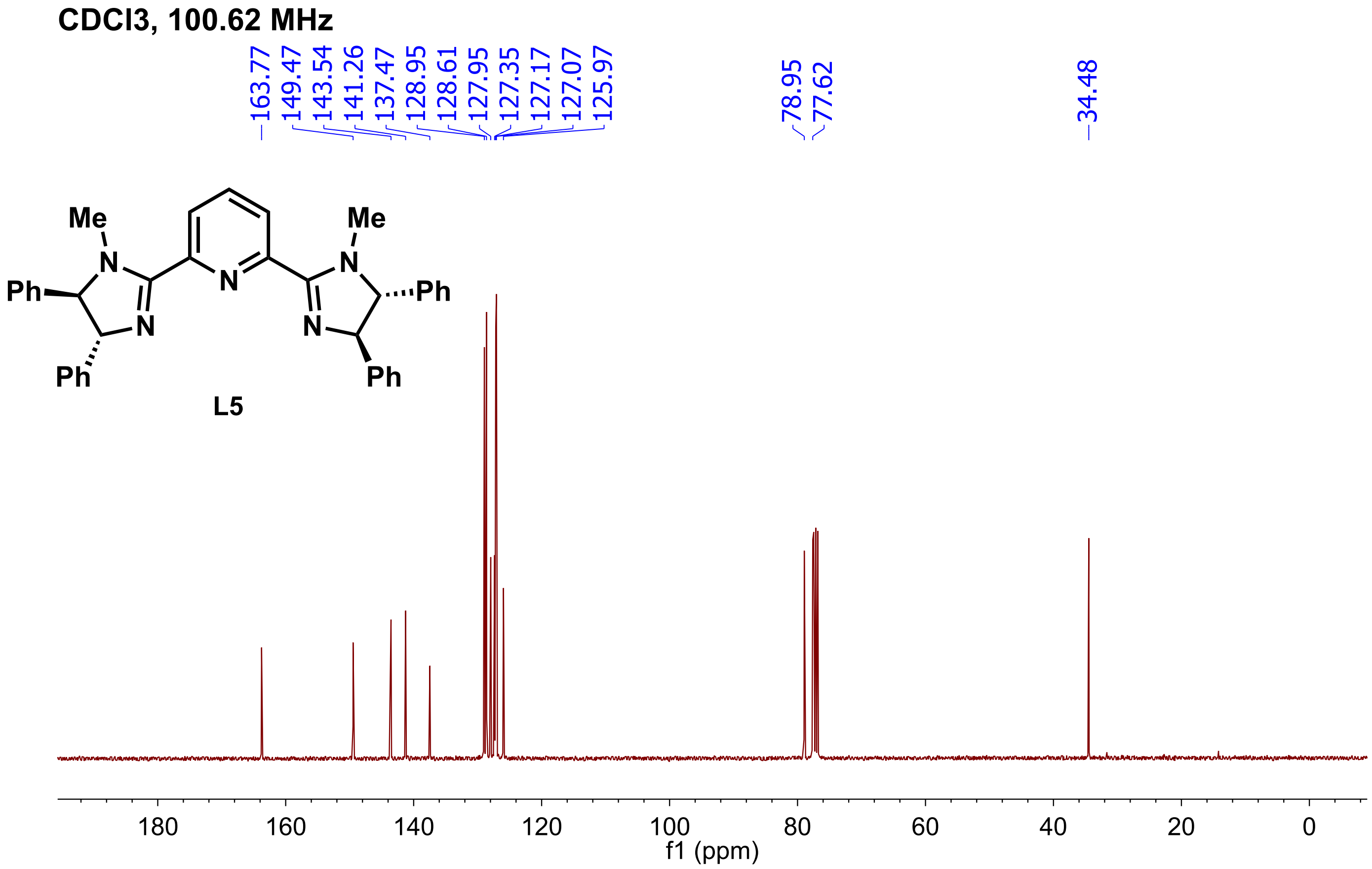

Supplementary Figure 105. ^{13}C NMR (101 MHz, $CDCl_3$) spectra for **L5**

CDCl3, 400.13 MHz

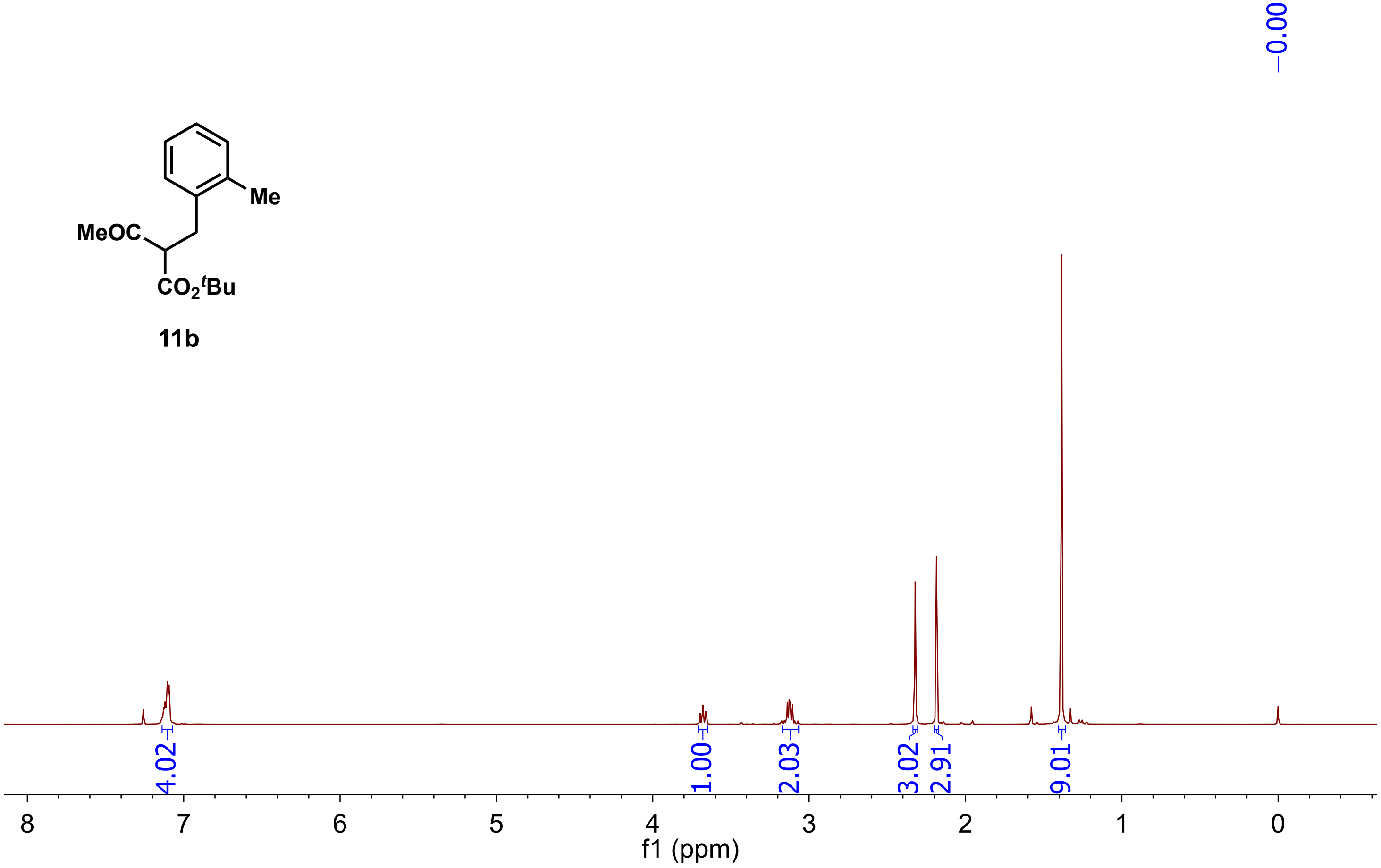

Supplementary Figure 106. ^{1}H NMR (400 MHz, $CDCl_3$) spectra for **11b**

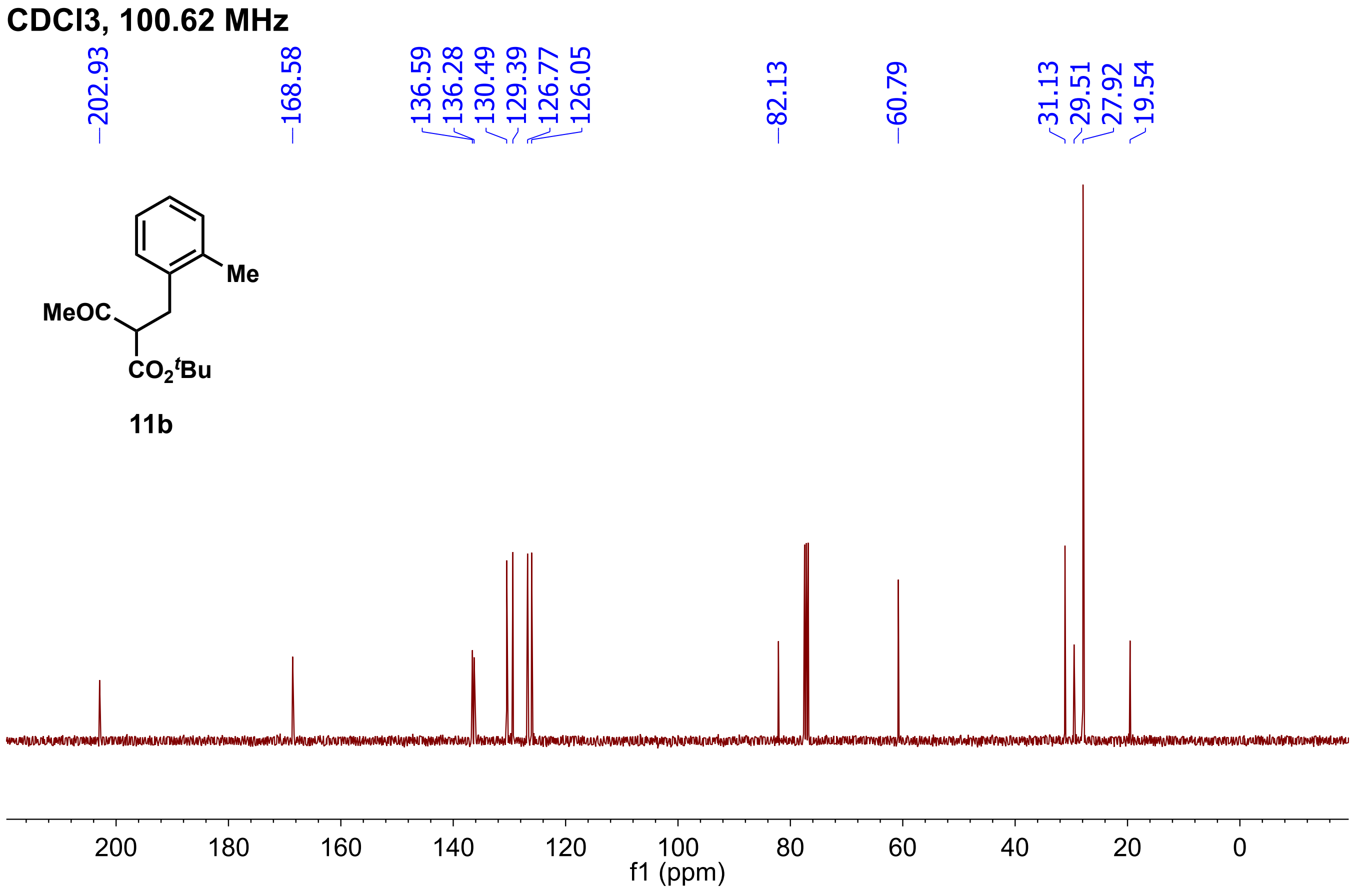

Supplementary Figure 107. ^{13}C NMR (101 MHz, $CDCl_3$) spectra for **11b**

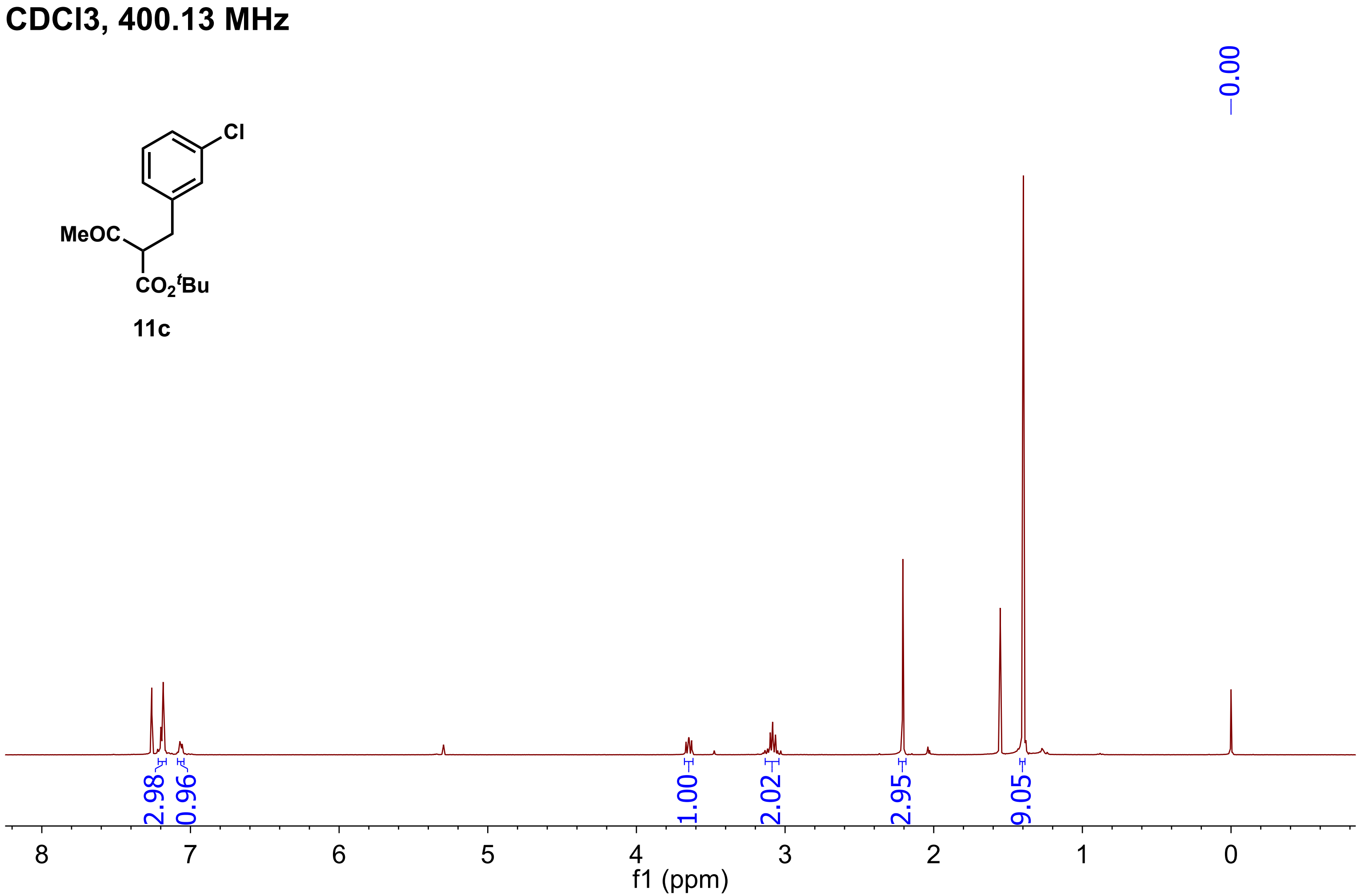

Supplementary Figure 108. ^{1}H NMR (400 MHz, $CDCl_3$) spectra for **11c**

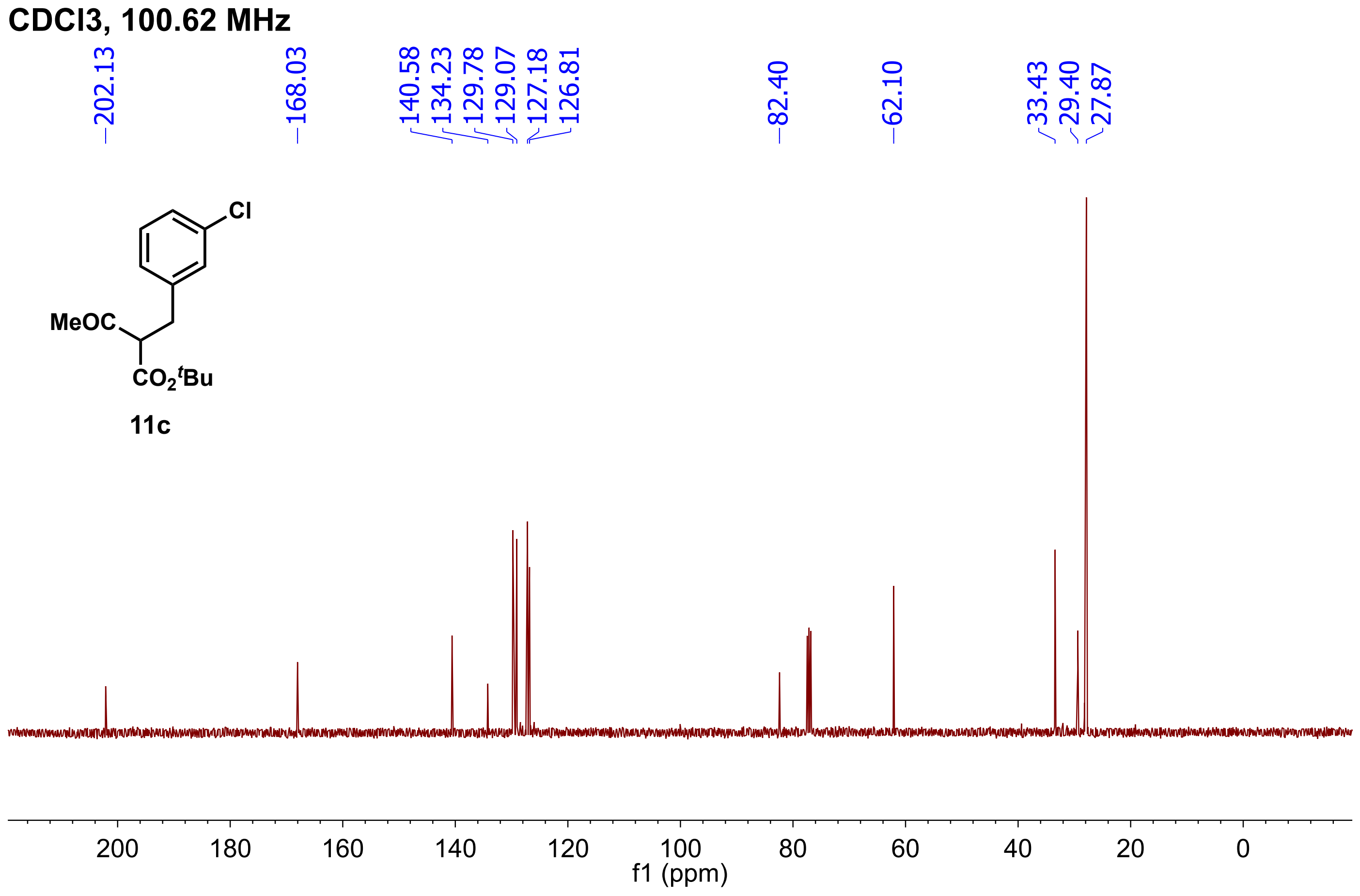

Supplementary Figure 109. ^{13}C NMR (101 MHz, $CDCl_3$) spectra for **11c**

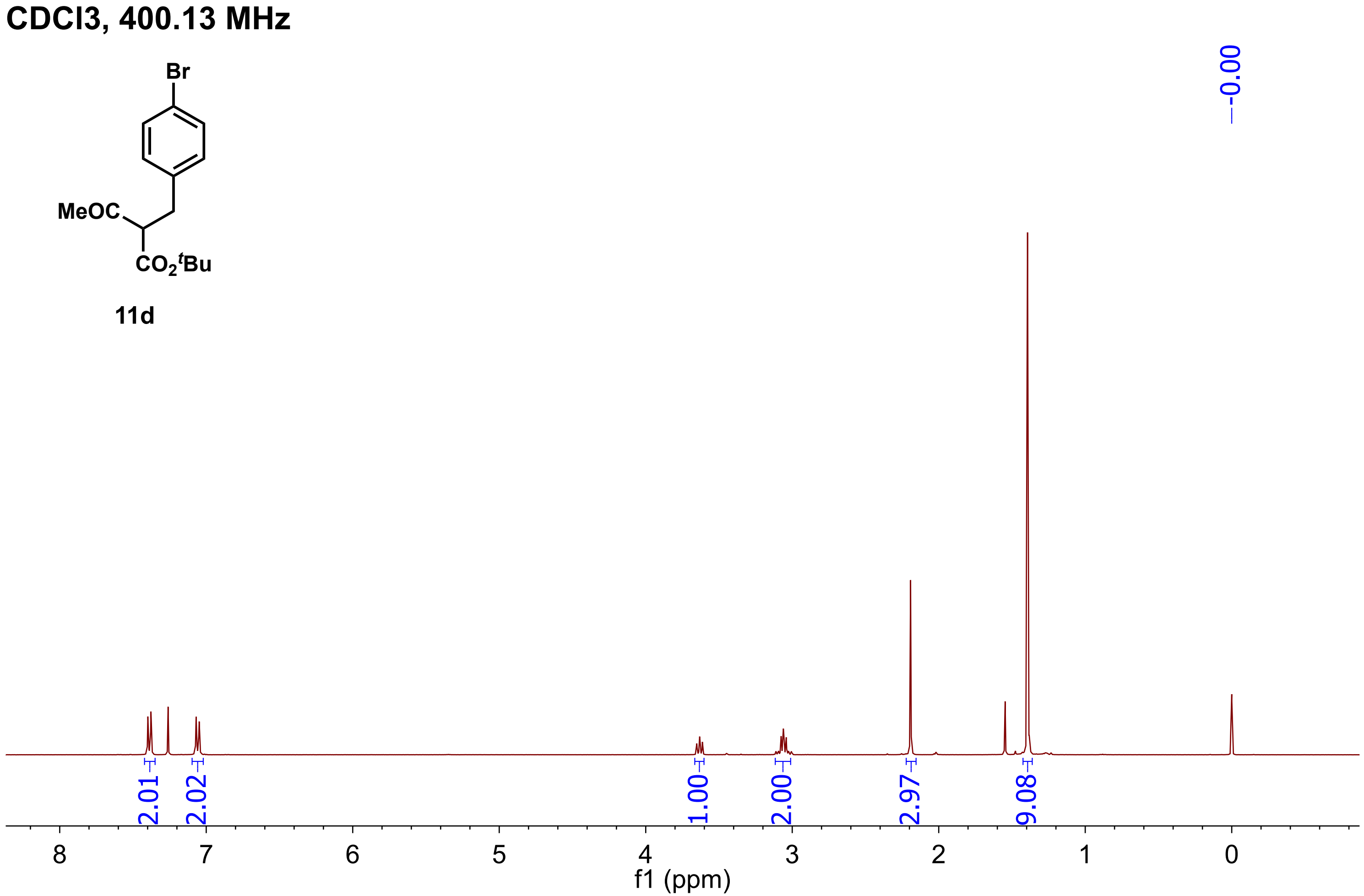

Supplementary Figure 110. ^{1}H NMR (400 MHz, $CDCl_3$) spectra for **11d**

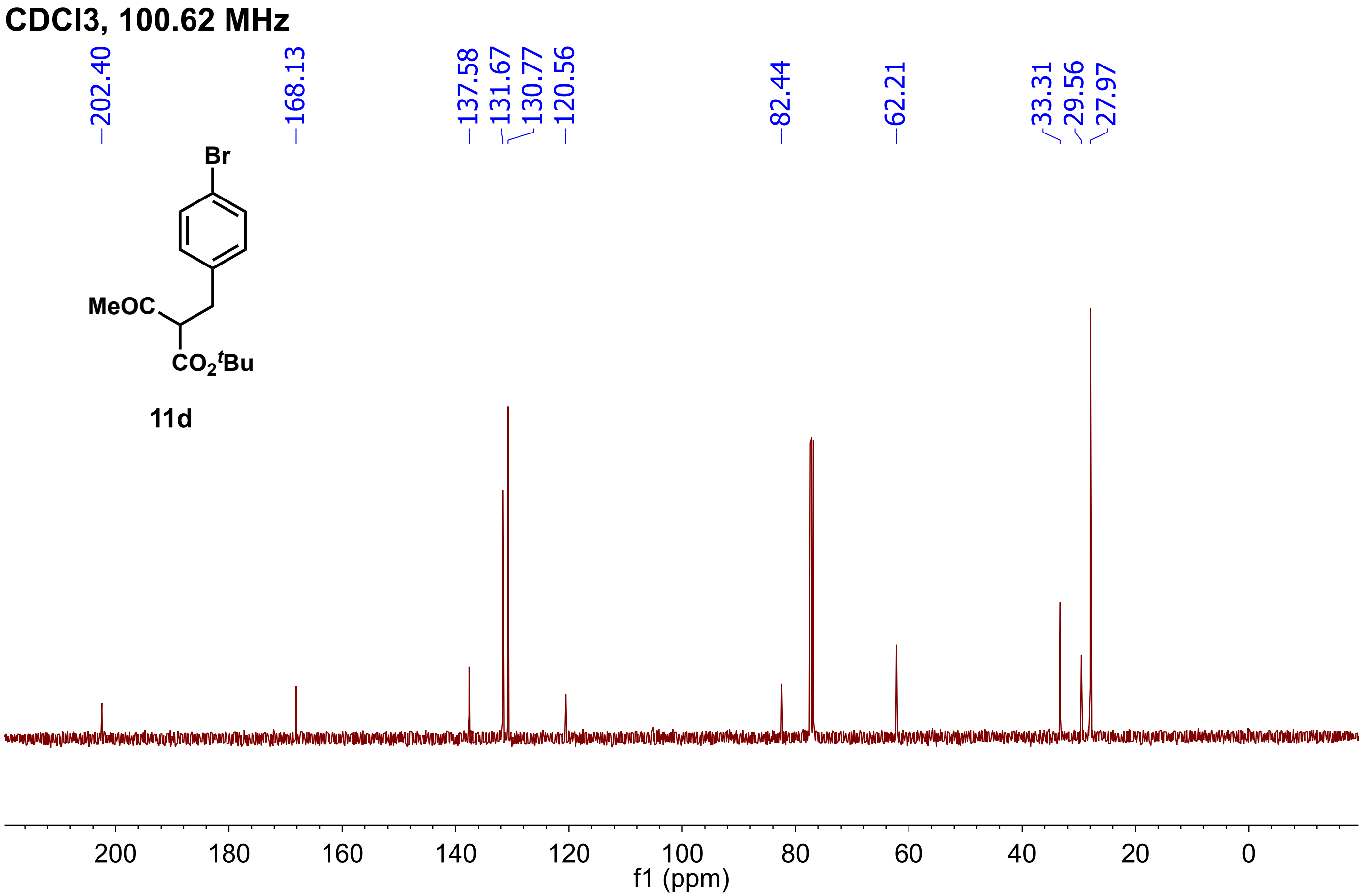

Supplementary Figure 111. ^{13}C NMR (101 MHz, $CDCl_3$) spectra for **11d**

CDCl3, 400.13 MHz

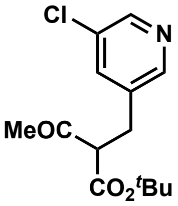

11f

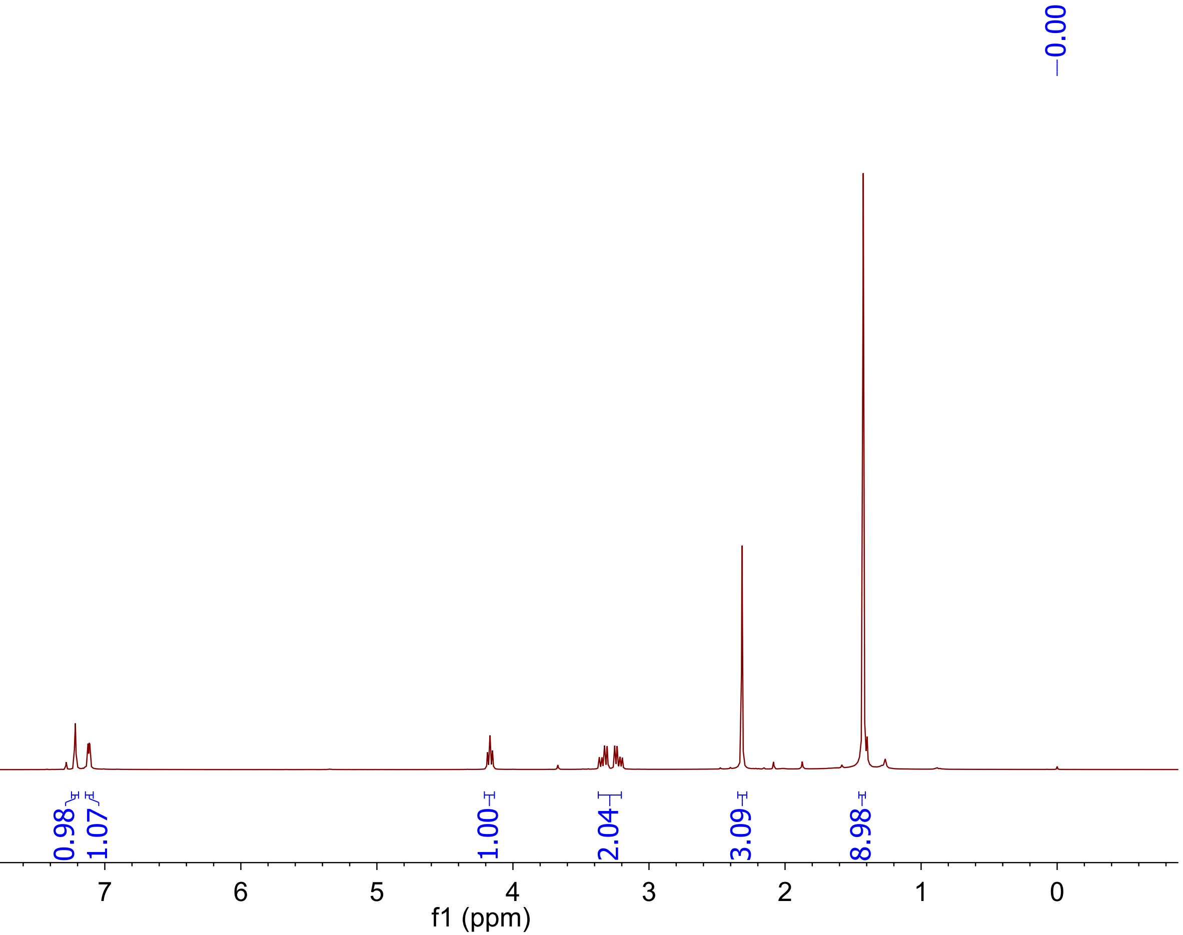

Supplementary Figure 112. ^{1}H NMR (400 MHz, $CDCl_3$) spectra for **11f**

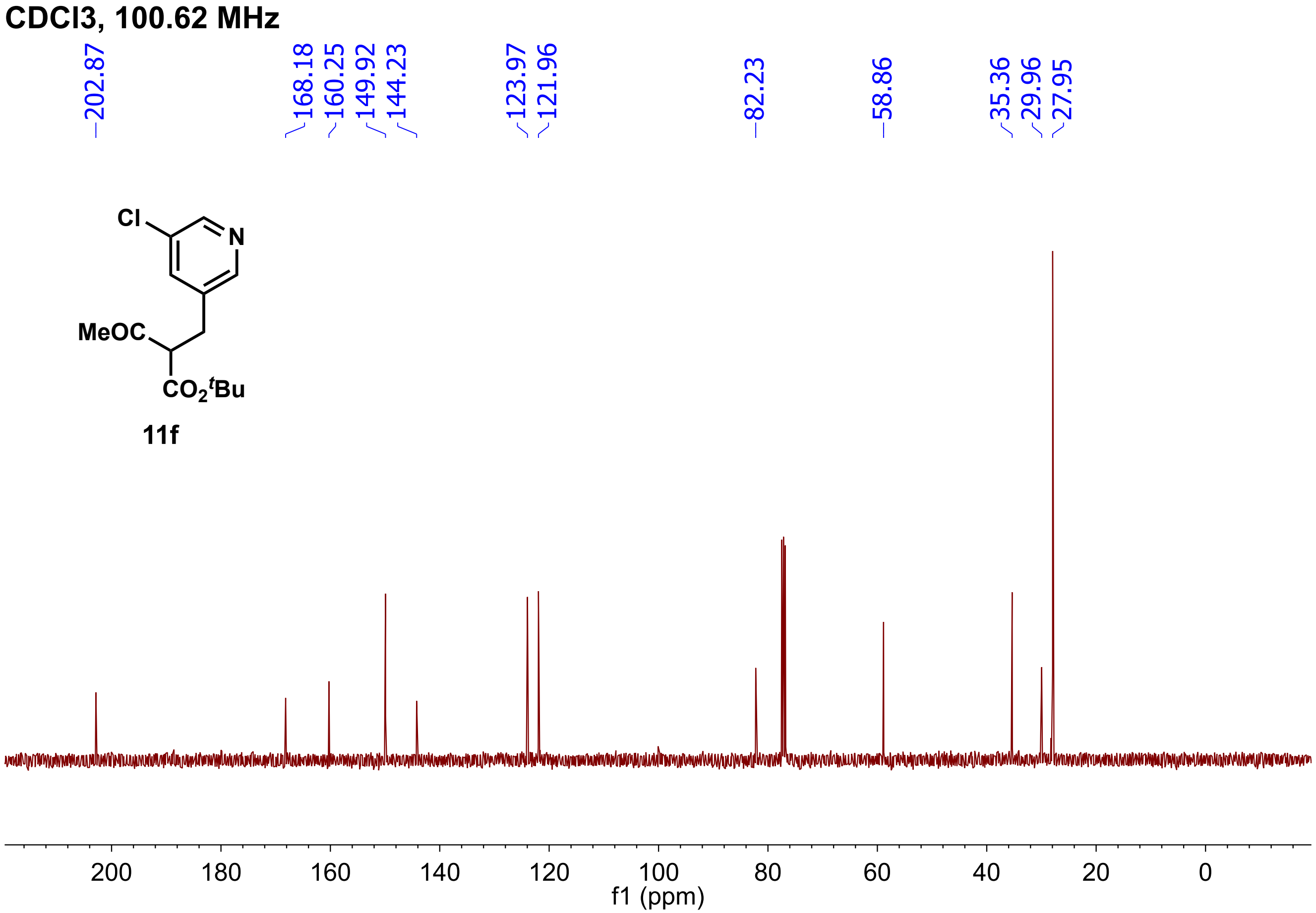

Supplementary Figure 113. ^{13}C NMR (101 MHz, $CDCl_3$) spectra for **11f**

CDCl3, 400.13 MHz

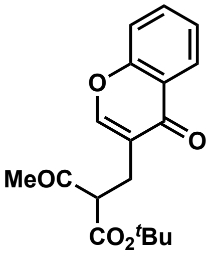

11g

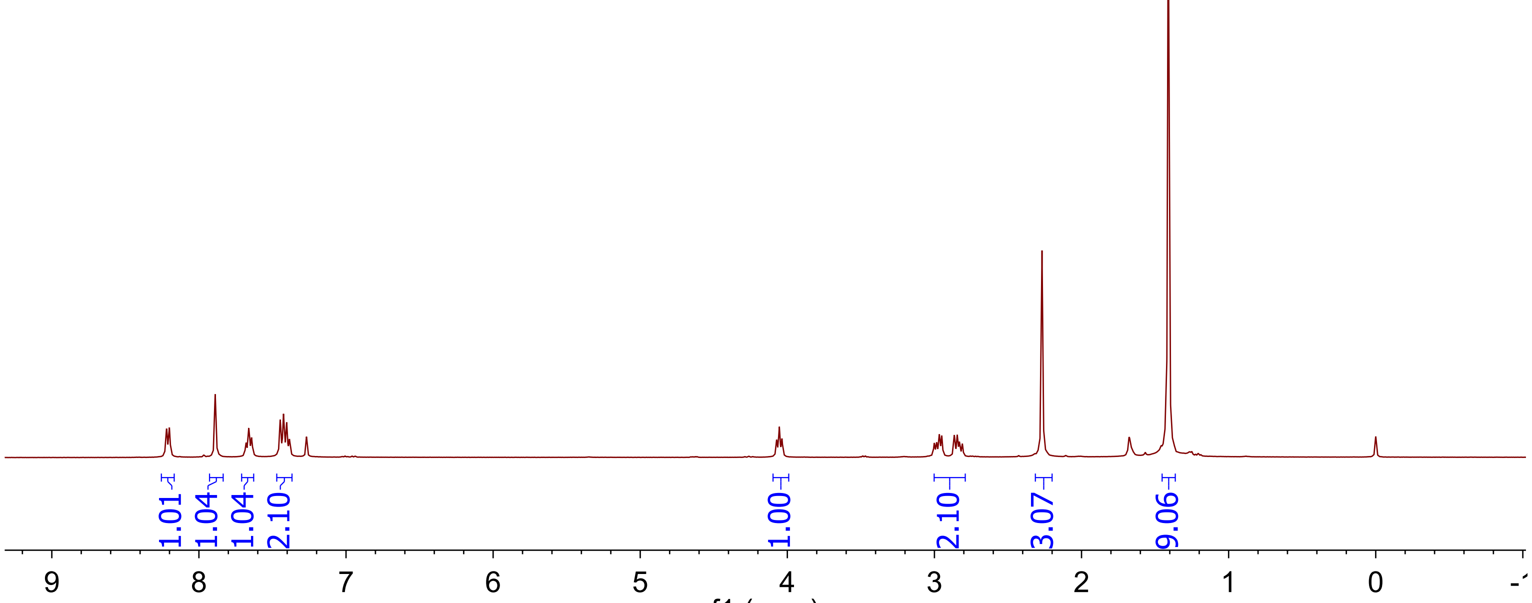

Supplementary Figure 114. ^{1}H NMR (400 MHz, $CDCl_3$) spectra for **11g**

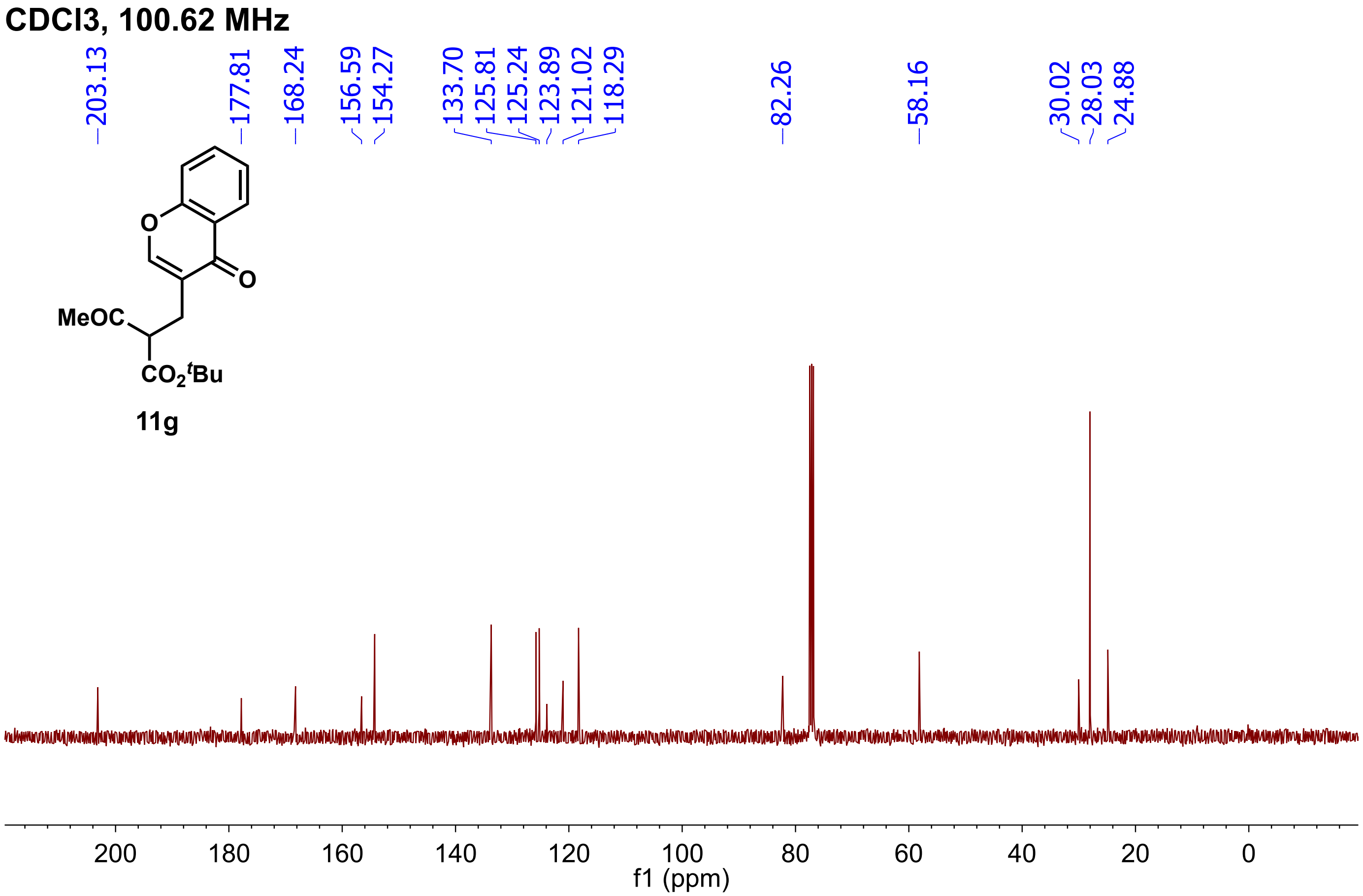

Supplementary Figure 115. ^{13}C NMR (101 MHz, $CDCl_3$) spectra for **11g**

CDCl3, 400.13 MHz

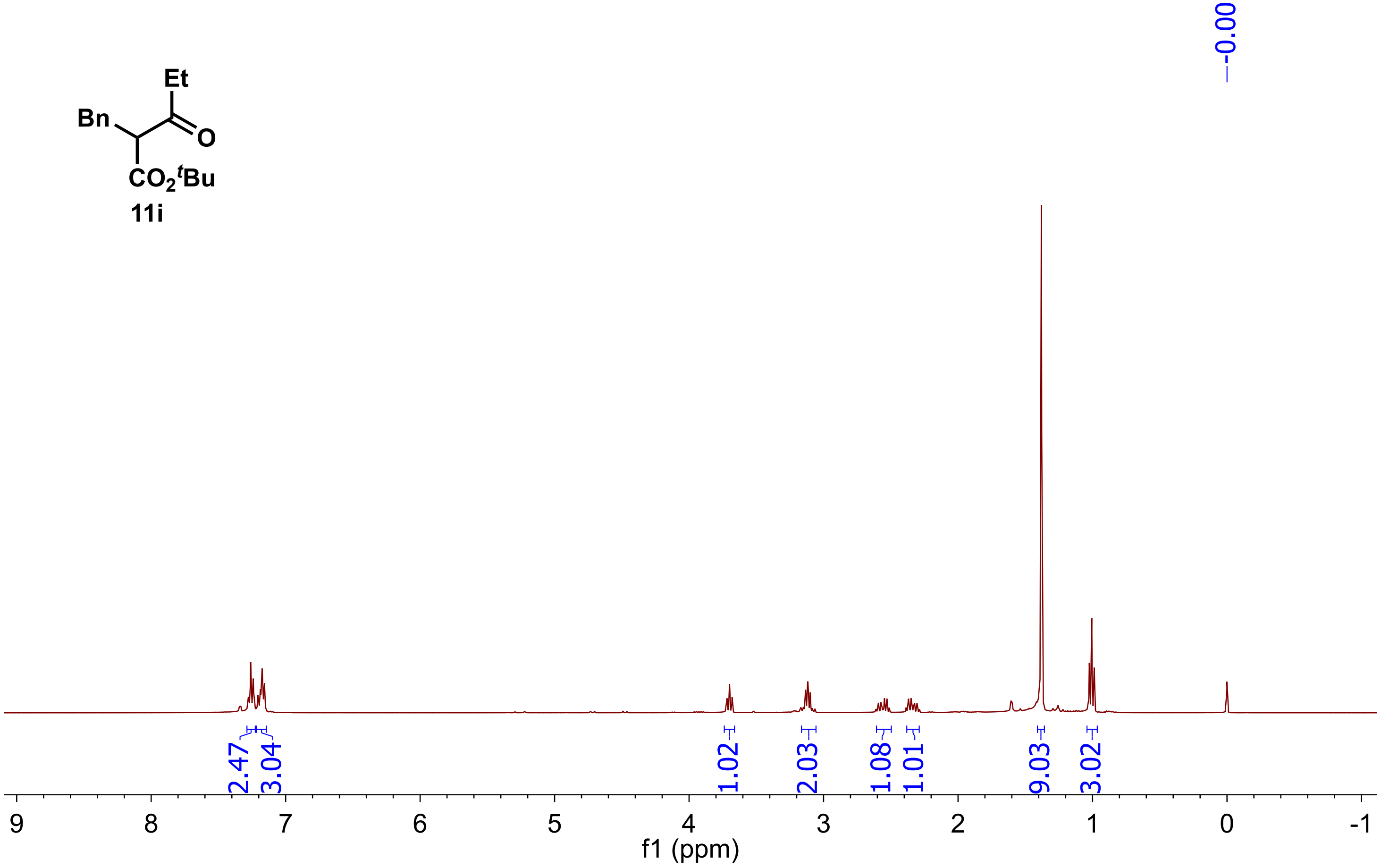

Supplementary Figure 116. ^{1}H NMR (400 MHz, $CDCl_3$) spectra for **11i**

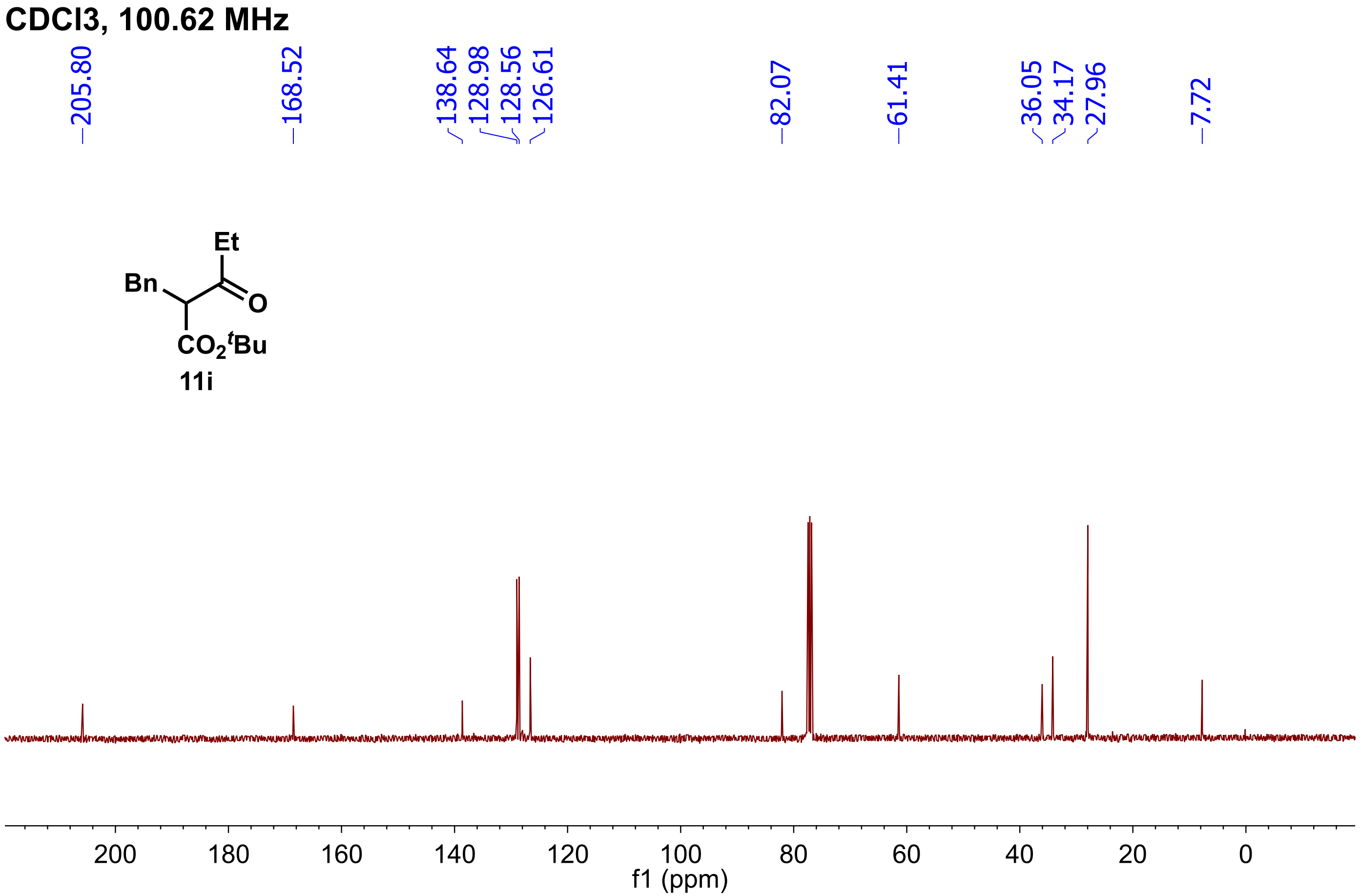

Supplementary Figure 117. ^{13}C NMR (101 MHz, $CDCl_3$) spectra for **11i**

CDCl3, 400.13 MHz

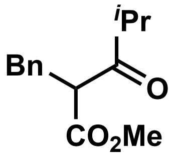

11j

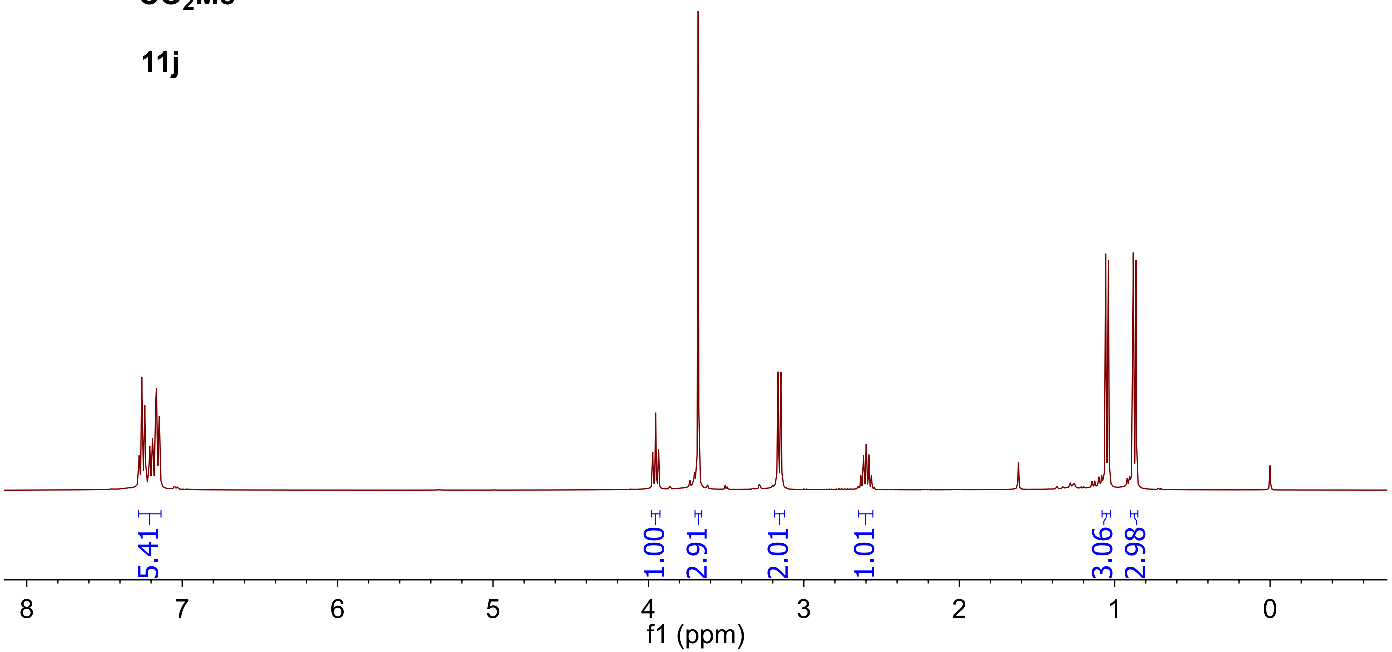

Supplementary Figure 118. ^{1}H NMR (400 MHz, $CDCl_3$) spectra for **11j**

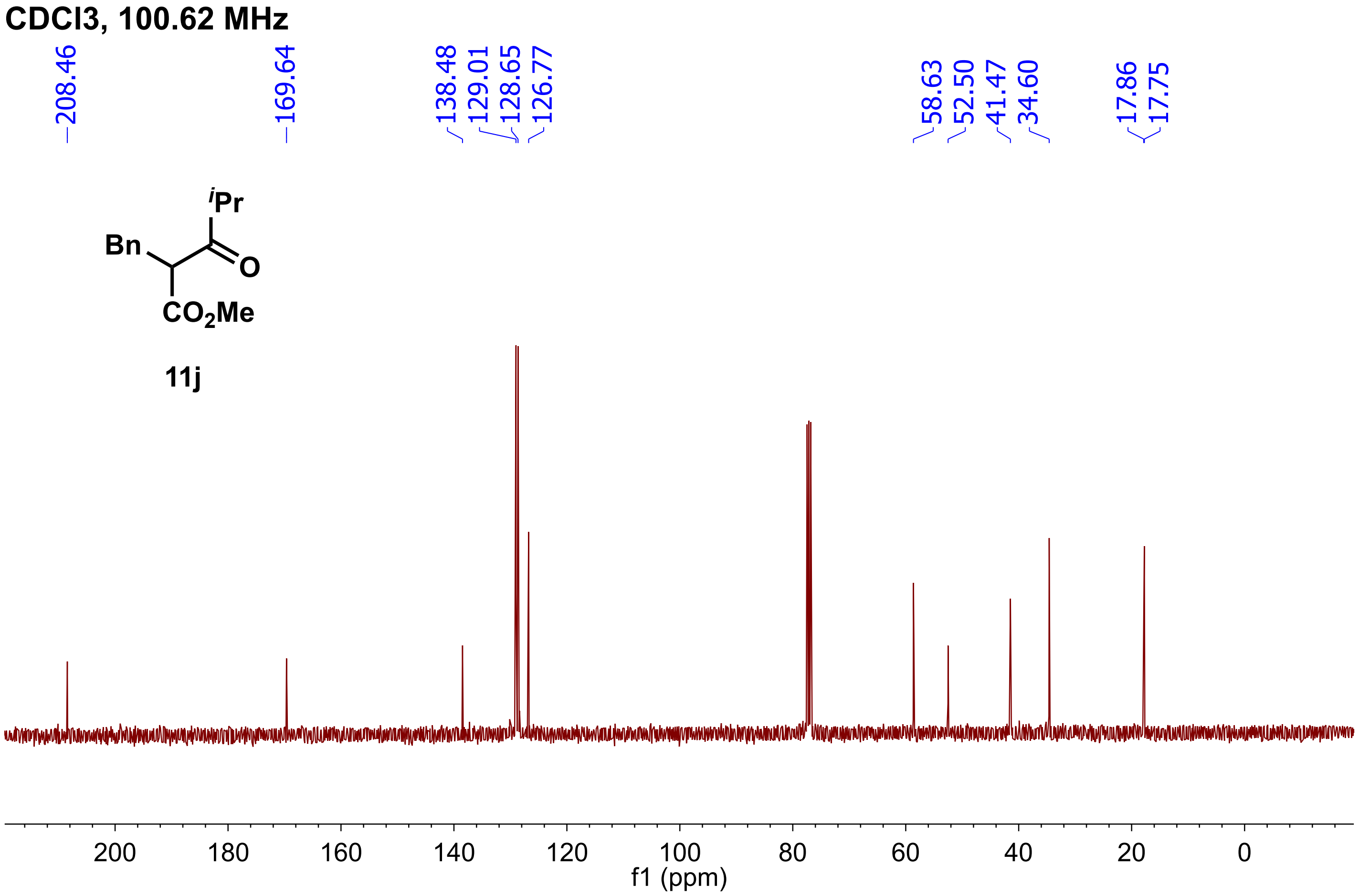

Supplementary Figure 119. ^{13}C NMR (101 MHz, $CDCl_3$) spectra for **11j**

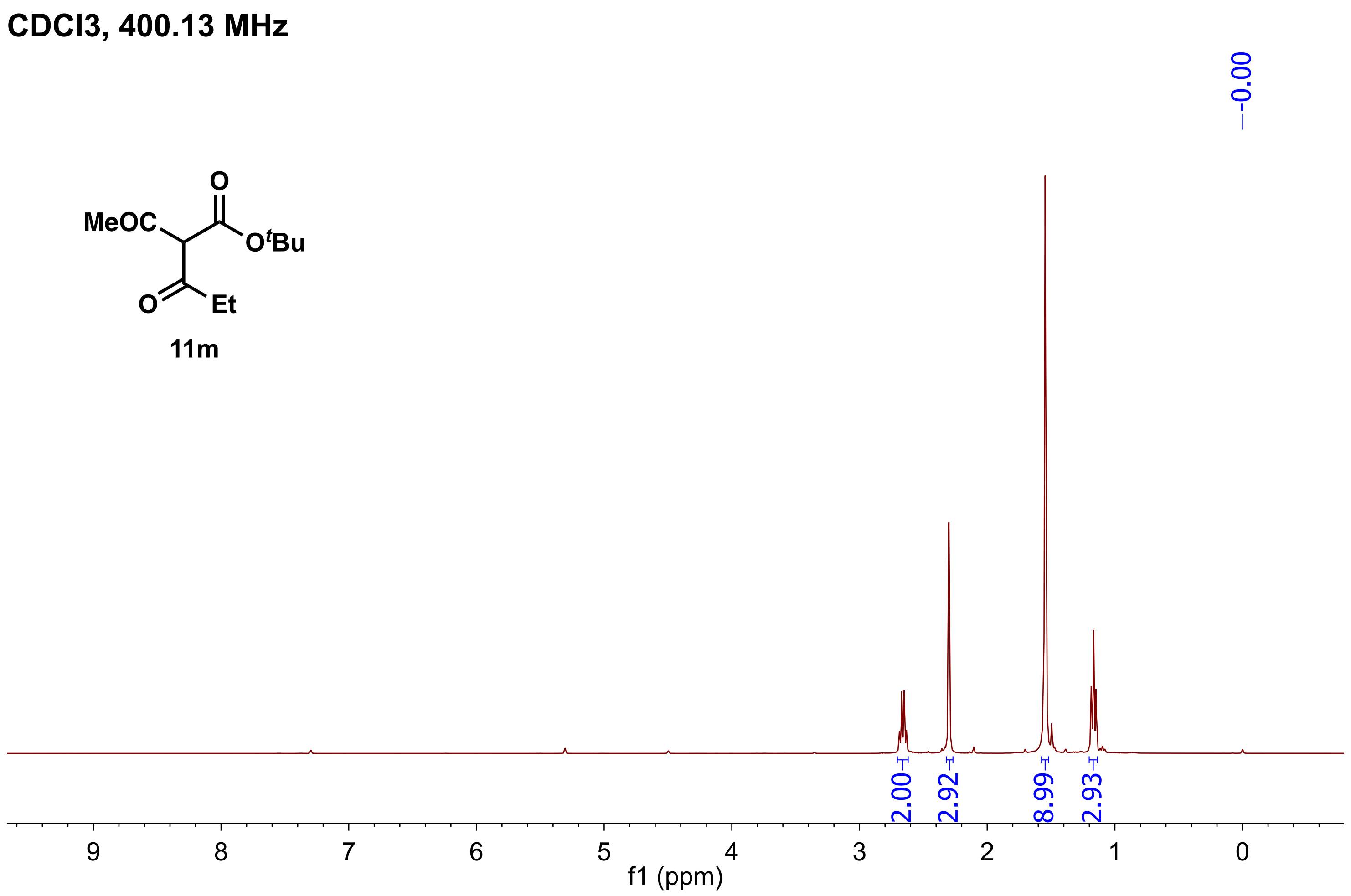

Supplementary Figure 120. ^{1}H NMR (400 MHz, $CDCl_3$) spectra for **11m**

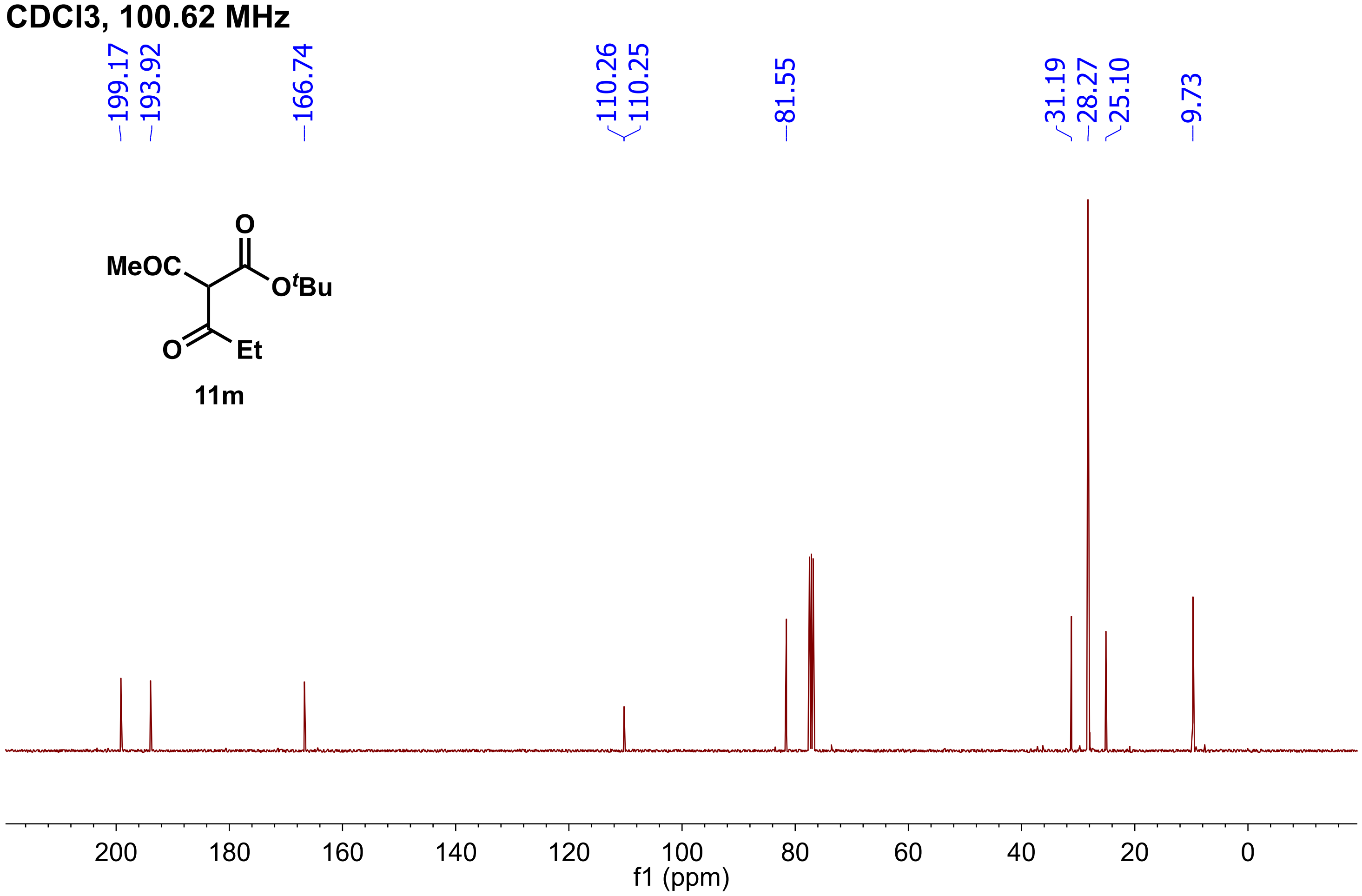

Supplementary Figure 121. ^{13}C NMR (101 MHz, $CDCl_3$) spectra for **11m**

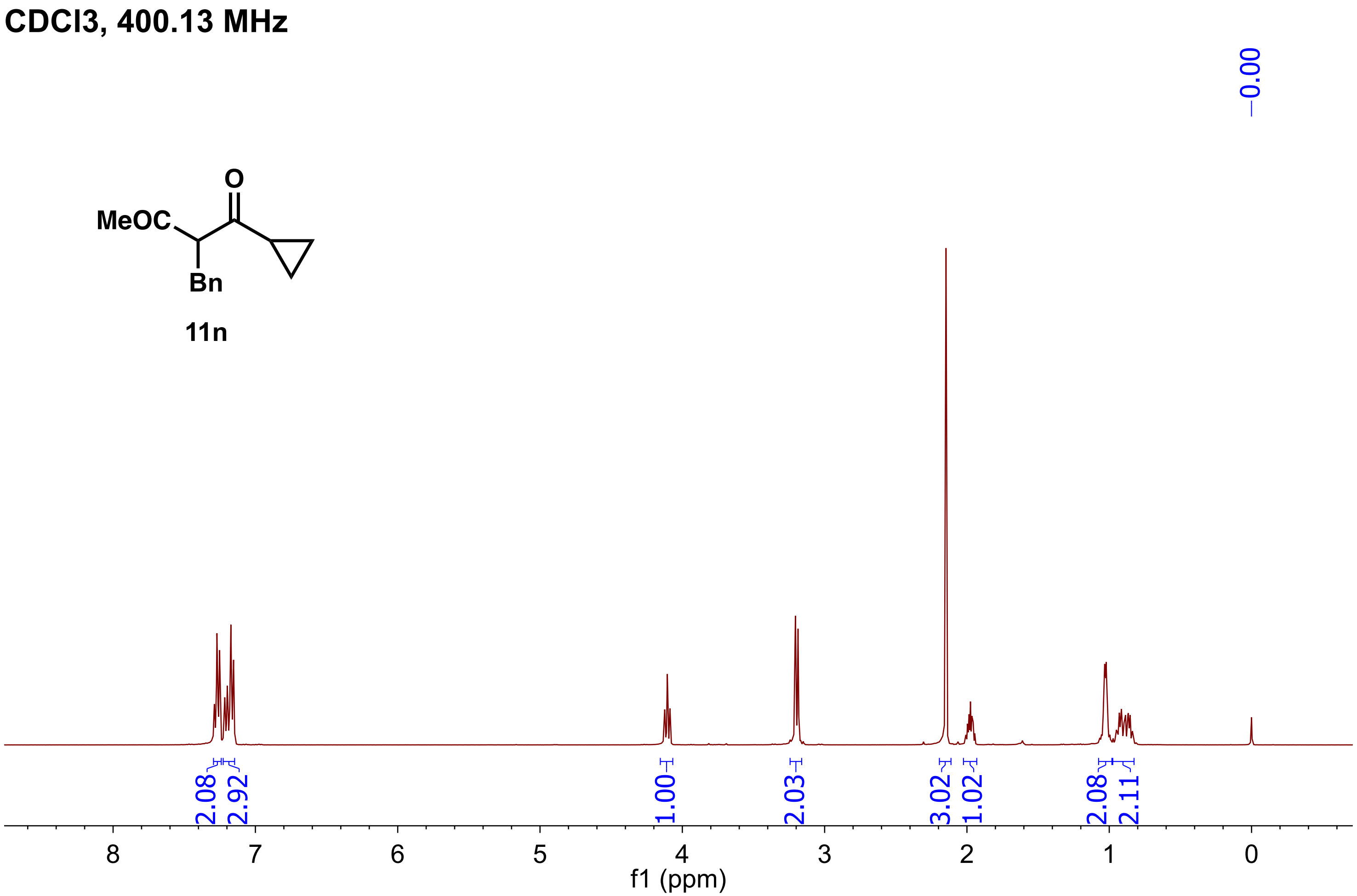

Supplementary Figure 122. ^{1}H NMR (400 MHz, $CDCl_3$) spectra for **11n**

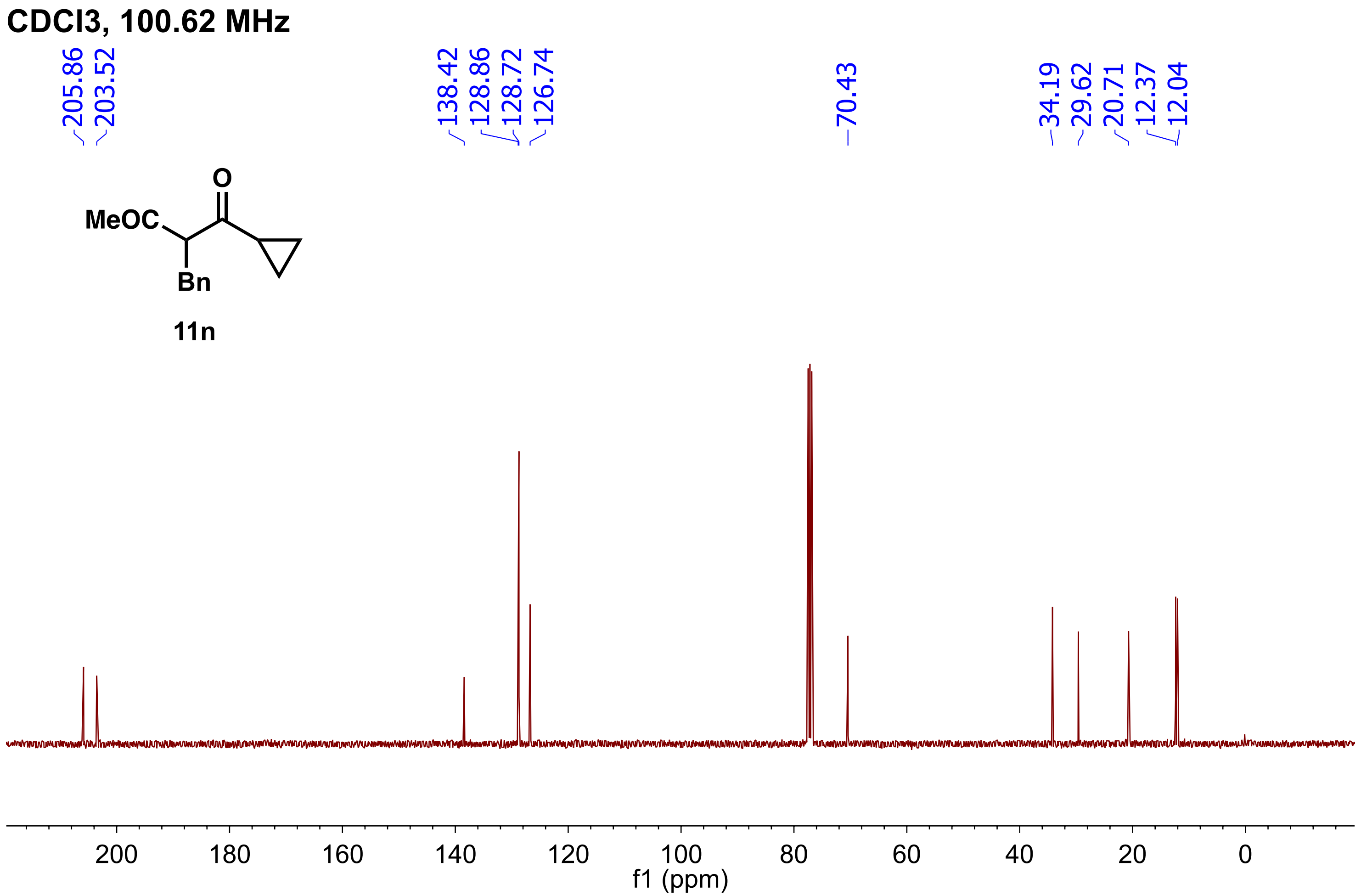

Supplementary Figure 123. ^{13}C NMR (101 MHz, $CDCl_3$) spectra for **11n**

CDCl3, 400.13 MHz

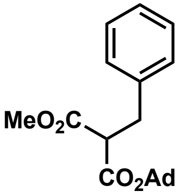

11o

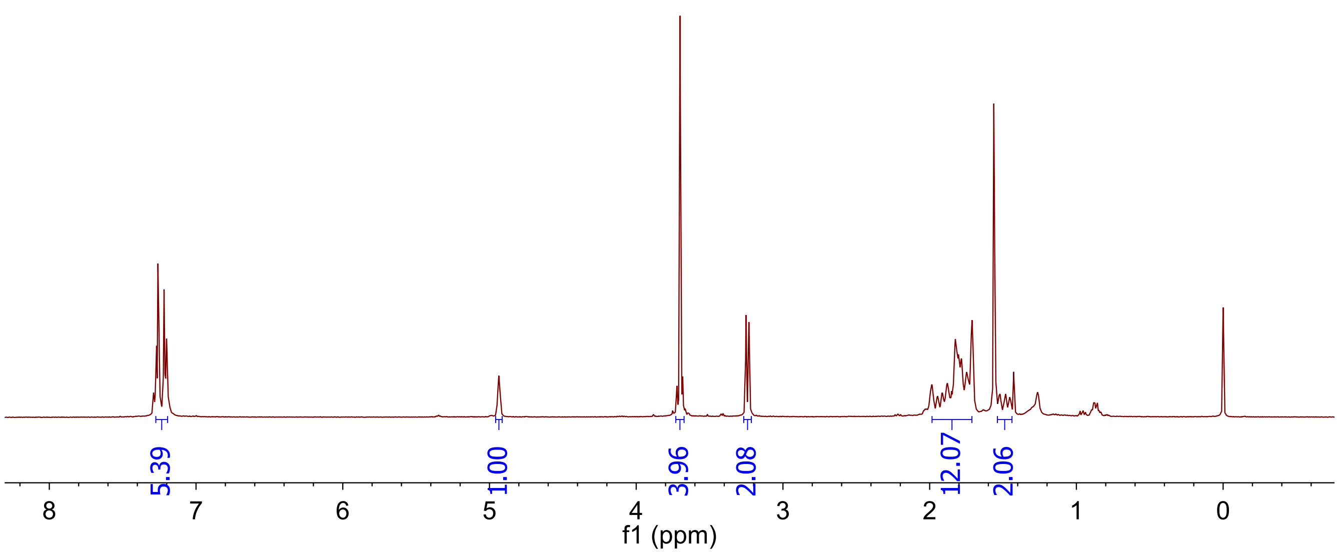

Supplementary Figure 124. ^{1}H NMR (400 MHz, $CDCl_3$) spectra for **11o**

CDCl3, 100.62 MHz

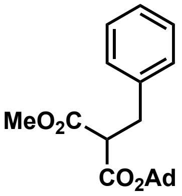

11o

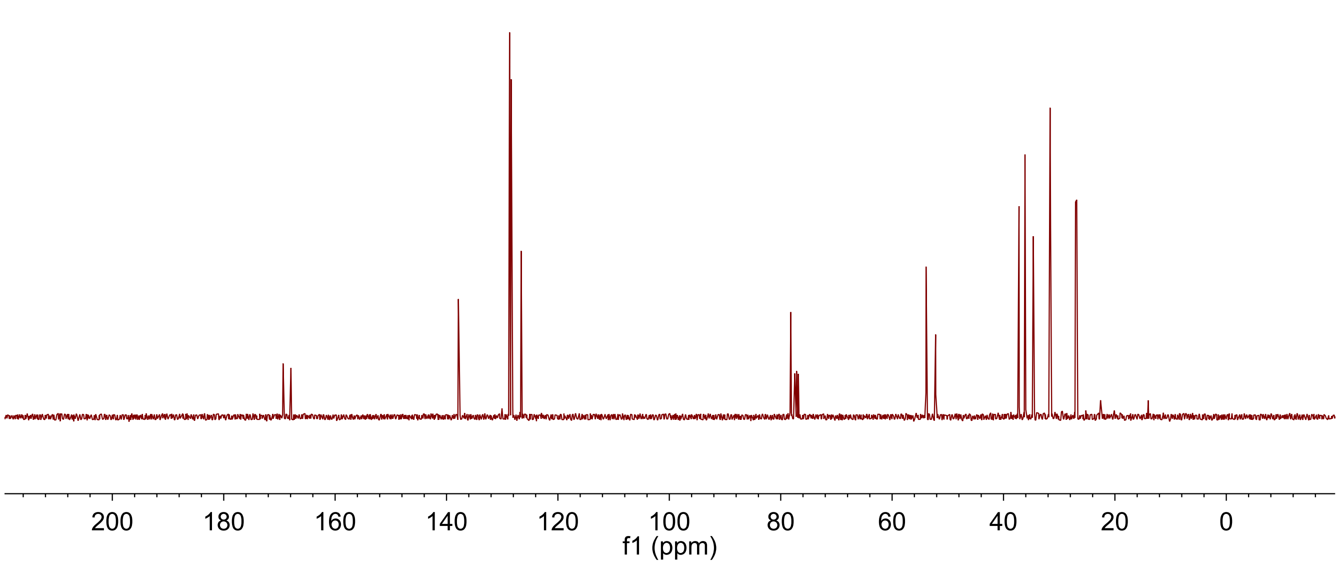

Supplementary Figure 125. ^{13}C NMR (101 MHz, $CDCl_3$) spectra for **11o**

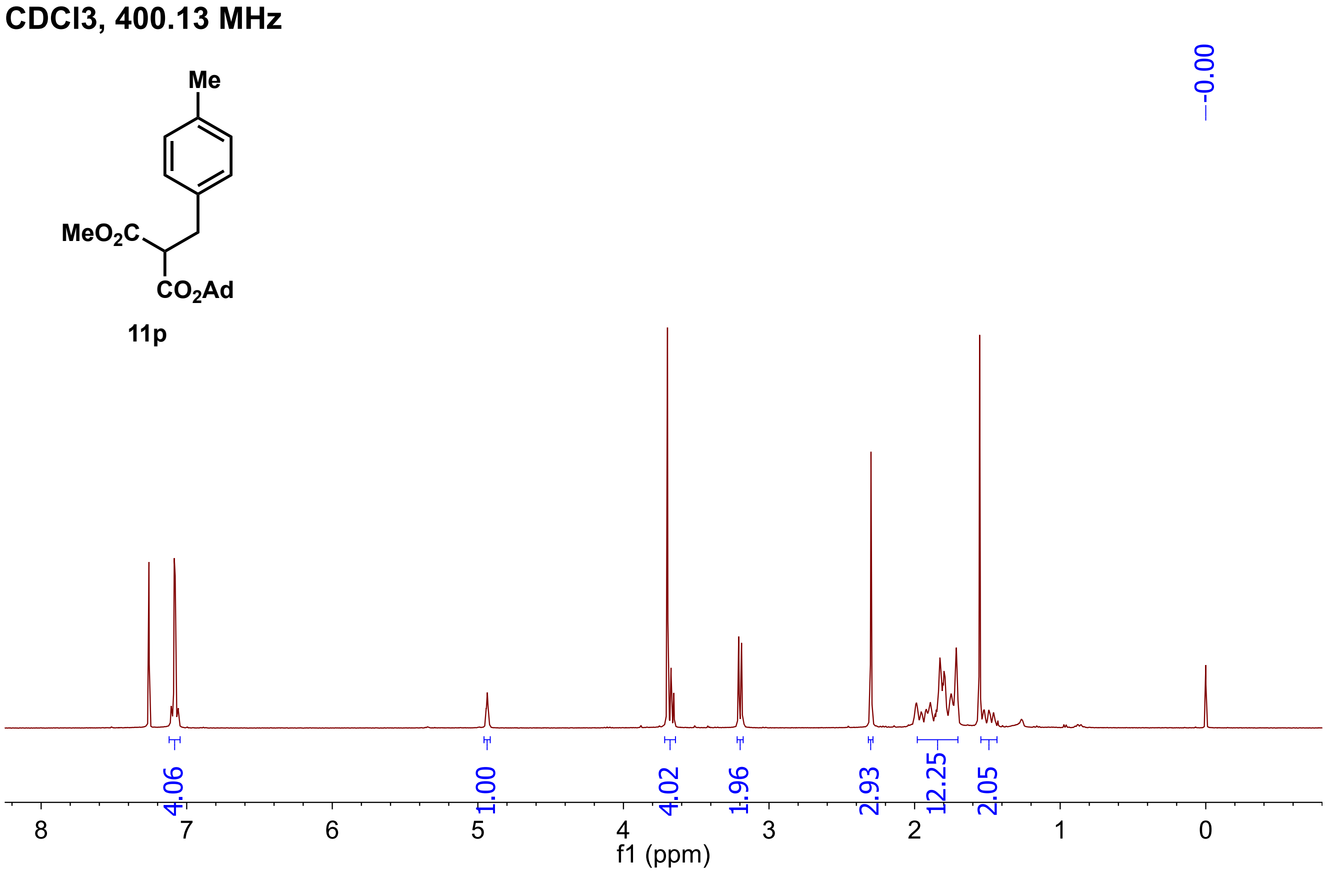

Supplementary Figure 126. ^{1}H NMR (400 MHz, $CDCl_3$) spectra for **11p**

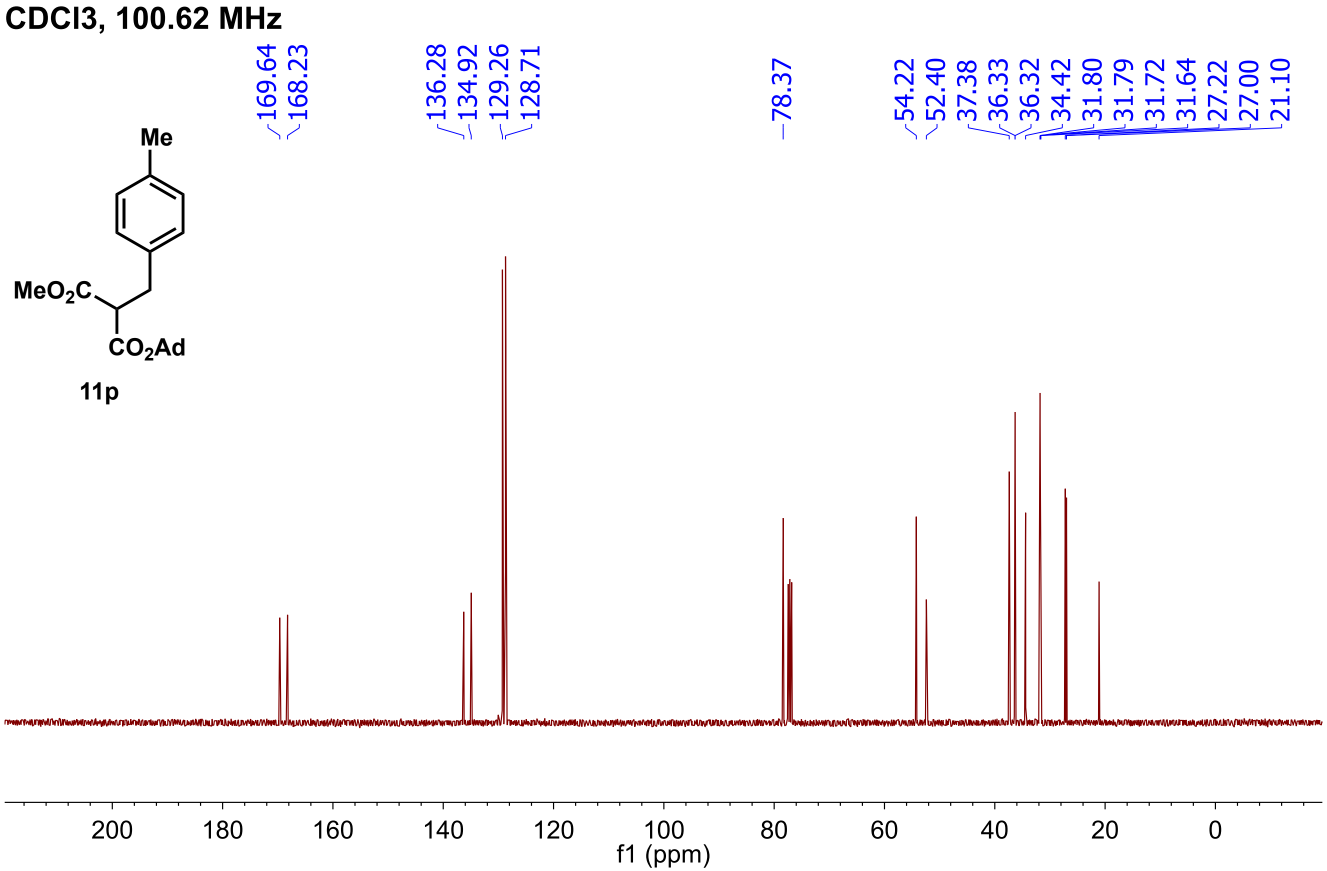

Supplementary Figure 127. ^{13}C NMR (101 MHz, $CDCl_3$) spectra for **11p**

CDCl3, 400.13 MHz

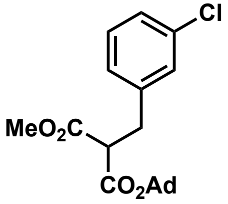

11q

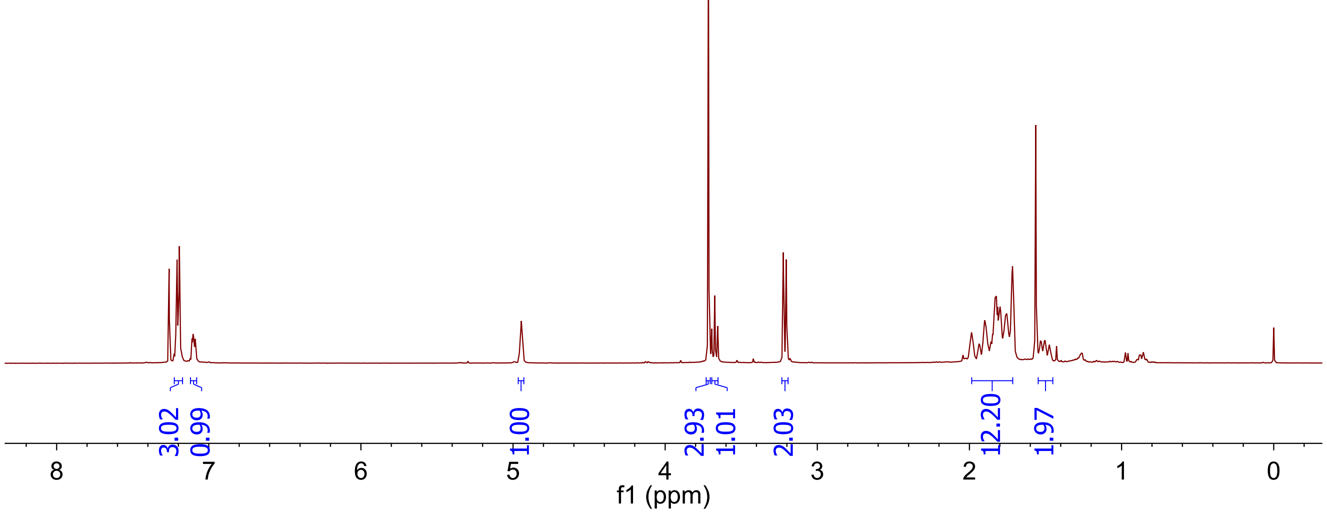

Supplementary Figure 128. ^{1}H NMR (400 MHz, $CDCl_3$) spectra for **11q**

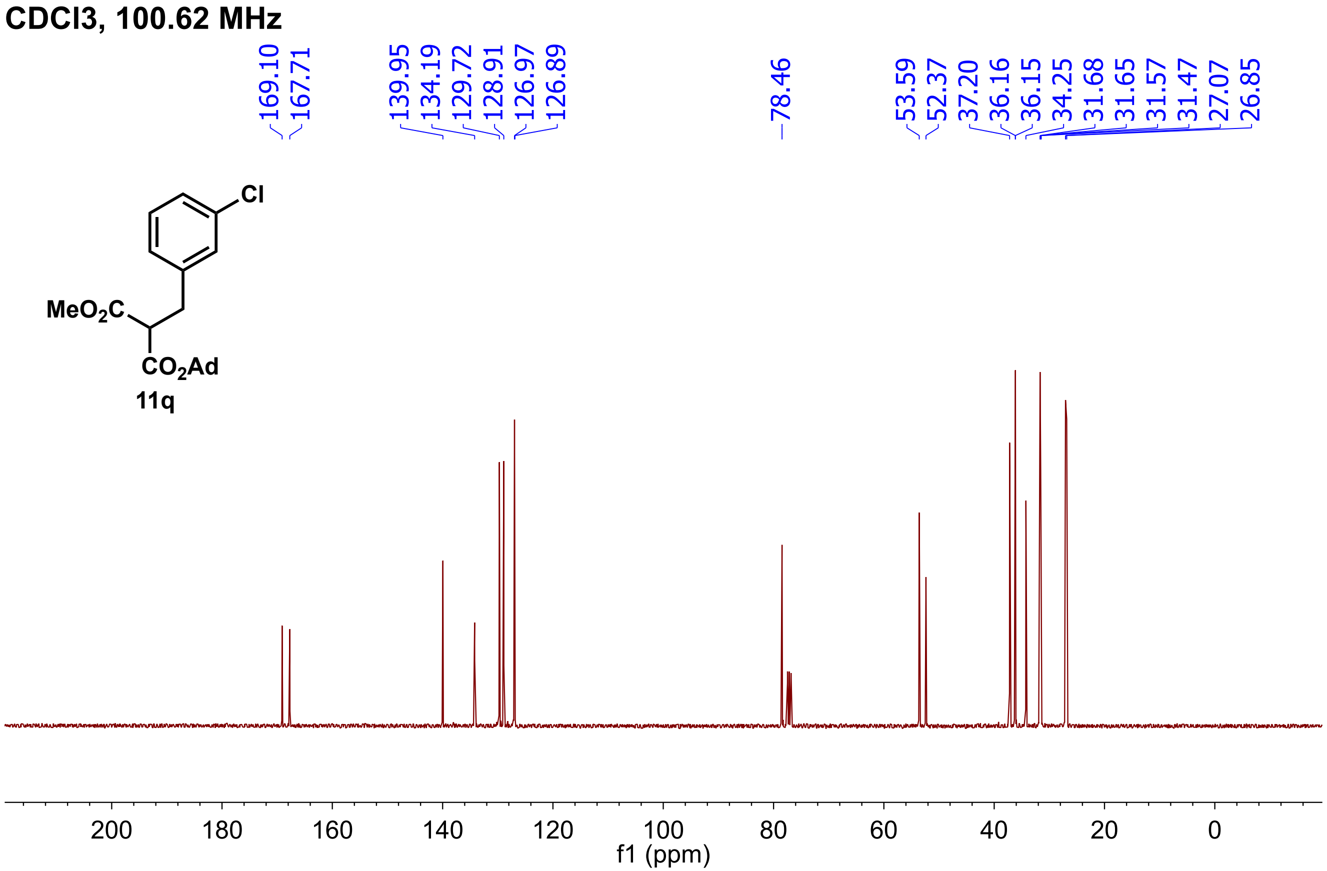

Supplementary Figure 129. ^{13}C NMR (101 MHz, $CDCl_3$) spectra for **11q**

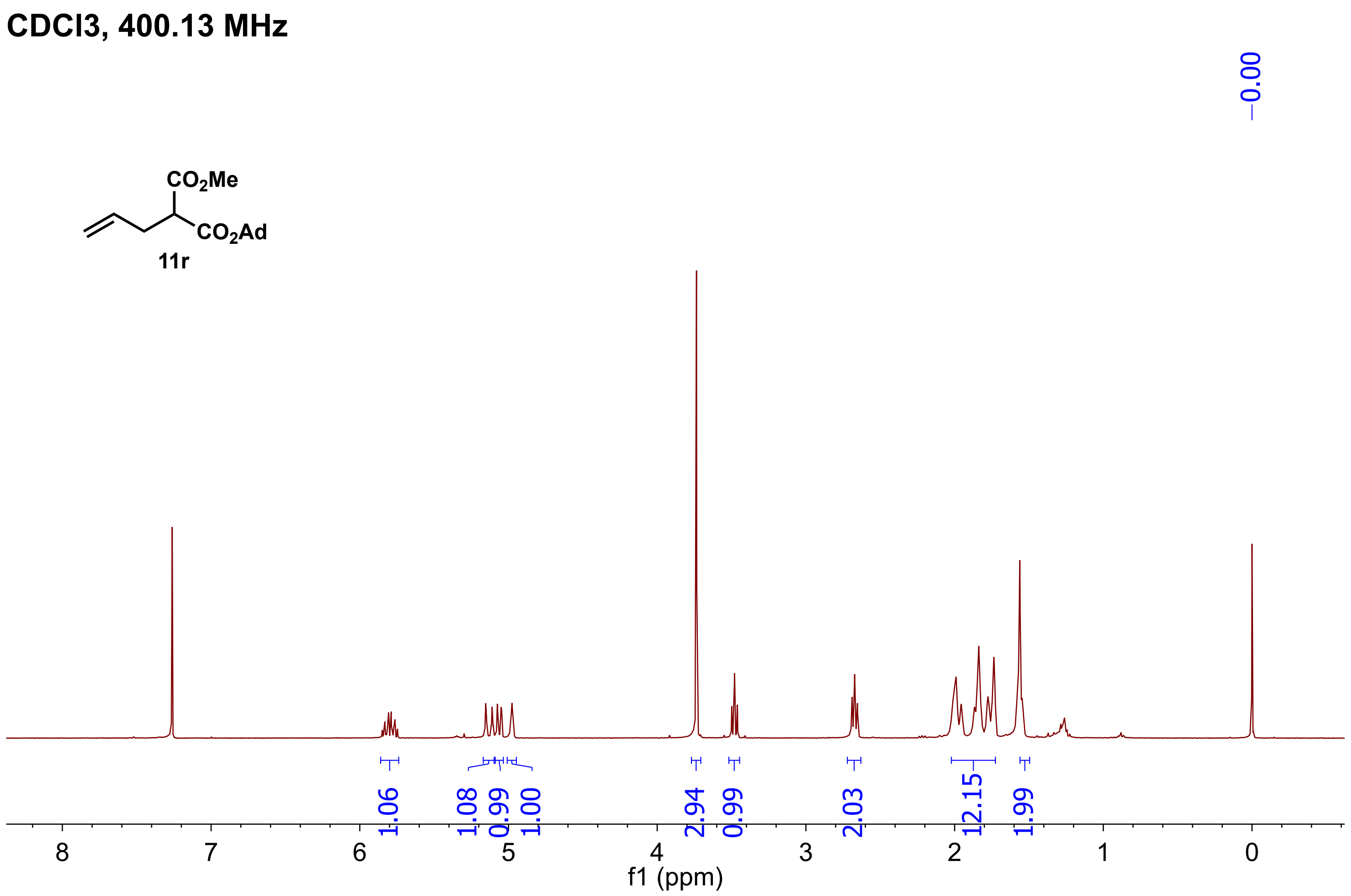

Supplementary Figure 130. ^{1}H NMR (400 MHz, $CDCl_3$) spectra for **11r**

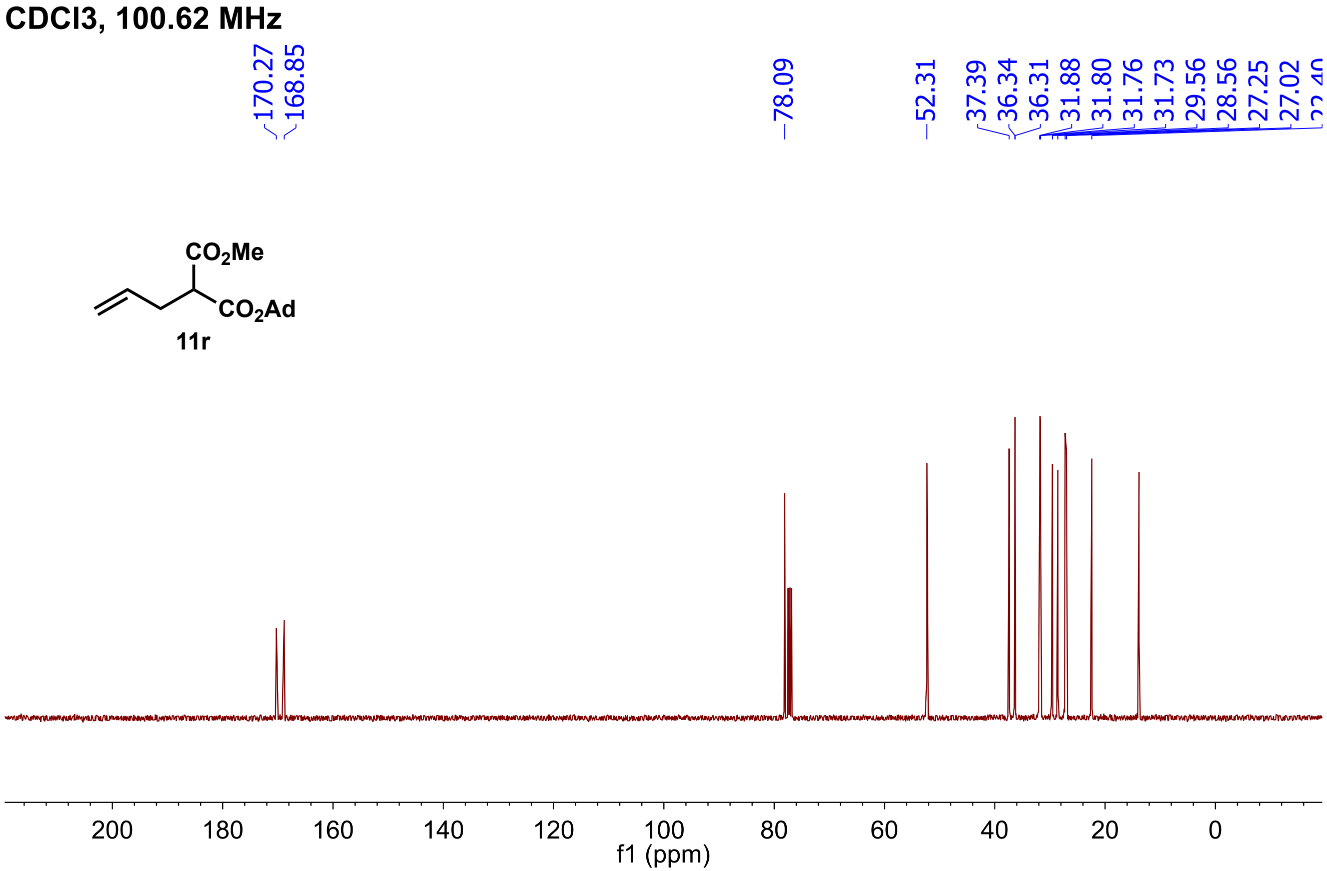

Supplementary Figure 131. ^{13}C NMR (101 MHz, $CDCl_3$) spectra for **11r**

CDCl3, 400.13 MHz

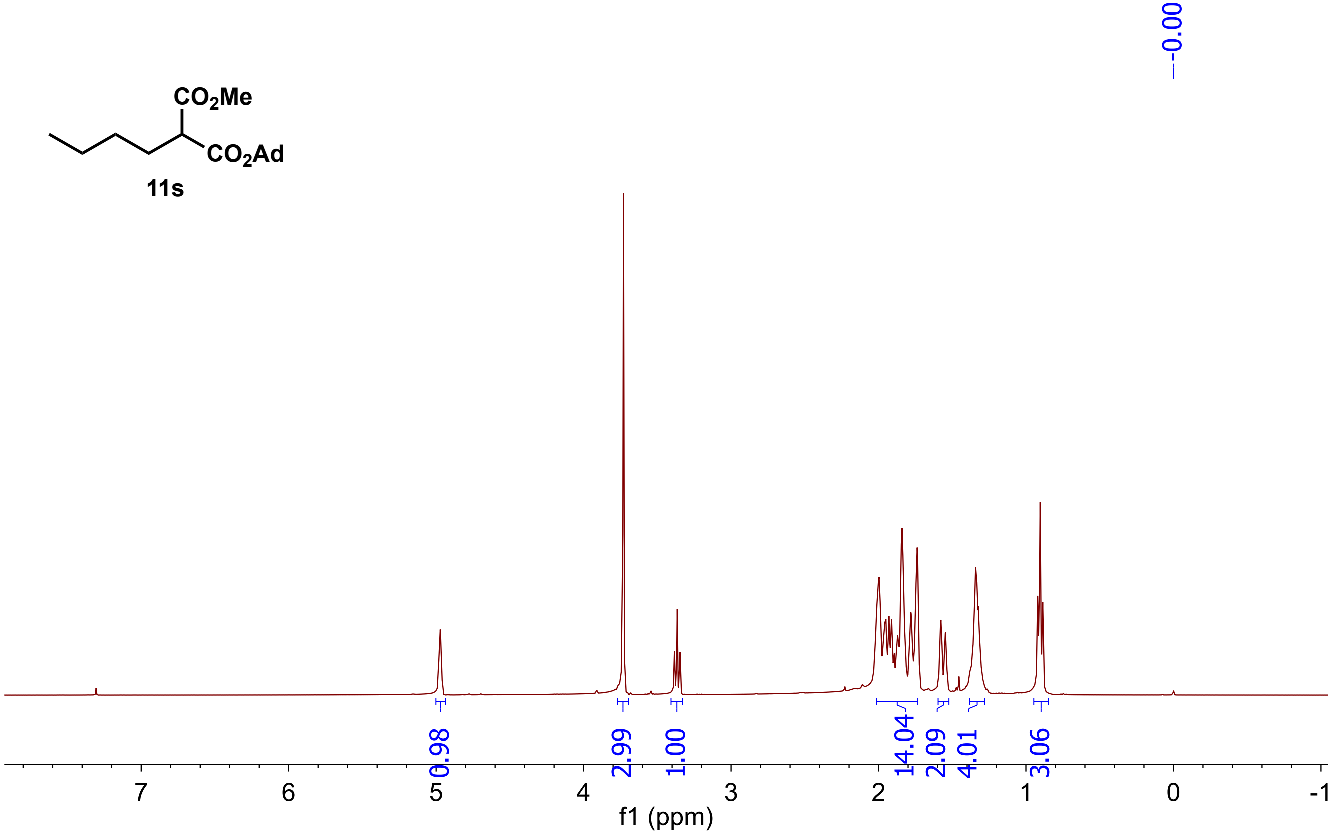

Supplementary Figure 132. ^{1}H NMR (400 MHz, $CDCl_3$) spectra for **11s**

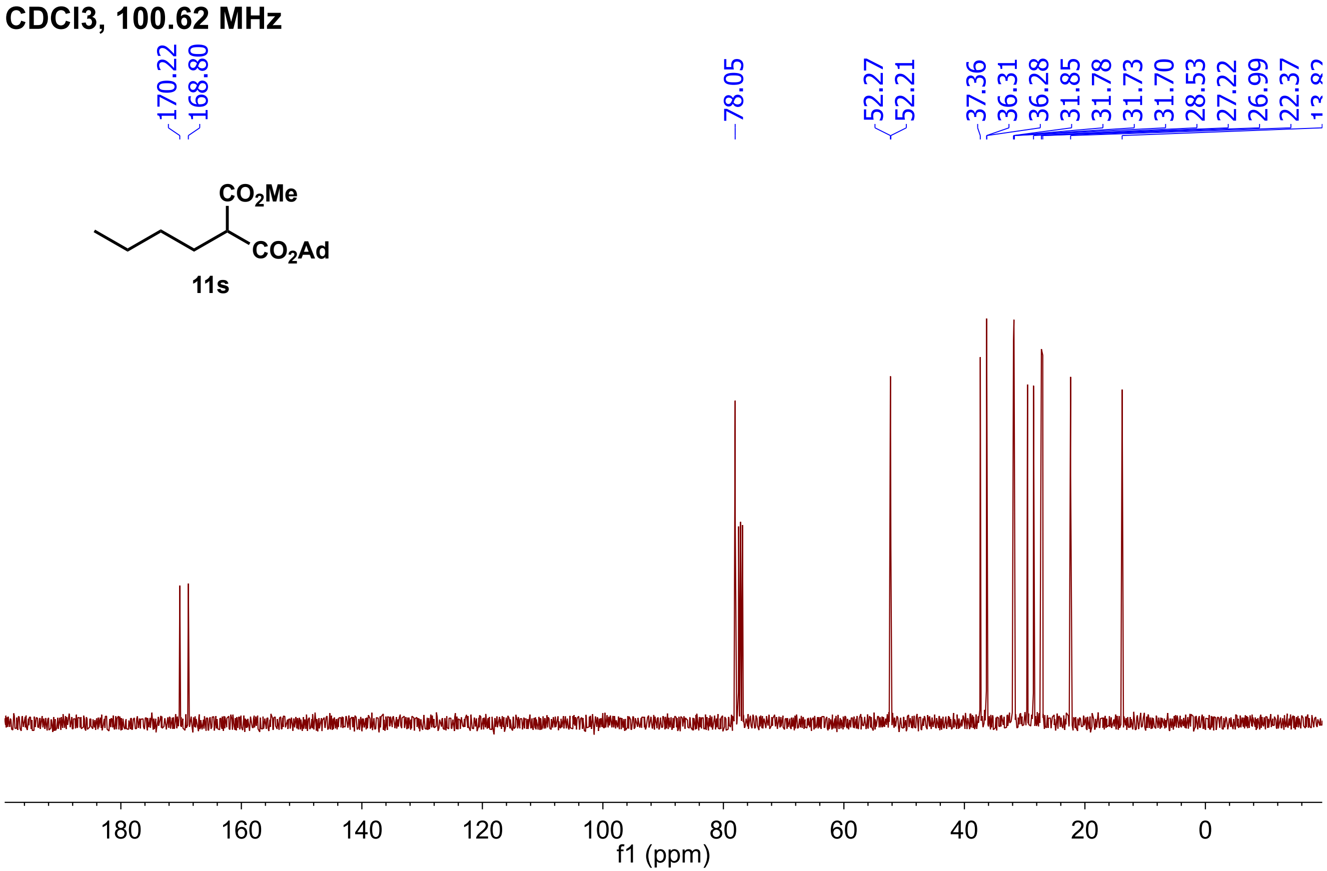

Supplementary Figure 133. ^{13}C NMR (101 MHz, $CDCl_3$) spectra for **11s**

CDCl3, 400.13 MHz

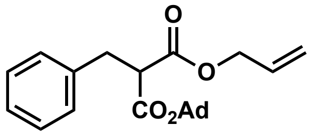

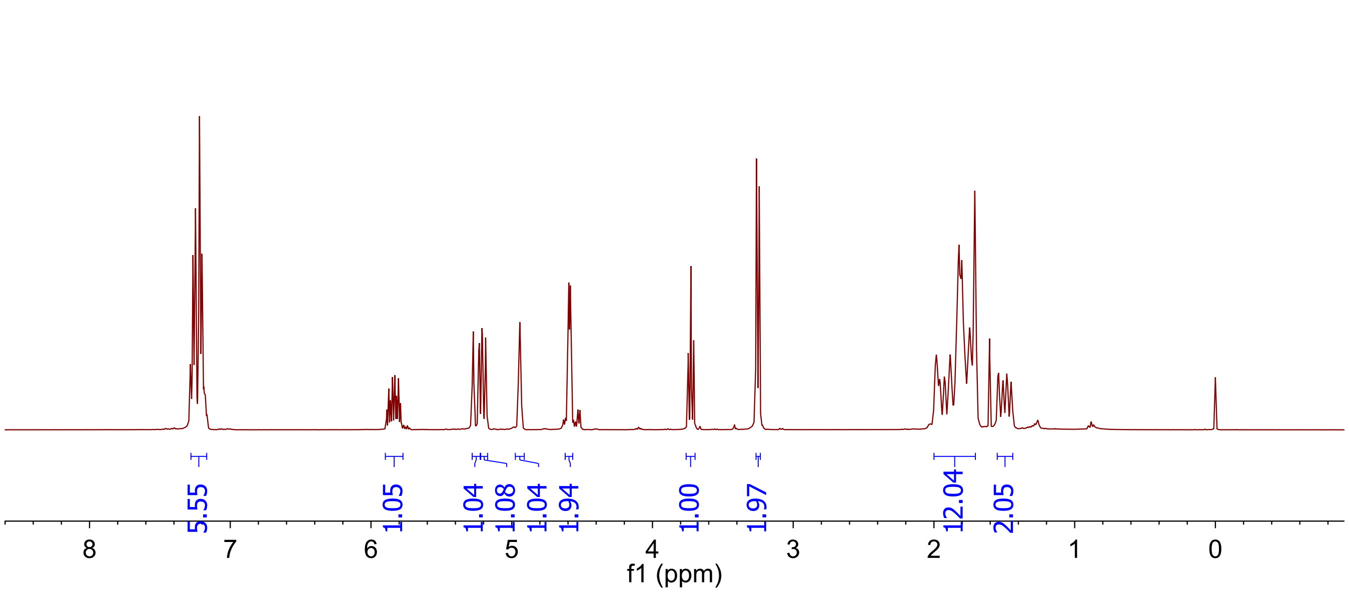

Supplementary Figure 134. ^{1}H NMR (400 MHz, $CDCl_3$) spectra for **11t**

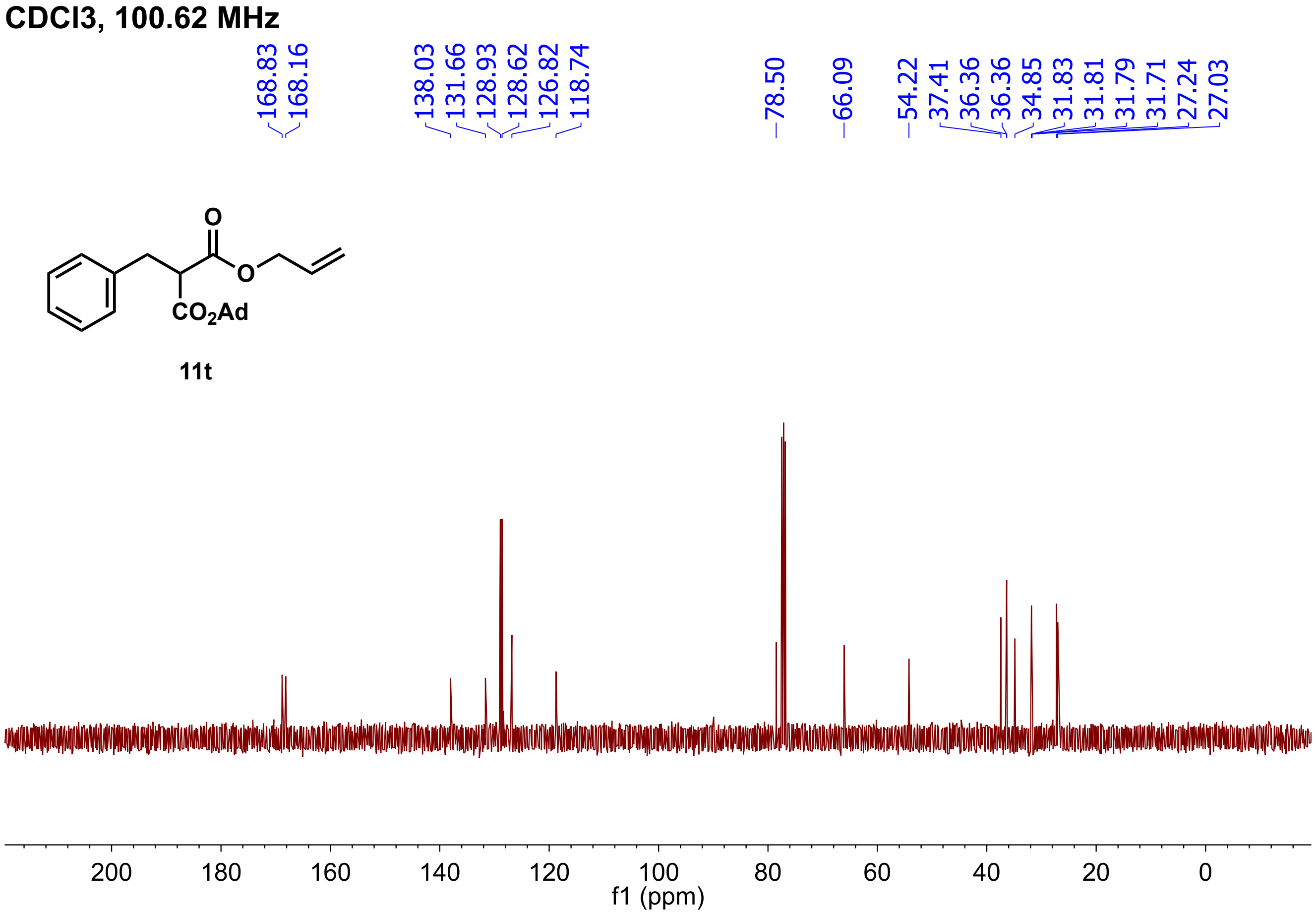

Supplementary Figure 135. ^{13}C NMR (101 MHz, $CDCl_3$) spectra for **11t**

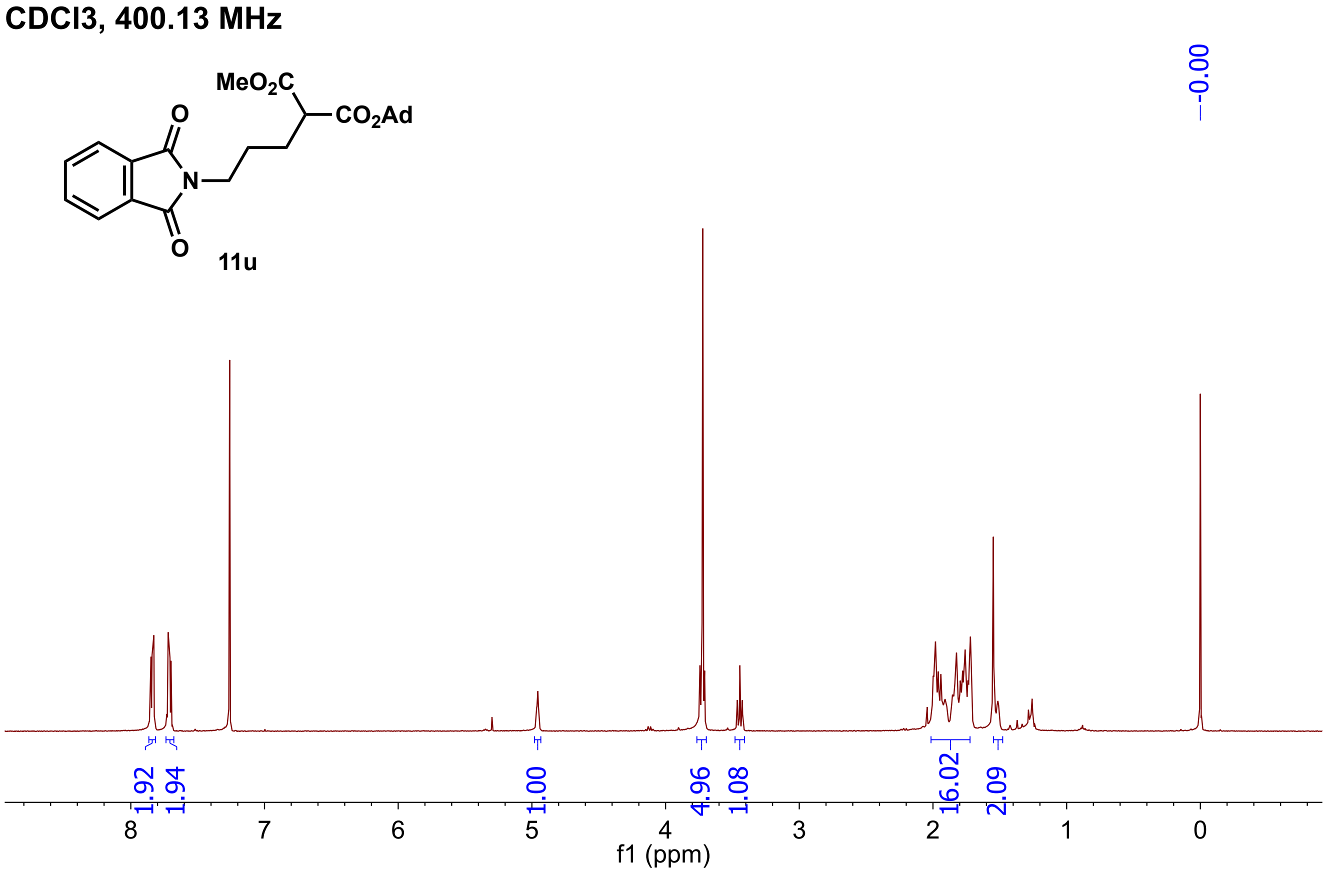

Supplementary Figure 136. ^{1}H NMR (400 MHz, $CDCl_3$) spectra for **11u**

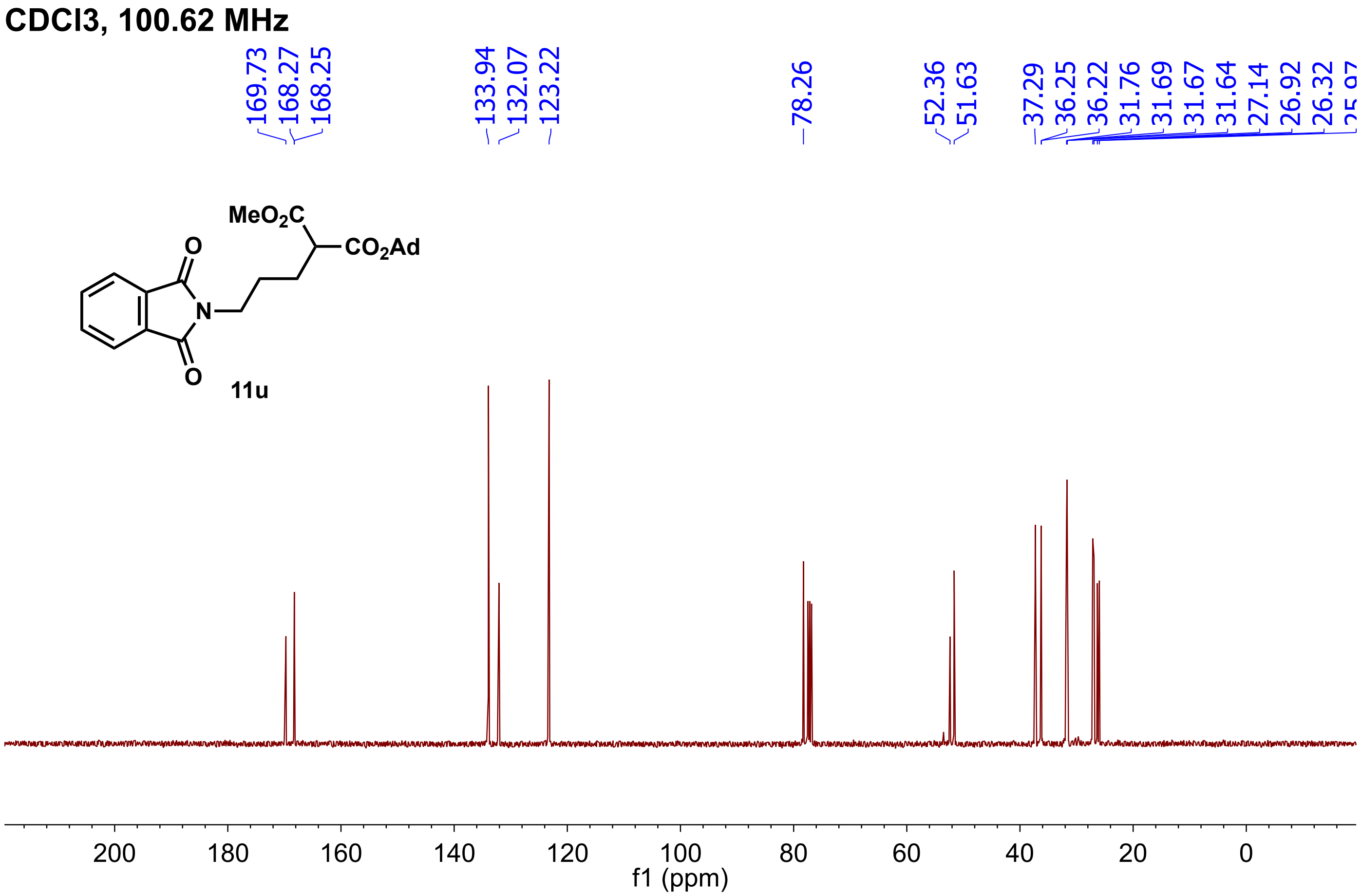

Supplementary Figure 137. ^{13}C NMR (101 MHz, $CDCl_3$) spectra for **11u**

CDCl3, 400.13 MHz

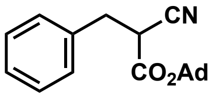

23

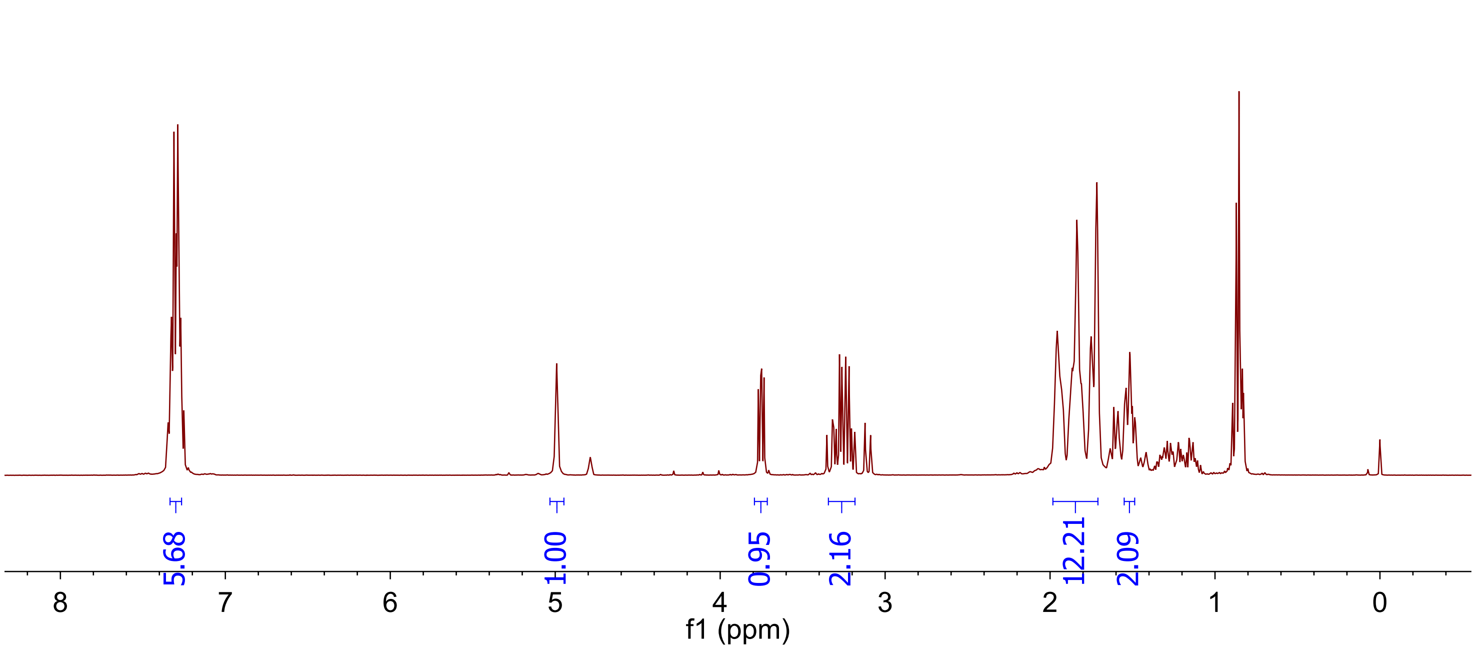

Supplementary Figure 138. ^1H NMR (400 MHz, $CDCl_3$) spectra for **23**

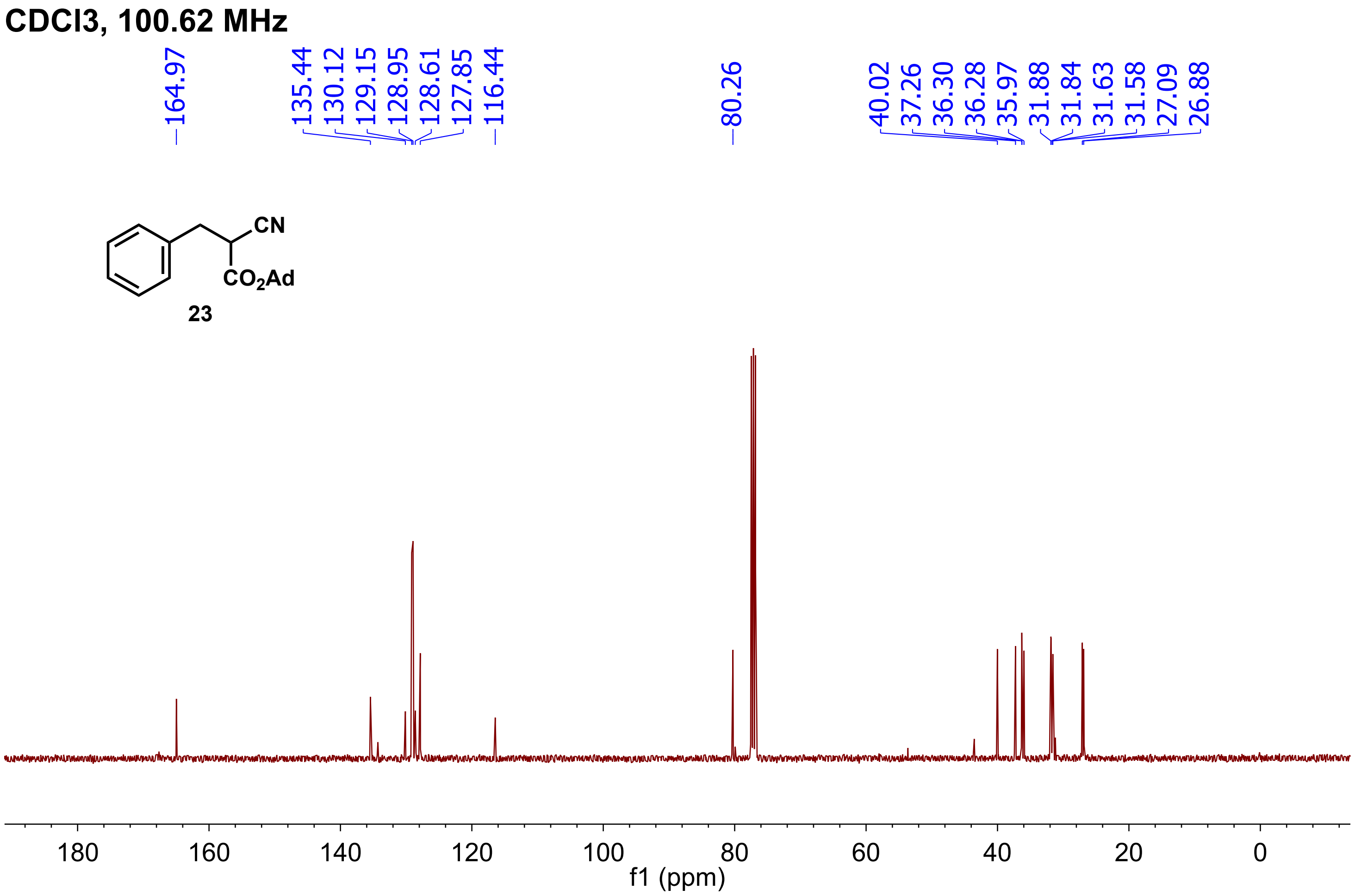

Supplementary Figure 139. ^{13}C NMR (101 MHz, $CDCl_3$) spectra for **23**

CDCl3, 400.13 MHz

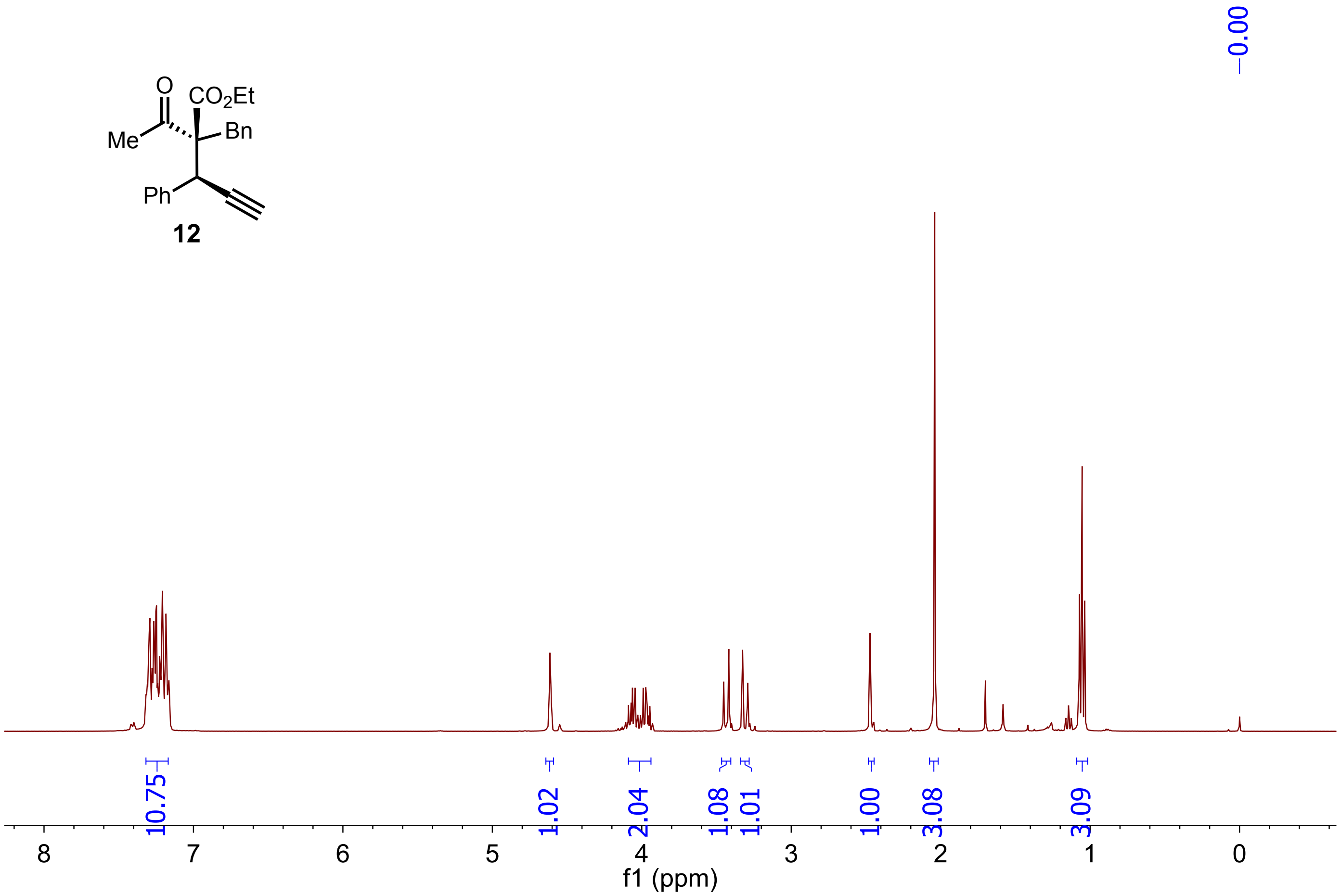

Supplementary Figure 140. ^{1}H NMR (400 MHz, $CDCl_3$) spectra for **12**

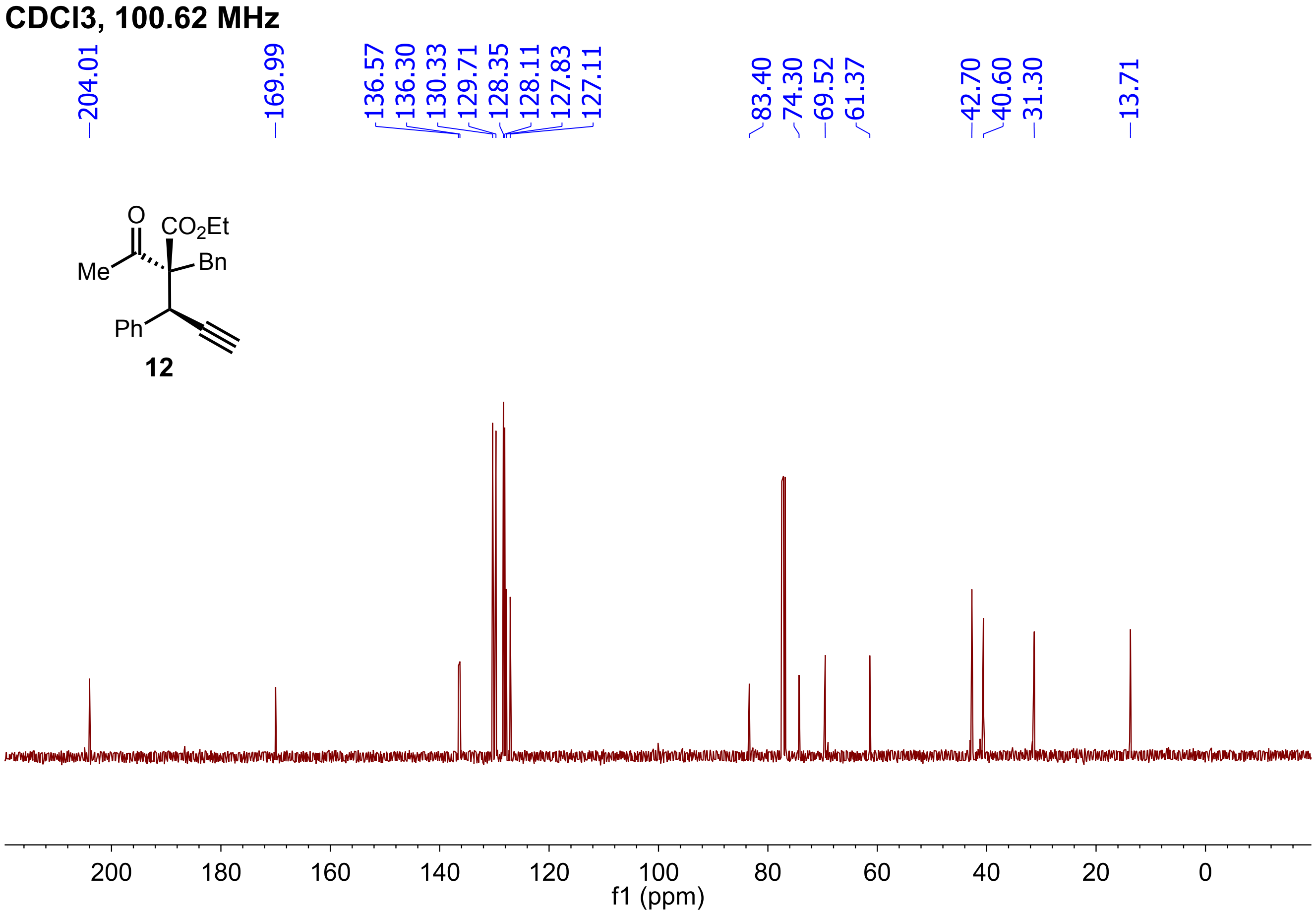

Supplementary Figure 141. ^{13}C NMR (101 MHz, $CDCl_3$) spectra for **12**

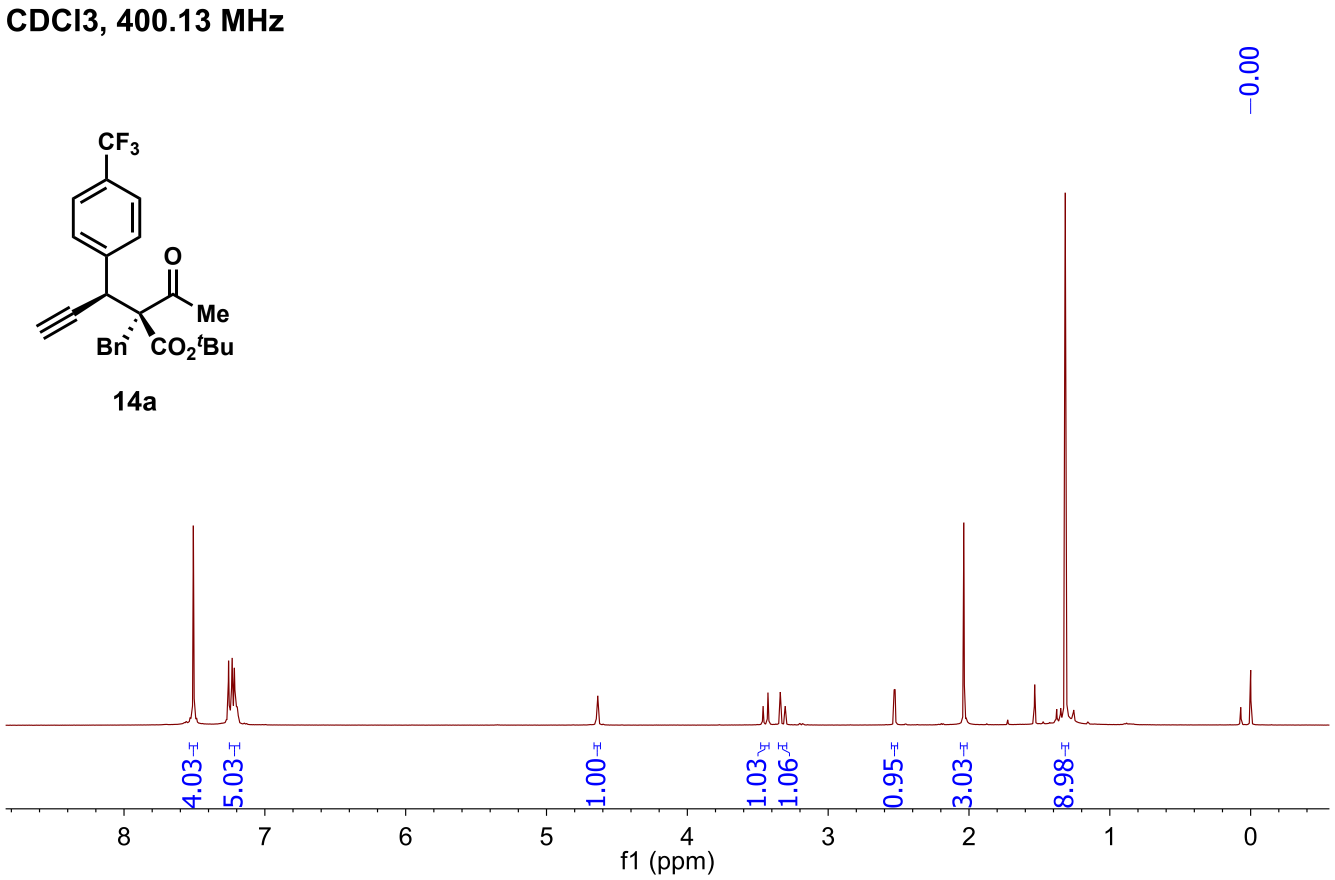

Supplementary Figure 142. ^{1}H NMR (400 MHz, $CDCl_3$) spectra for **14a**

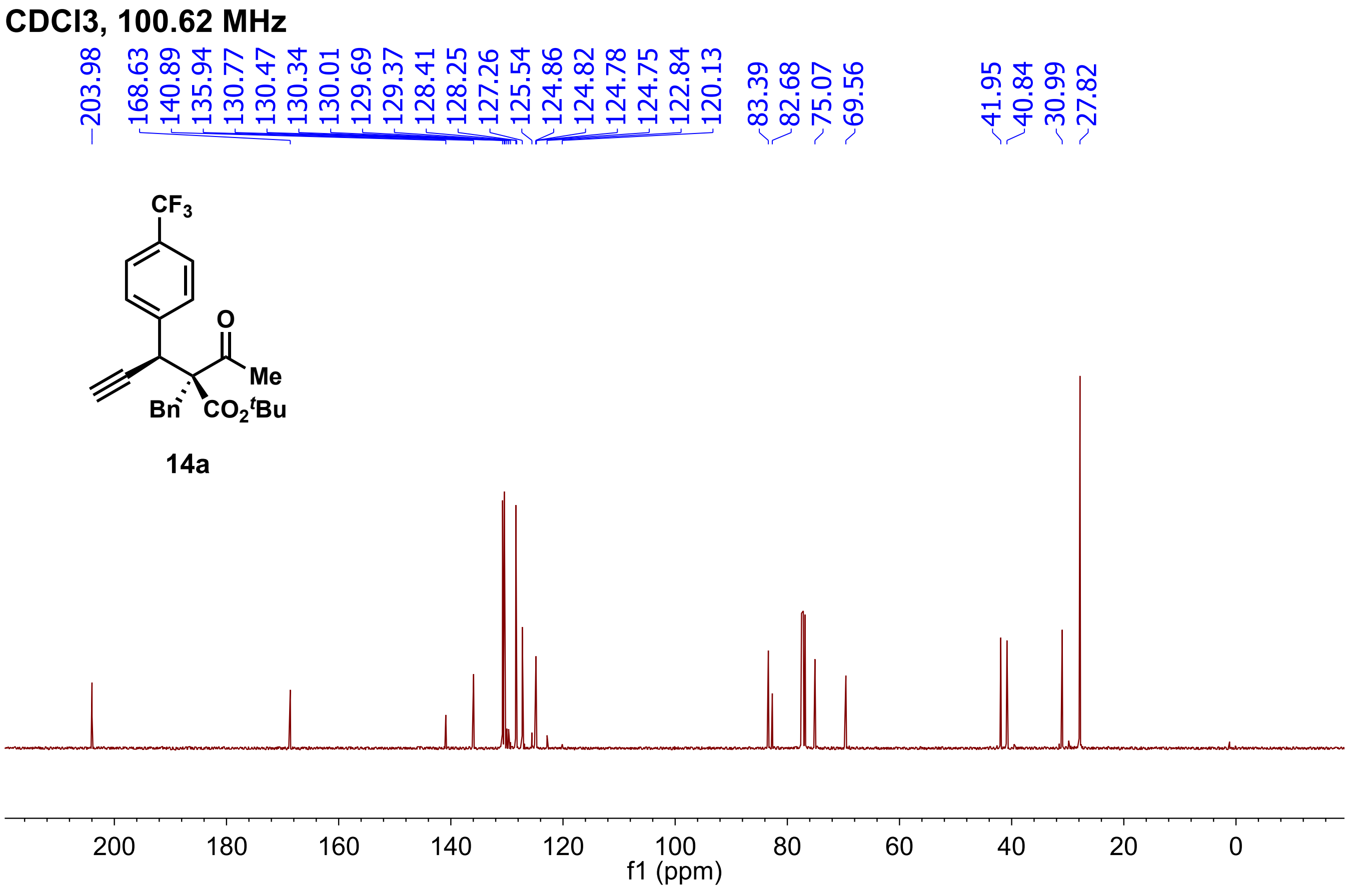

Supplementary Figure 143. ^{13}C NMR (101 MHz, $CDCl_3$) spectra for **14a**

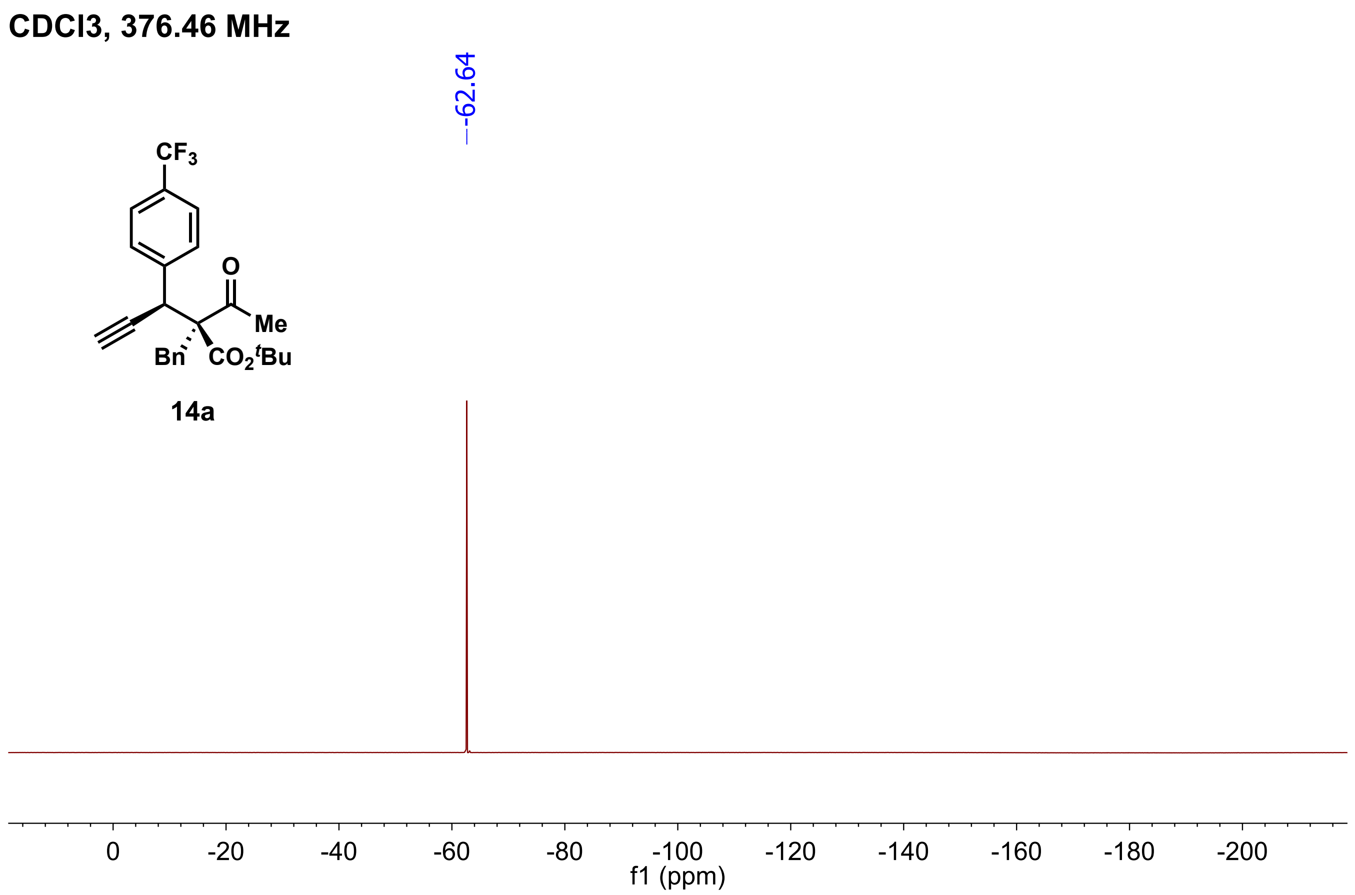

Supplementary Figure 144. ^{19}F NMR (376 MHz, $CDCl_3$) spectra for **14a**

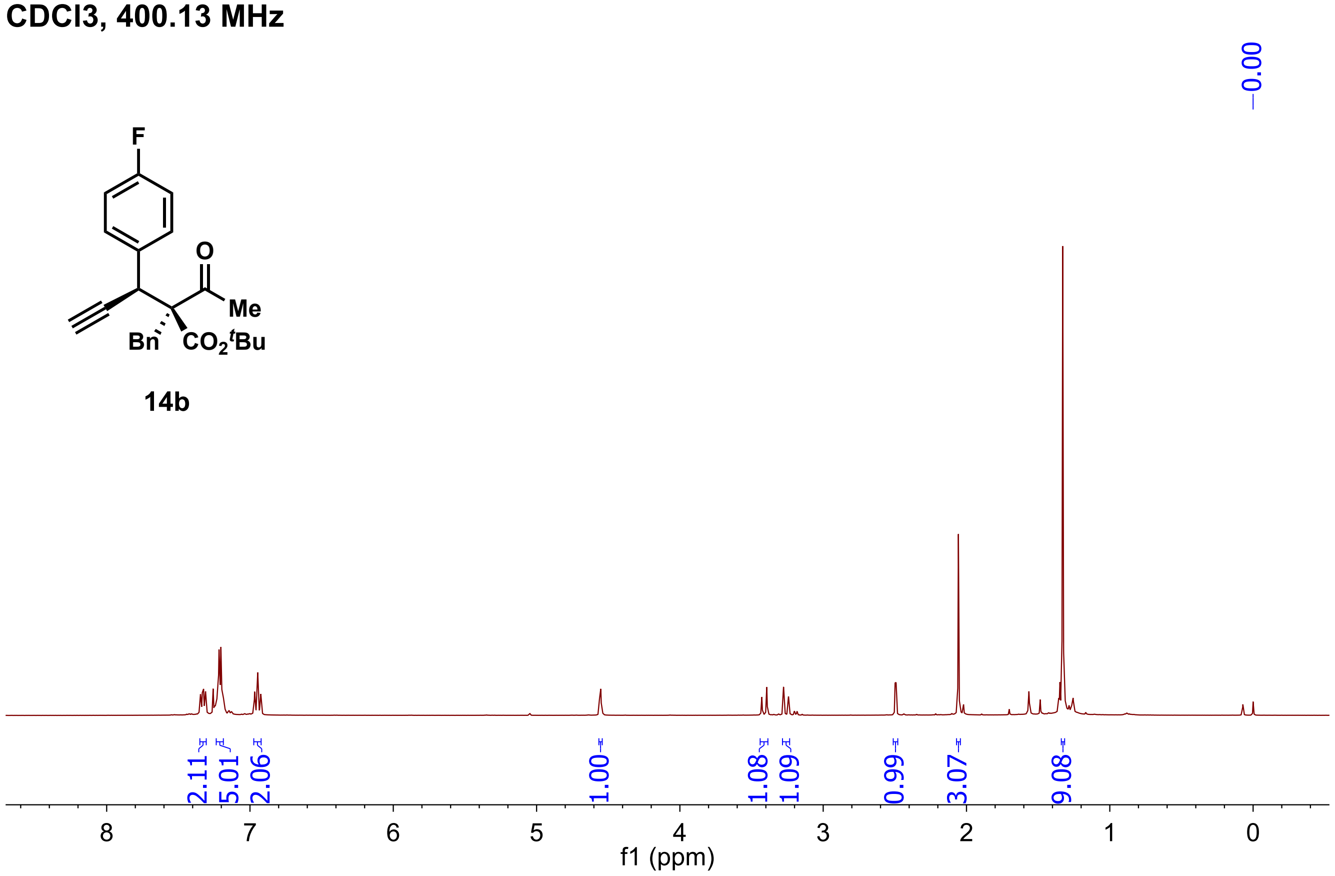

Supplementary Figure 145. ^{1}H NMR (400 MHz, $CDCl_3$) spectra for **16b**

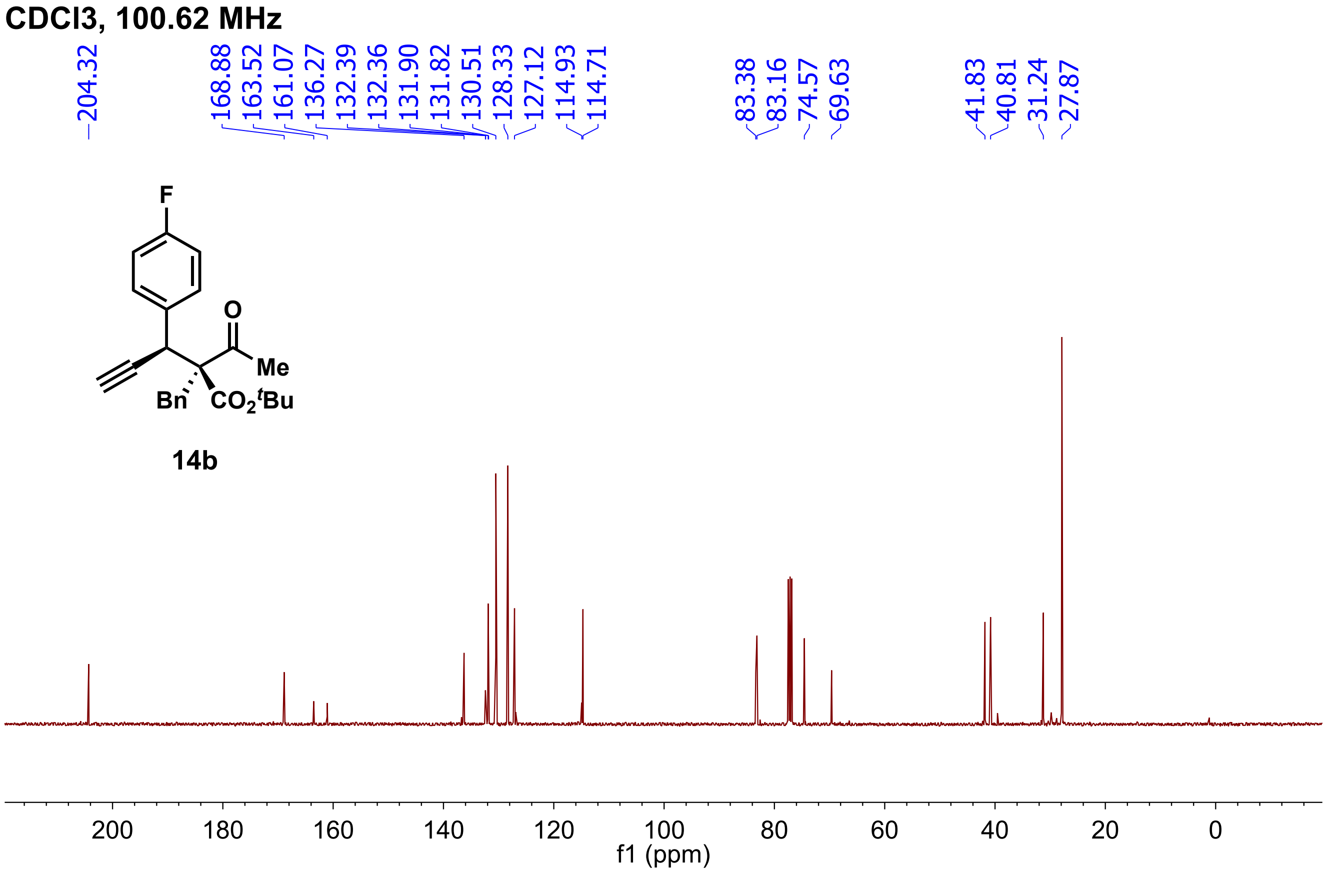

Supplementary Figure 146. ^{13}C NMR (101 MHz, $CDCl_3$) spectra for **14b**

CDCl3, 376.46 MHz

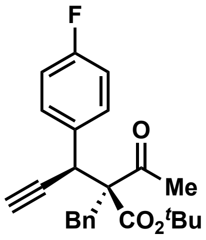

14b

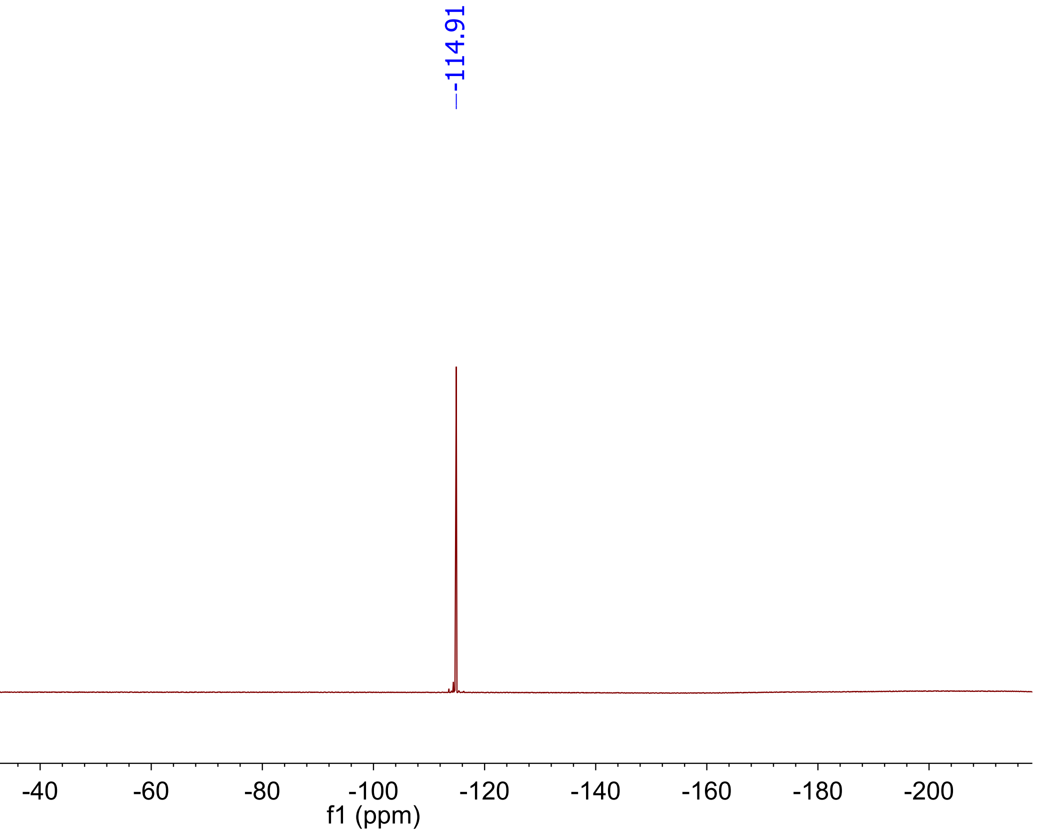

Supplementary Figure 147. ^{19}F NMR (376 MHz, $CDCl_3$) spectra for **14b**

CDCl3, 400.13 MHz

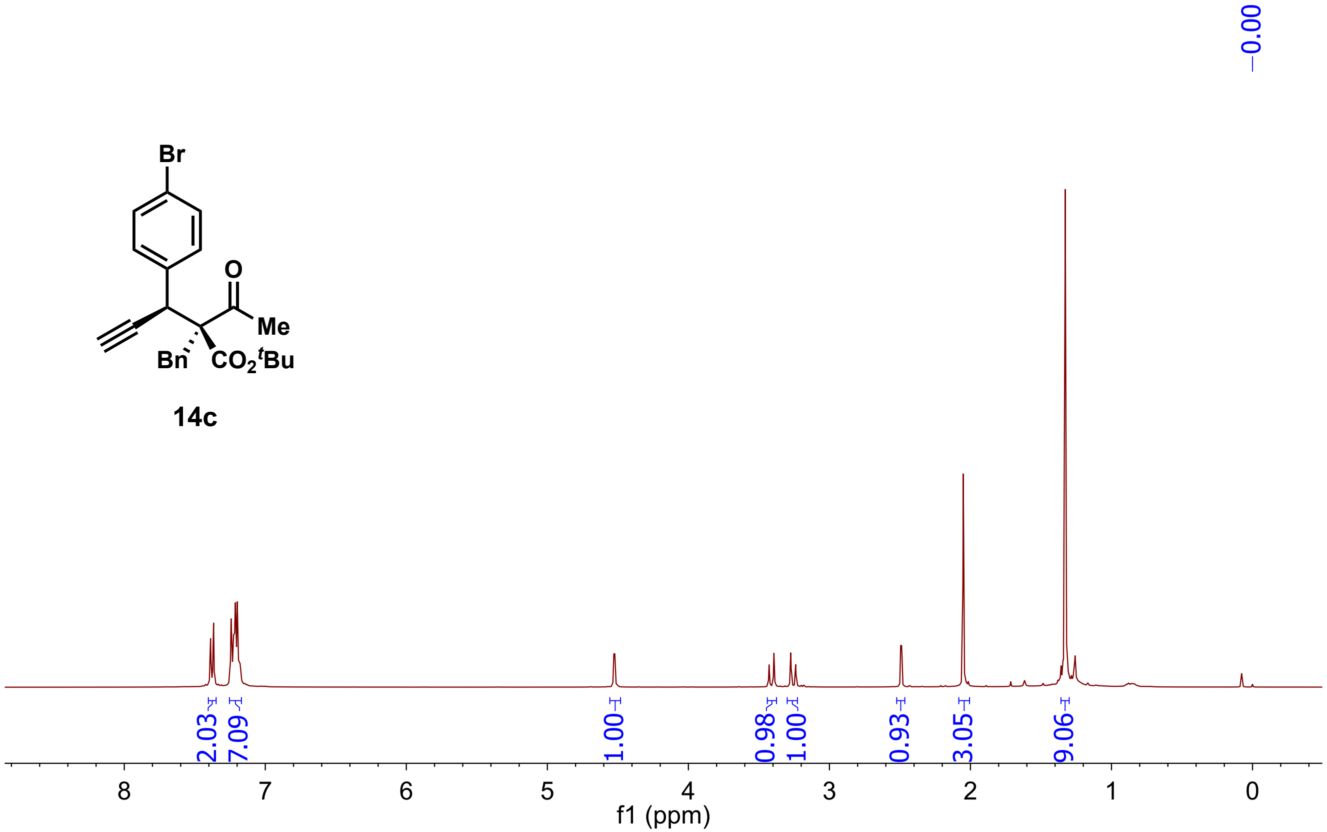

Supplementary Figure 148. ^{1}H NMR (400 MHz, $CDCl_3$) spectra for **14c**

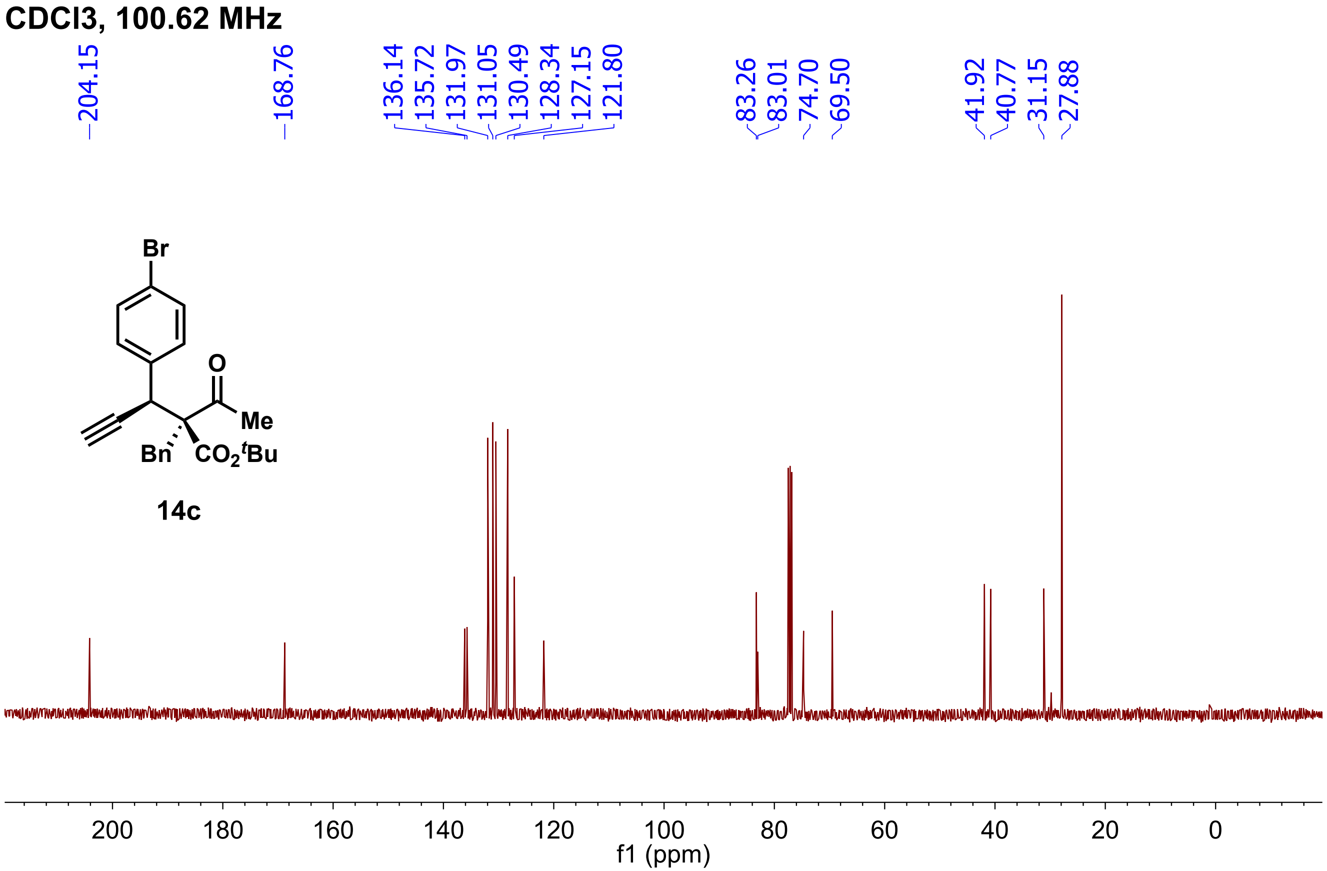

Supplementary Figure 149. ^{13}C NMR (101 MHz, $CDCl_3$) spectra for **14c**

CDCl3, 400.13 MHz

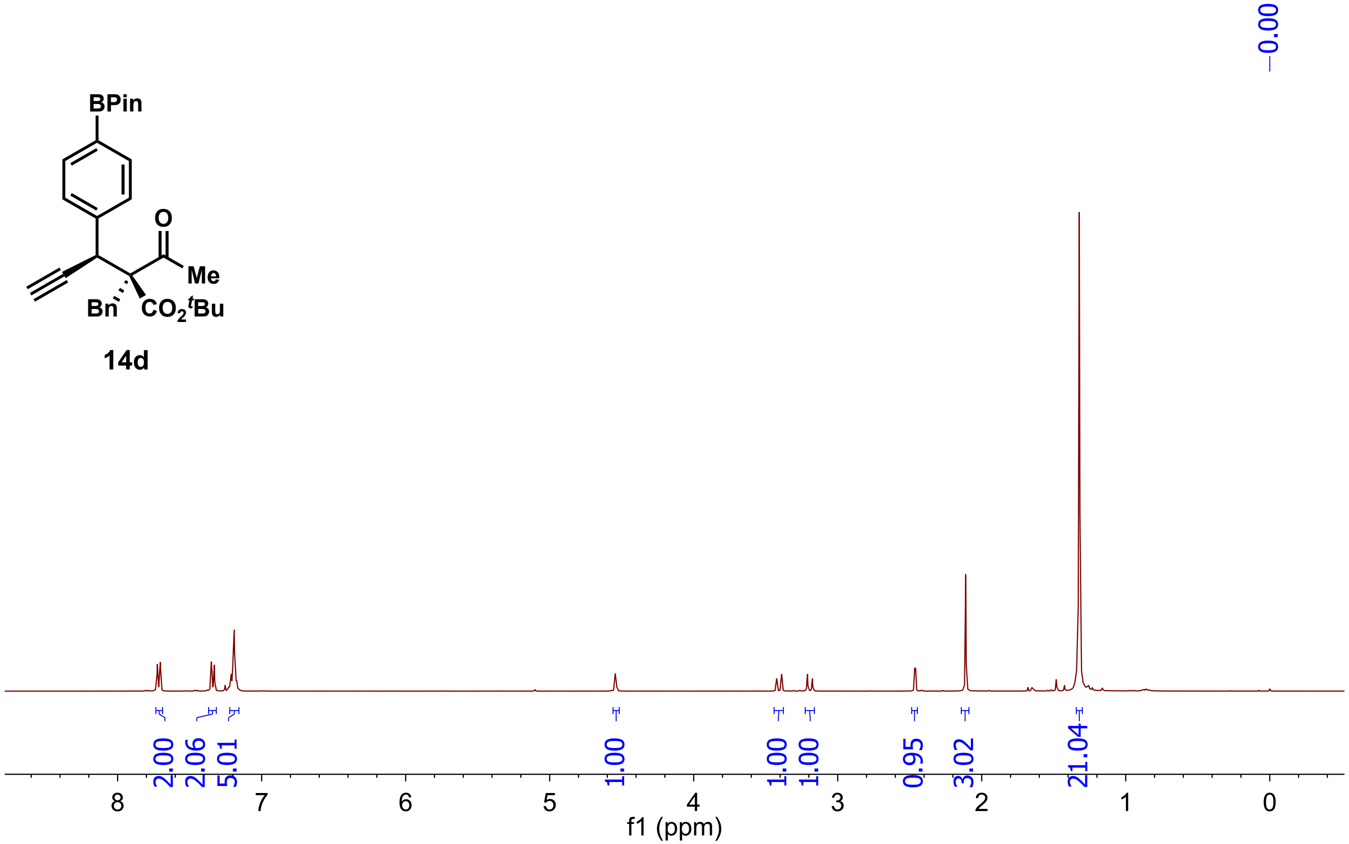

Supplementary Figure 150. ^{1}H NMR (400 MHz, $CDCl_3$) spectra for **14d**

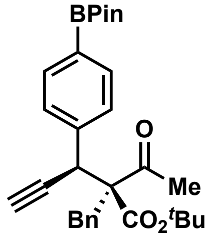

14d

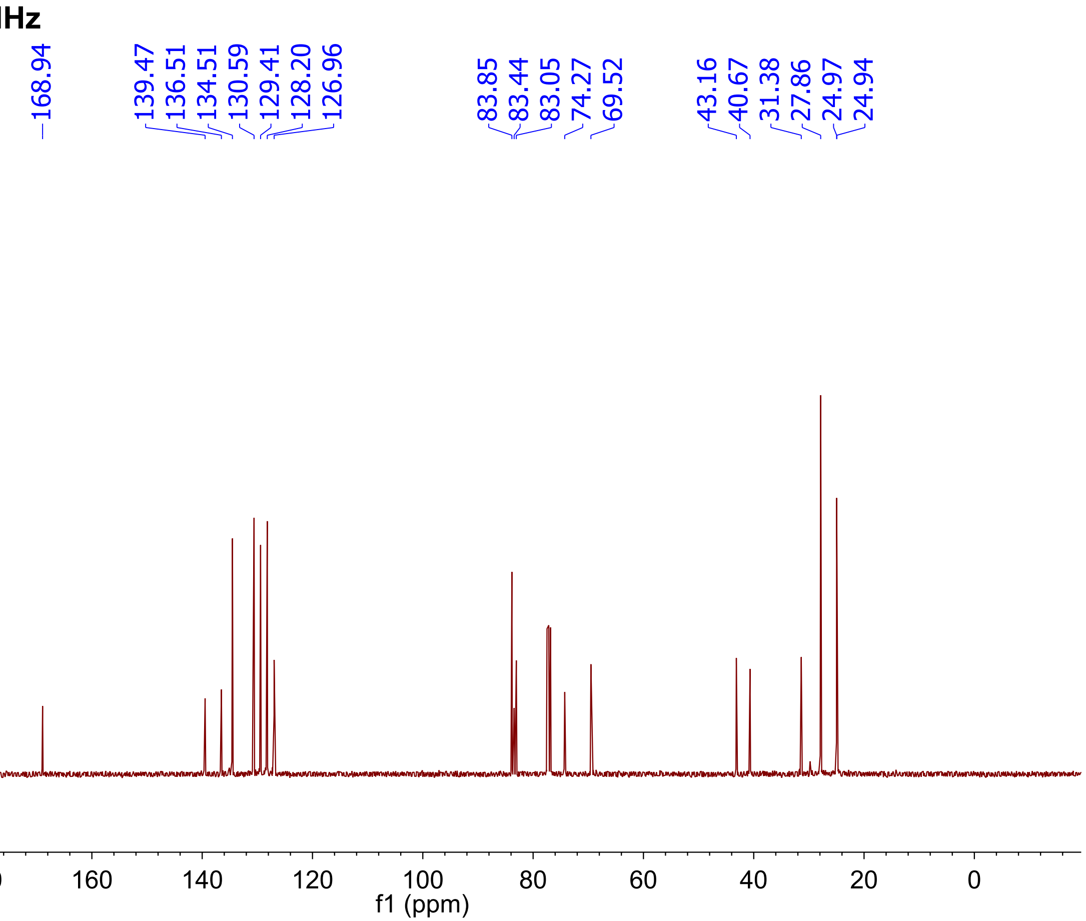

Supplementary Figure 151. ^{13}C NMR (101 MHz, $CDCl_3$) spectra for **14d**

CDCl3, 400.13 MHz

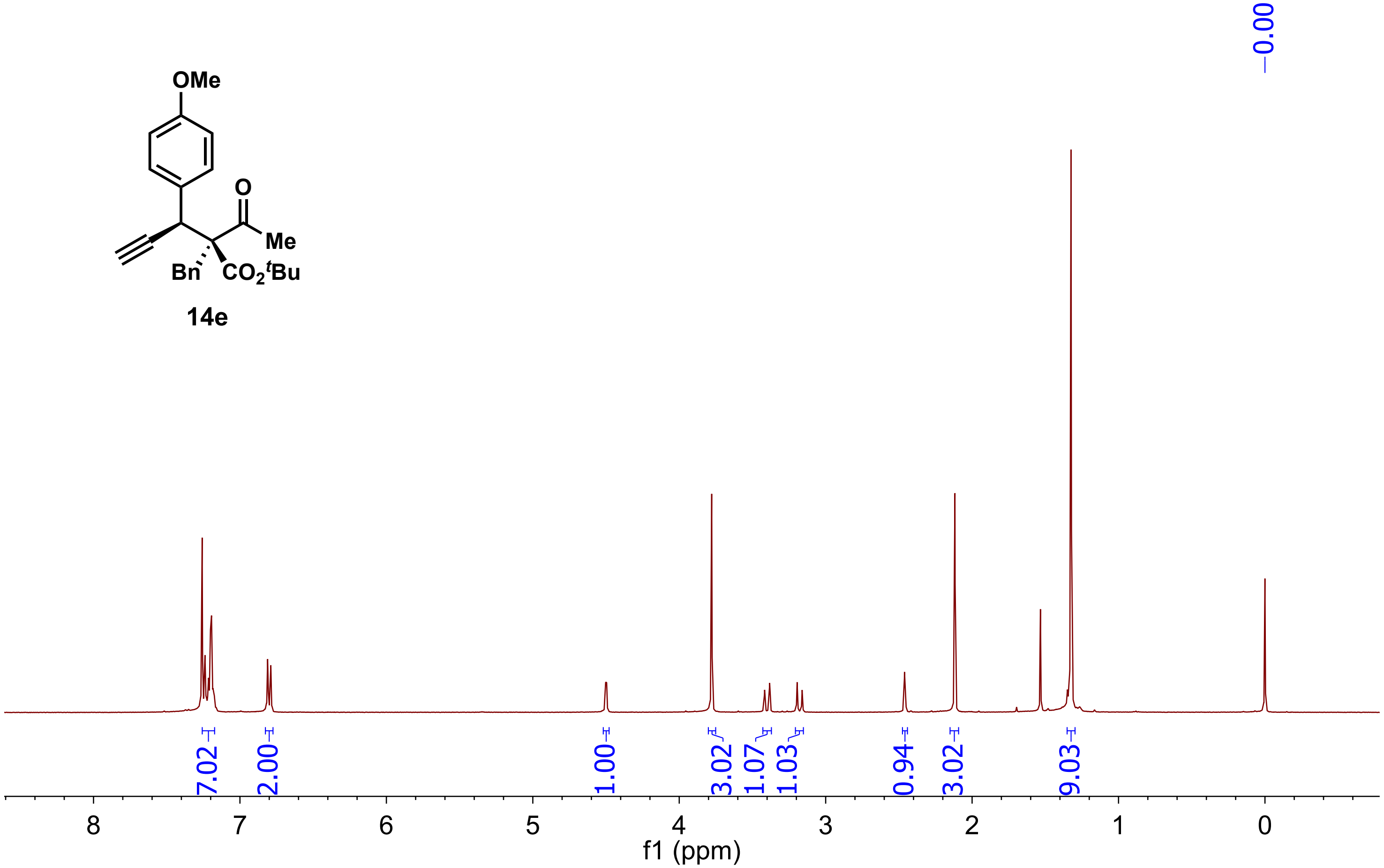

Supplementary Figure 152. ^{1}H NMR (400 MHz, $CDCl_3$) spectra for **14e**

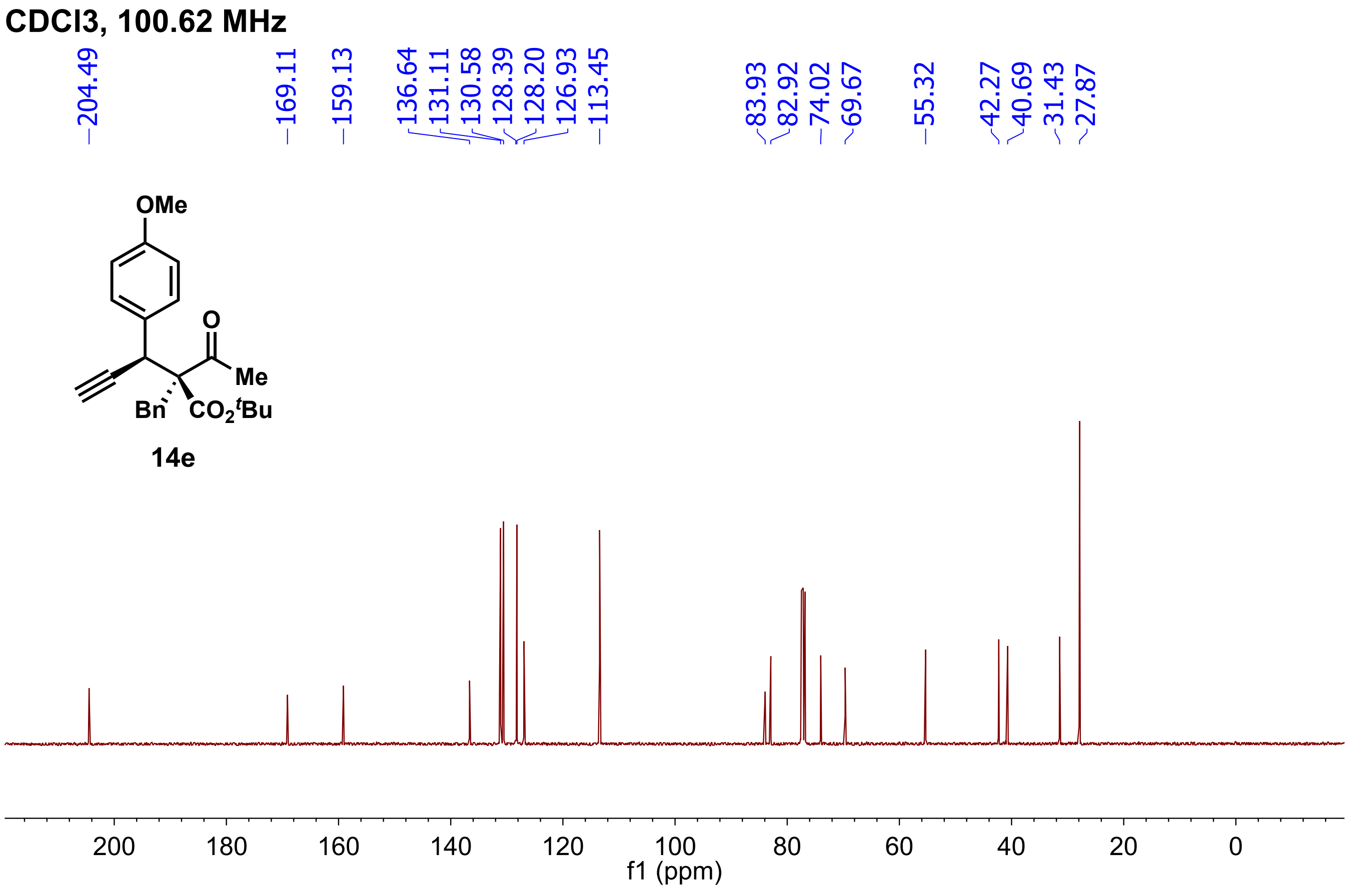

Supplementary Figure 153. ^{13}C NMR (101 MHz, $CDCl_3$) spectra for **14e**

CDCl3, 400.13 MHz

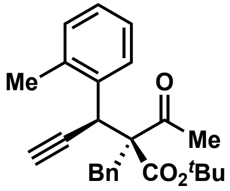

14f

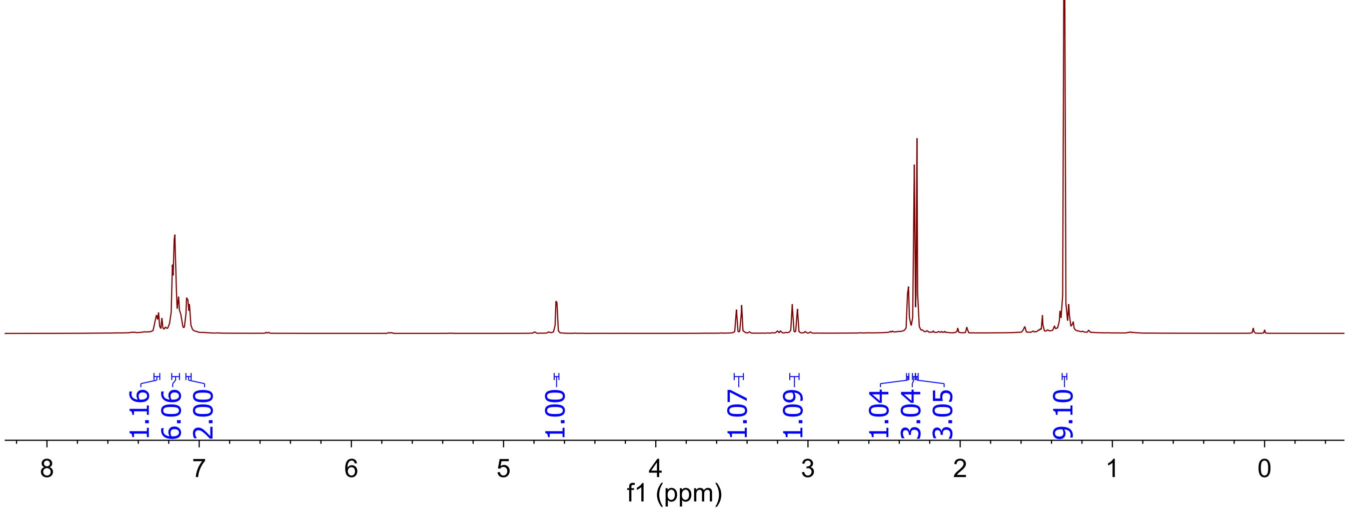

Supplementary Figure 154. ^{1}H NMR (400 MHz, $CDCl_3$) spectra for **14f**

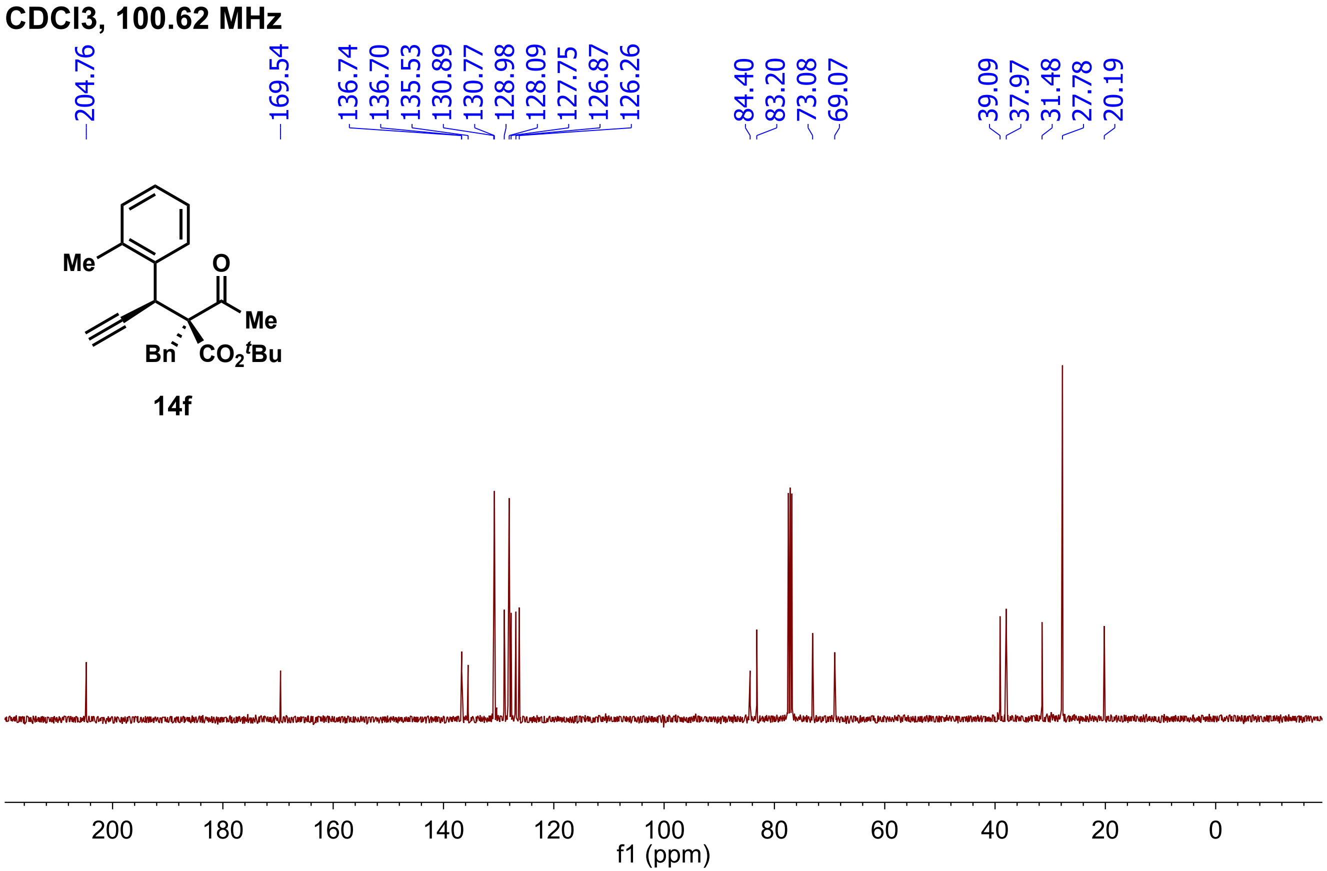

Supplementary Figure 155. ^{13}C NMR (101 MHz, $CDCl_3$) spectra for **14f**

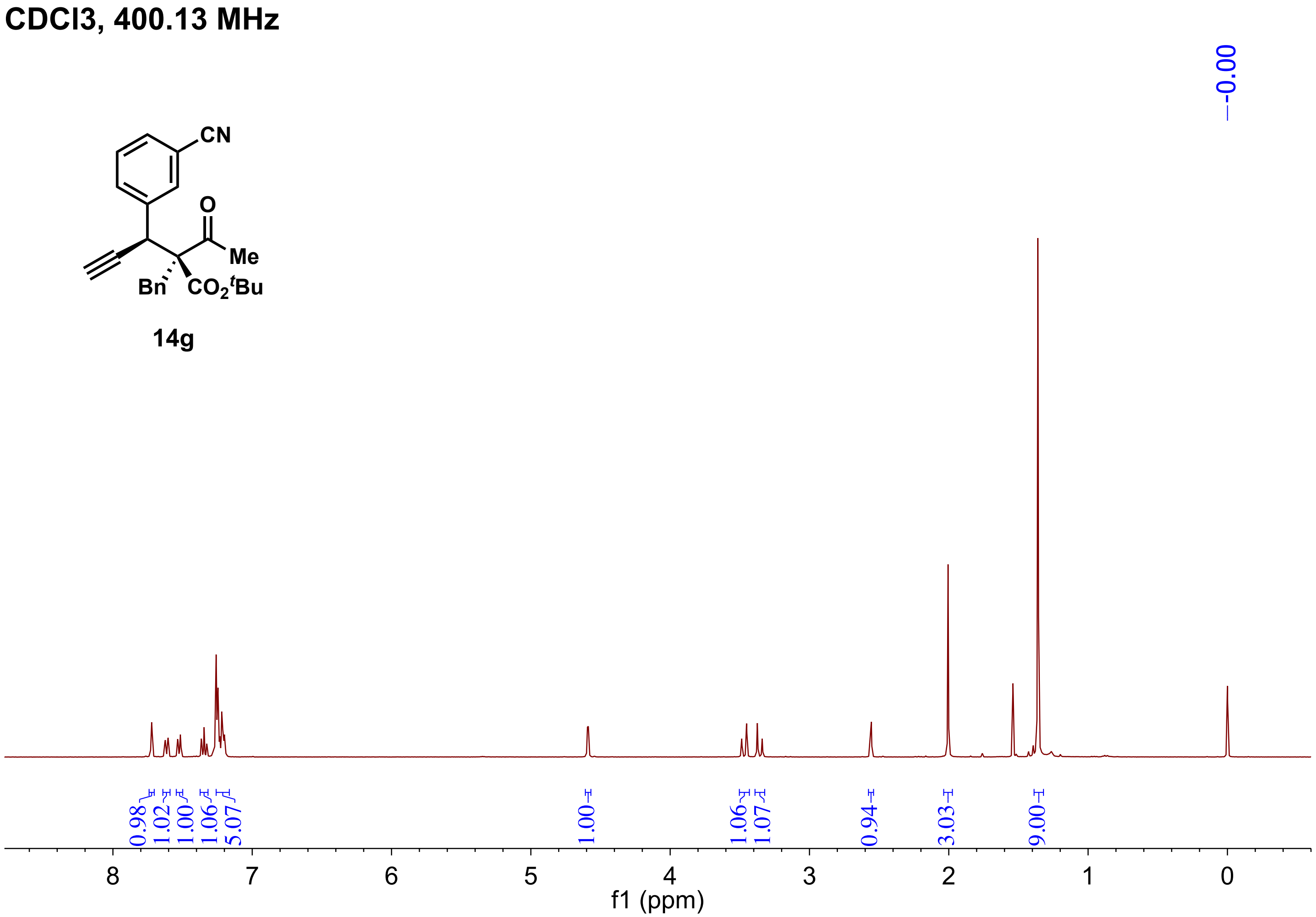

Supplementary Figure 156. ^{1}H NMR (400 MHz, $CDCl_3$) spectra for **14g**

CDCl3, 100.62 MHz

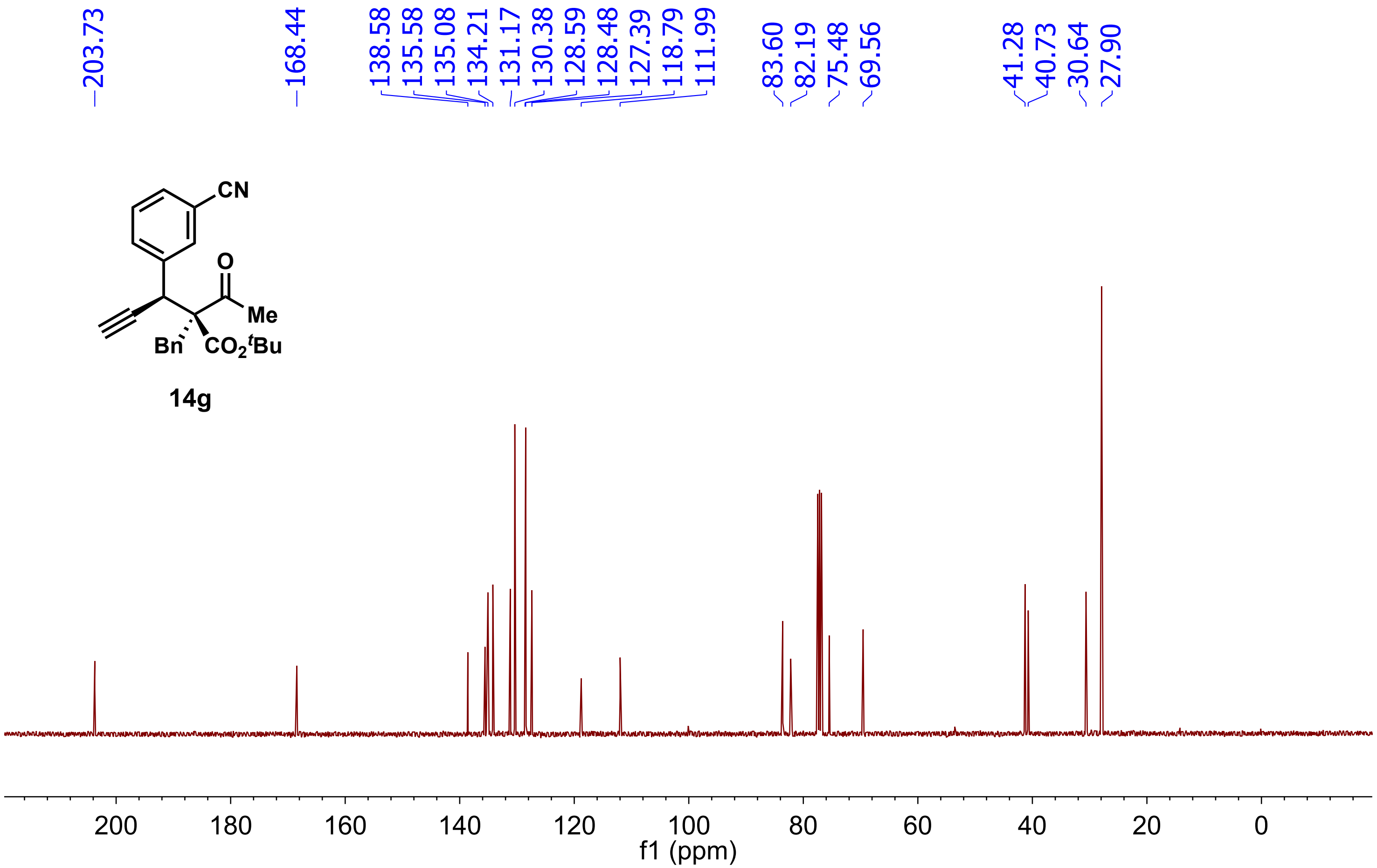

14g

Supplementary Figure 157. ^{13}C NMR (101 MHz, $CDCl_3$) spectra for **14g**

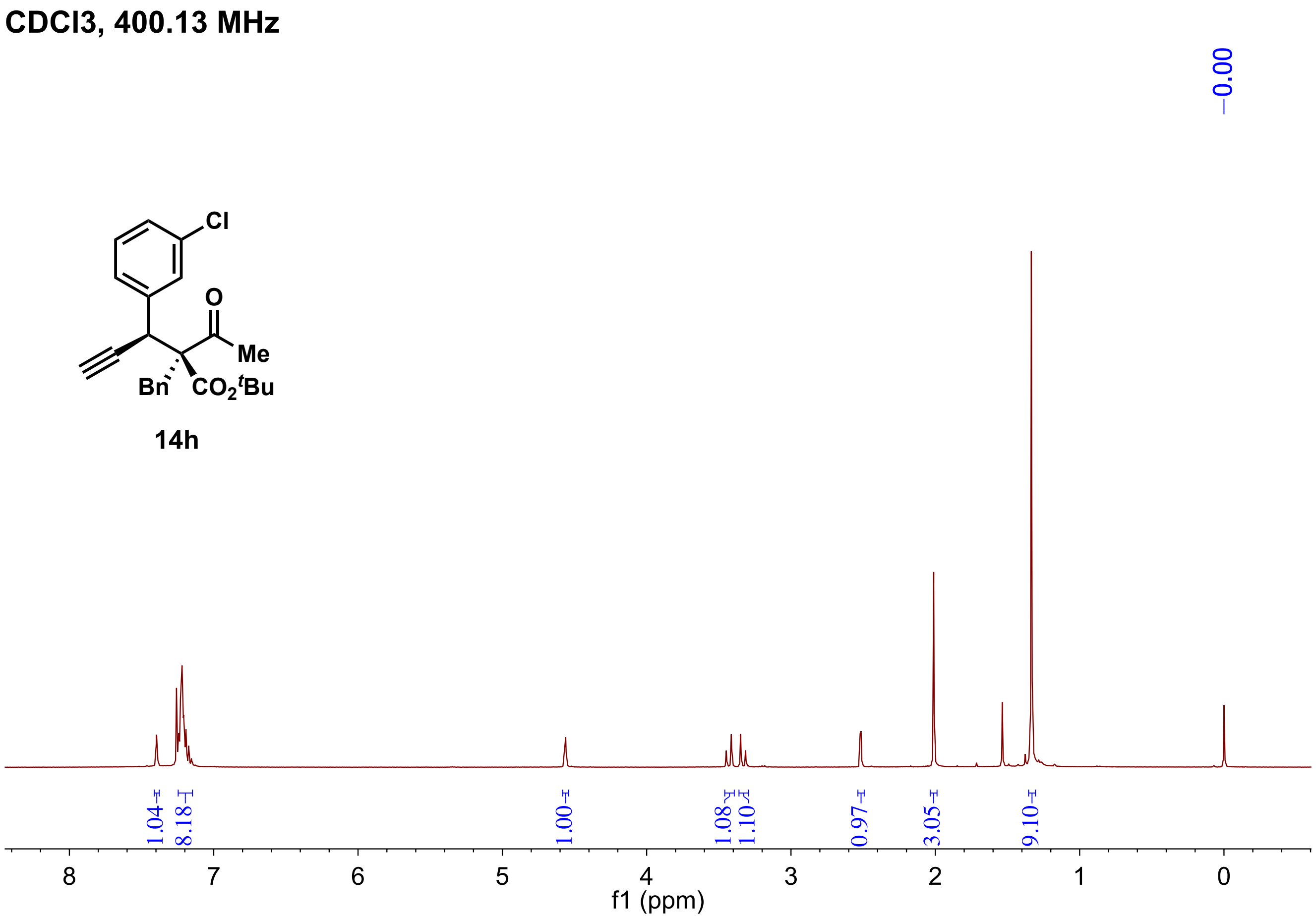

Supplementary Figure 158. ^{1}H NMR (400 MHz, $CDCl_3$) spectra for **14h**

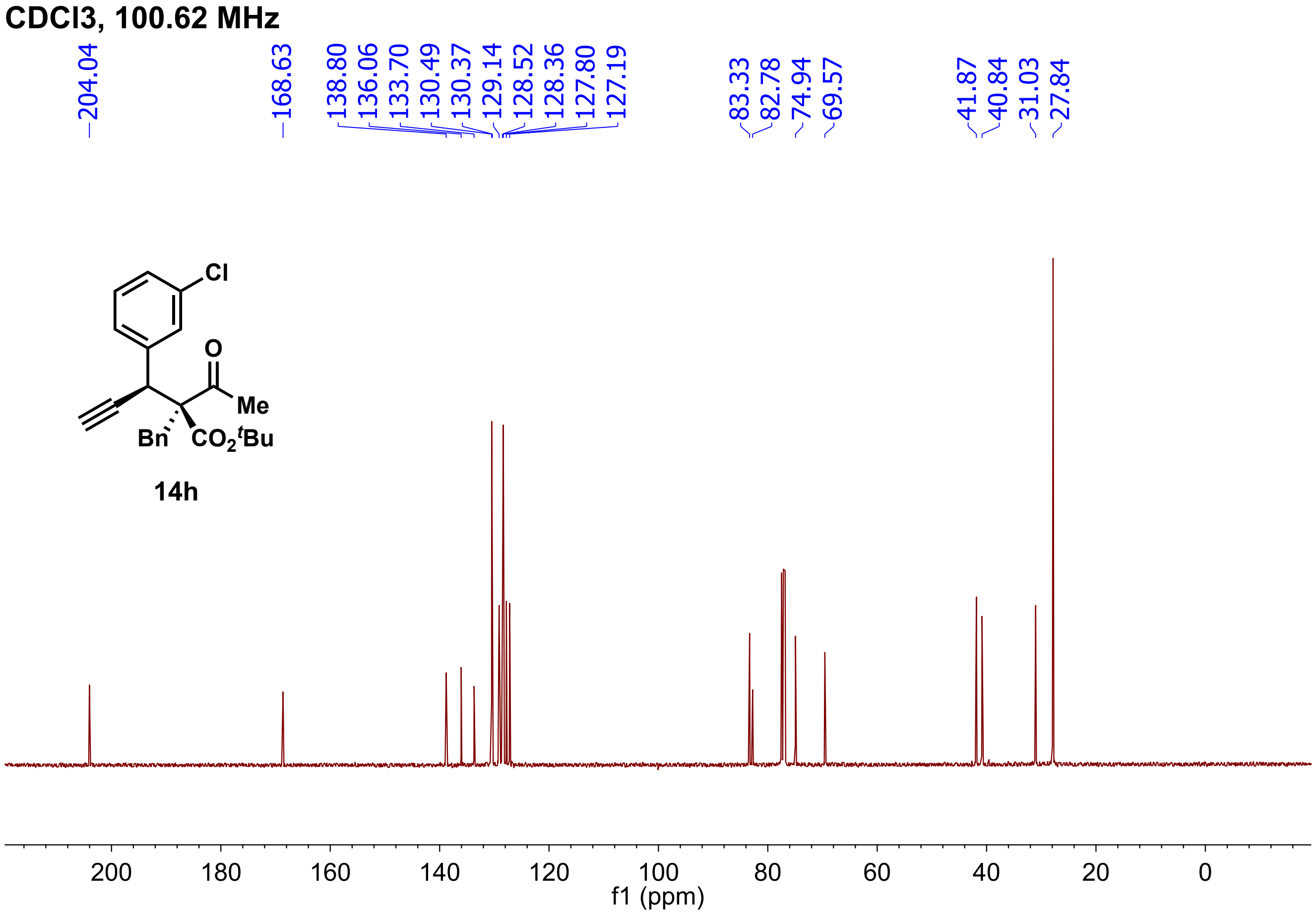

Supplementary Figure 159. ^{13}C NMR (101 MHz, $CDCl_3$) spectra for **14h**

CDCl3, 400.13 MHz

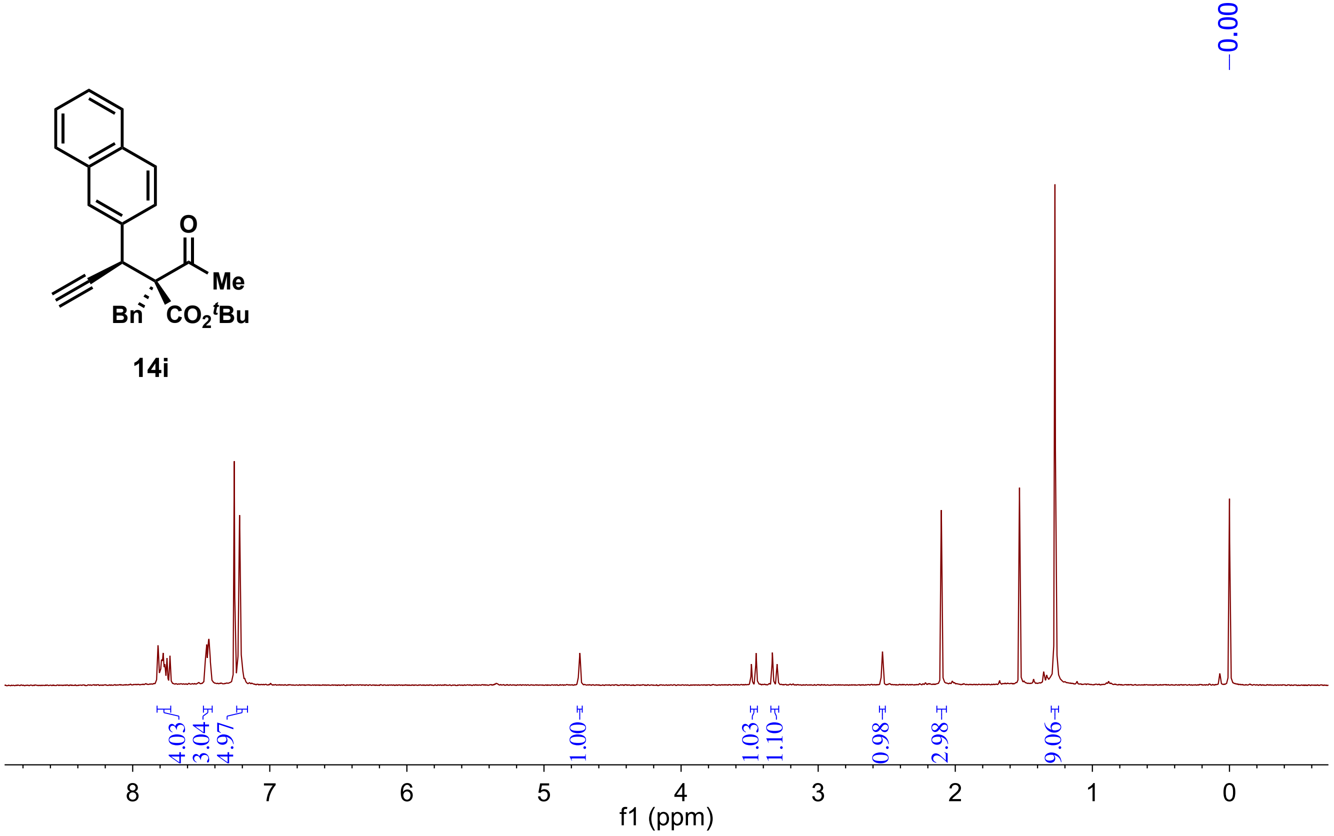

Supplementary Figure 160. ^{1}H NMR (400 MHz, $CDCl_3$) spectra for **14i**

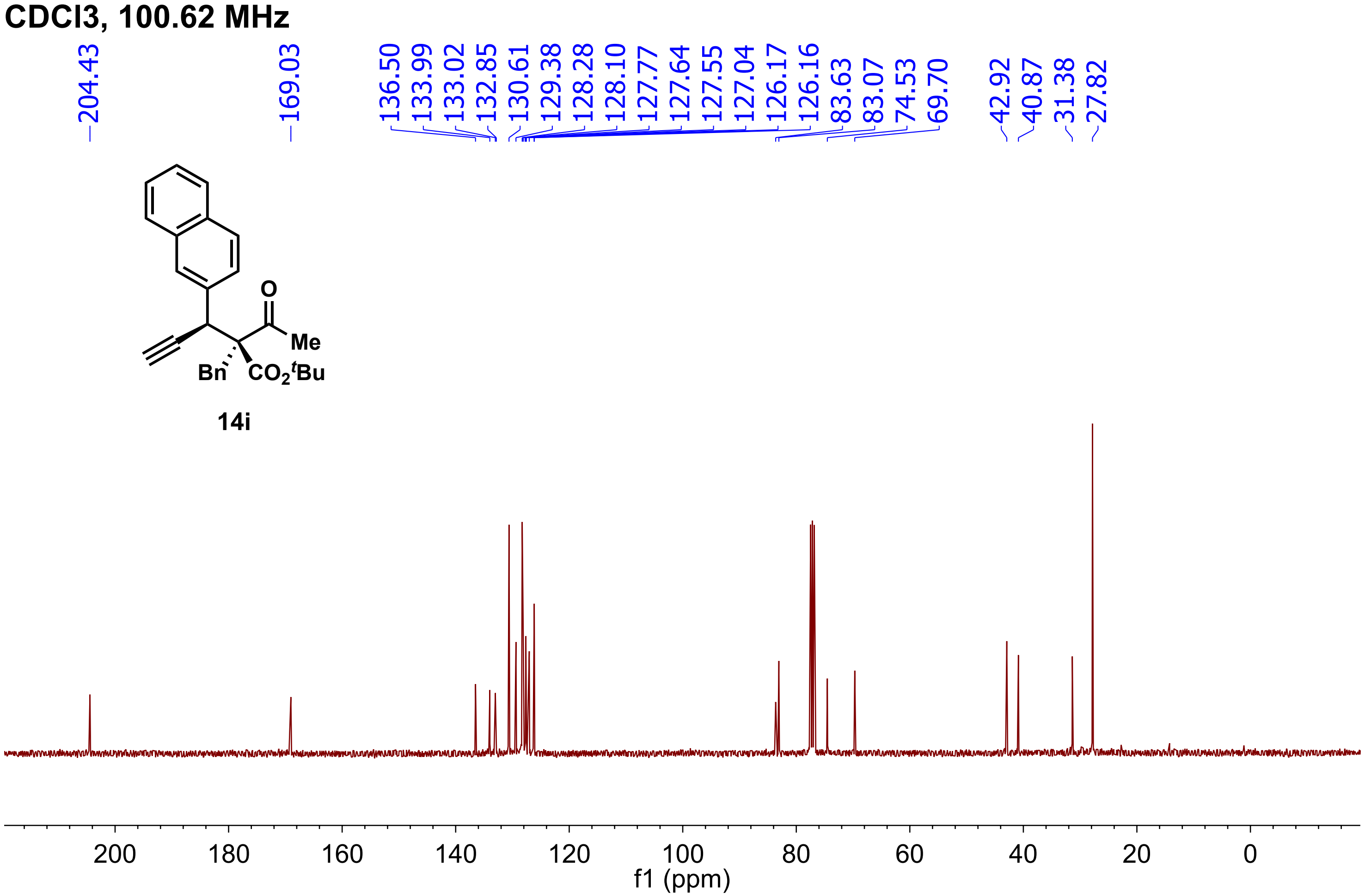

Supplementary Figure 161. ^{13}C NMR (101 MHz, $CDCl_3$) spectra for **14i**

CDCl3, 400.13 MHz

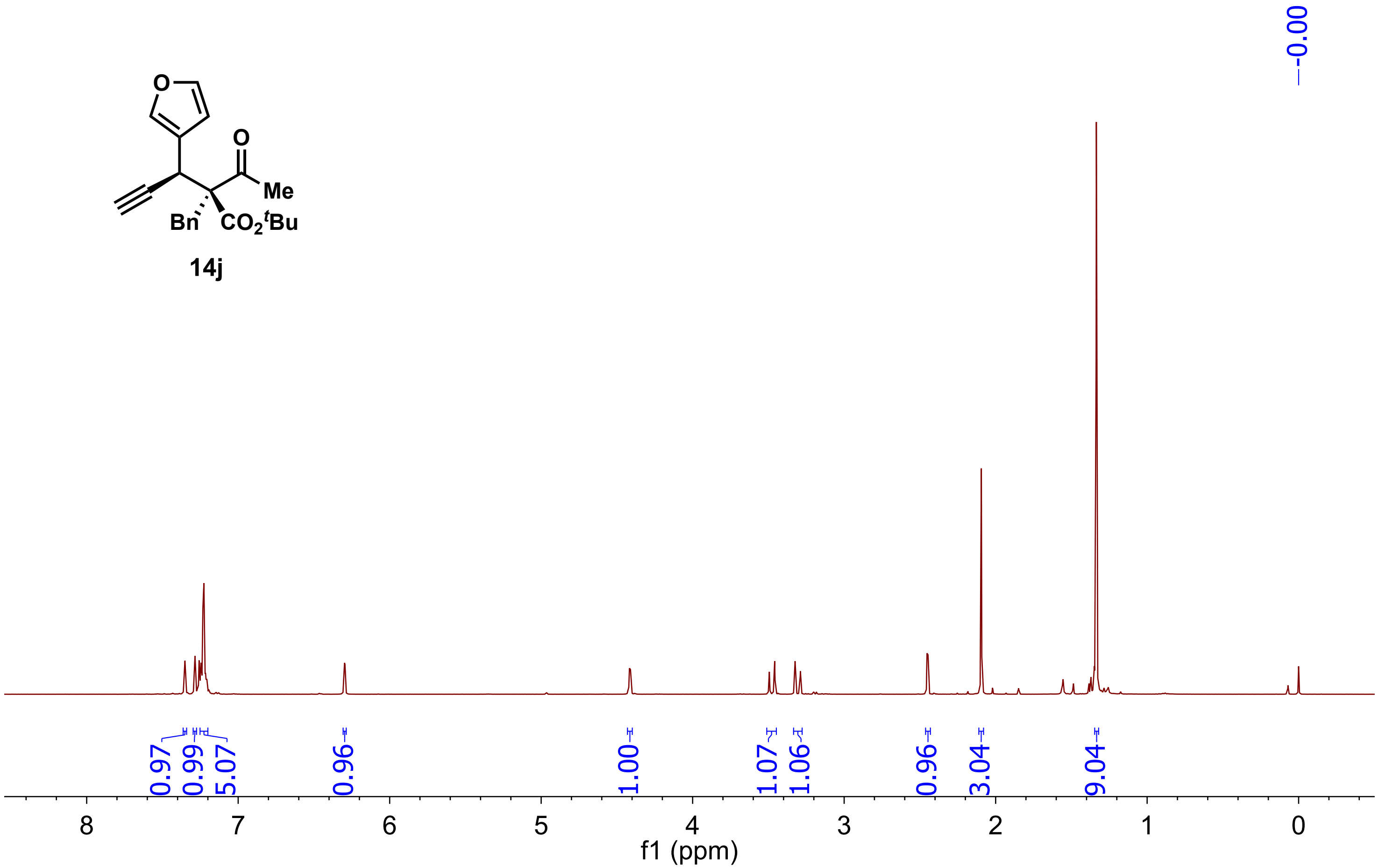

Supplementary Figure 162. ^{1}H NMR (400 MHz, $CDCl_3$) spectra for **14j**

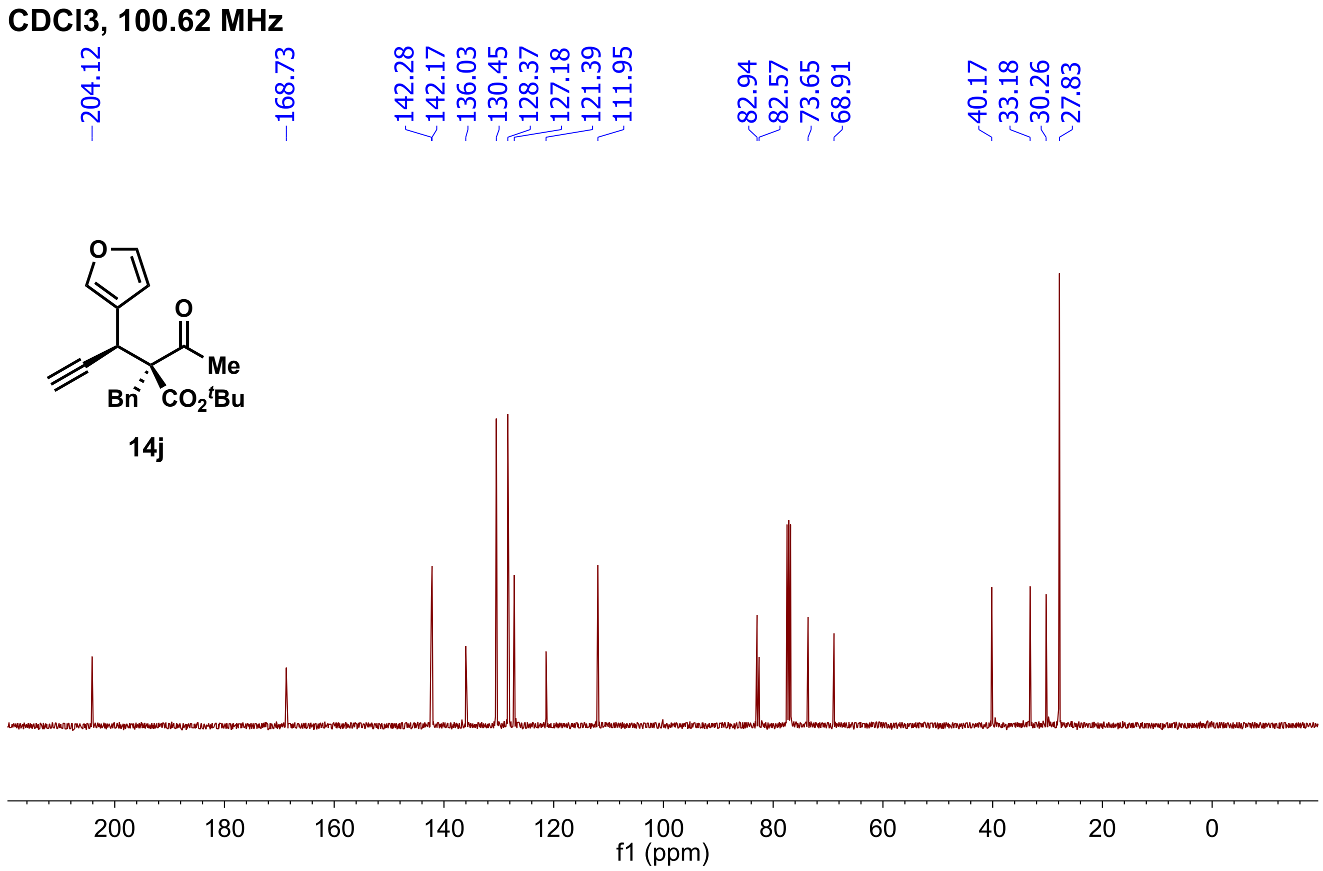

Supplementary Figure 163. ^{13}C NMR (101 MHz, $CDCl_3$) spectra for **14j**

CDCl3, 400.13 MHz

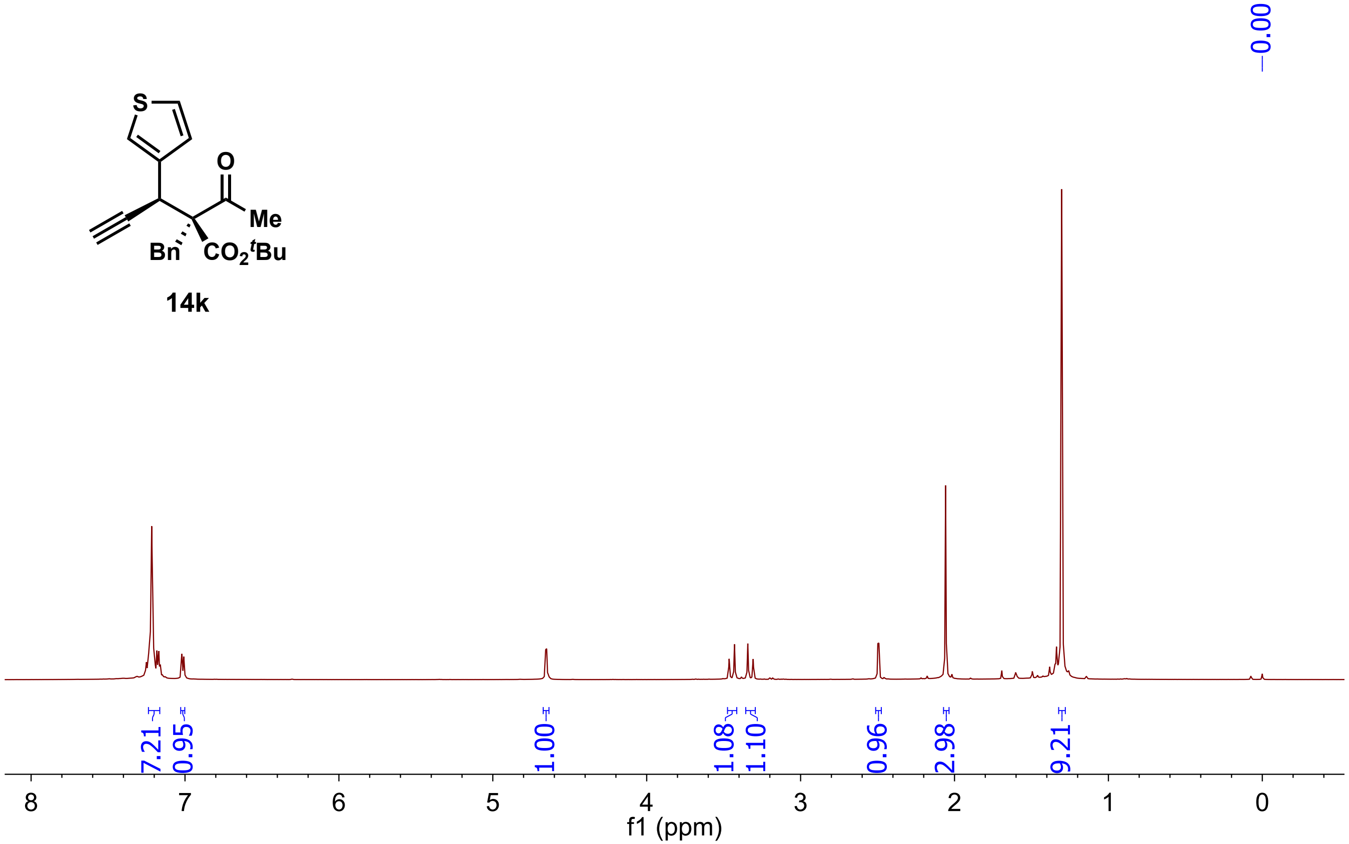

Supplementary Figure 164. ^{1}H NMR (400 MHz, $CDCl_3$) spectra for **14k**

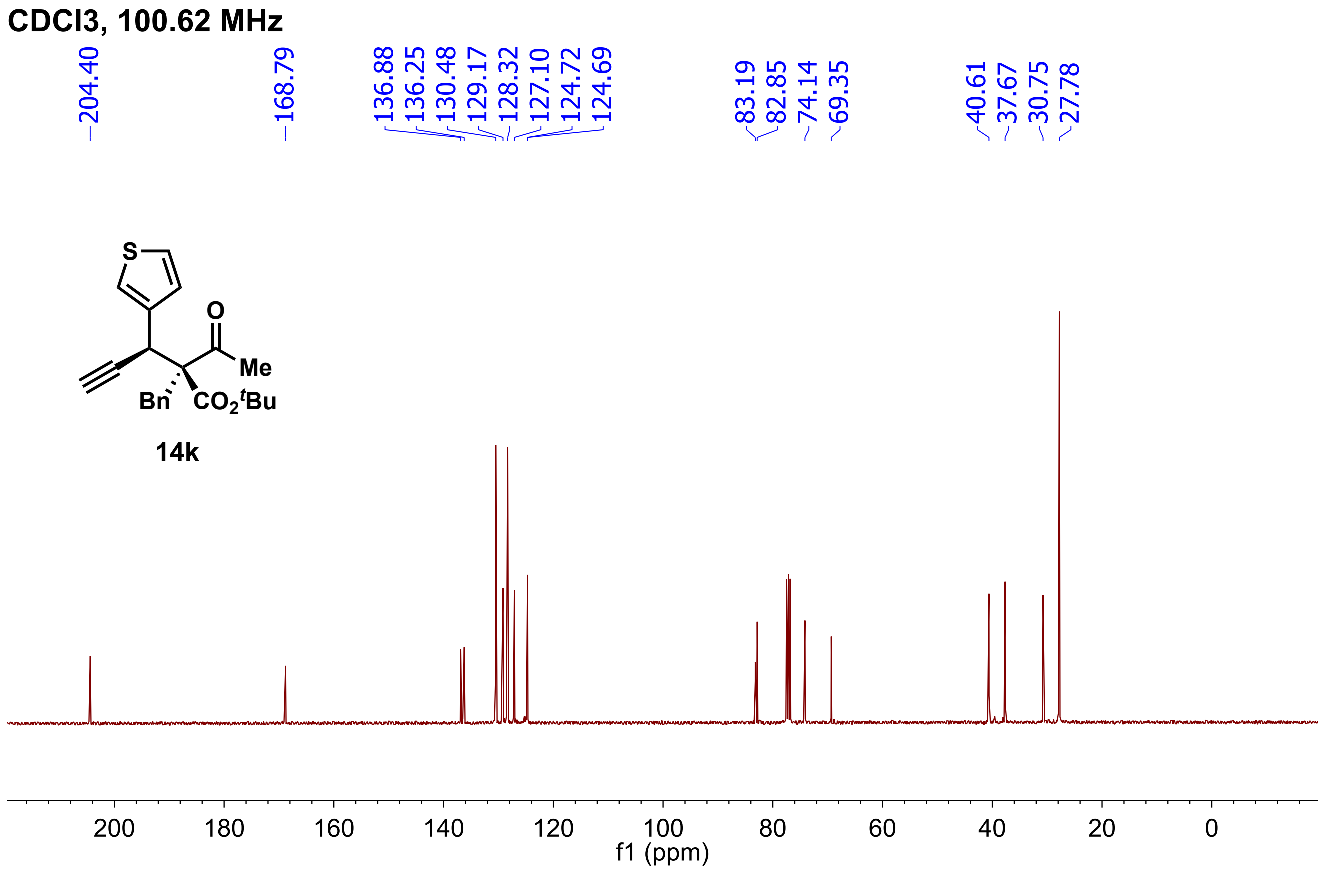

Supplementary Figure 165. ^{13}C NMR (101 MHz, $CDCl_3$) spectra for **14k**

CDCl3, 400.13 MHz

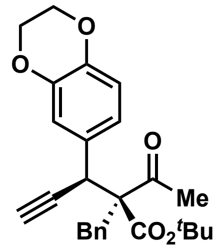

14l

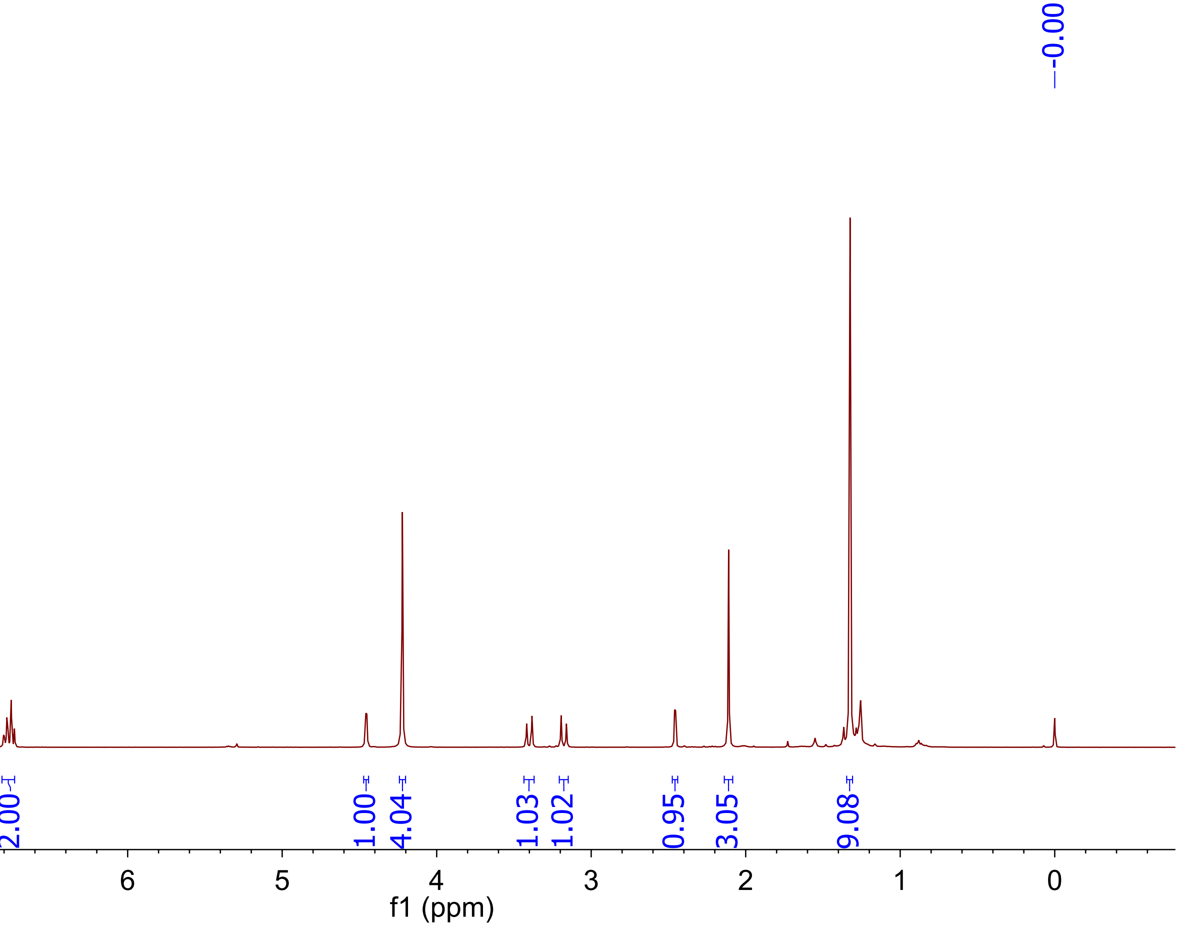

Supplementary Figure 166. ^{1}H NMR (400 MHz, $CDCl_3$) spectra for **14l**

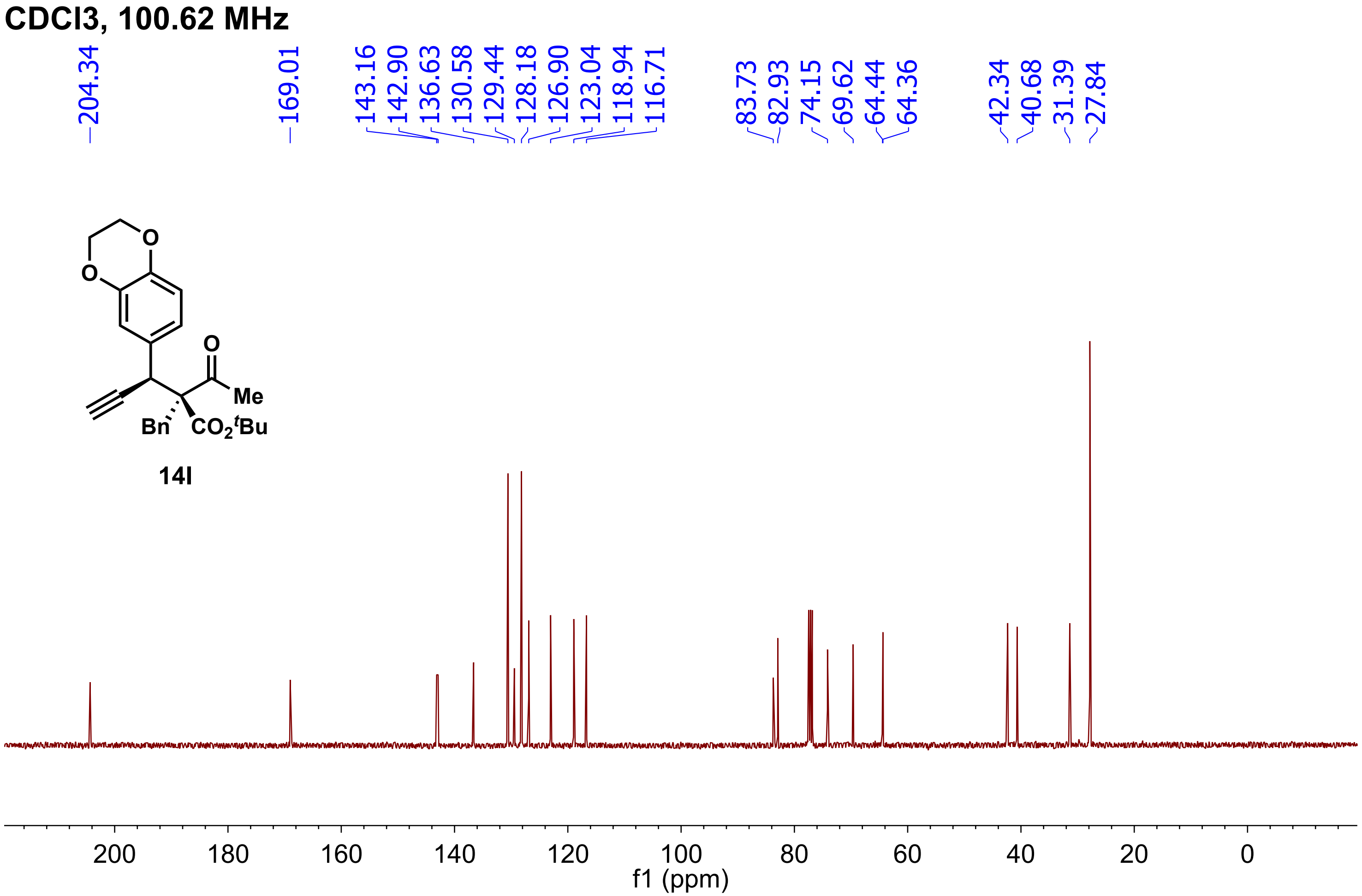

Supplementary Figure 167. ^{13}C NMR (101 MHz, $CDCl_3$) spectra for **14l**

CDCl3, 400.13 MHz

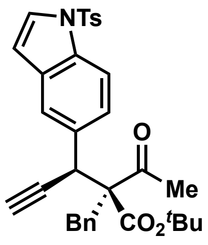

14m

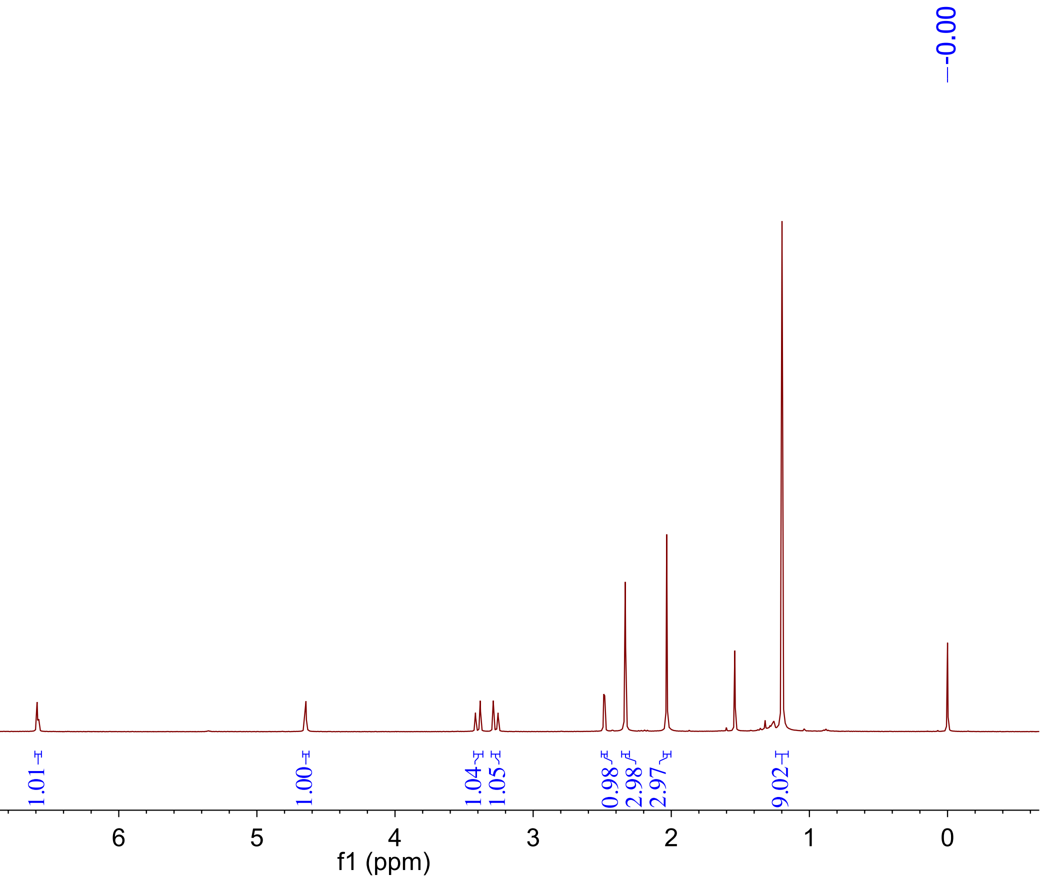

Supplementary Figure 168. ^{1}H NMR (400 MHz, $CDCl_3$) spectra for **14m**

CDCl3, 100.62 MHz

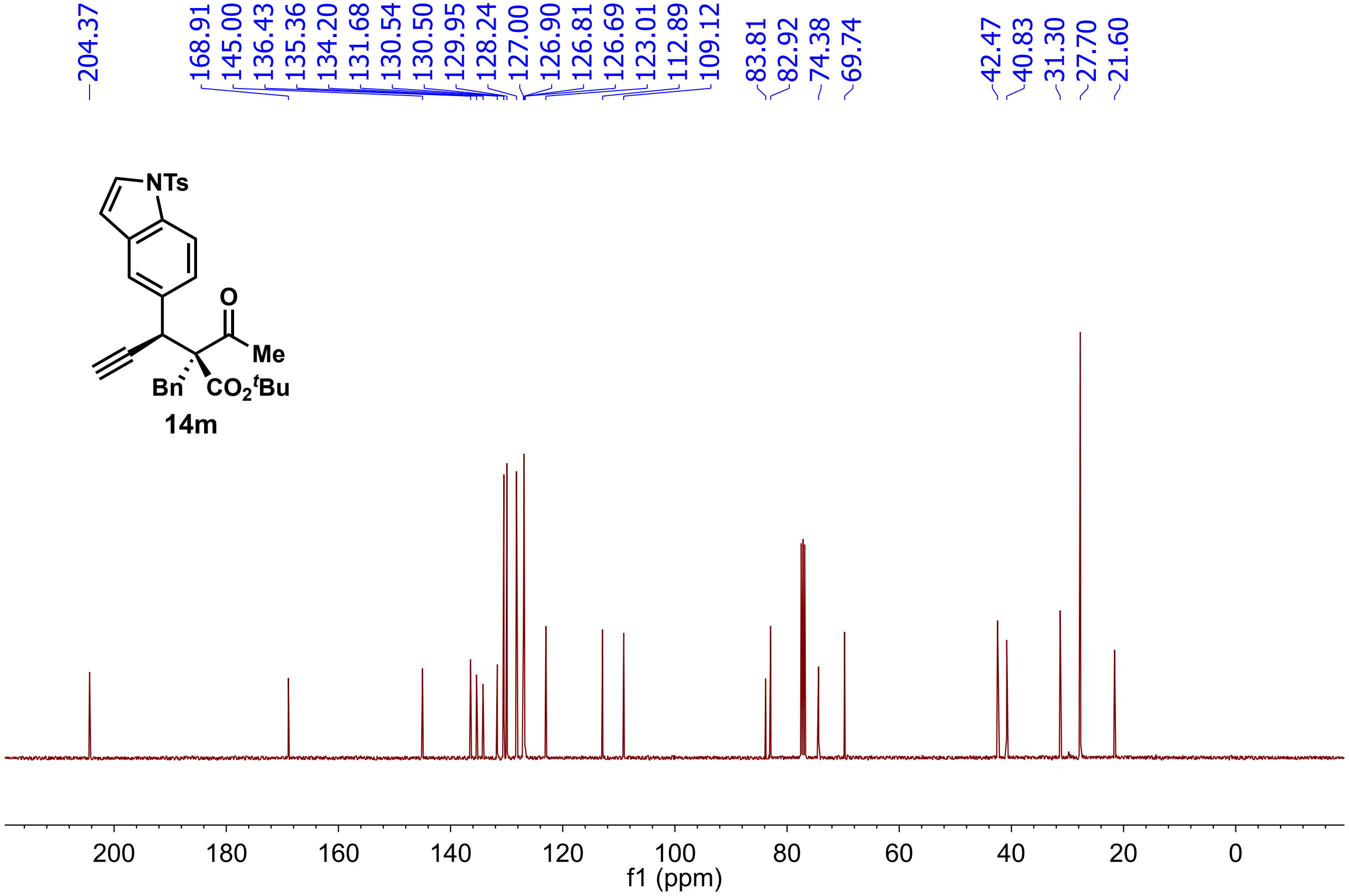

Supplementary Figure 169. ^{13}C NMR (101 MHz, $CDCl_3$) spectra for **14m**

CDCl3, 400.13 MHz

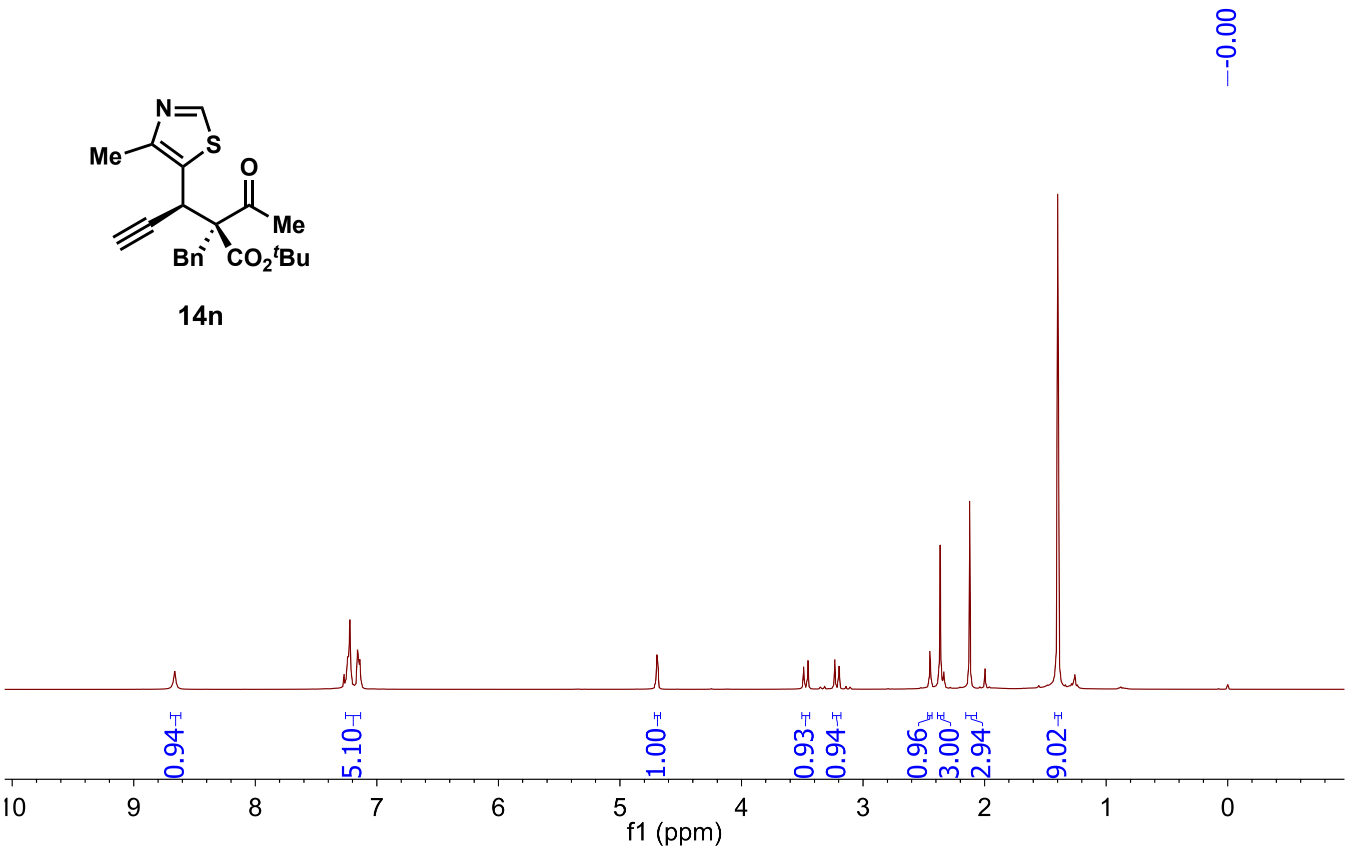

Supplementary Figure 170. ^{1}H NMR (400 MHz, $CDCl_3$) spectra for **14n**

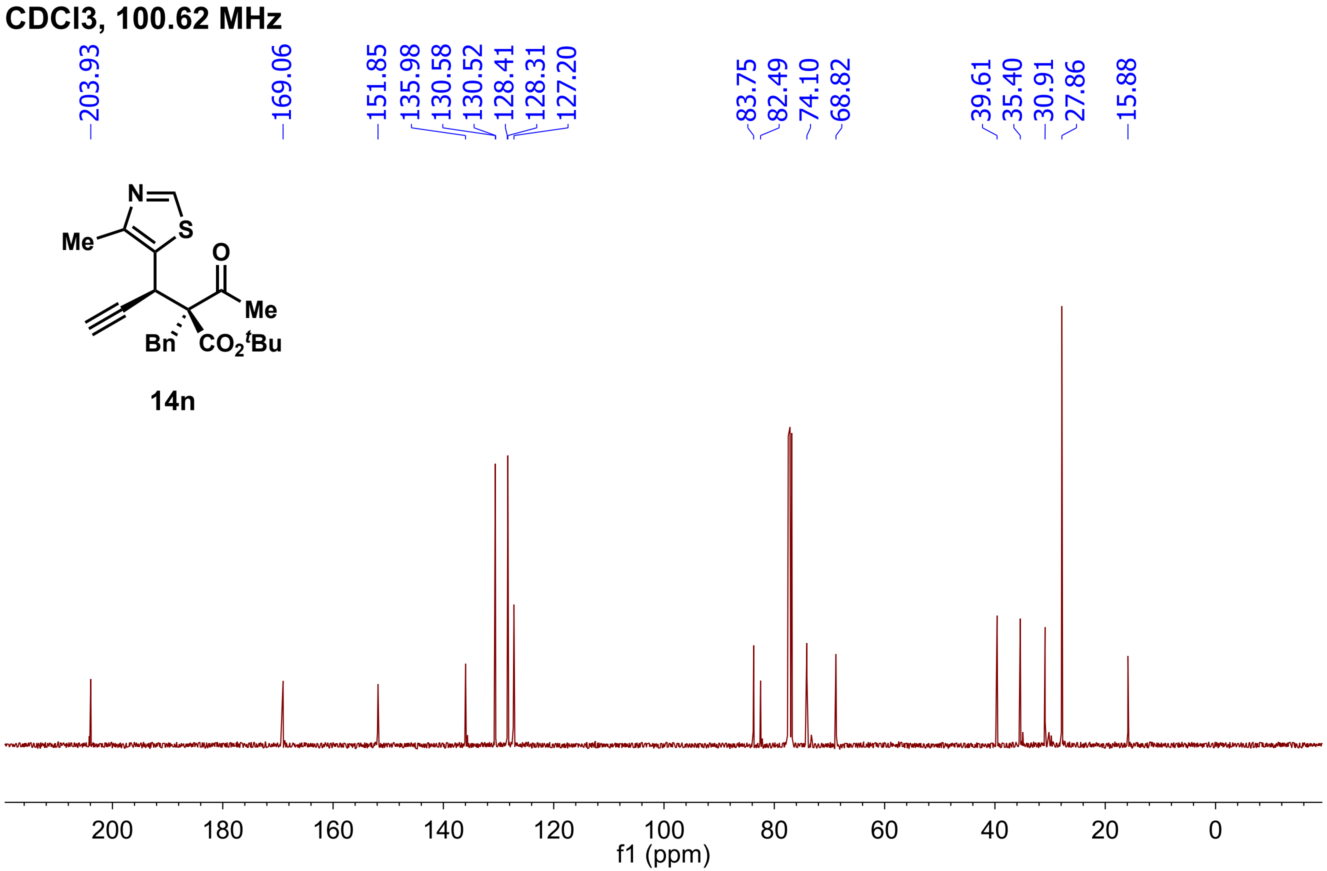

Supplementary Figure 171. ^{13}C NMR (101 MHz, $CDCl_3$) spectra for **14n**

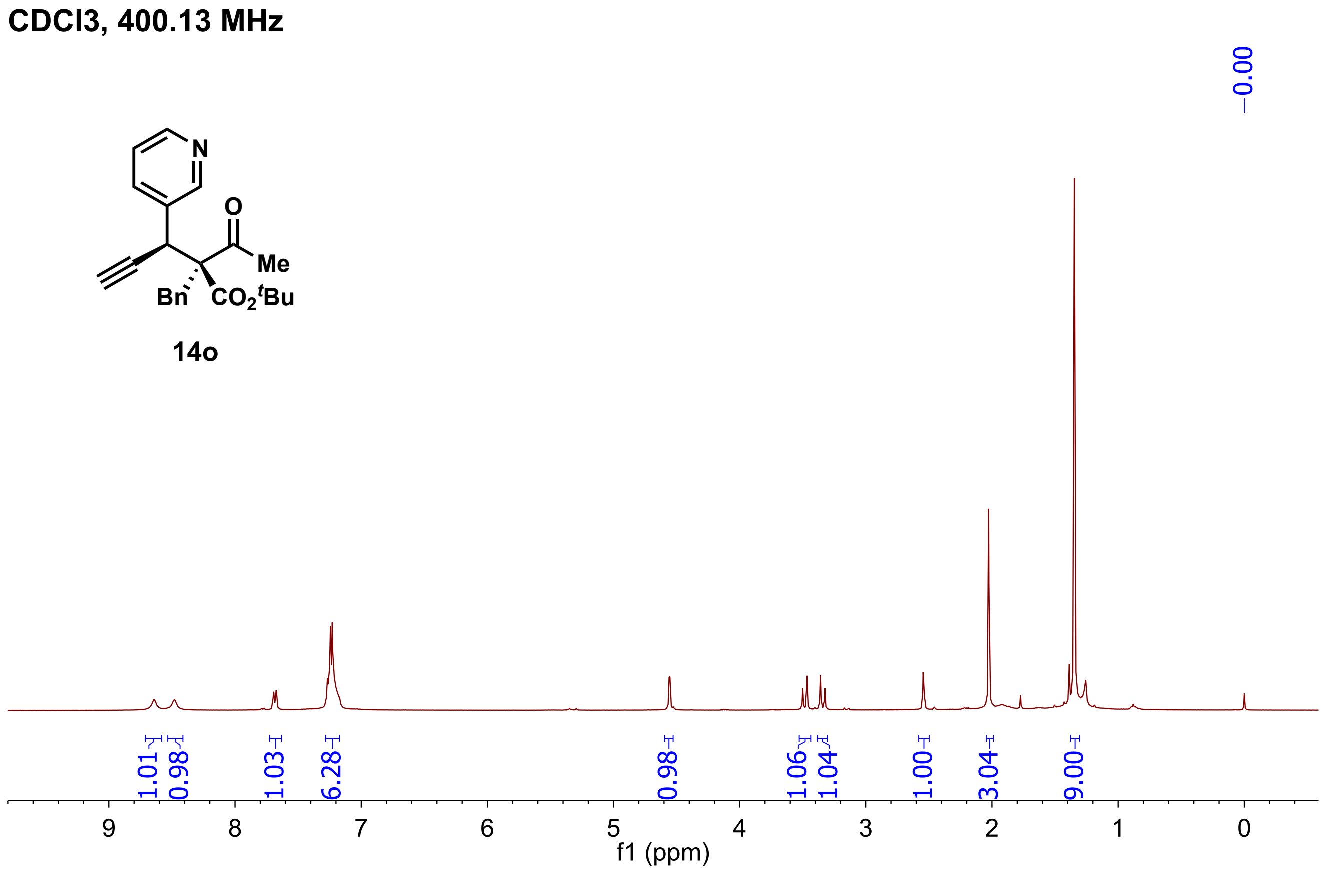

Supplementary Figure 172. ^{1}H NMR (400 MHz, $CDCl_3$) spectra for **14o**

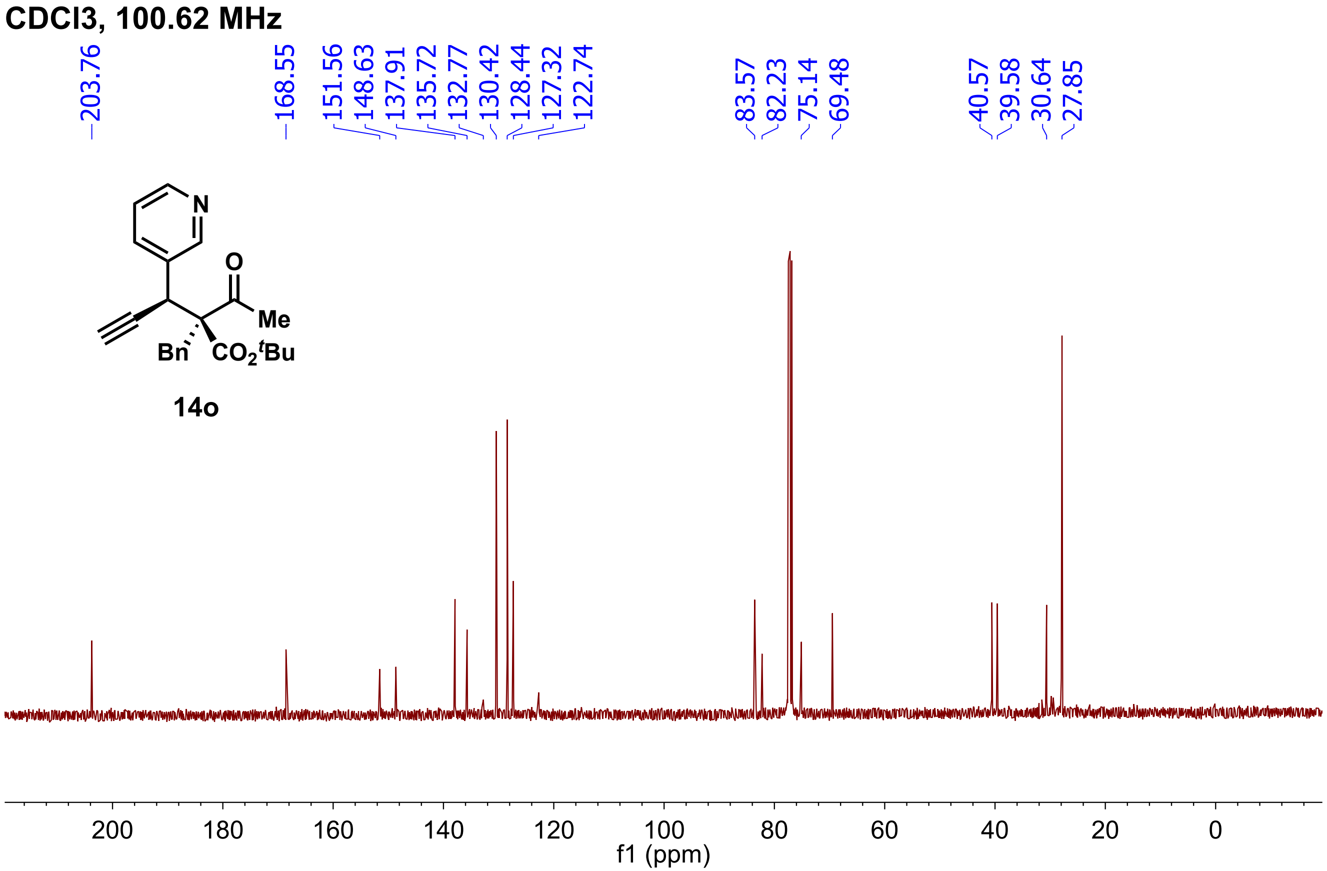

Supplementary Figure 173. ^{13}C NMR (101 MHz, $CDCl_3$) spectra for **14o**

CDCl3, 400.13 MHz

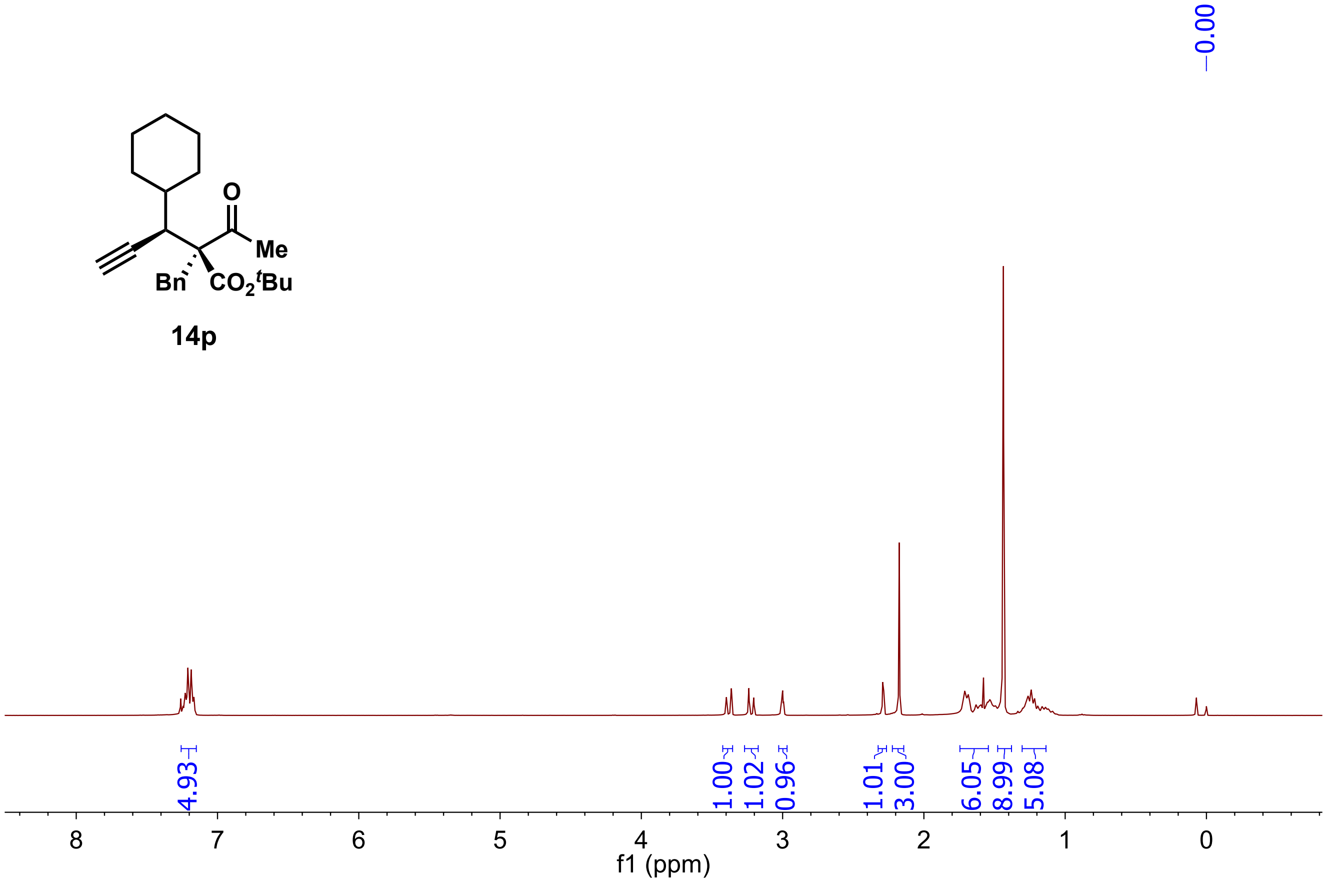

Supplementary Figure 174. ^{1}H NMR (400 MHz, $CDCl_3$) spectra for **14p**

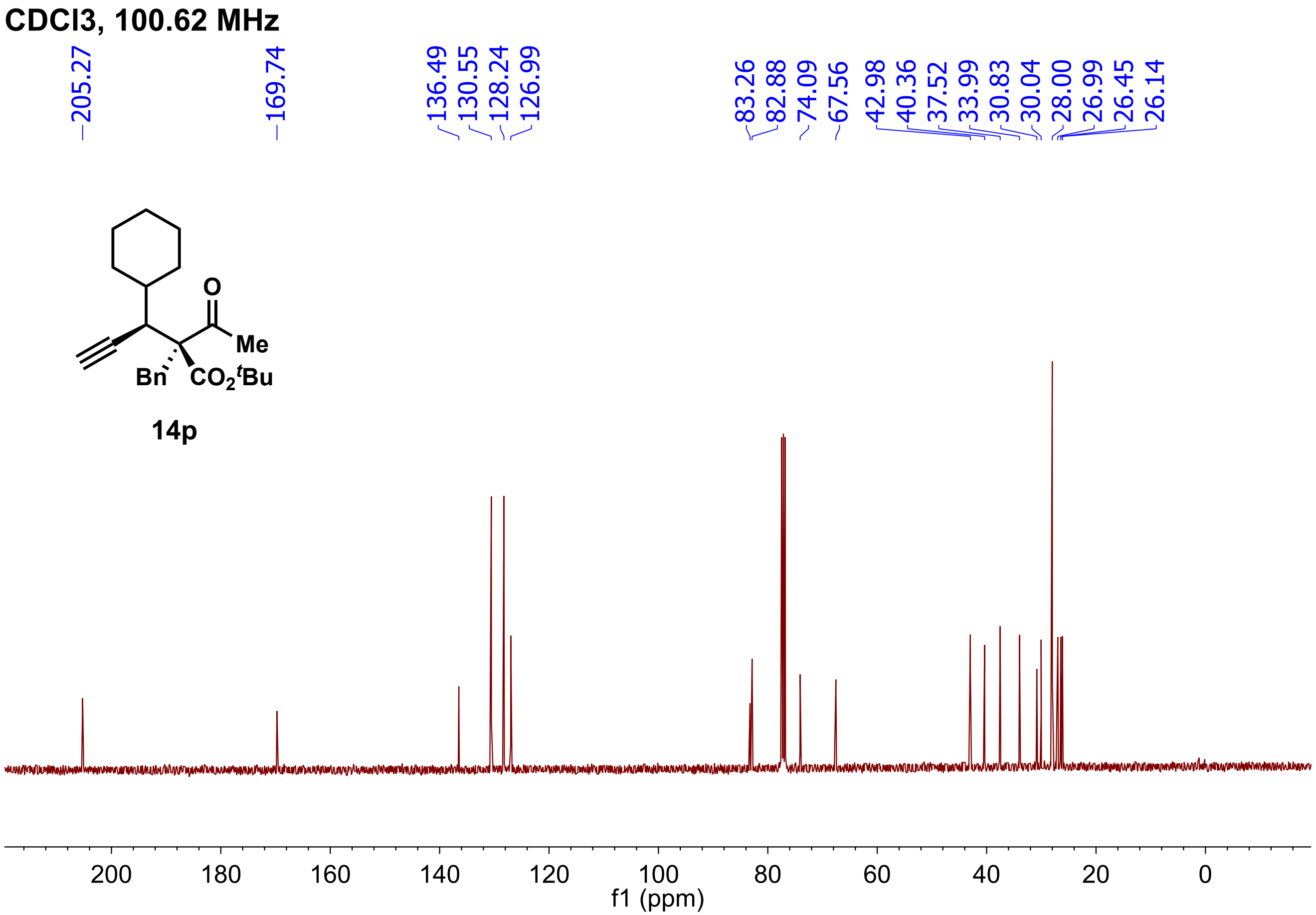

Supplementary Figure 175. ^{13}C NMR (101 MHz, $CDCl_3$) spectra for **14p**

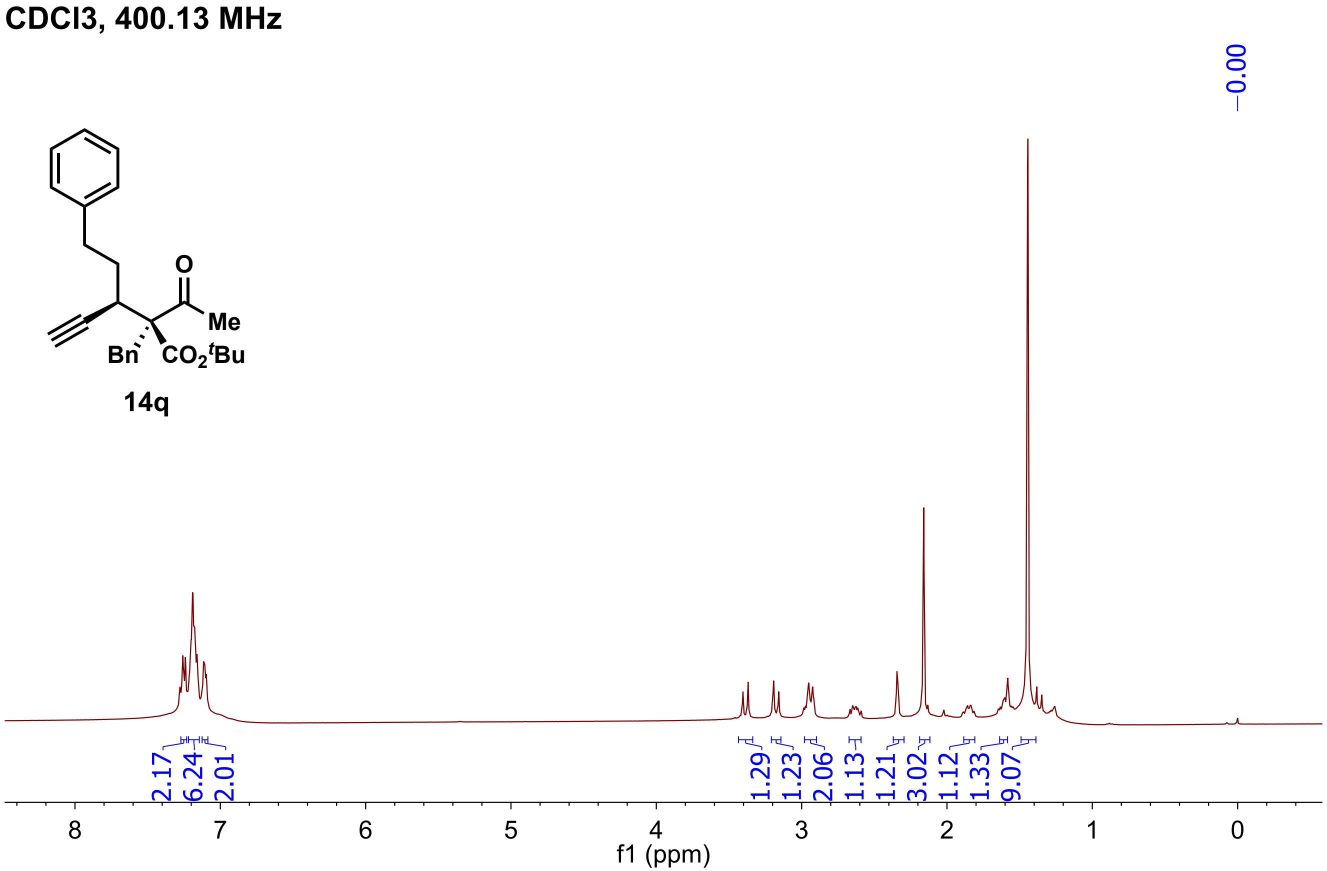

Supplementary Figure 176. ^{1}H NMR (400 MHz, $CDCl_3$) spectra for **14q**

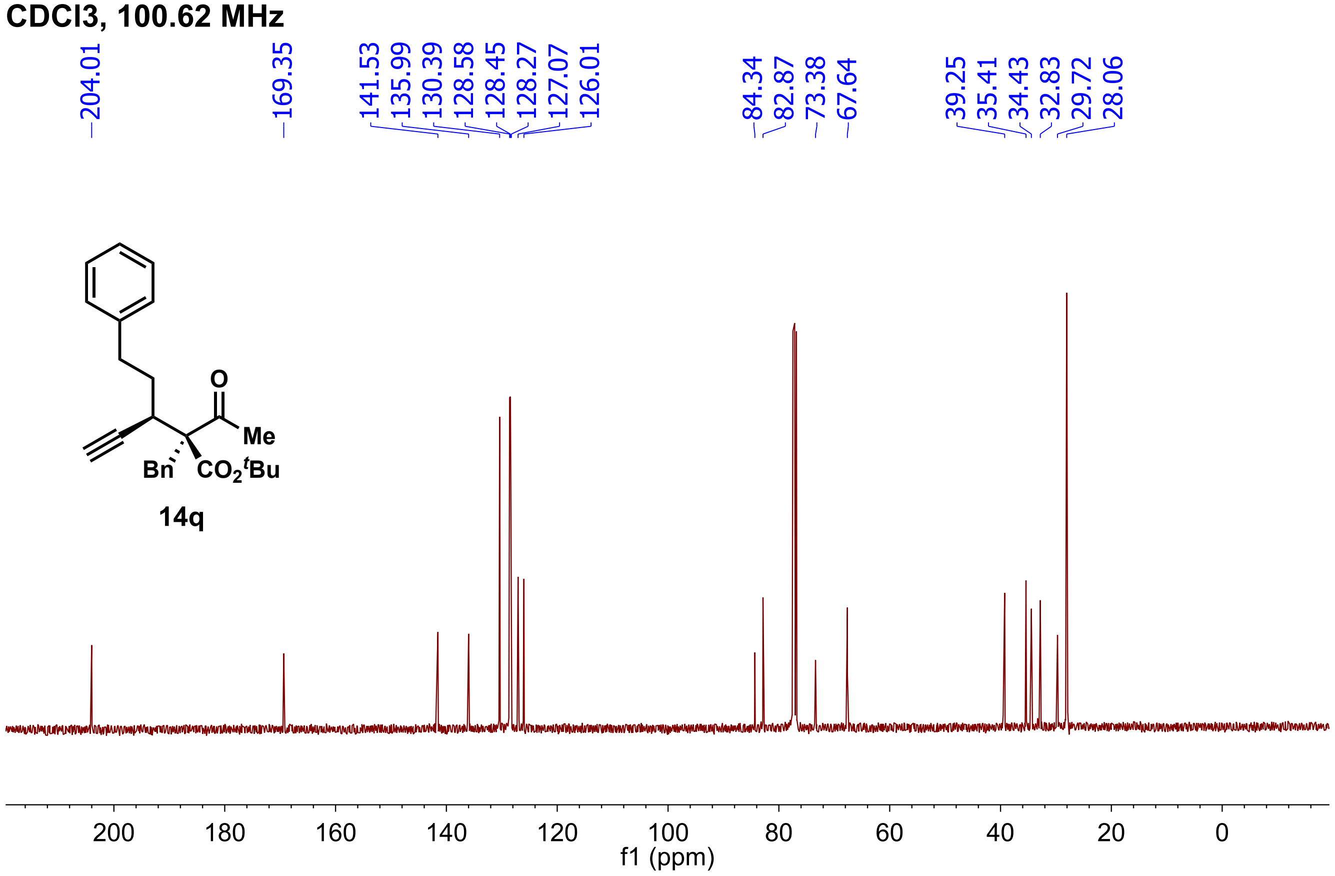

Supplementary Figure 177. ^{13}C NMR (101 MHz, $CDCl_3$) spectra for **14q**

CDCl3, 400.13 MHz

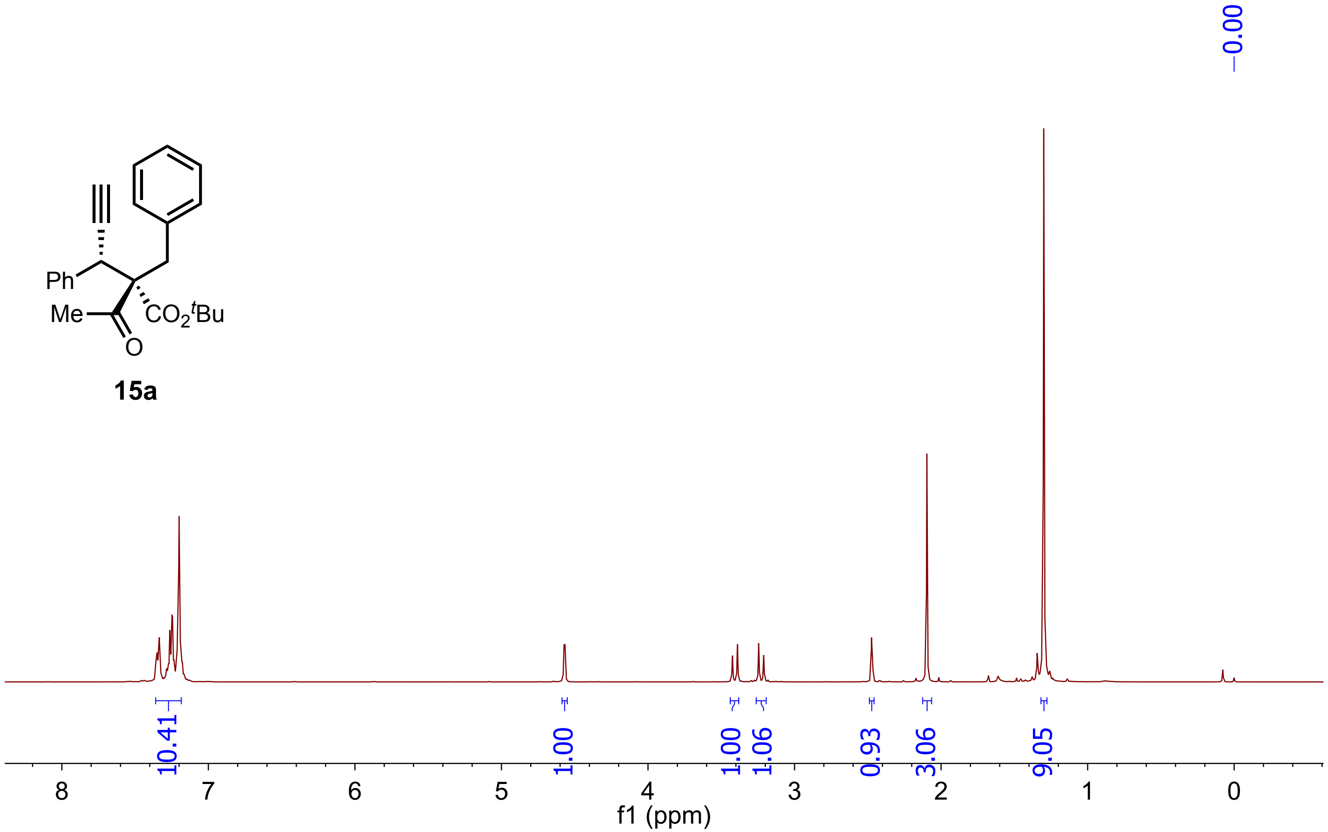

Supplementary Figure 178. ^{1}H NMR (400 MHz, $CDCl_3$) spectra for **15a**

CDCl3, 100.62 MHz

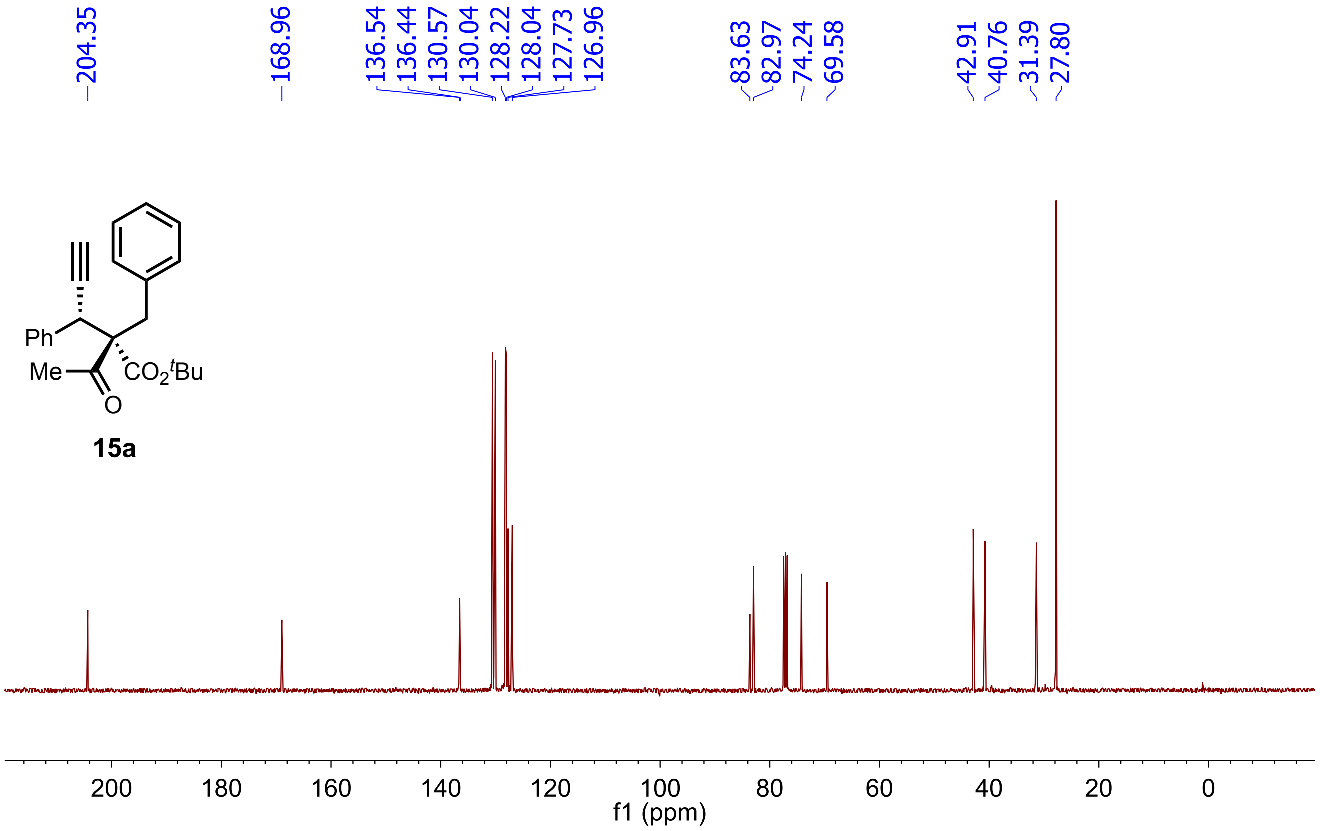

Supplementary Figure 179. ^{13}C NMR (101 MHz, $CDCl_3$) spectra for **15a**

CDCl3, 400.13 MHz

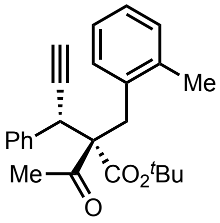

15b

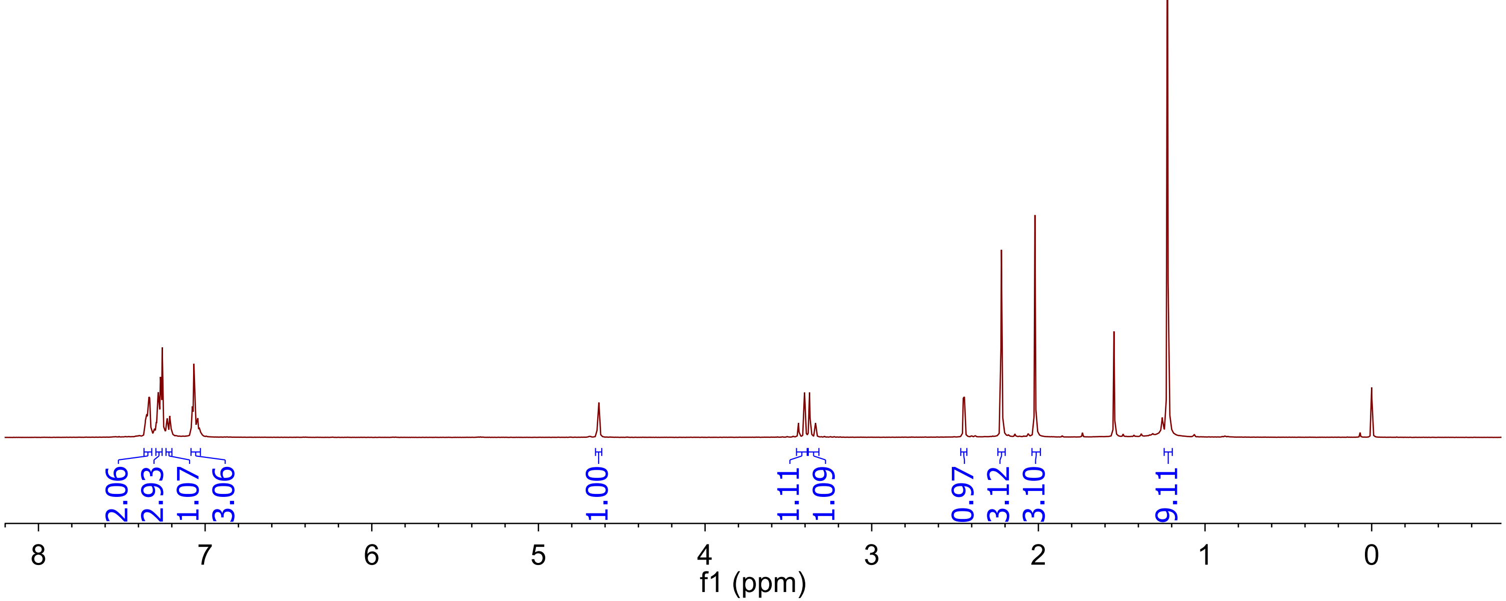

Supplementary Figure 180. ^{1}H NMR (400 MHz, $CDCl_3$) spectra for **15b**

CDCl3, 100.62 MHz

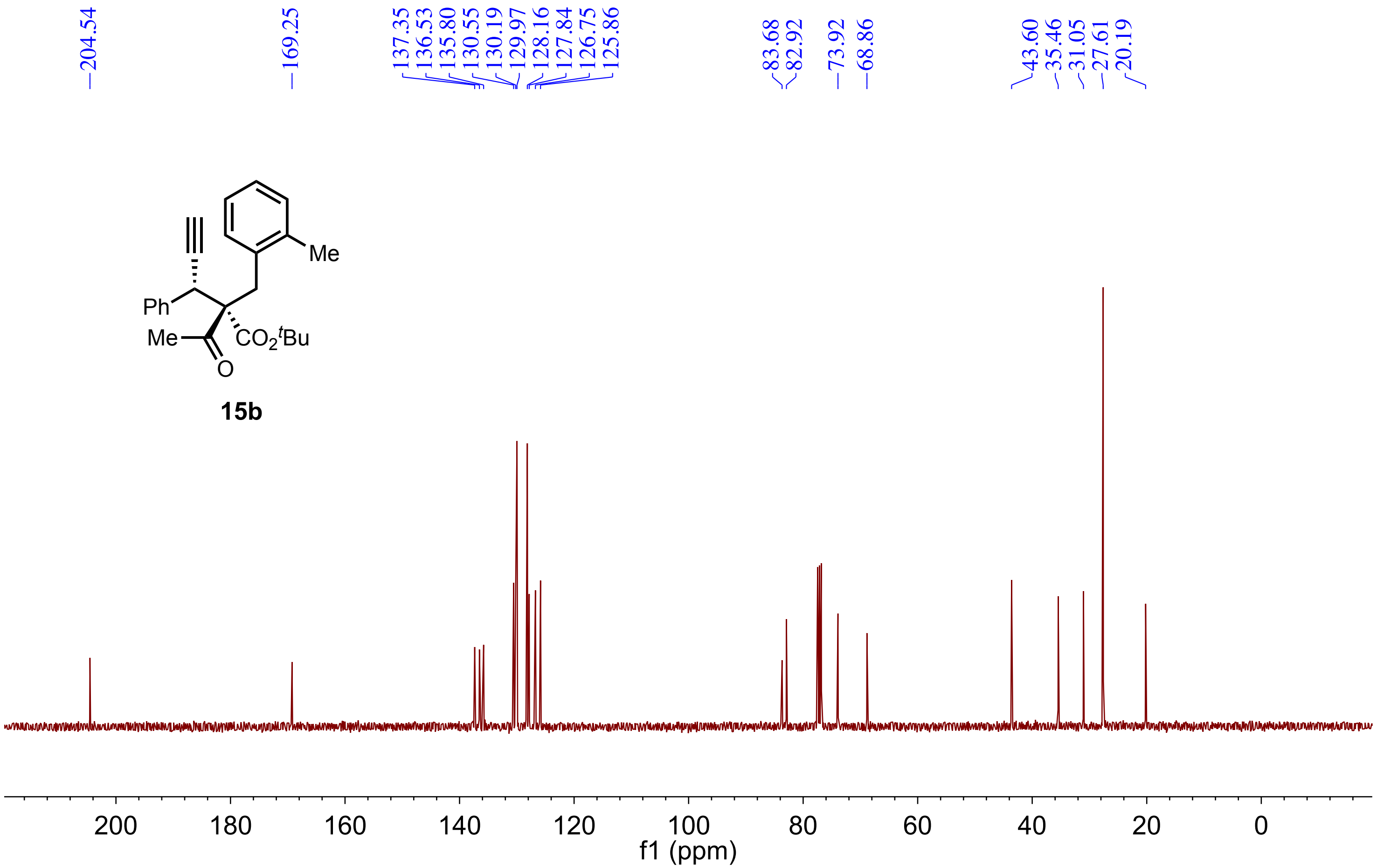

Supplementary Figure 181. ^{13}C NMR (101 MHz, $CDCl_3$) spectra for **15b**

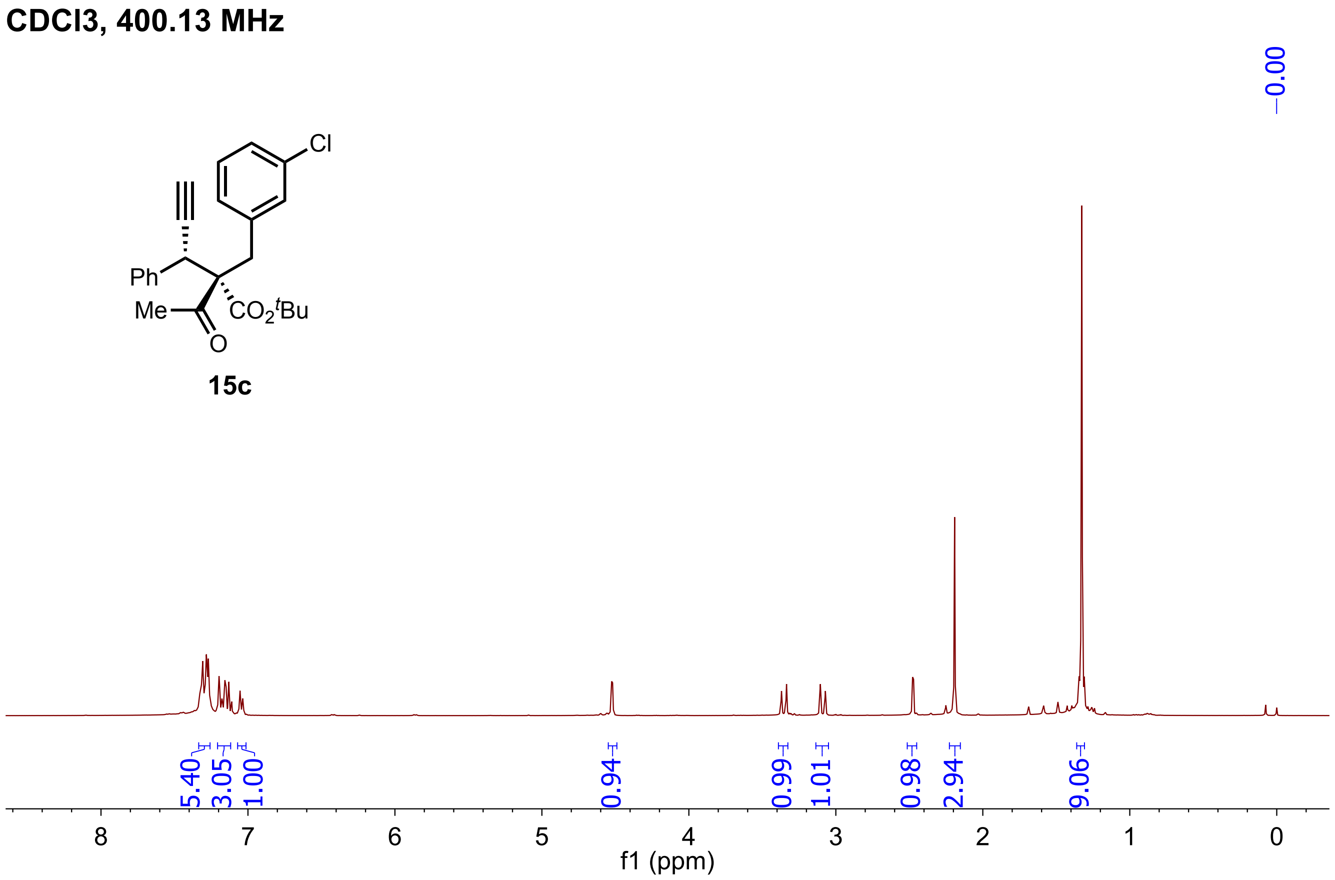

Supplementary Figure 182. ^{1}H NMR (400 MHz, $CDCl_3$) spectra for **15c**

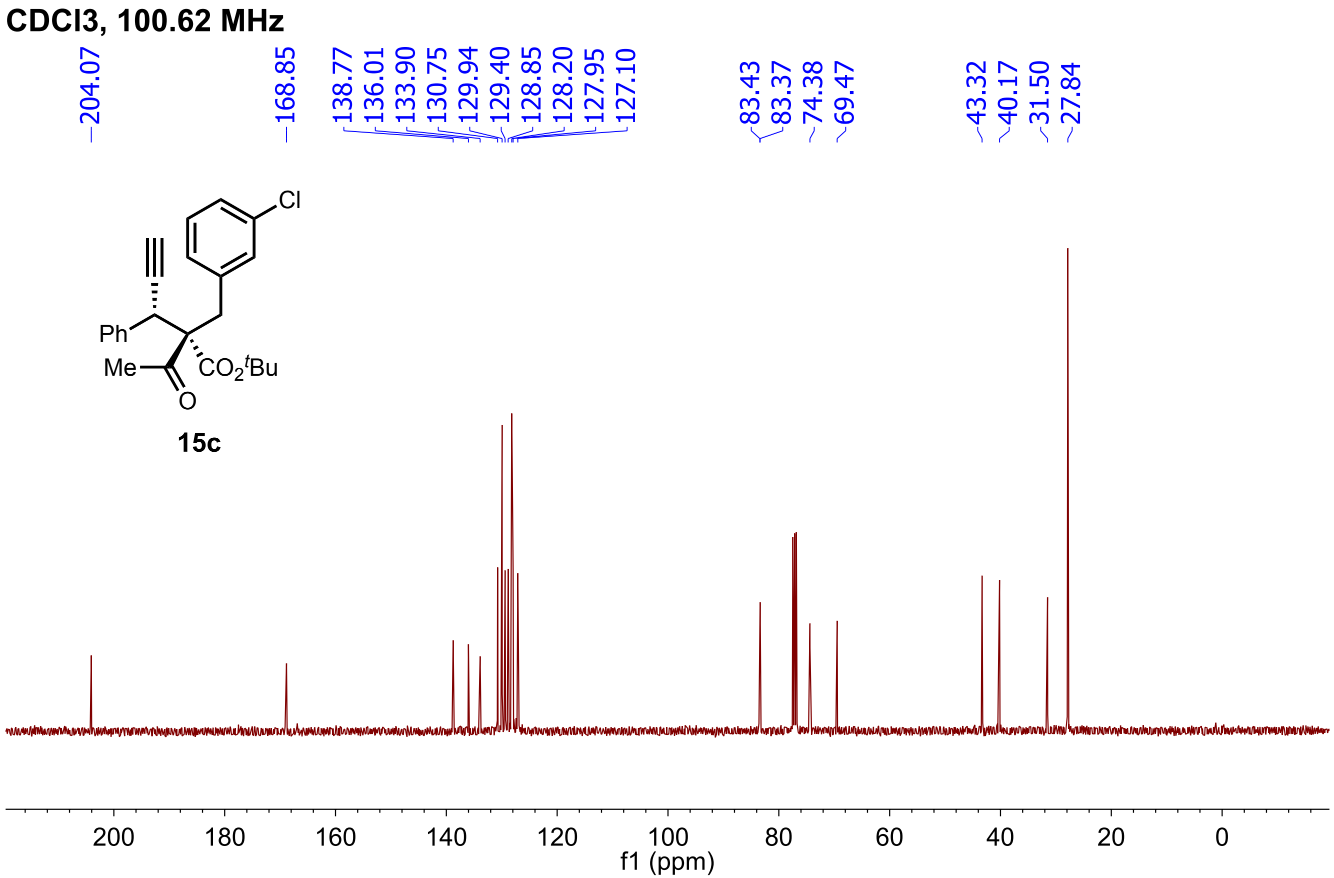

Supplementary Figure 183. ^{13}C NMR (101 MHz, $CDCl_3$) spectra for **15c**

CDCl3, 400.13 MHz

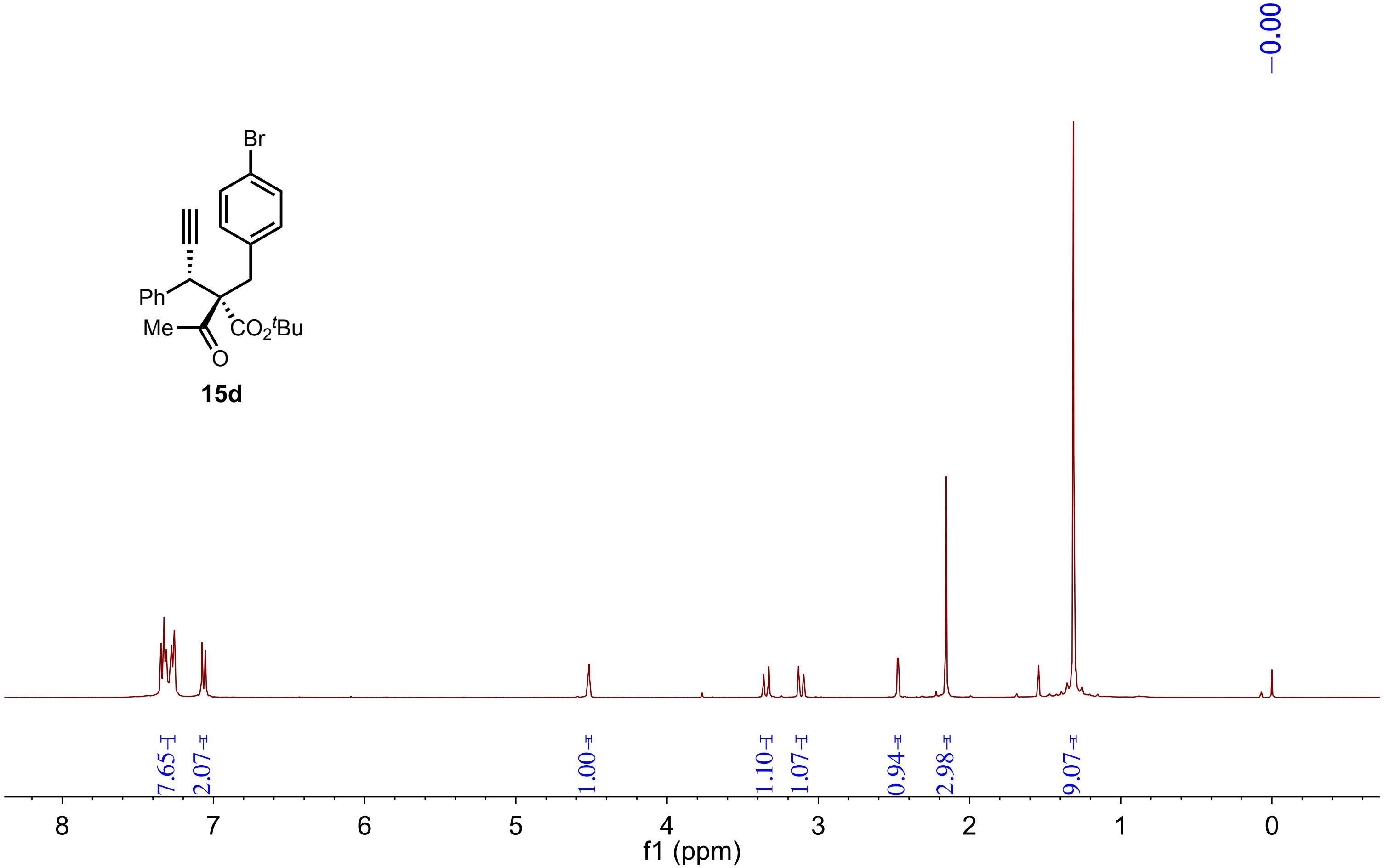

Supplementary Figure 184. ^{1}H NMR (400 MHz, $CDCl_3$) spectra for **15d**

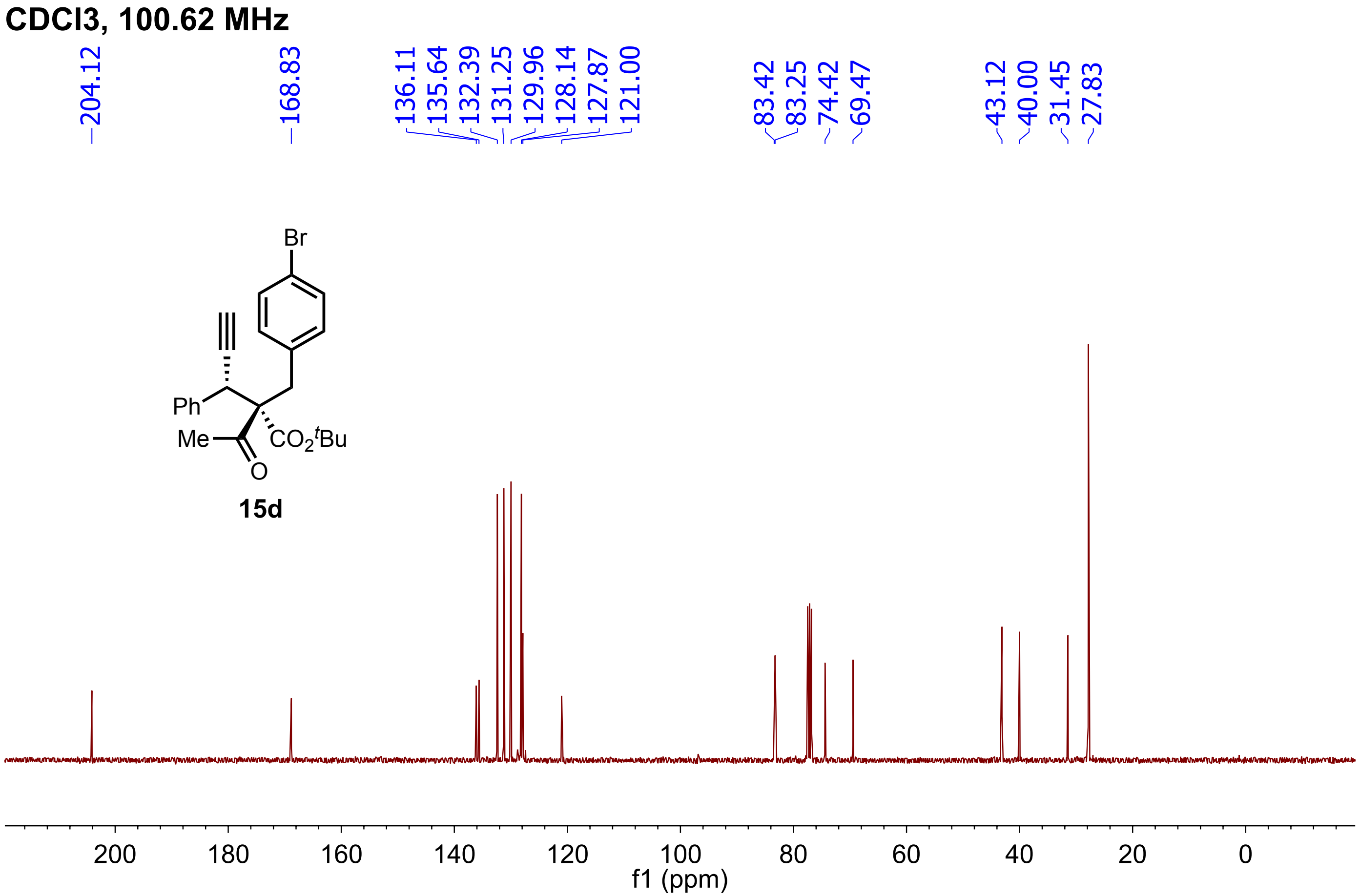

Supplementary Figure 185. ^{13}C NMR (101 MHz, $CDCl_3$) spectra for **15d**

CDCl3, 400.13 MHz

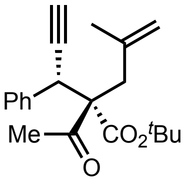

15e

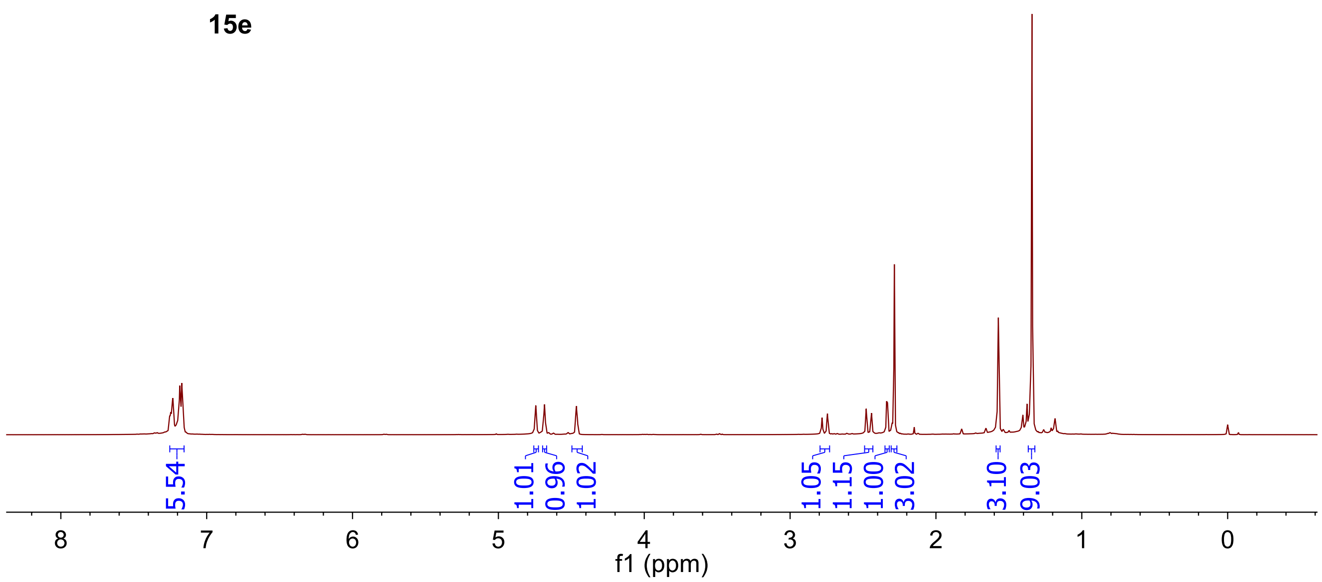

Supplementary Figure 186. ^{1}H NMR (400 MHz, $CDCl_3$) spectra for **15e**

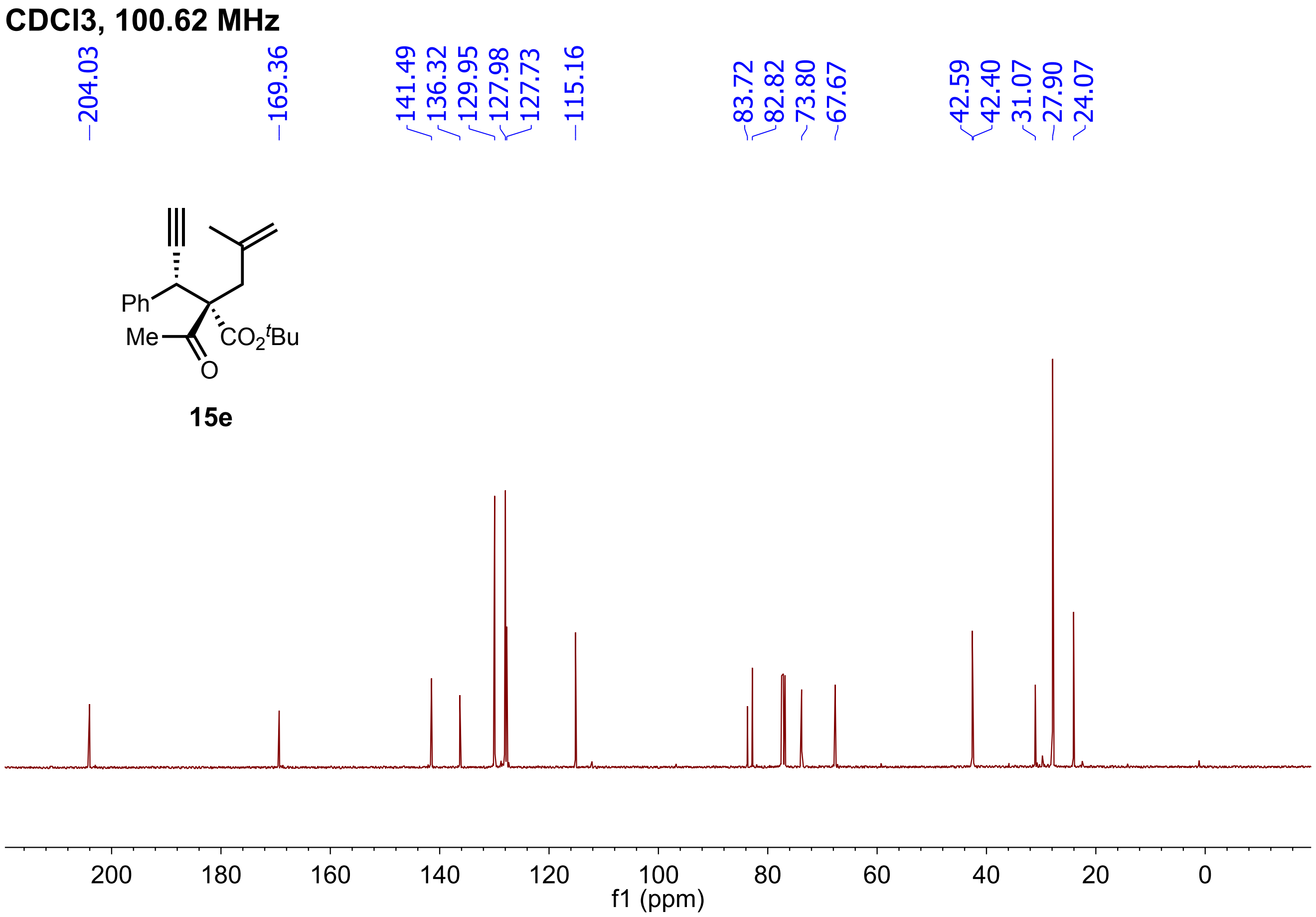

Supplementary Figure 187. ^{13}C NMR (101 MHz, $CDCl_3$) spectra for **15e**

CDCl3, 400.13 MHz

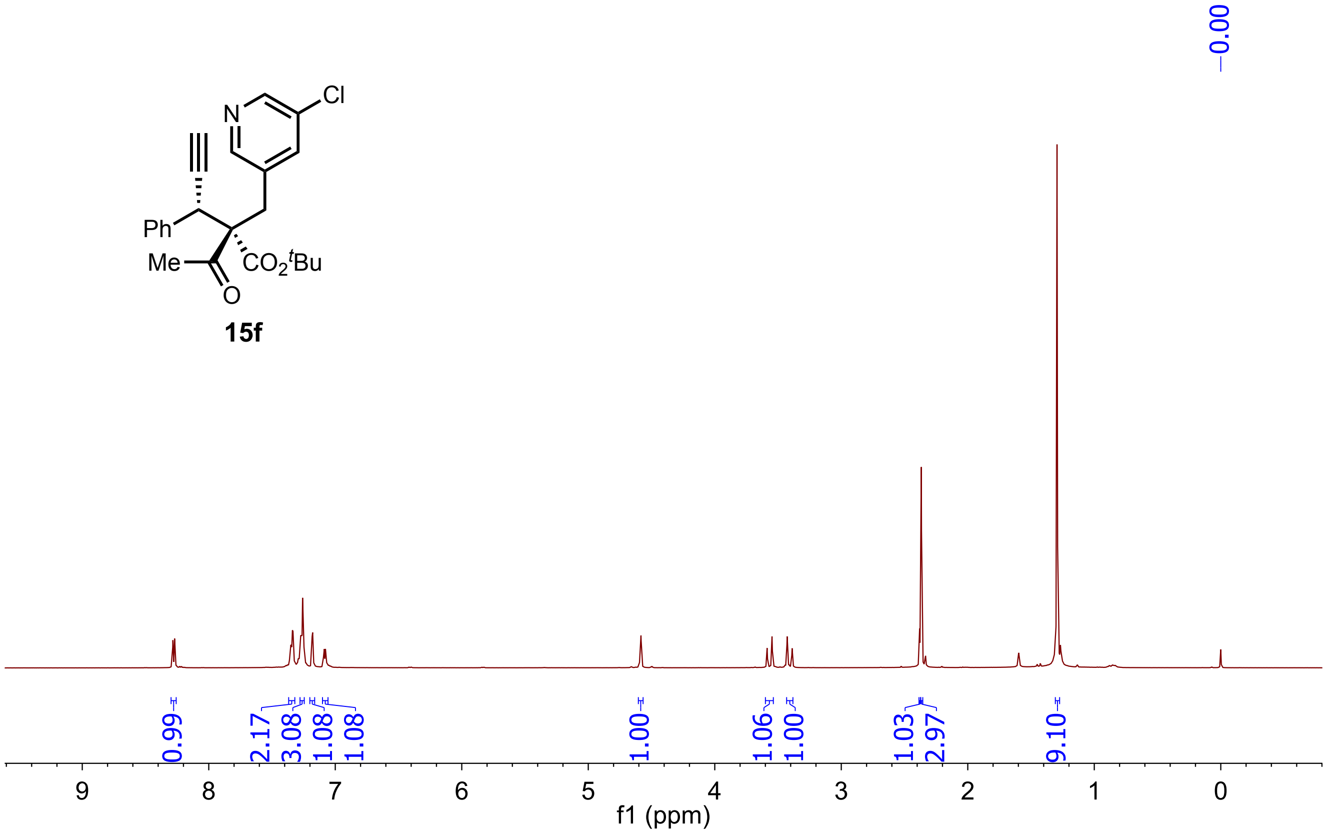

Supplementary Figure 188. ^{1}H NMR (400 MHz, $CDCl_3$) spectra for **15f**

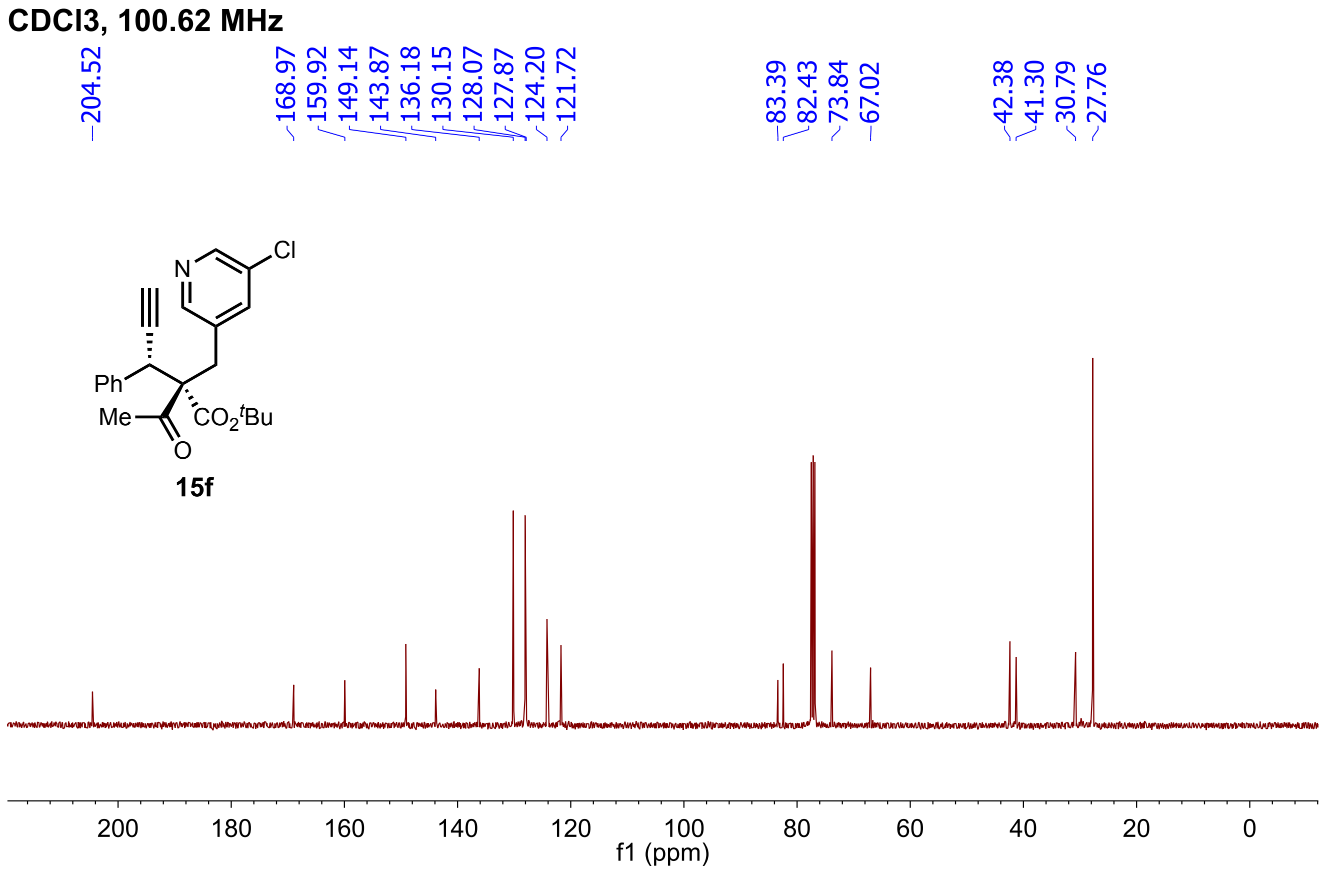

Supplementary Figure 189. ^{13}C NMR (101 MHz, $CDCl_3$) spectra for **15f**

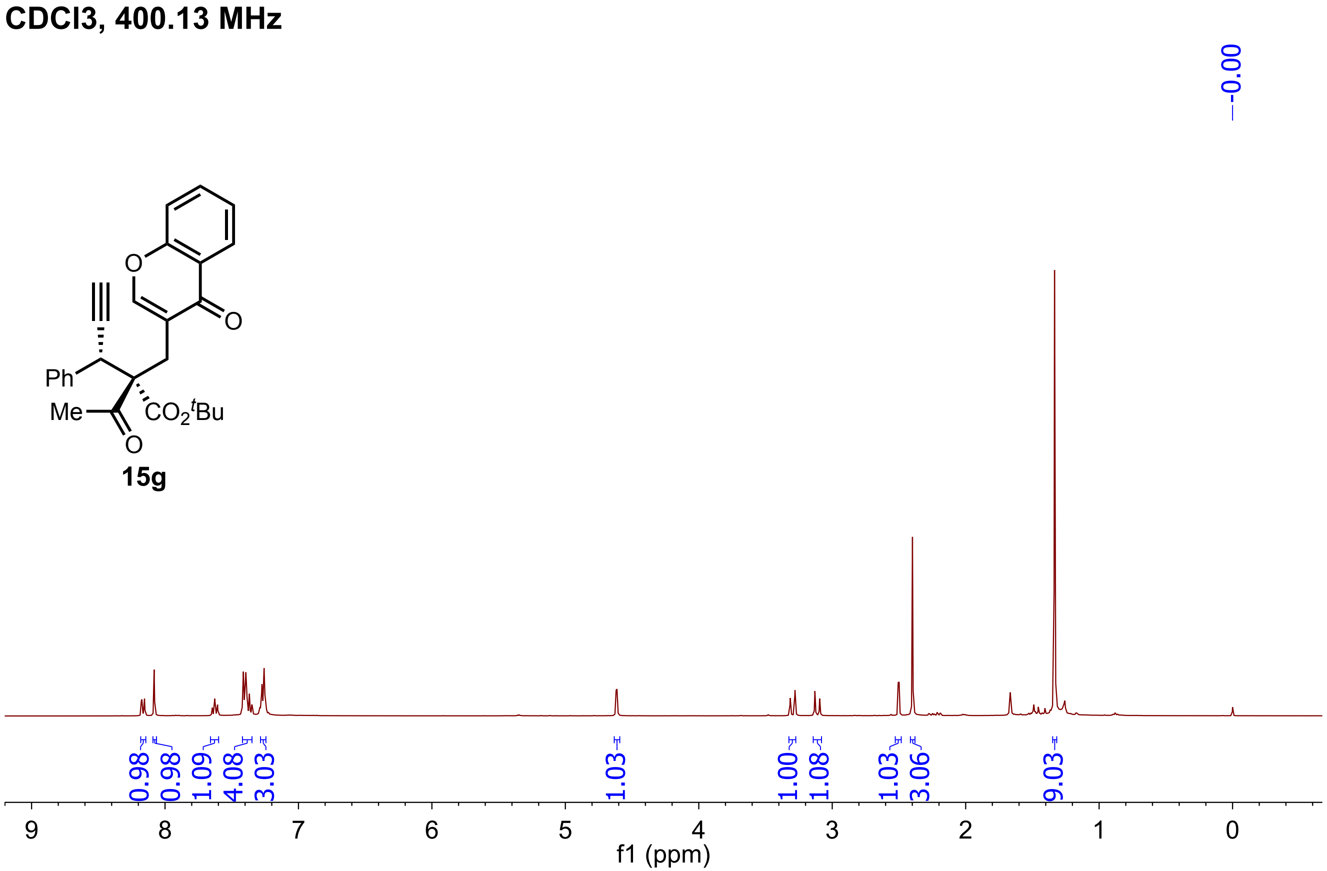

Supplementary Figure 190. ^{1}H NMR (400 MHz, $CDCl_3$) spectra for **15g**

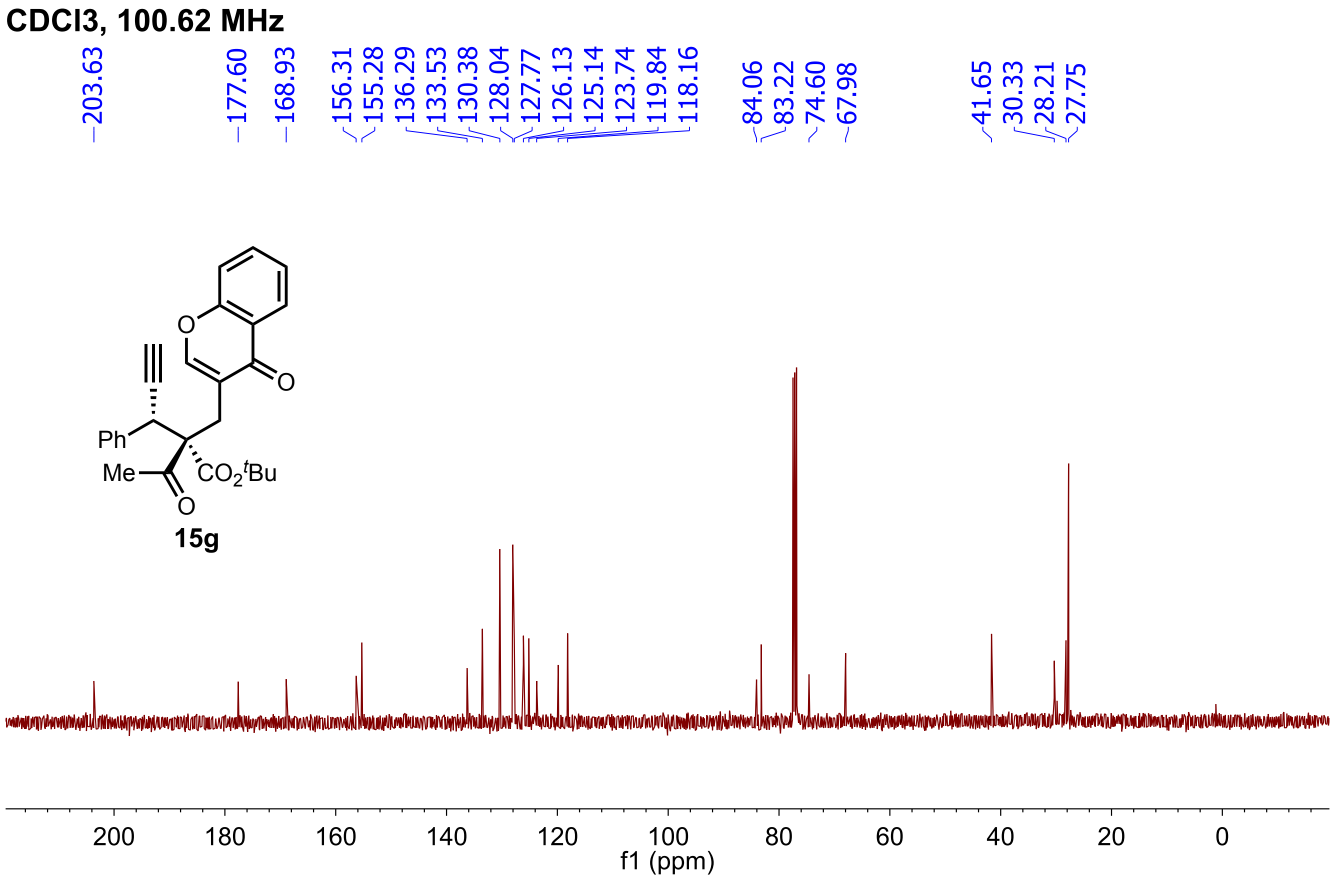

Supplementary Figure 191. ^{13}C NMR (101 MHz, $CDCl_3$) spectra for **15g**

CDCl3, 400.13 MHz

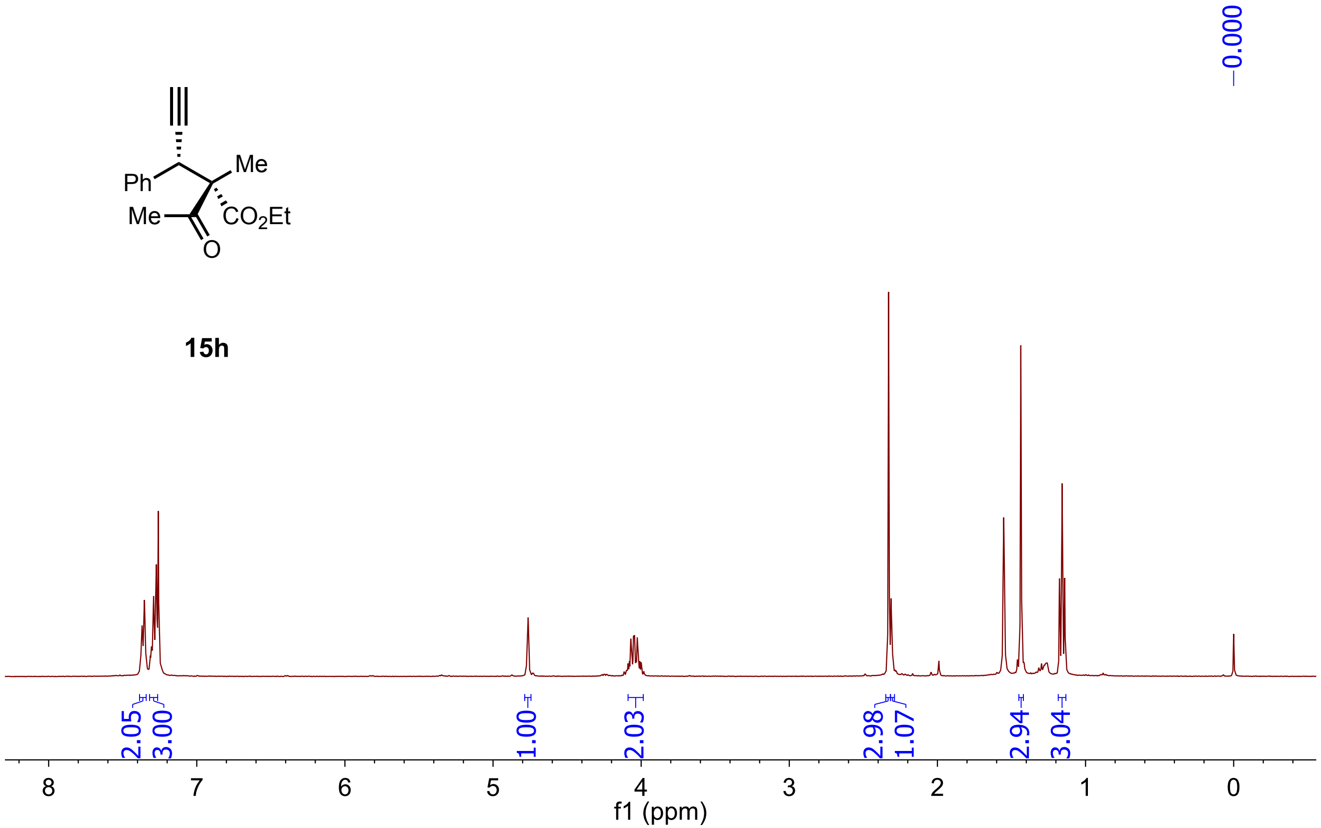

Supplementary Figure 192. ^{1}H NMR (400 MHz, $CDCl_3$) spectra for **15h**

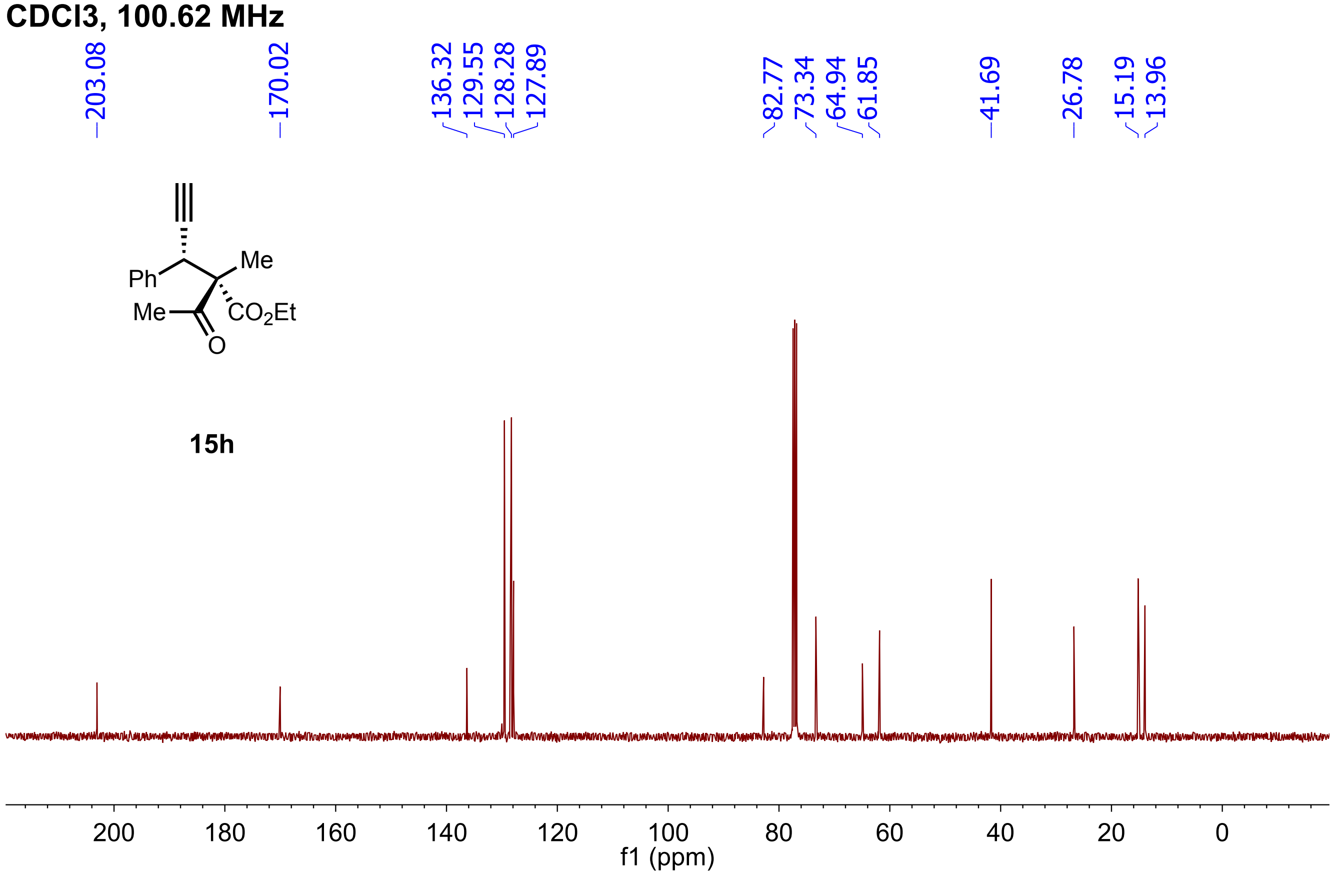

Supplementary Figure 193. ^{13}C NMR (101 MHz, $CDCl_3$) spectra for **15h**

CDCl3, 400.13 MHz

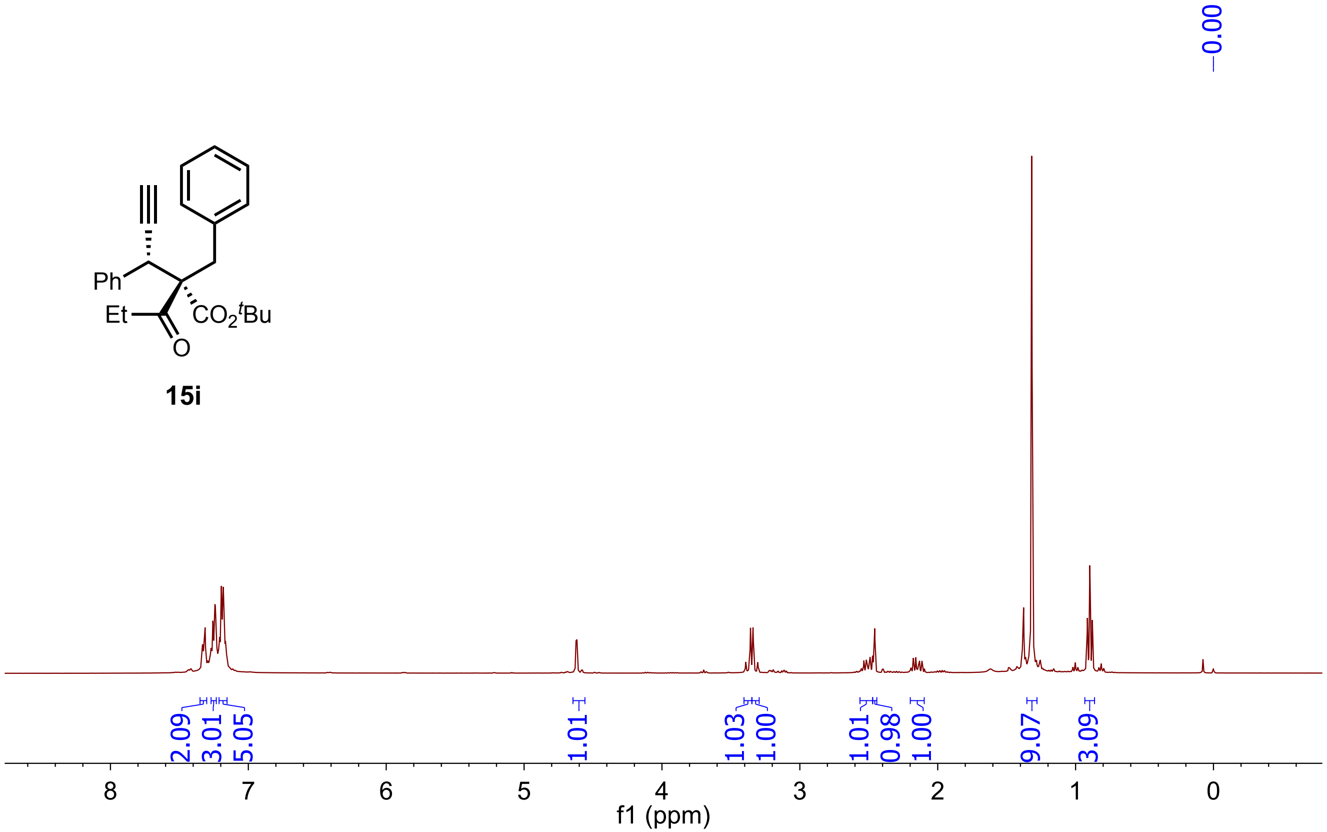

Supplementary Figure 194. ^{1}H NMR (400 MHz, $CDCl_3$) spectra for **15i**

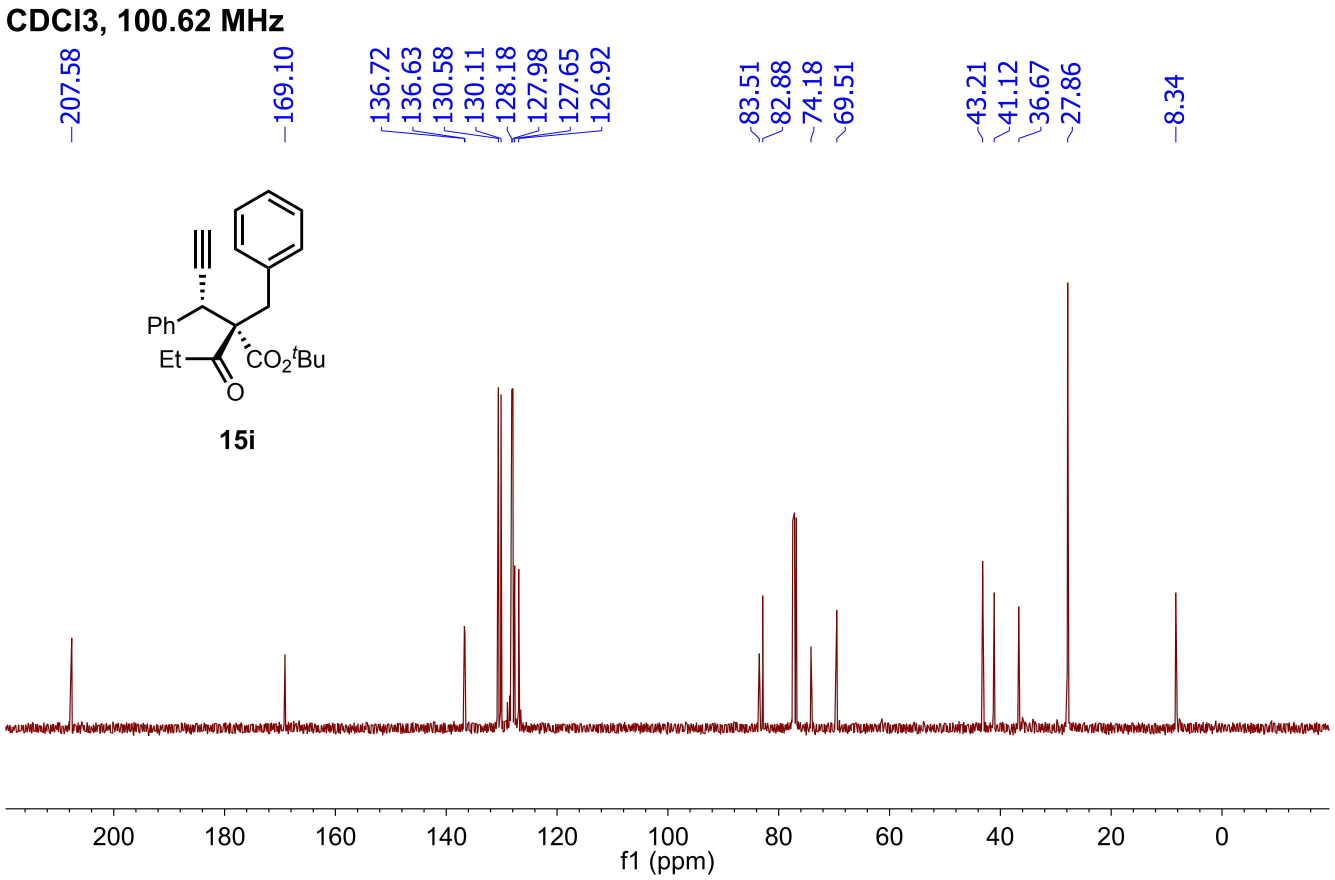

Supplementary Figure 195. ^{13}C NMR (101 MHz, $CDCl_3$) spectra for **15i**

CDCl3, 400.13 MHz

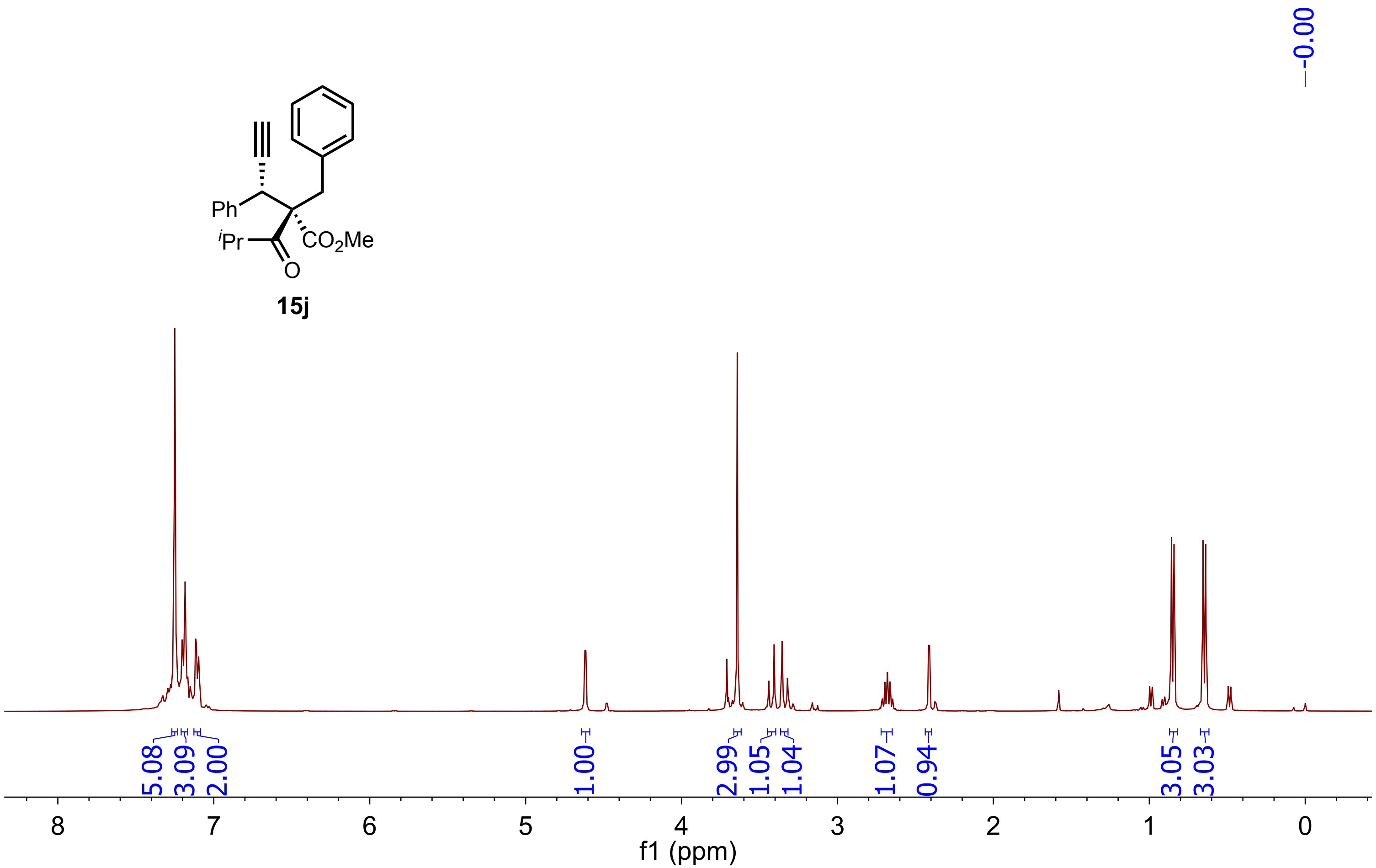

Supplementary Figure 196. ^{1}H NMR (400 MHz, $CDCl_3$) spectra for **15j**

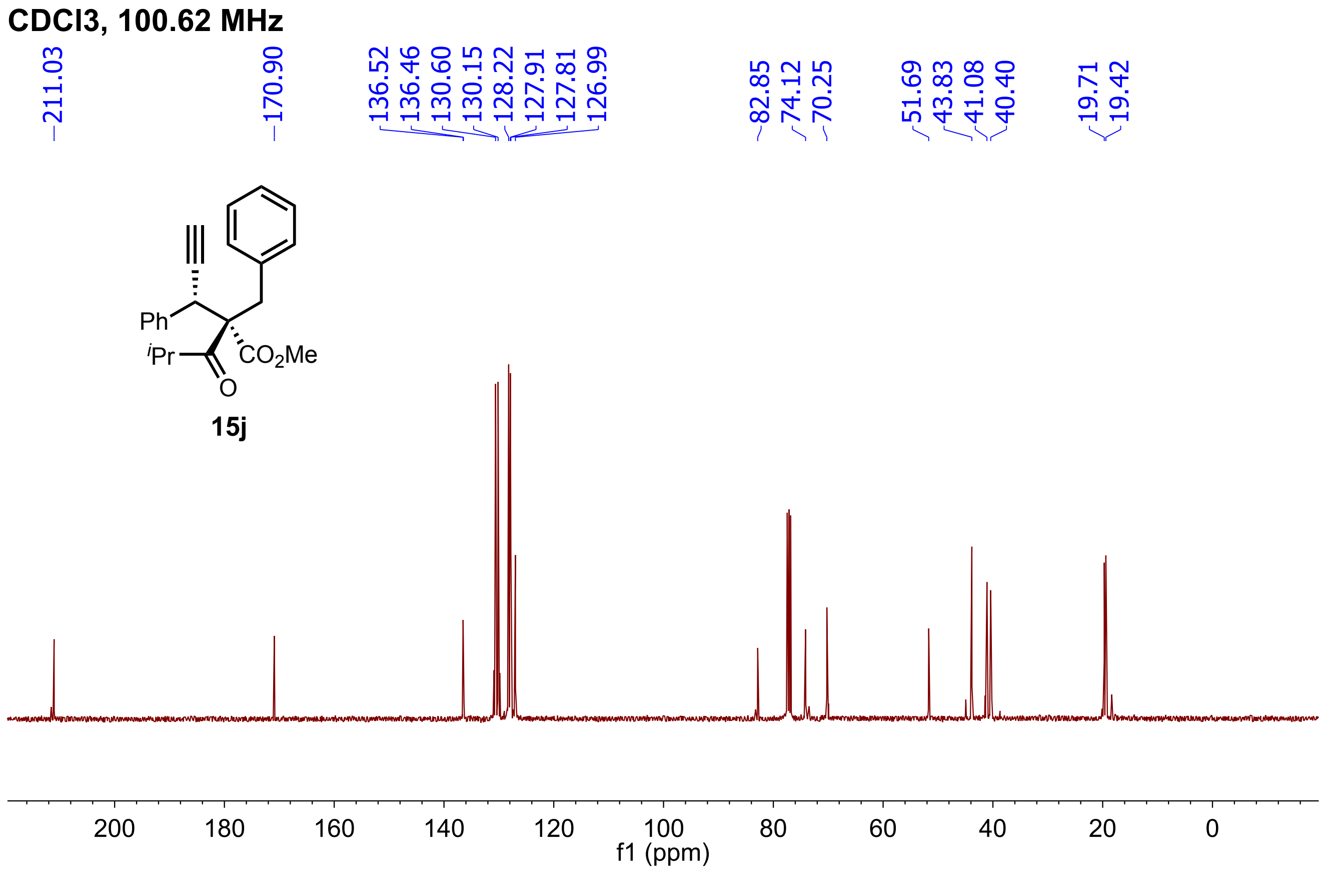

Supplementary Figure 197. ^{13}C NMR (101 MHz, $CDCl_3$) spectra for **15j**

CDCl3, 400.13 MHz

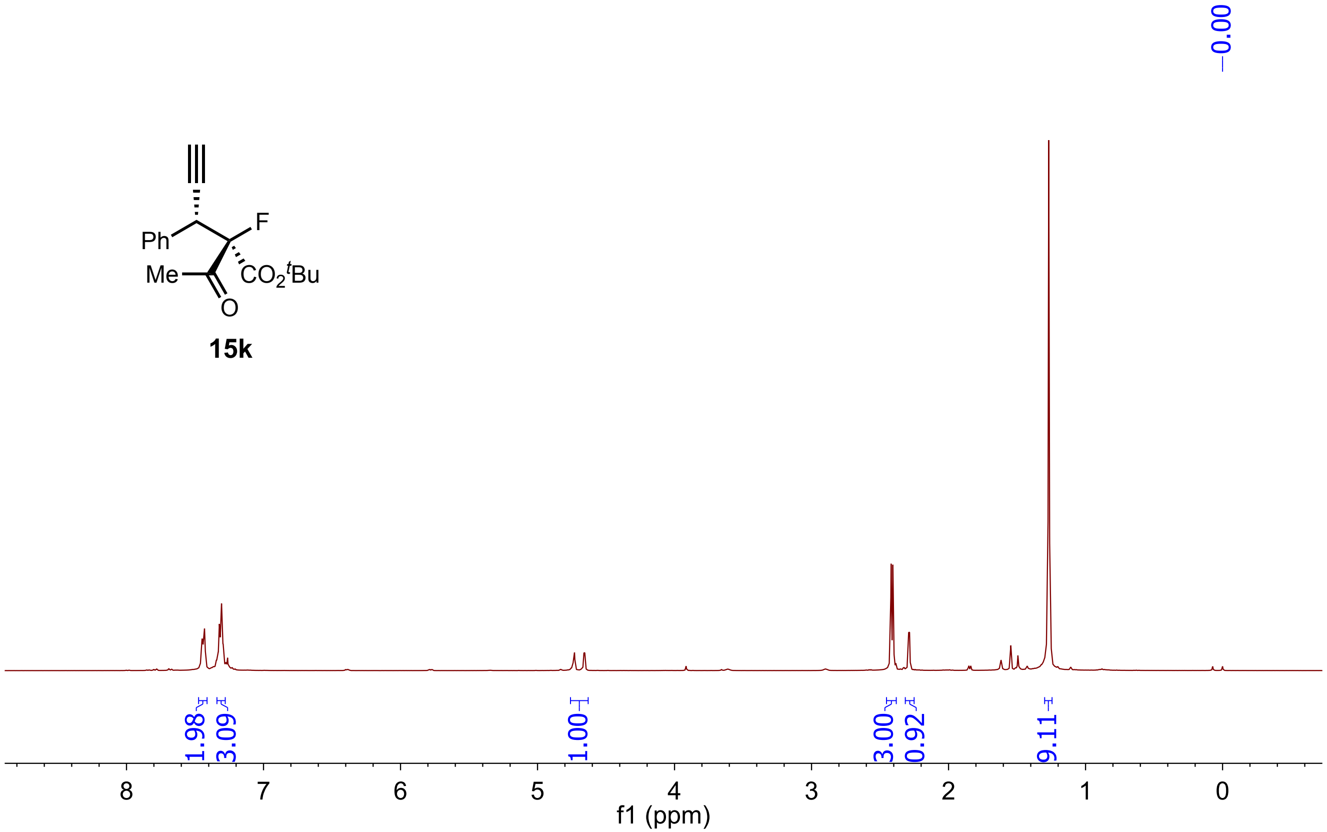

Supplementary Figure 198. ^{1}H NMR (400 MHz, $CDCl_3$) spectra for **15k**

CDCl3, 100.62 MHz

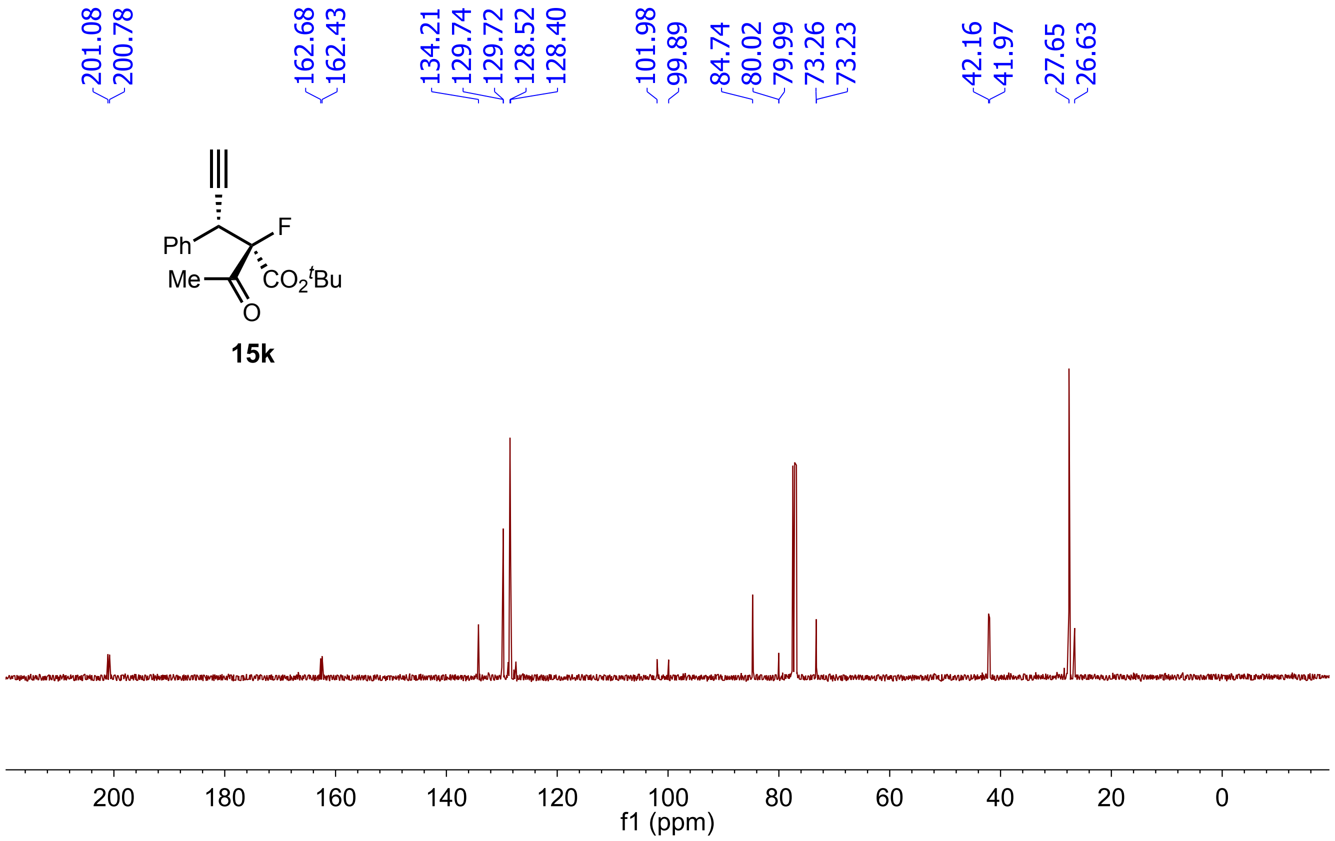

Supplementary Figure 199. ^{13}C NMR (101 MHz, $CDCl_3$) spectra for **15k**

CDCl3, 376.46 MHz

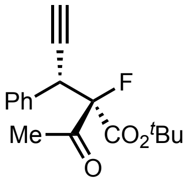

15k

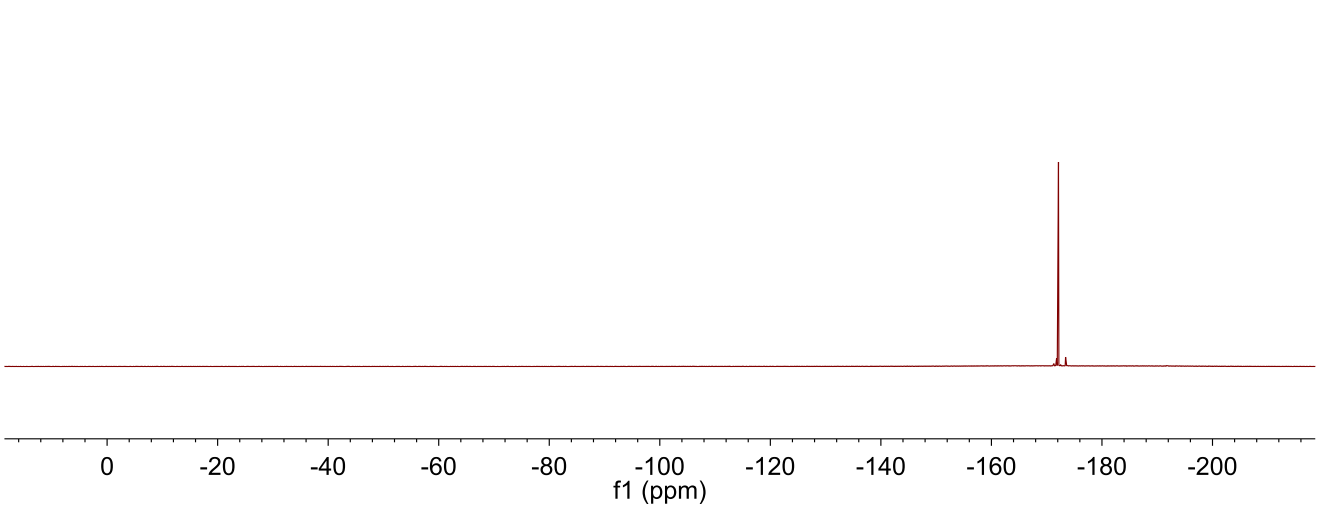

Supplementary Figure 200. ^{19}F NMR (376 MHz, $CDCl_3$) spectra for **15k**

CDCl3, 400.13 MHz

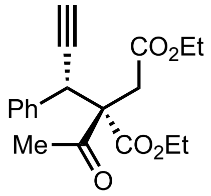

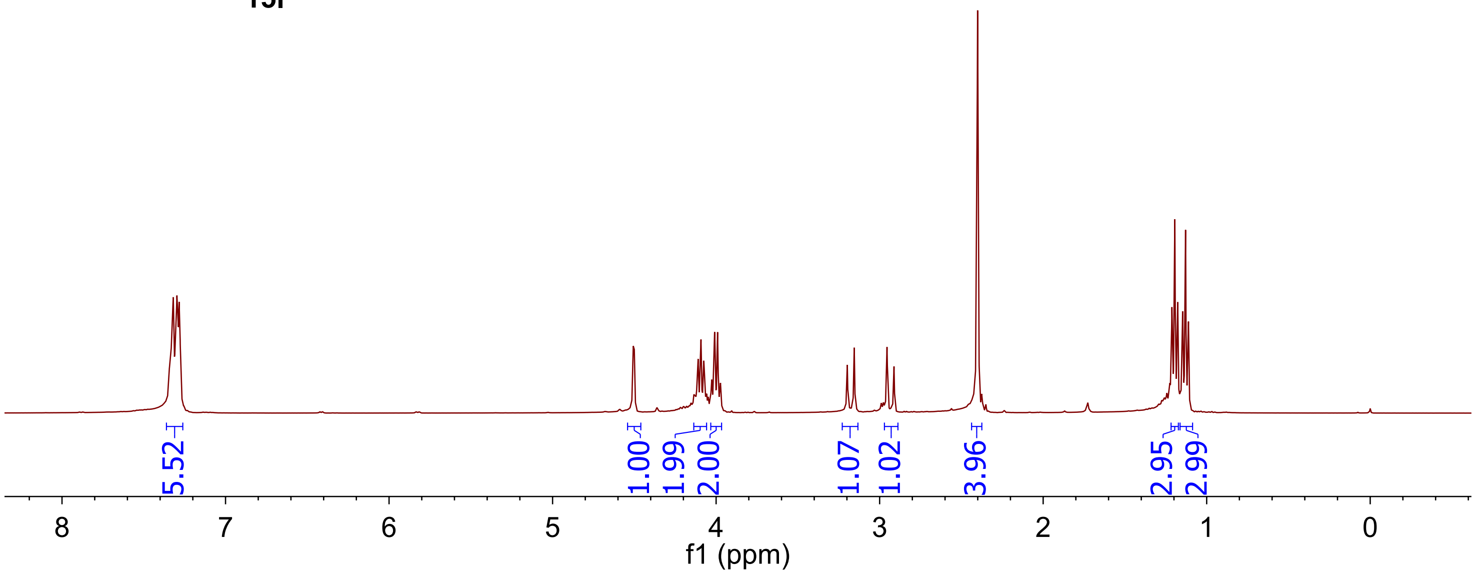

Supplementary Figure 201. ^{1}H NMR (400 MHz, $CDCl_3$) spectra for **15l**

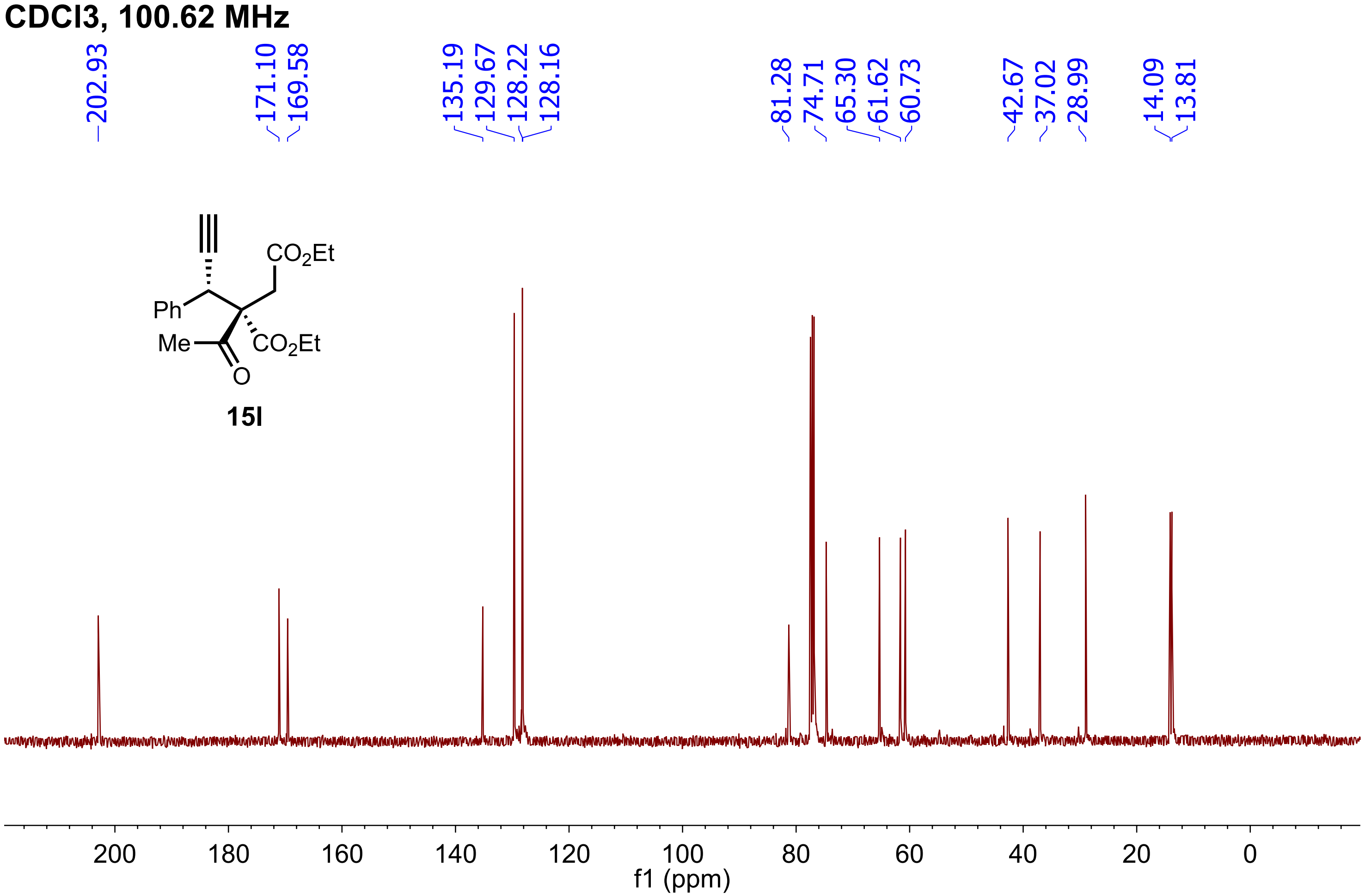

Supplementary Figure 202. ^{13}C NMR (101 MHz, $CDCl_3$) spectra for **15l**

CDCl3, 400.13 MHz

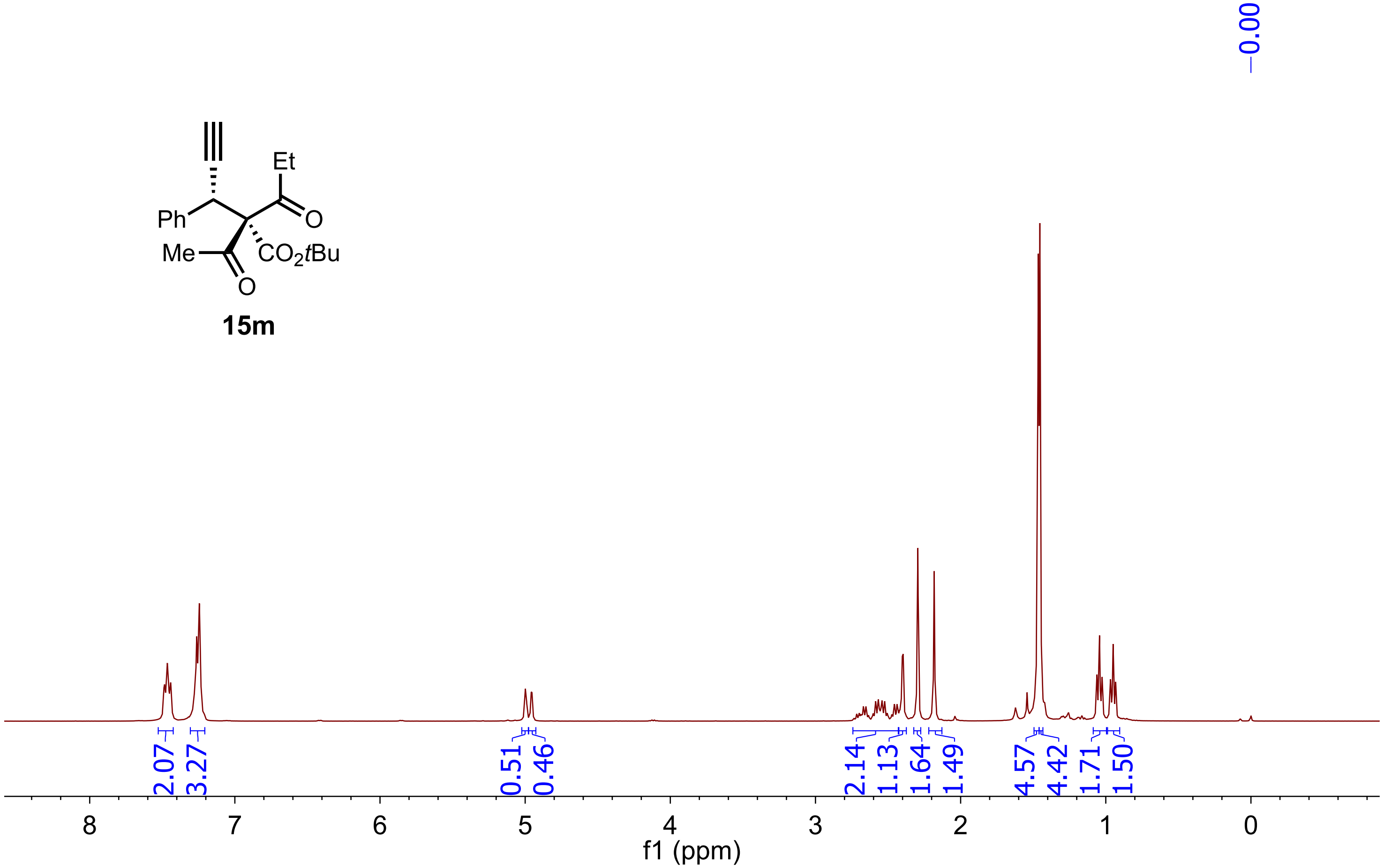

Supplementary Figure 203. ^{1}H NMR (400 MHz, $CDCl_3$) spectra for **15m**

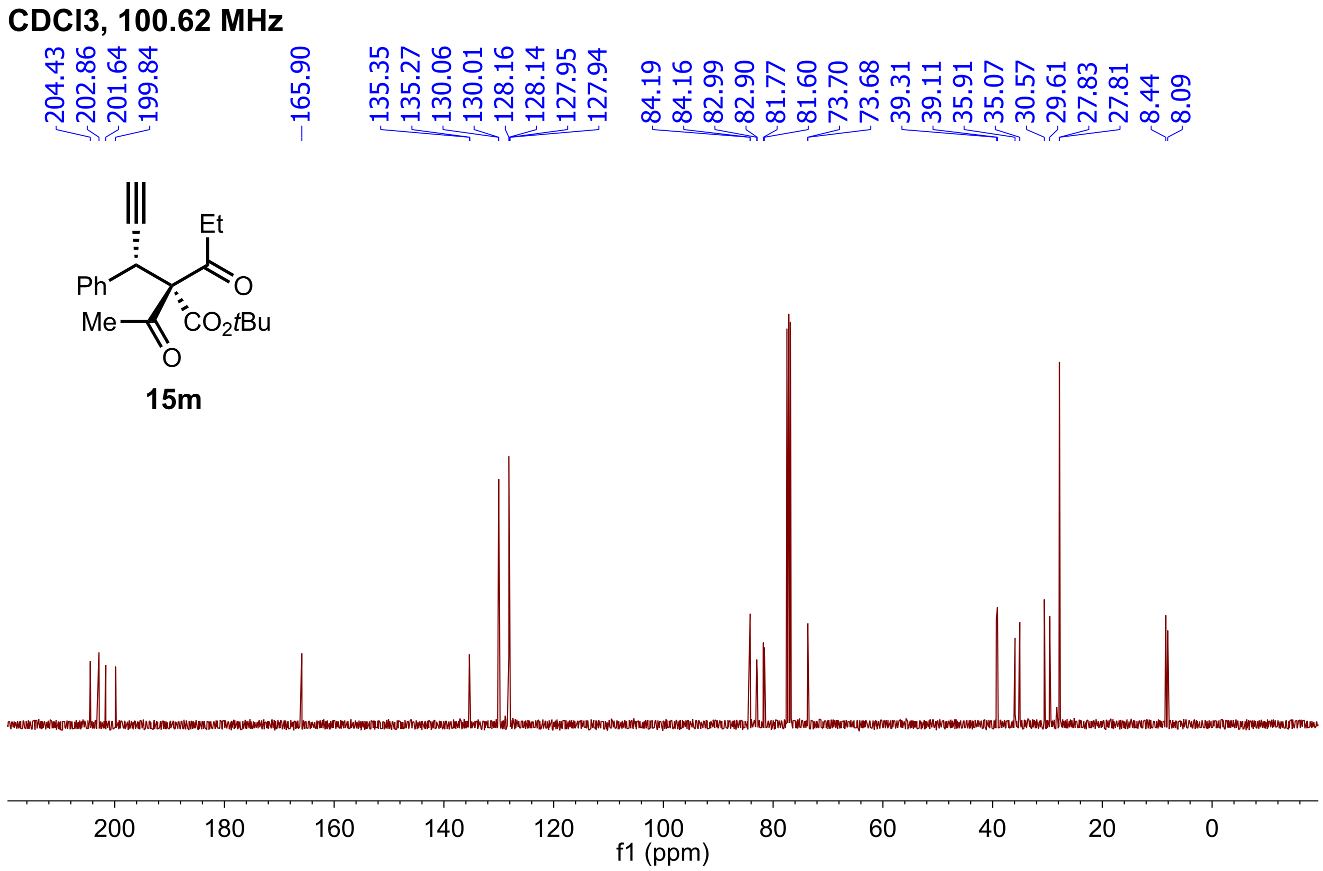

Supplementary Figure 204. ^{13}C NMR (101 MHz, $CDCl_3$) spectra for **15m**

CDCl3, 400.13 MHz

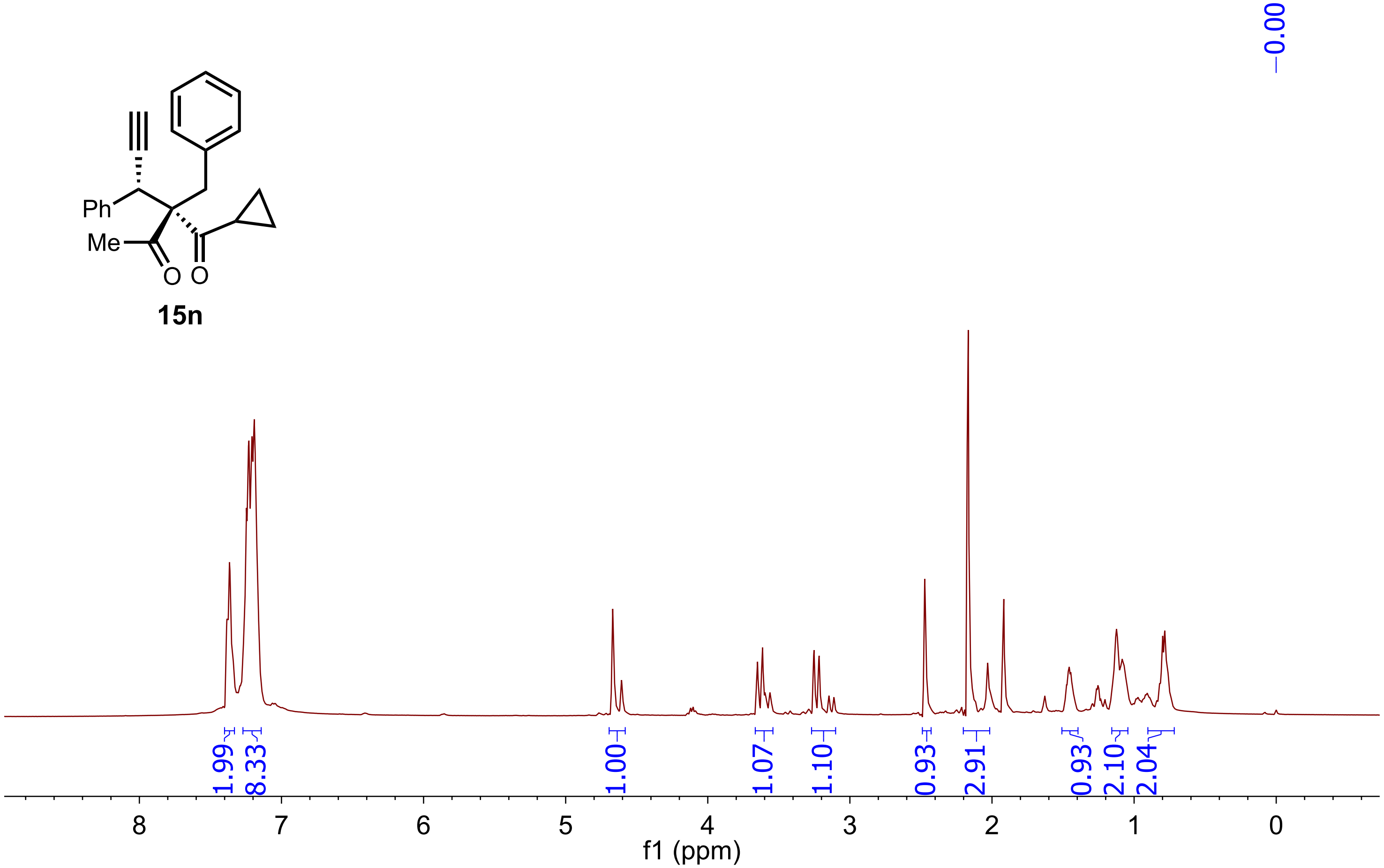

Supplementary Figure 205. ^{1}H NMR (400 MHz, $CDCl_3$) spectra for **15n**

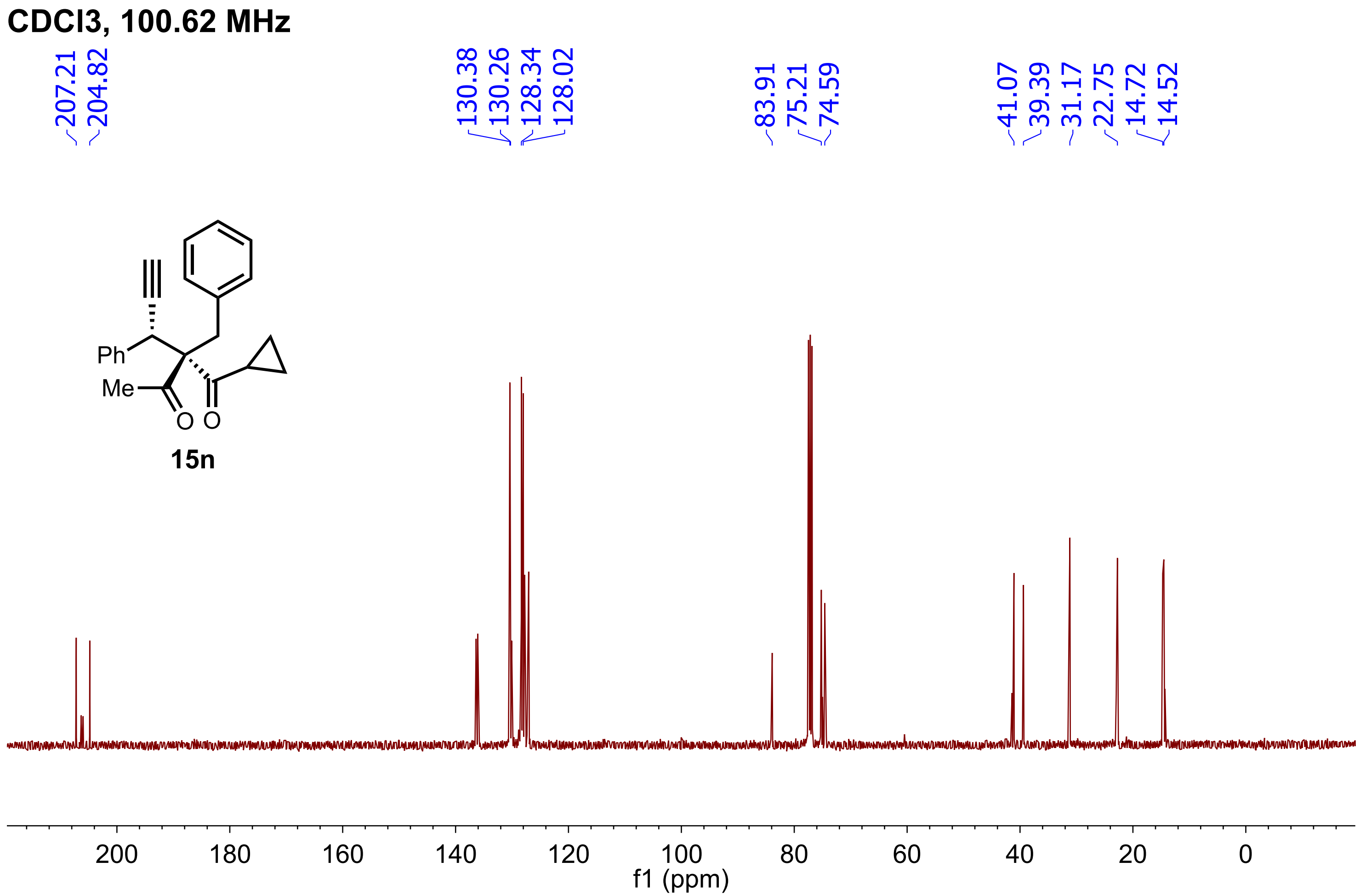

Supplementary Figure 206. ^{13}C NMR (101 MHz, $CDCl_3$) spectra for **15n**

CDCl3, 400.13 MHz

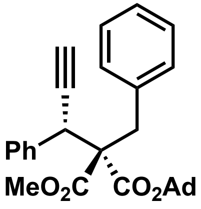

15o

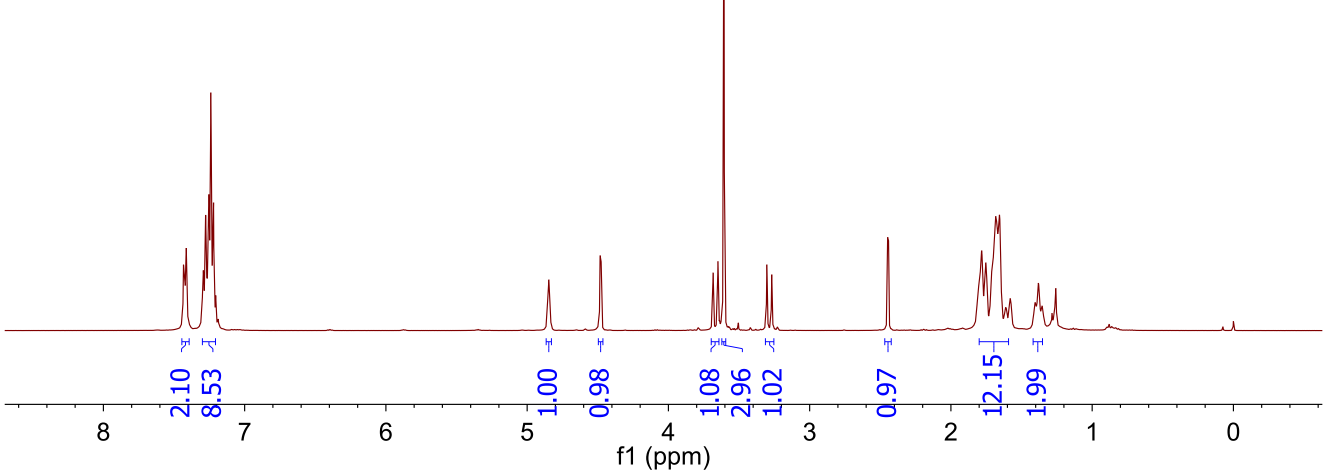

Supplementary Figure 207. ^{1}H NMR (400 MHz, $CDCl_3$) spectra for **15o**

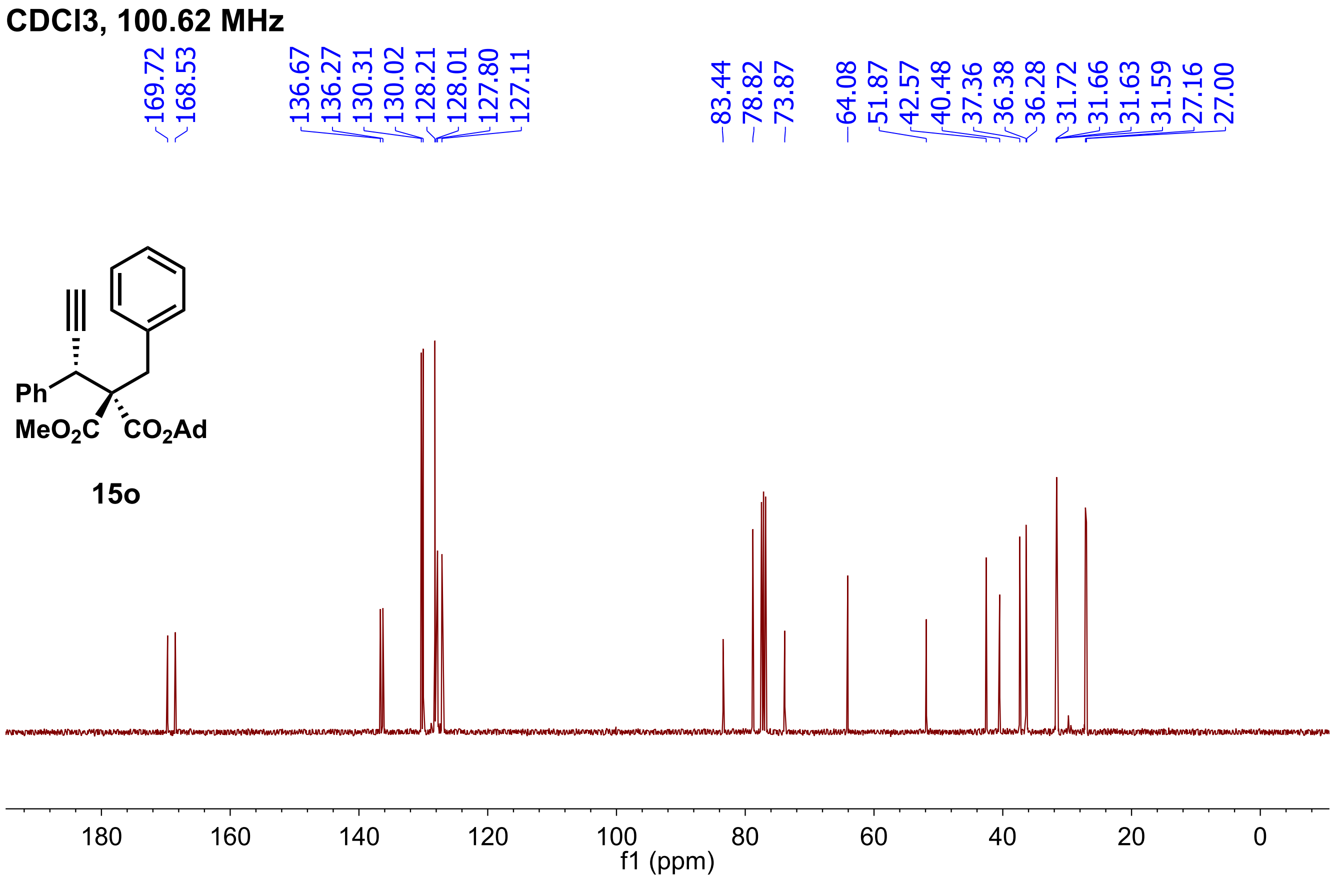

Supplementary Figure 208. ^{13}C NMR (101 MHz, $CDCl_3$) spectra for **15o**

CDCl3, 400.13 MHz

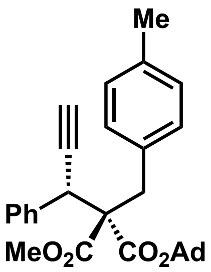

15p

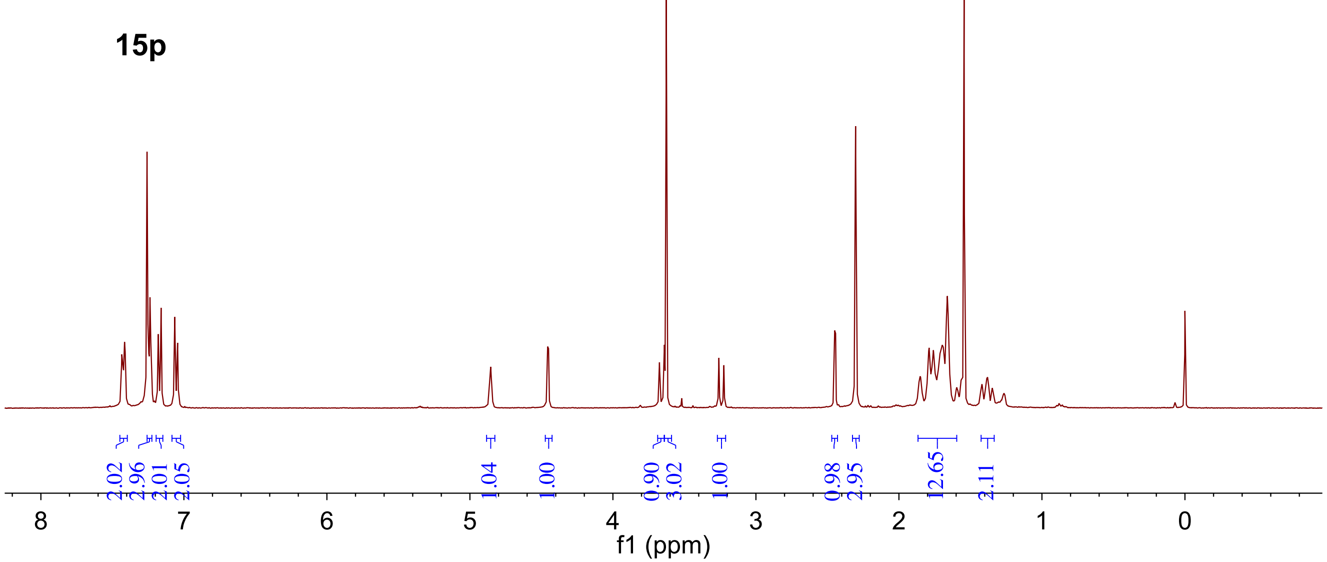

Supplementary Figure 209. ^{1}H NMR (400 MHz, $CDCl_3$) spectra for **15p**

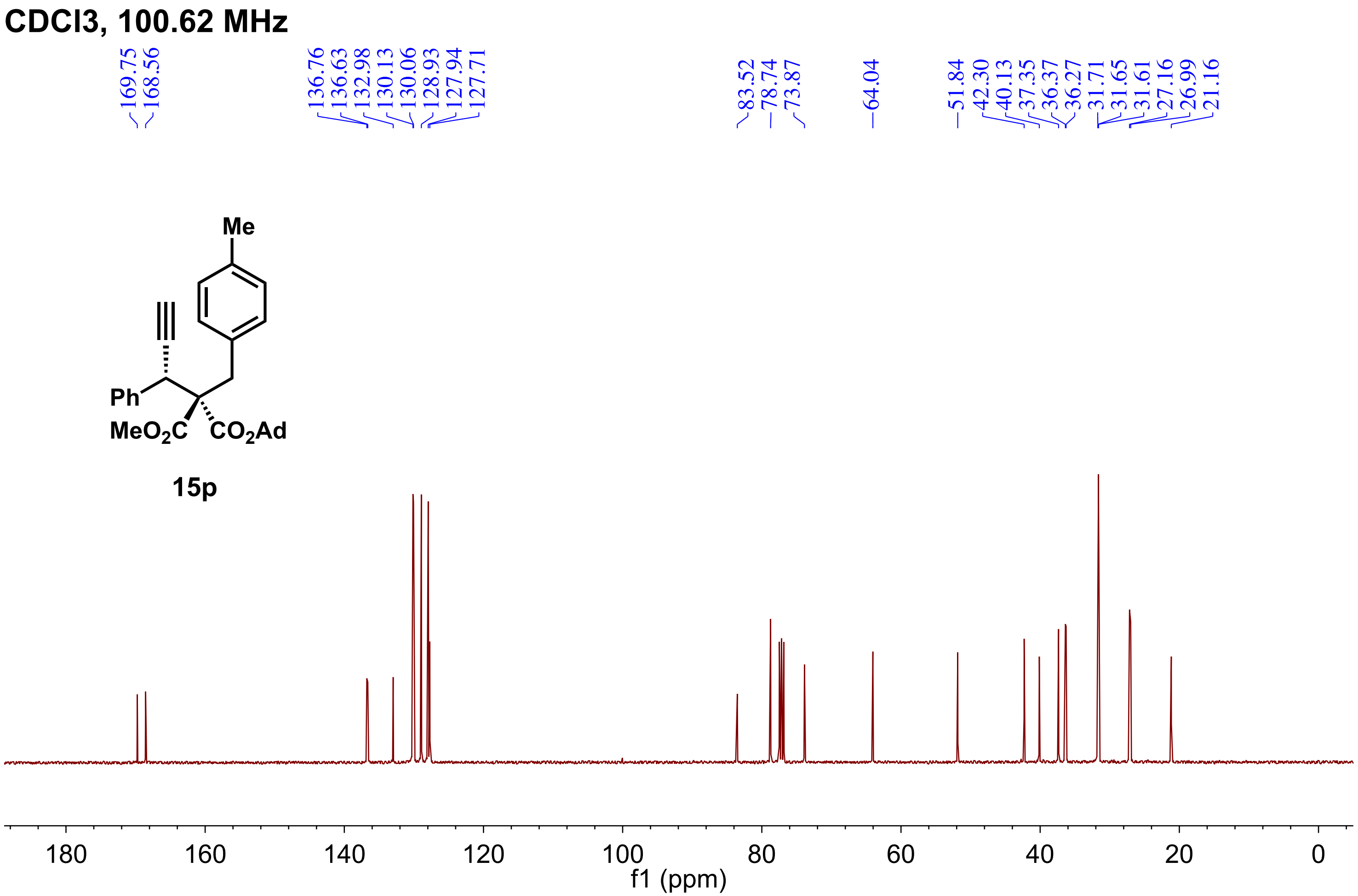

Supplementary Figure 210. ^{13}C NMR (101 MHz, $CDCl_3$) spectra for **15p**

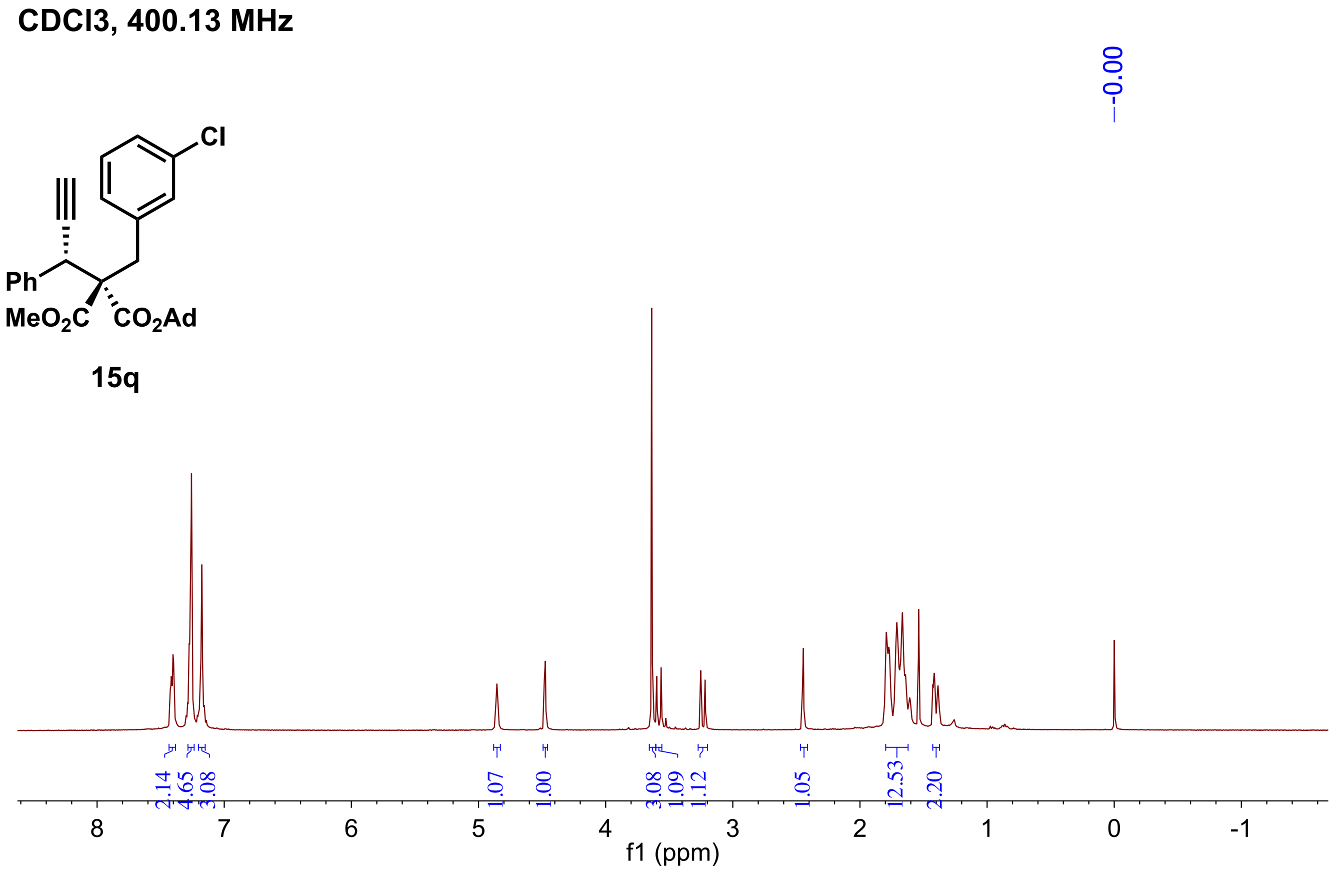

Supplementary Figure 211. ^{1}H NMR (400 MHz, $CDCl_3$) spectra for **15q**

CDCl3, 100.62 MHz

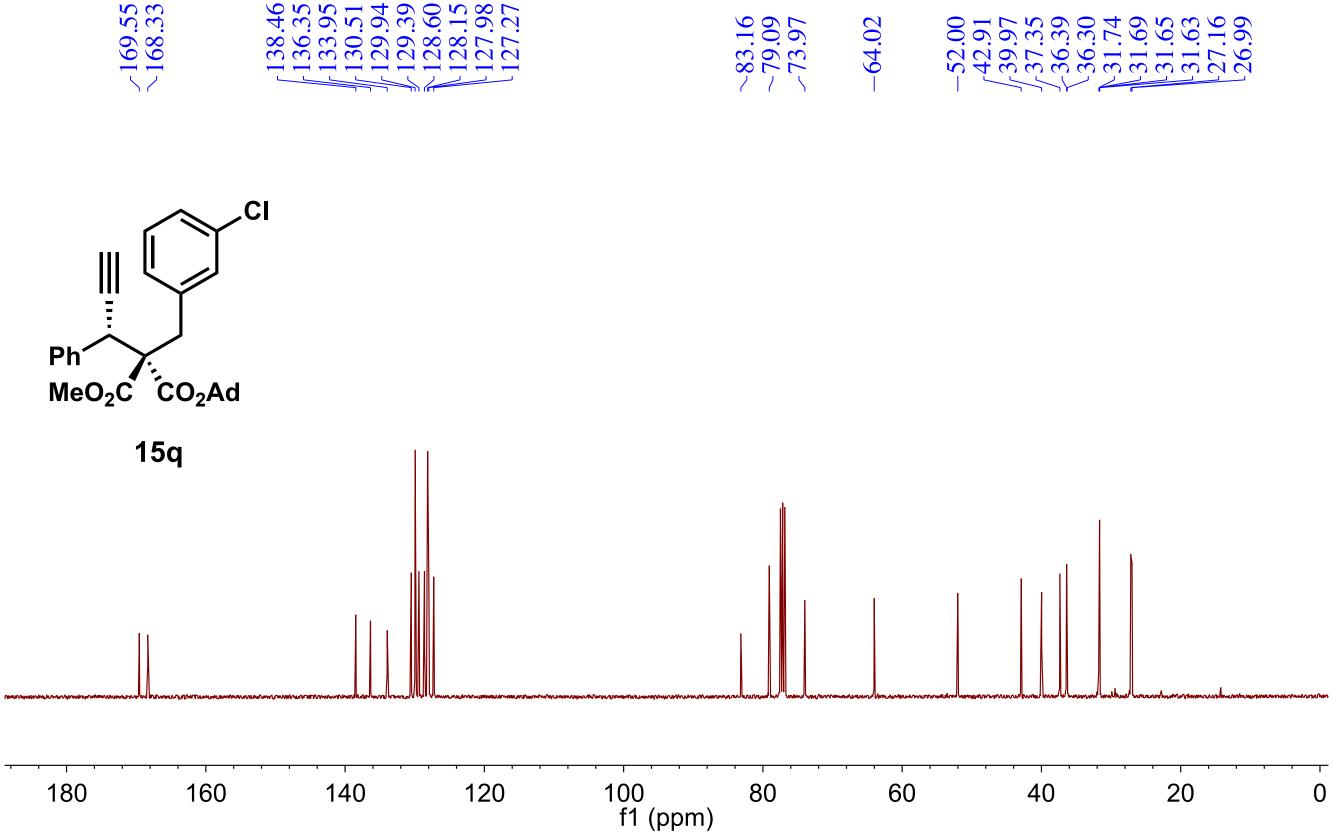

15q

Supplementary Figure 212. ^{13}C NMR (101 MHz, $CDCl_3$) spectra for **15q**

CDCl3, 400.13 MHz

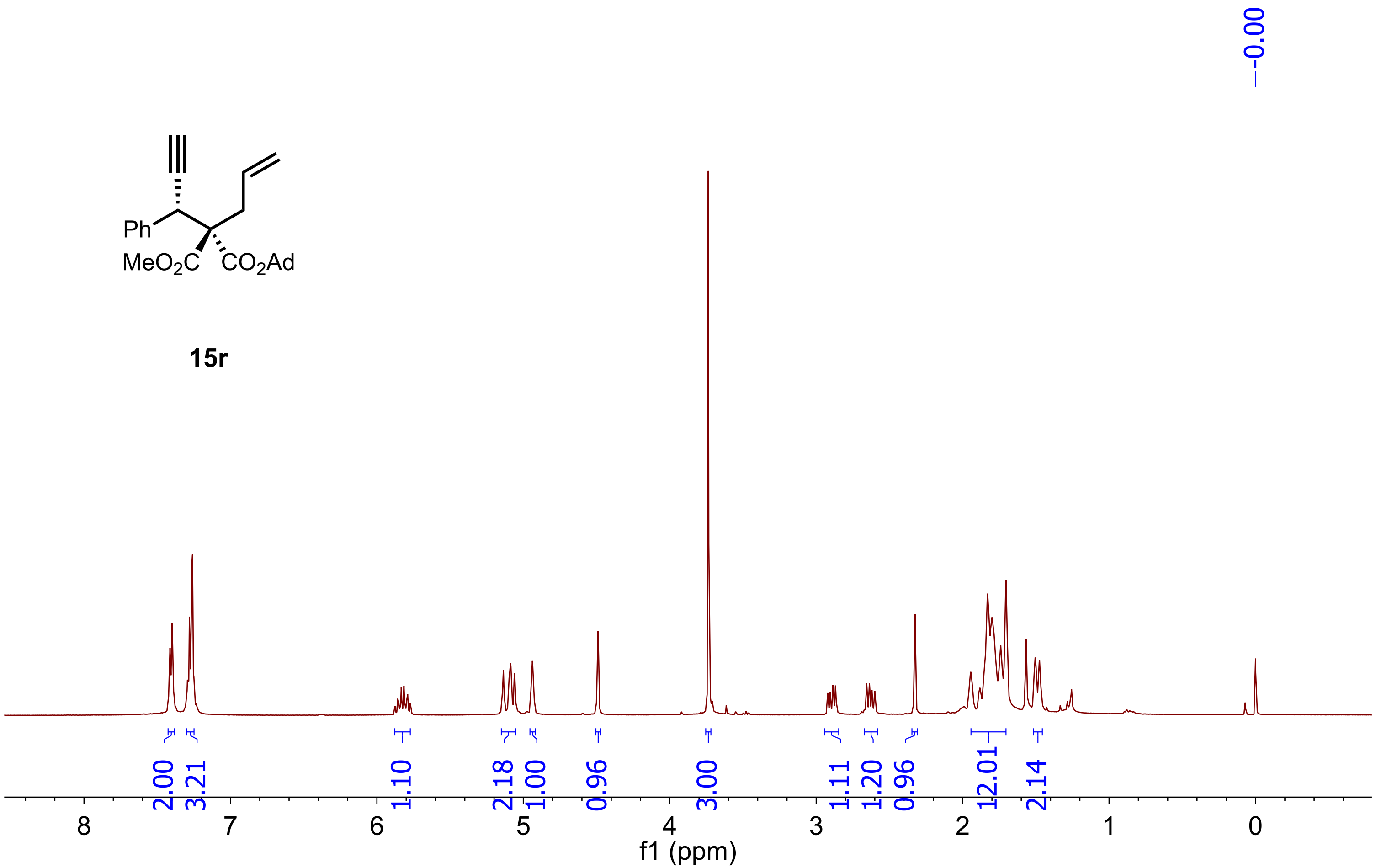

Supplementary Figure 213. ^{1}H NMR (400 MHz, $CDCl_3$) spectra for **15r**

CDCl3, 100.62 MHz

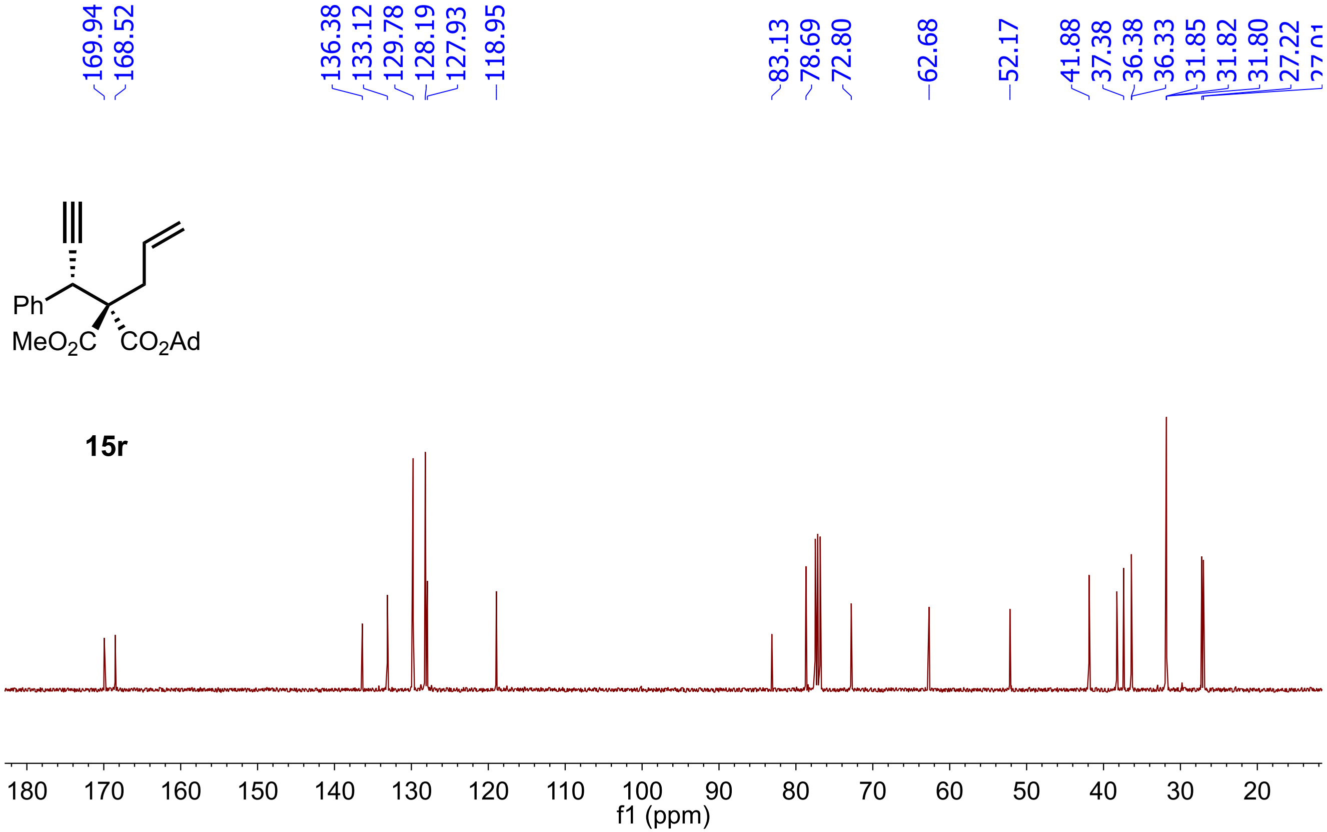

Supplementary Figure 214. ^{13}C NMR (101 MHz, $CDCl_3$) spectra for **15r**

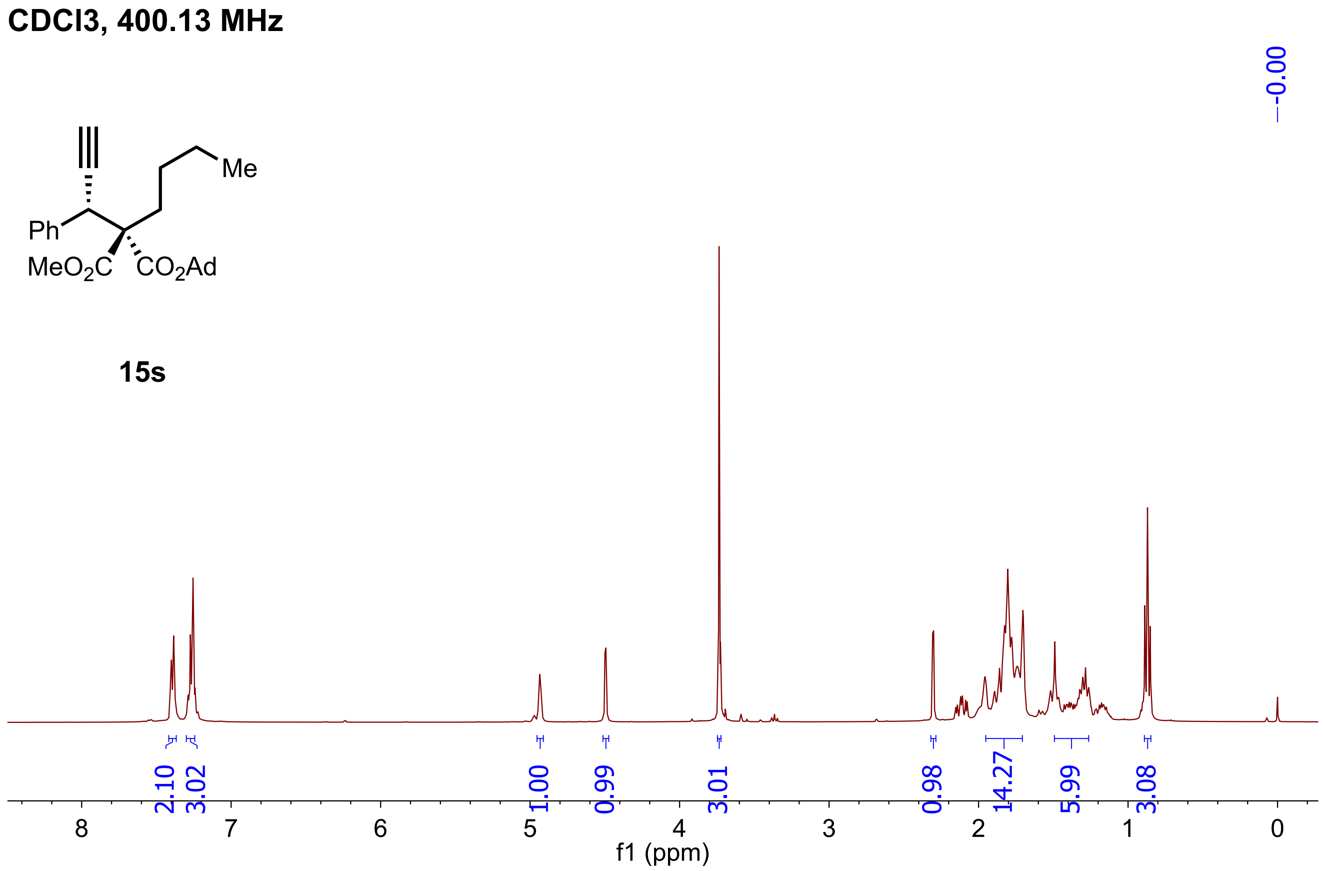

Supplementary Figure 215. ^{1}H NMR (400 MHz, $CDCl_3$) spectra for **15s**

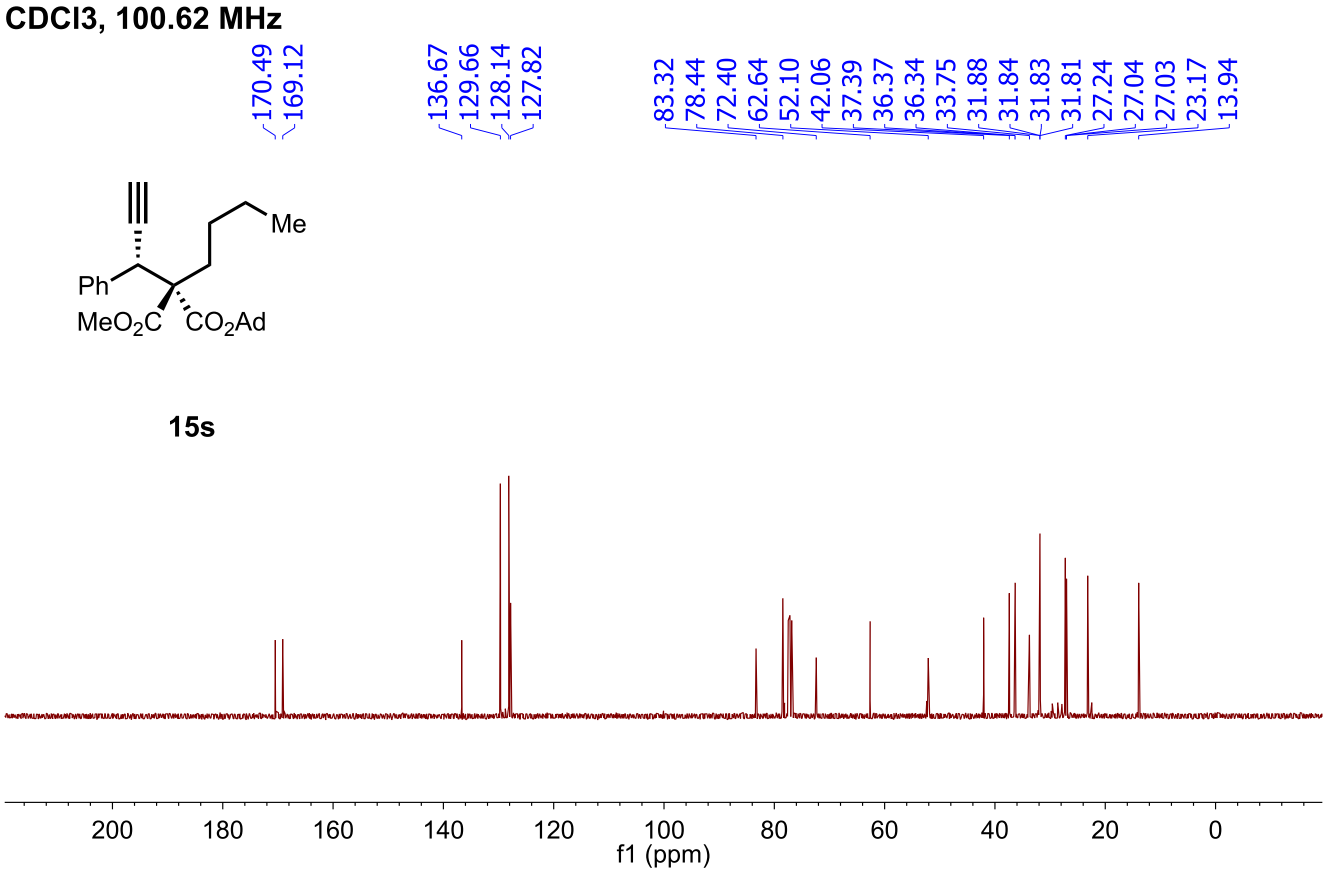

Supplementary Figure 216. ^{13}C NMR (101 MHz, $CDCl_3$) spectra for **15s**

CDCl3, 400.13 MHz

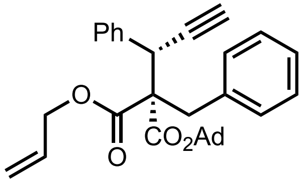

15t

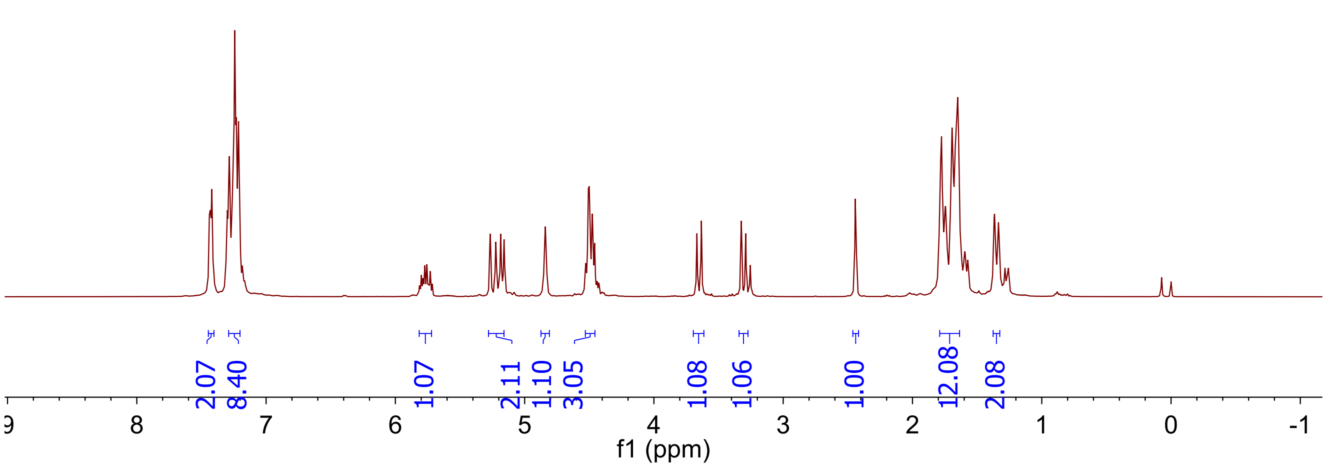

Supplementary Figure 217. ^{1}H NMR (400 MHz, $CDCl_3$) spectra for **15t**

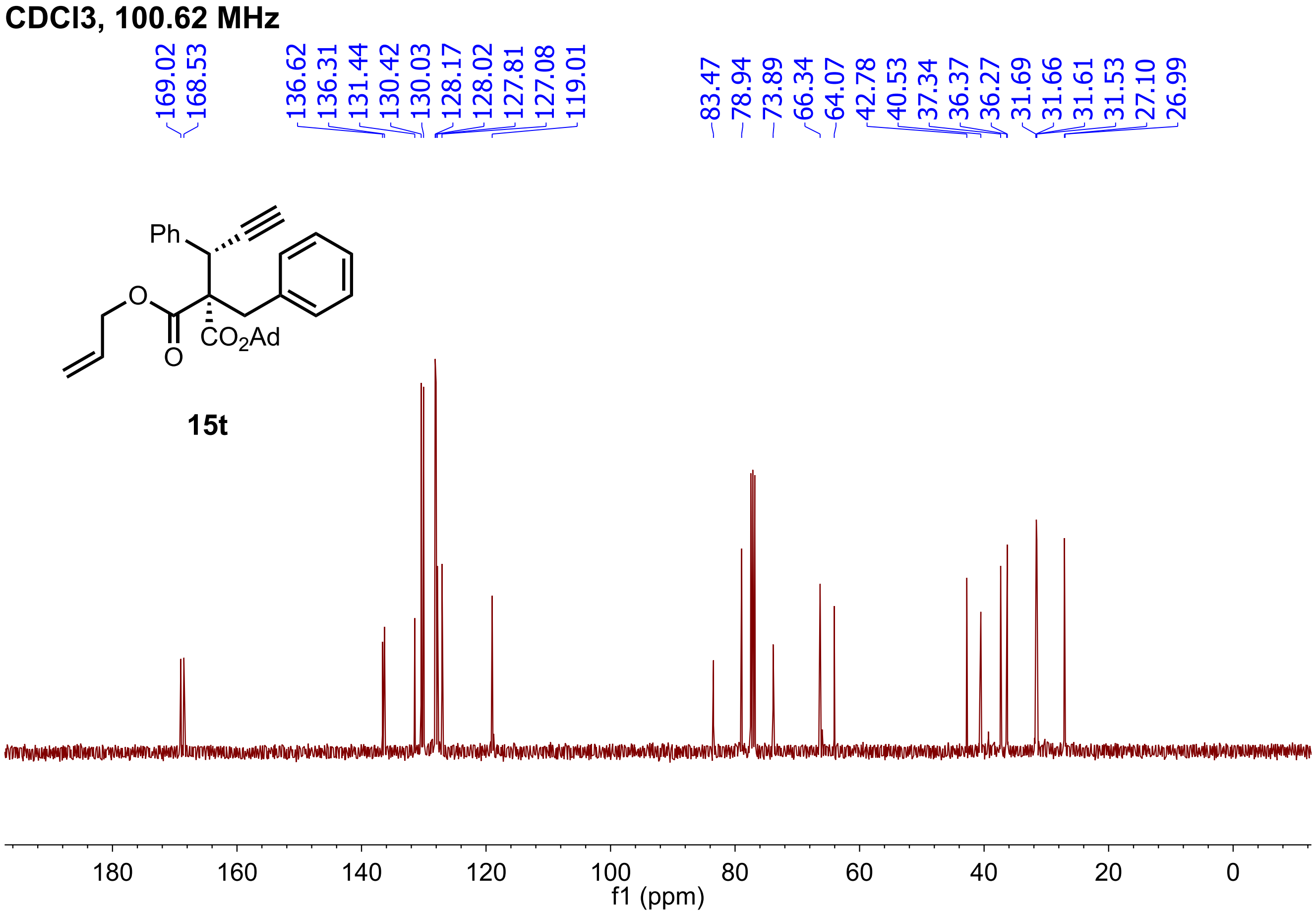

Supplementary Figure 218. ^{13}C NMR (101 MHz, $CDCl_3$) spectra for **15t**

CDCl3, 400.13 MHz

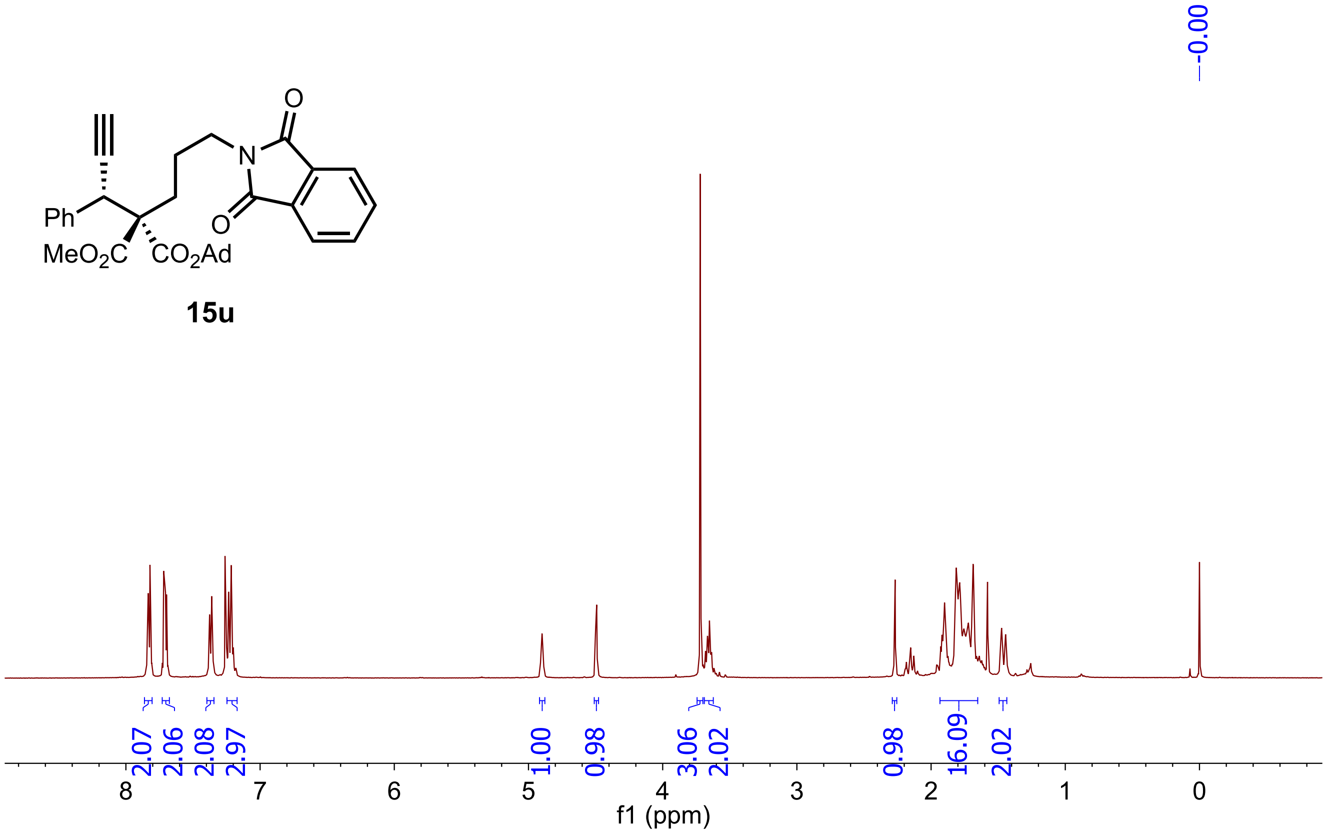

Supplementary Figure 219. ^{1}H NMR (400 MHz, $CDCl_3$) spectra for **15u**

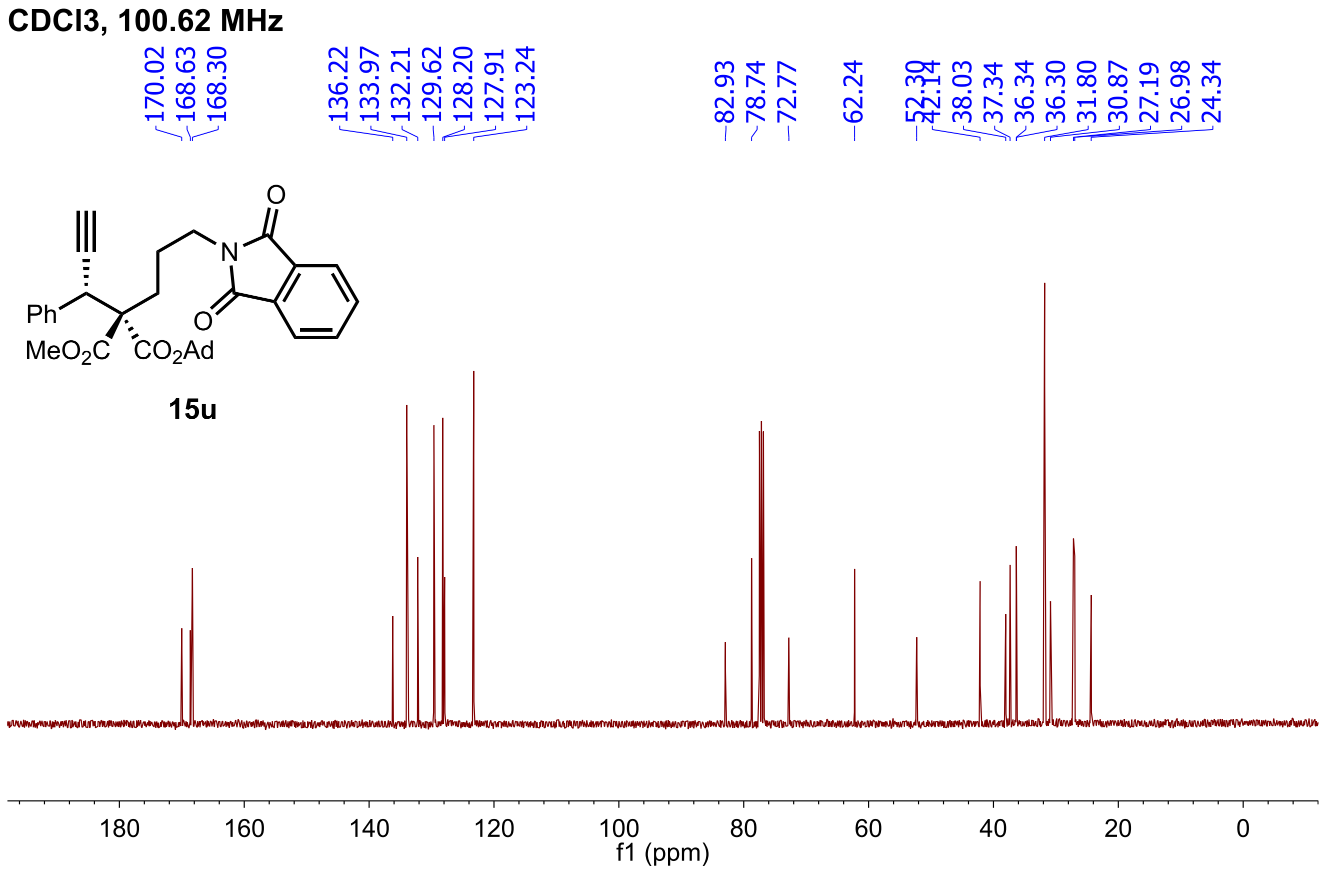

Supplementary Figure 220. ^{13}C NMR (101 MHz, $CDCl_3$) spectra for **15u**

CDCl3, 400.13 MHz

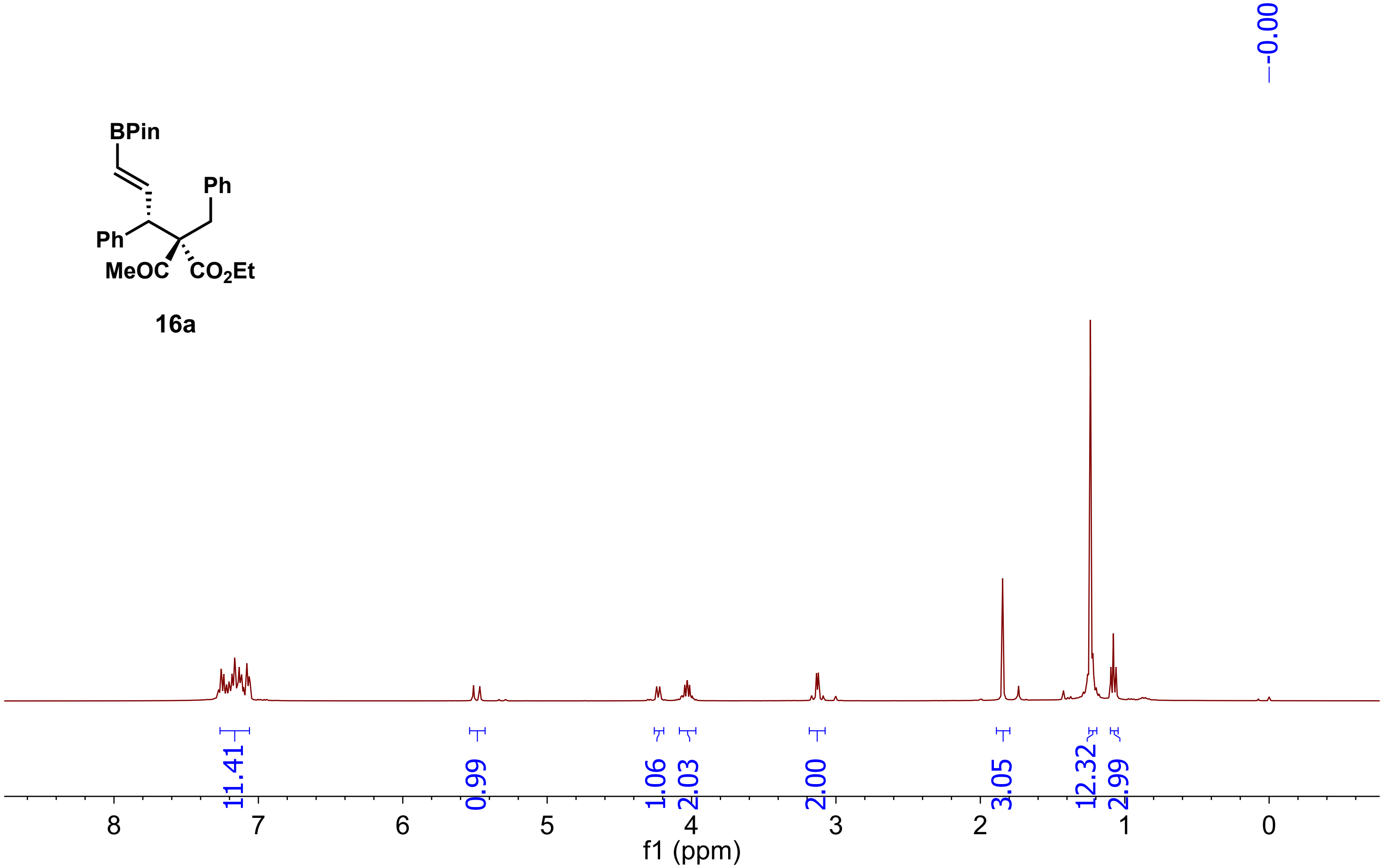

Supplementary Figure 221. ^{1}H NMR (400 MHz, $CDCl_3$) spectra for **16a**

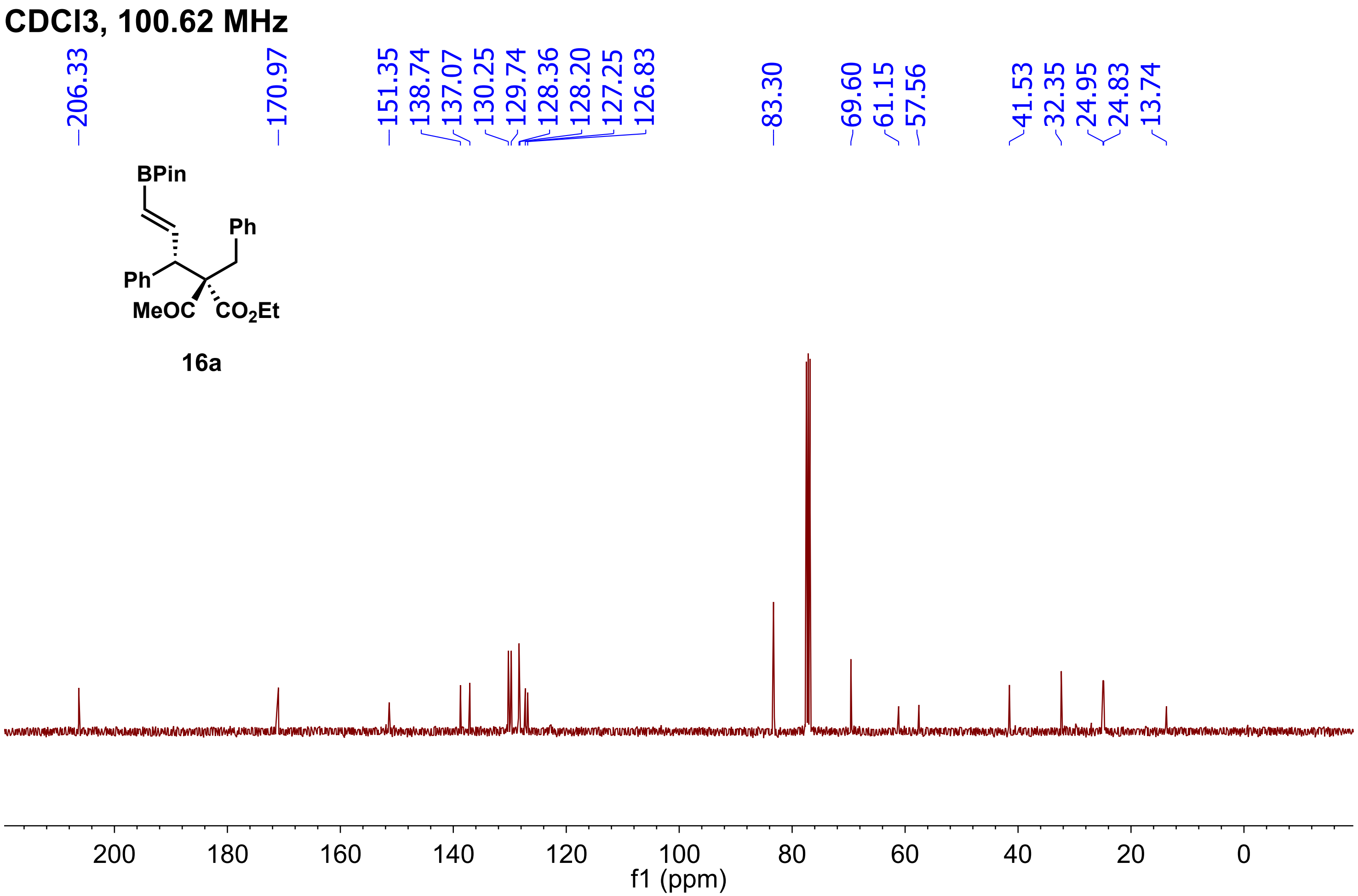

Supplementary Figure 222. ^{13}C NMR (101 MHz, $CDCl_3$) spectra for **16a**

CDCl3, 400.13 MHz

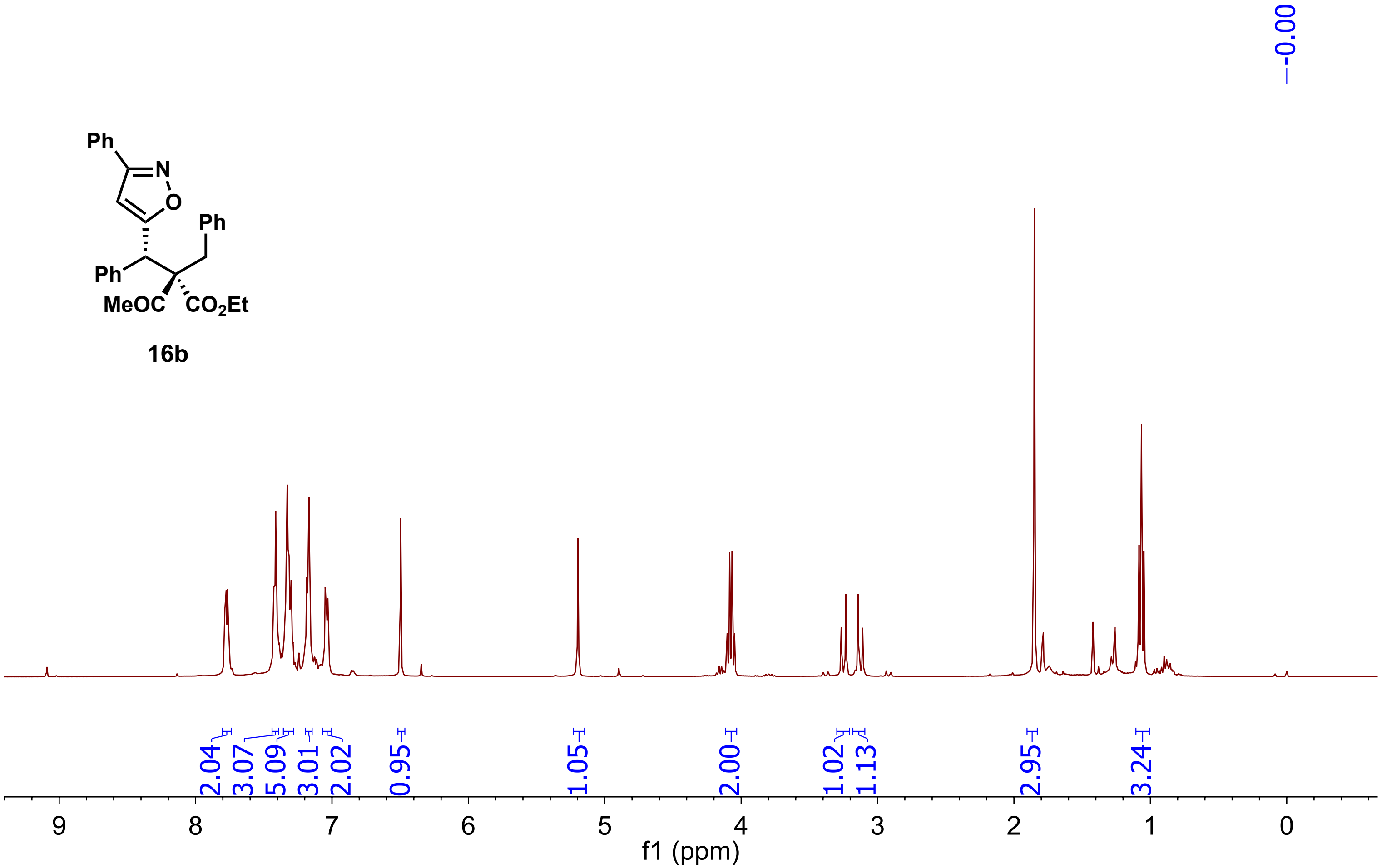

Supplementary Figure 223. ^{1}H NMR (400 MHz, $CDCl_3$) spectra for **16b**

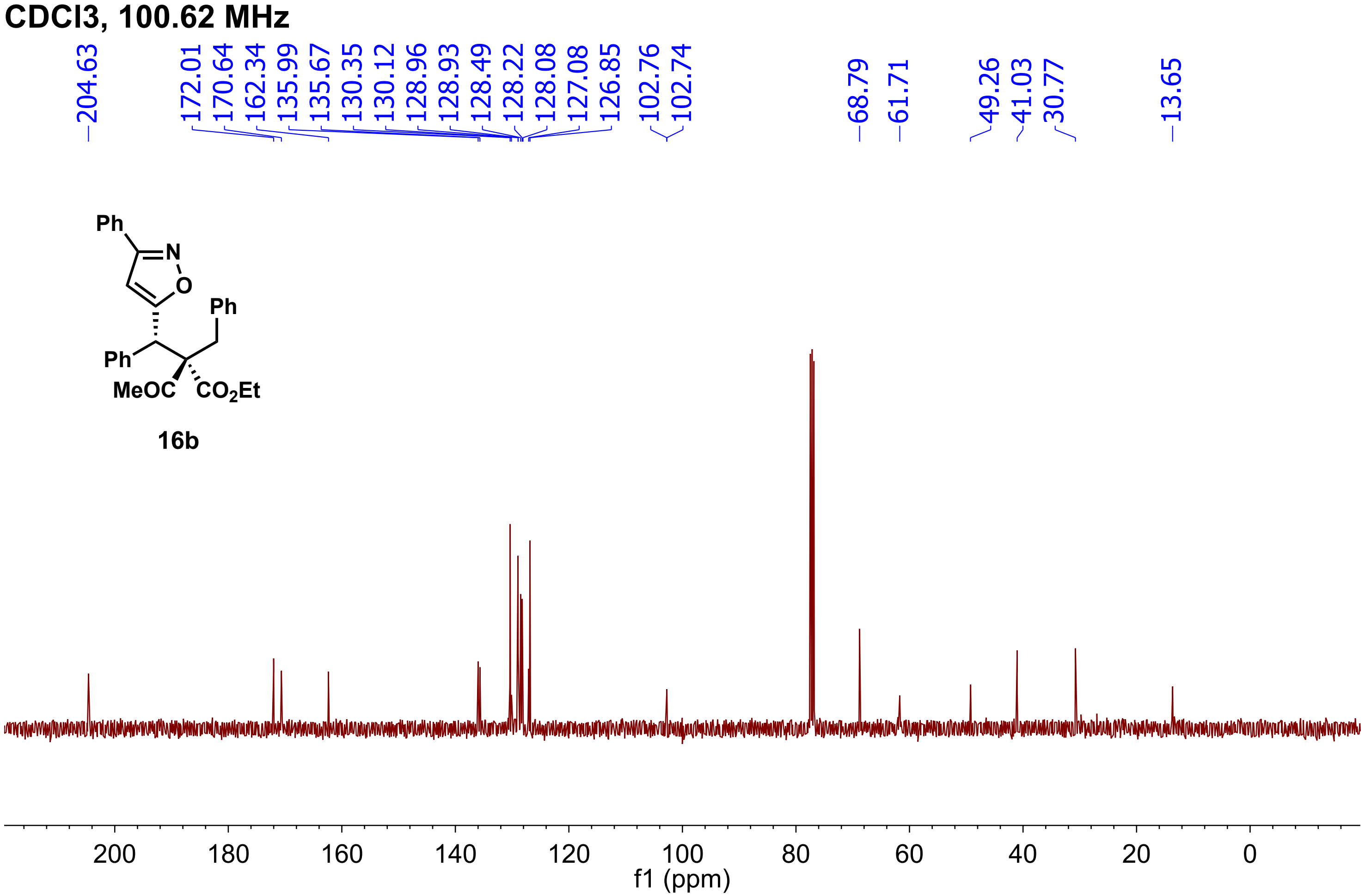

Supplementary Figure 224. ^{13}C NMR (101 MHz, $CDCl_3$) spectra for **16b**

CDCl3, 400.13 MHz

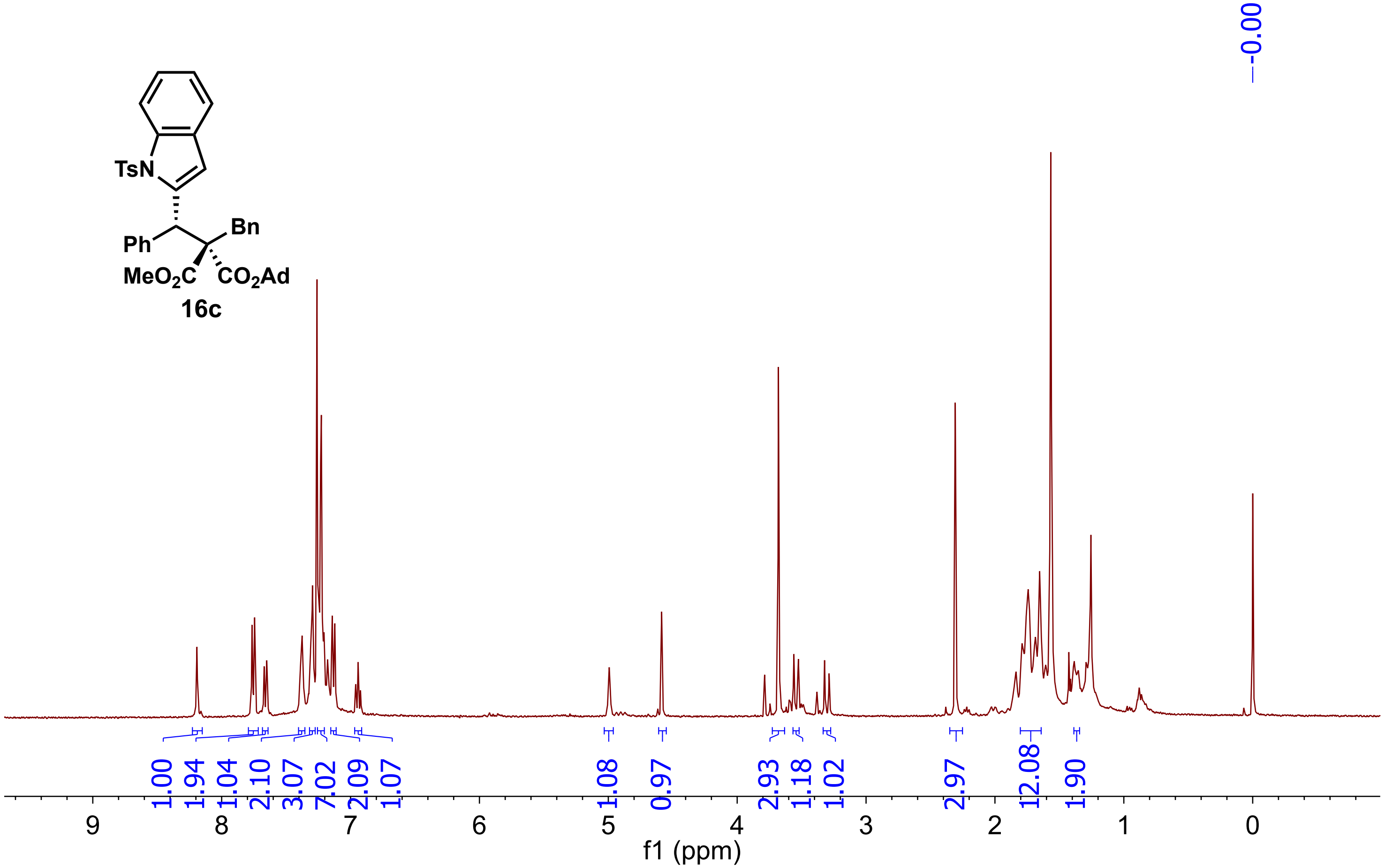

Supplementary Figure 225. ^{1}H NMR (400 MHz, $CDCl_3$) spectra for **16c**

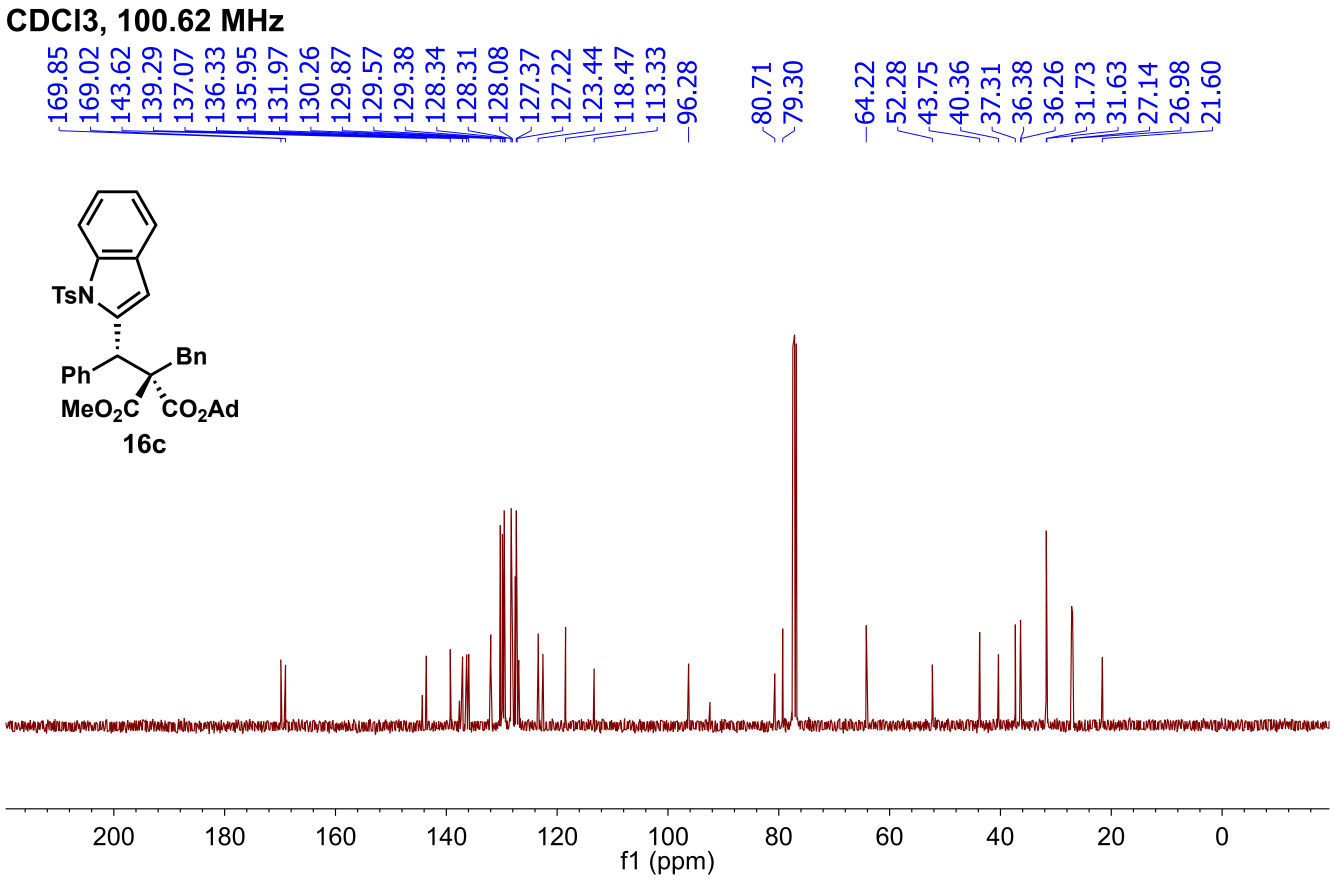

Supplementary Figure 226. ^{13}C NMR (101 MHz, $CDCl_3$) spectra for **16c**

CDCl3, 400.13 MHz

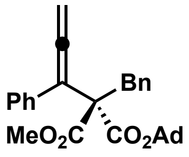

16d

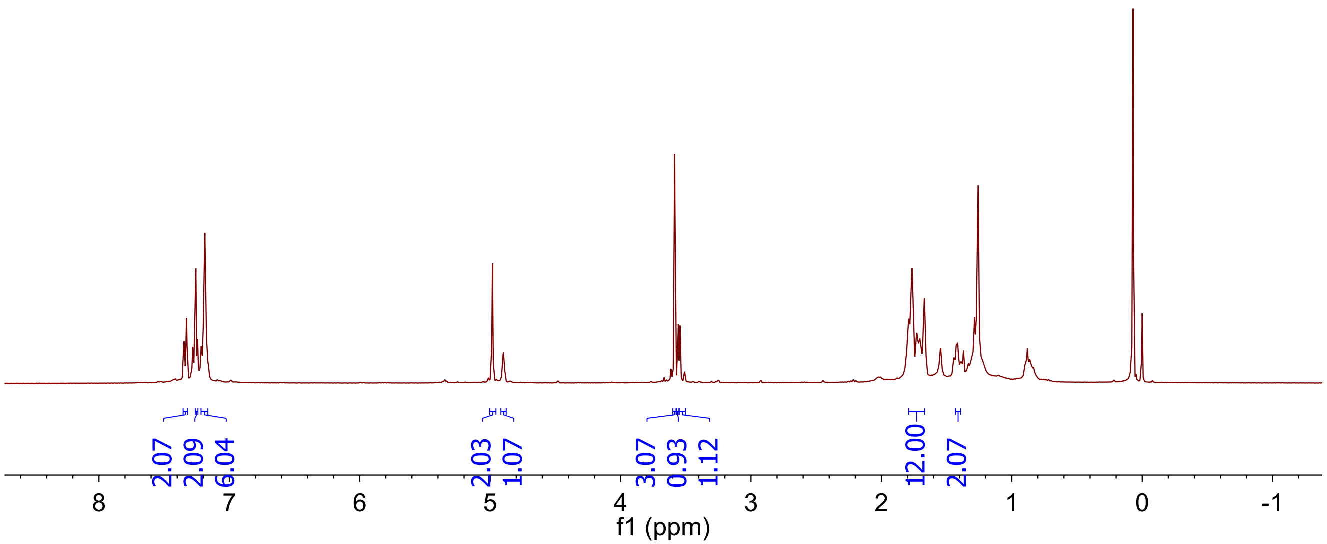

Supplementary Figure 227. ^{1}H NMR (400 MHz, $CDCl_3$) spectra for **16d**

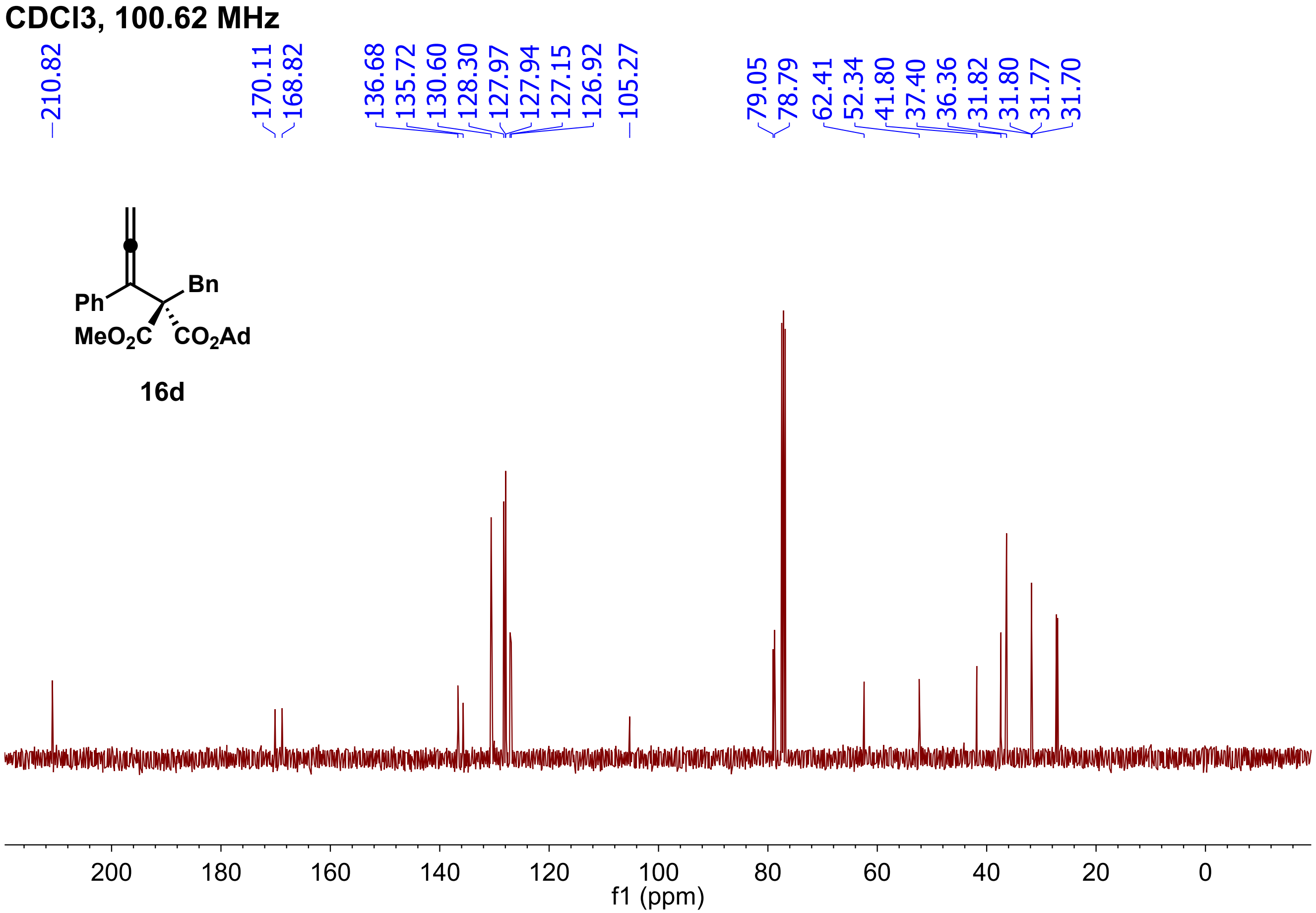

Supplementary Figure 228. ^{13}C NMR (101 MHz, $CDCl_3$) spectra for **16d**

CDCl3, 400.13 MHz

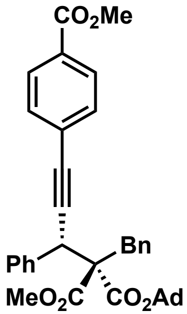

16e

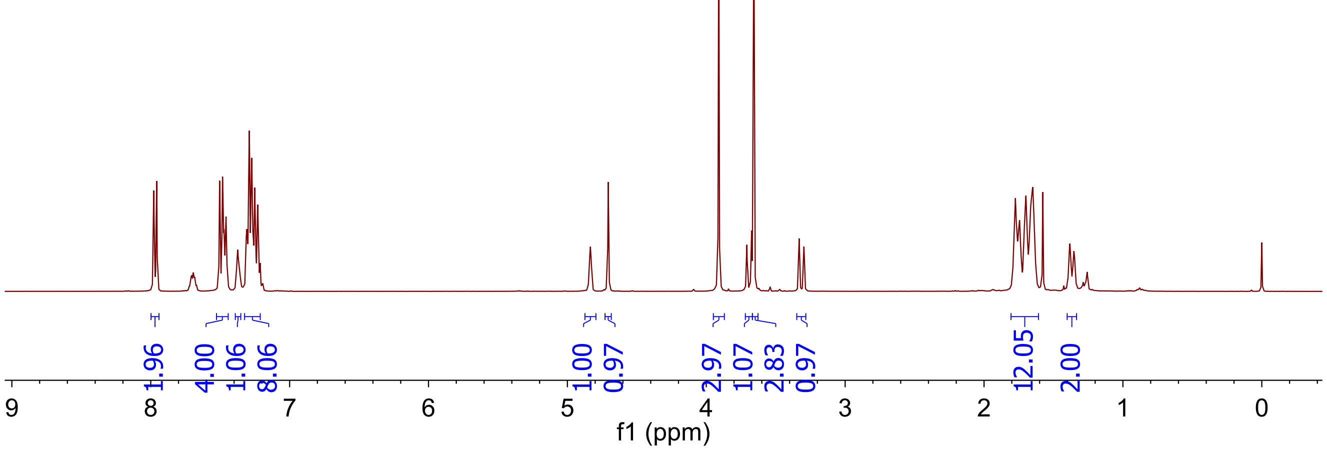

Supplementary Figure 229. ^{1}H NMR (400 MHz, $CDCl_3$) spectra for **16e**

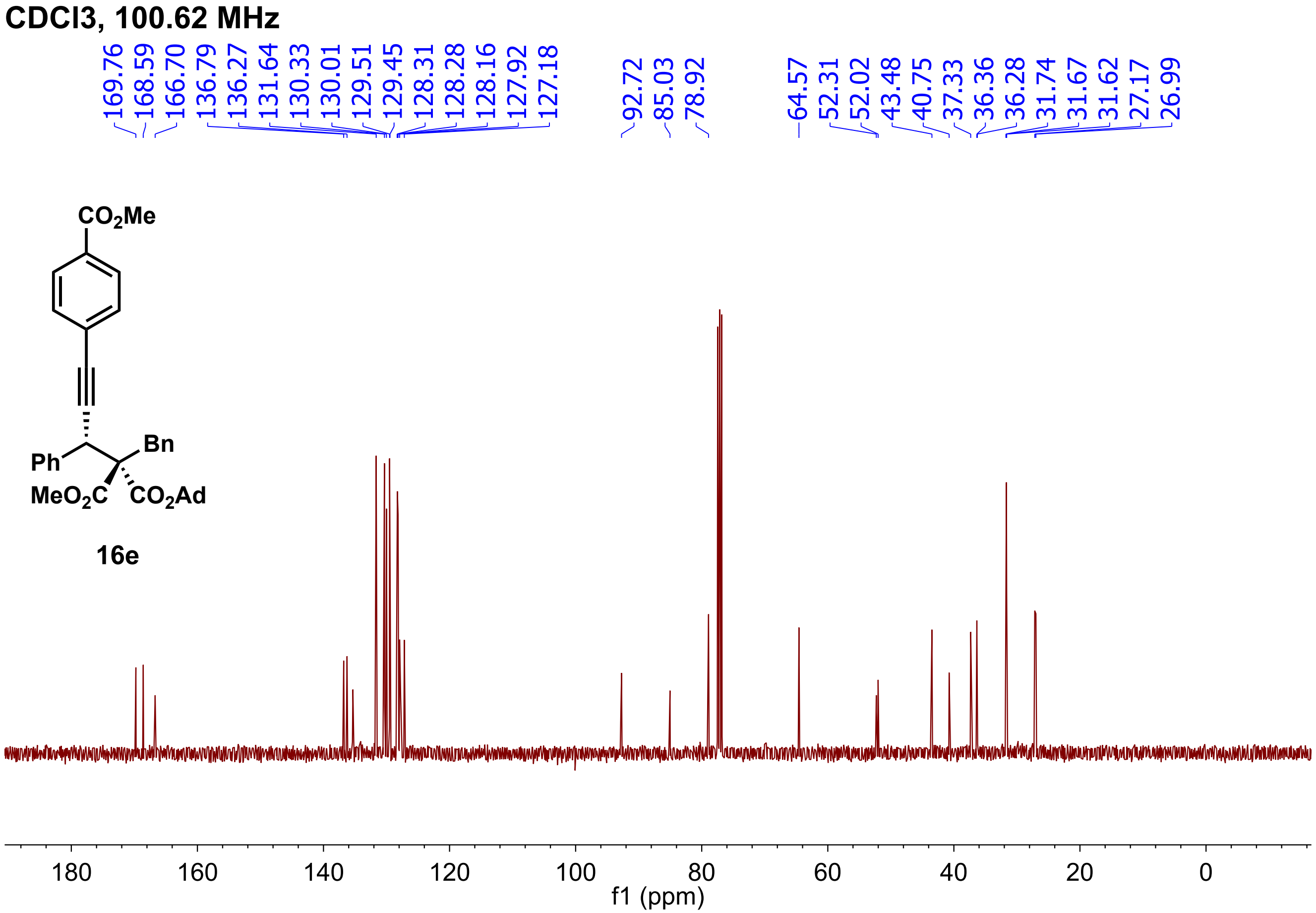

Supplementary Figure 230. ^{13}C NMR (101 MHz, $CDCl_3$) spectra for **16e**

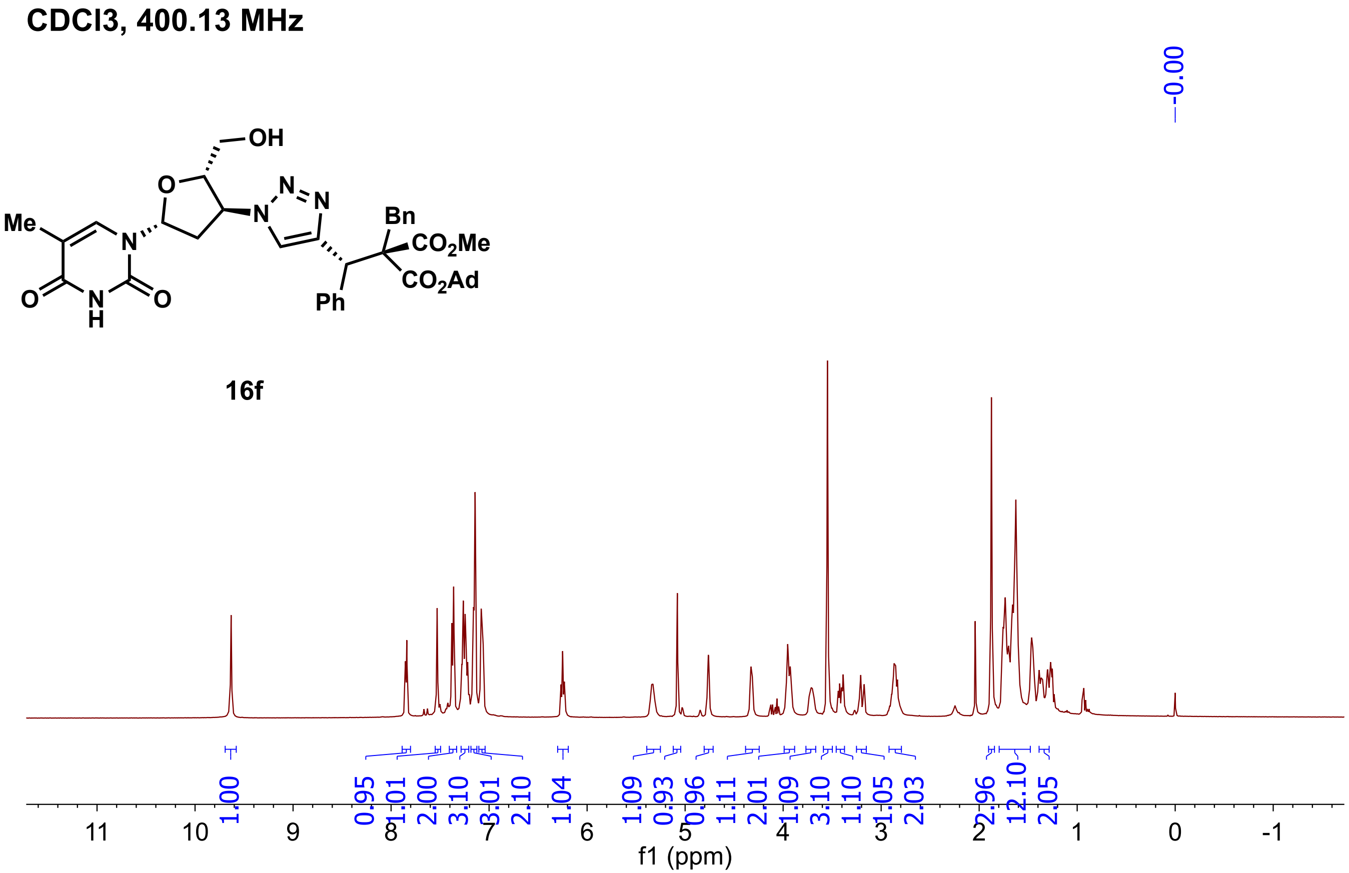

Supplementary Figure 231. ^{1}H NMR (400 MHz, $CDCl_3$) spectra for **16f**

CDCl3, 100.62 MHz

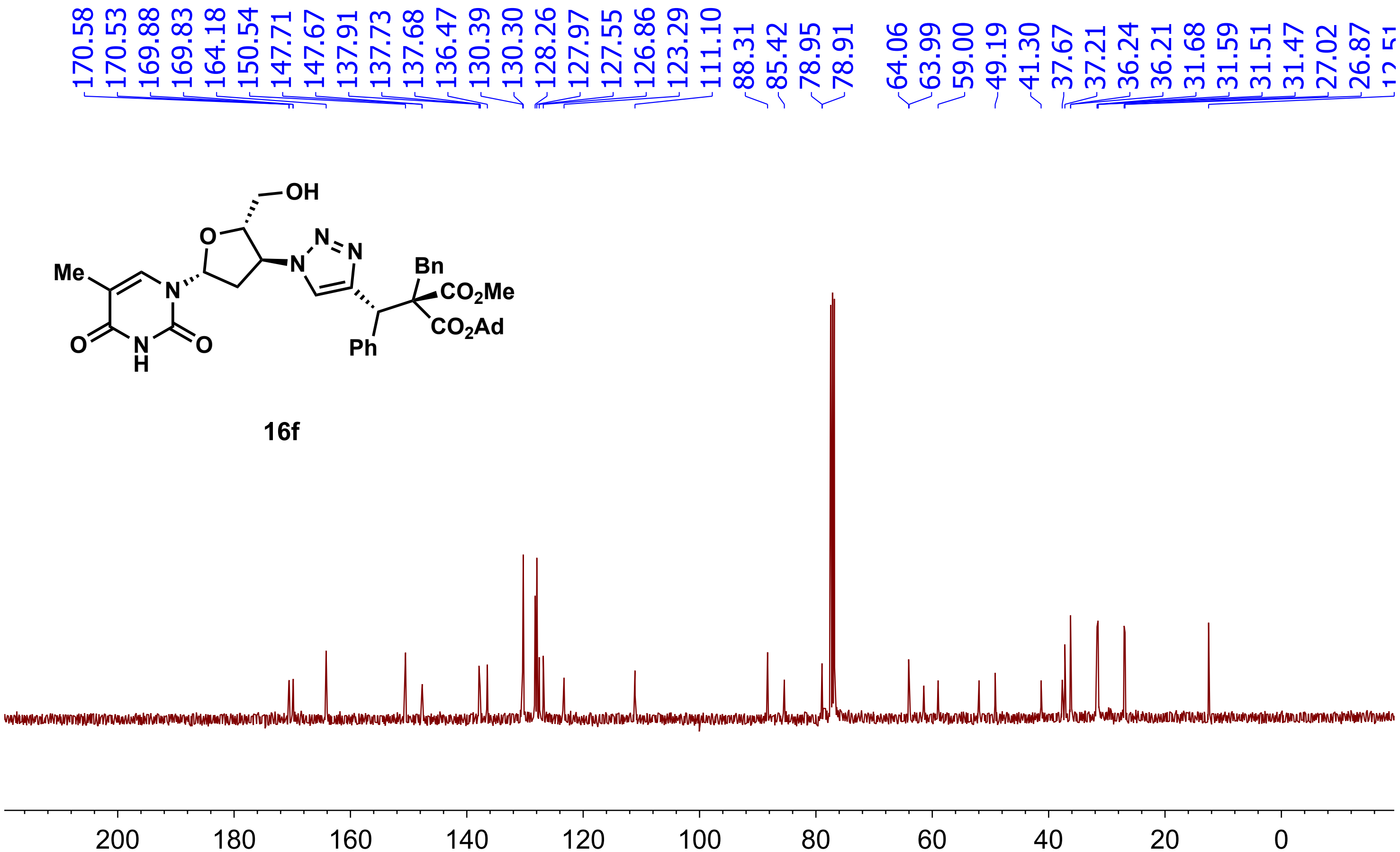

Supplementary Figure 232. ^{13}C NMR (101 MHz, $CDCl_3$) spectra for **16f**

CDCl3, 400.13 MHz

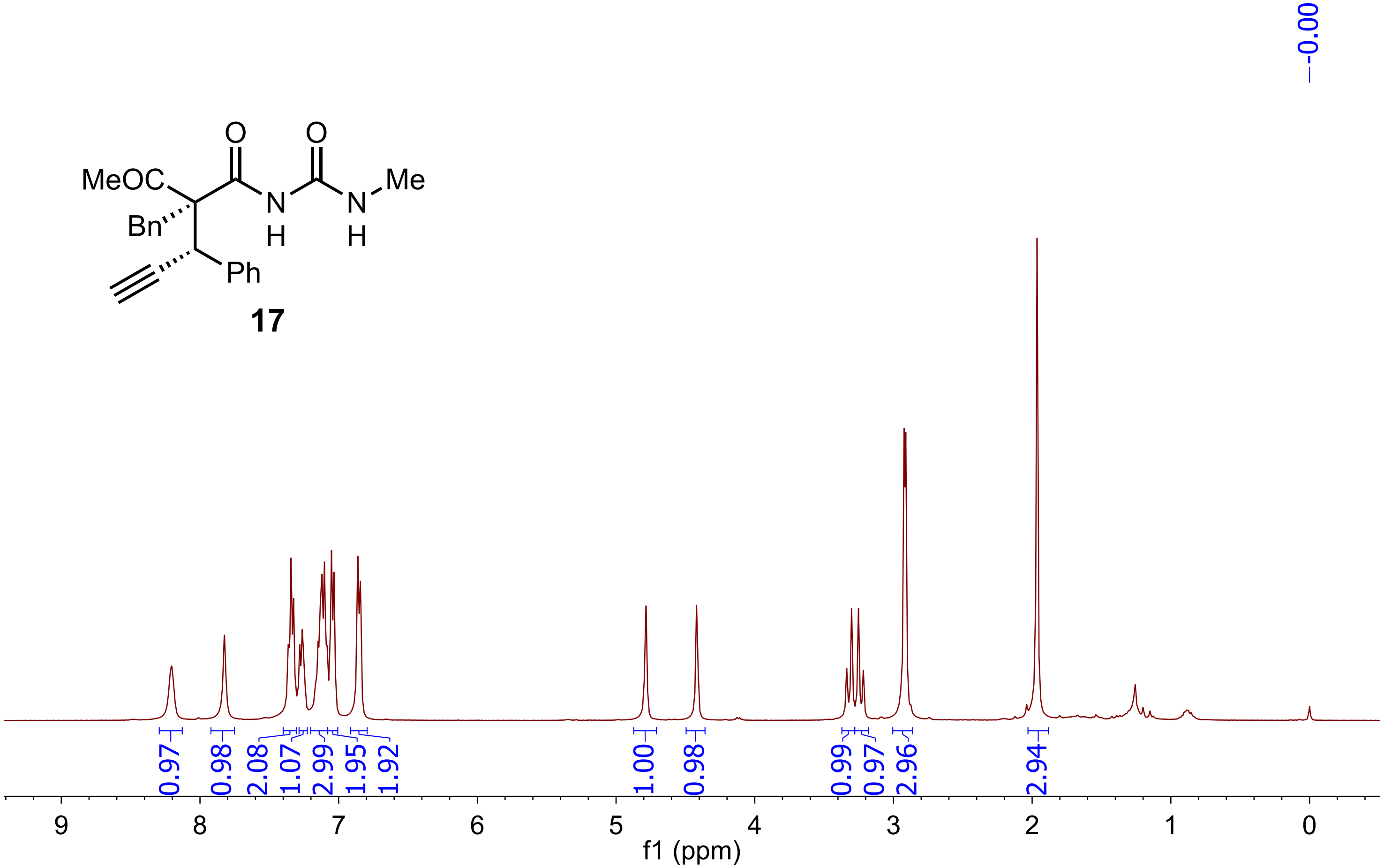

Supplementary Figure 233. ^{1}H NMR (400 MHz, $CDCl_3$) spectra for **17**

CDCl3, 100.62 MHz

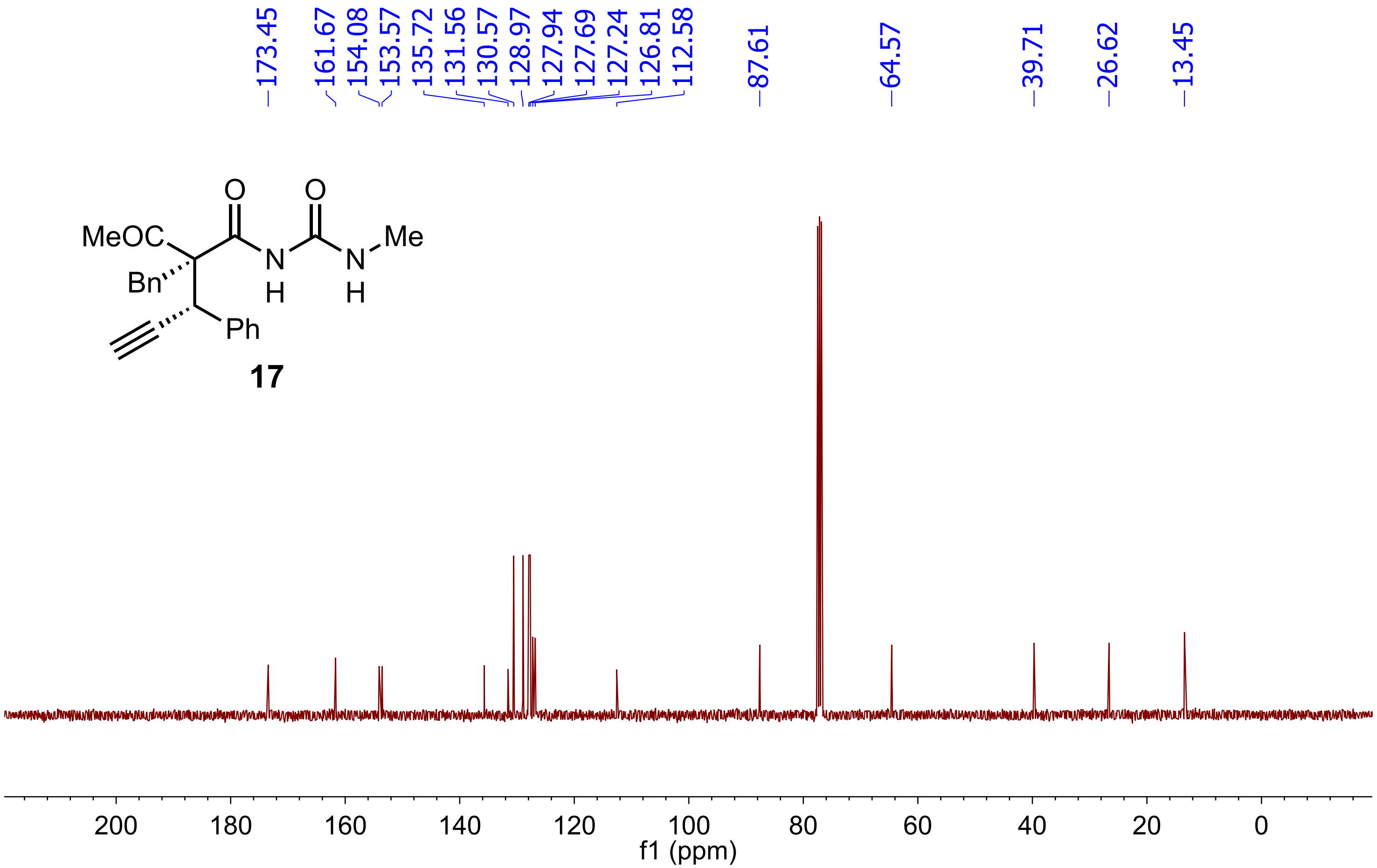

17

Supplementary Figure 234. ^{13}C NMR (101 MHz, $CDCl_3$) spectra for **17**

CDCl3, 400.13 MHz

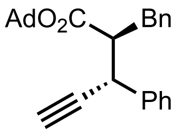

18

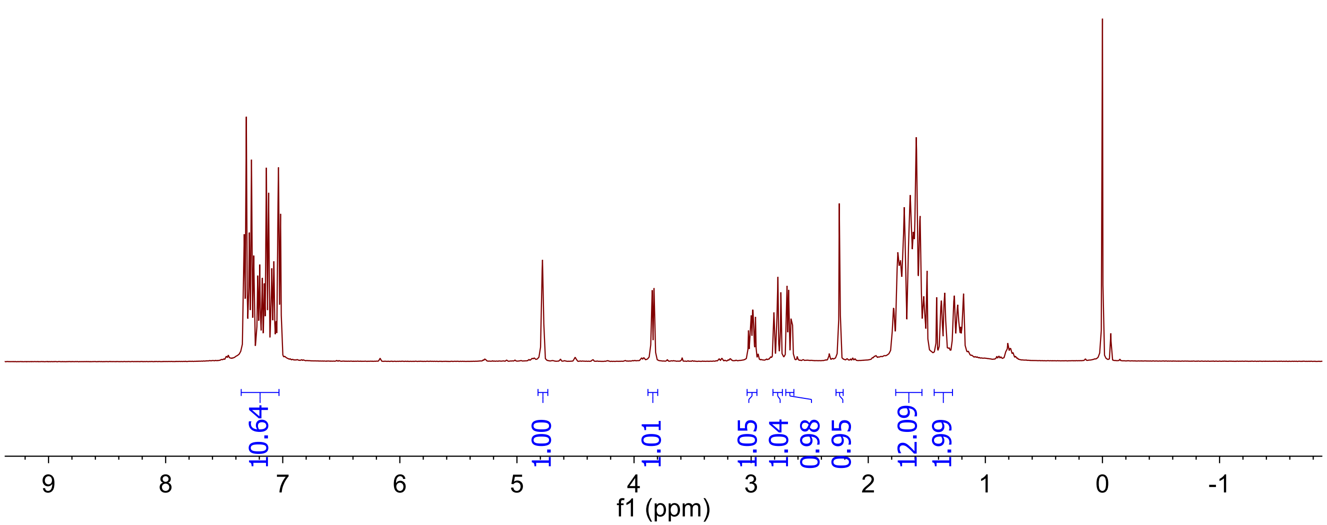

Supplementary Figure 235. ^{1}H NMR (400 MHz, $CDCl_3$) spectra for **18**

CDCl3, 100.62 MHz

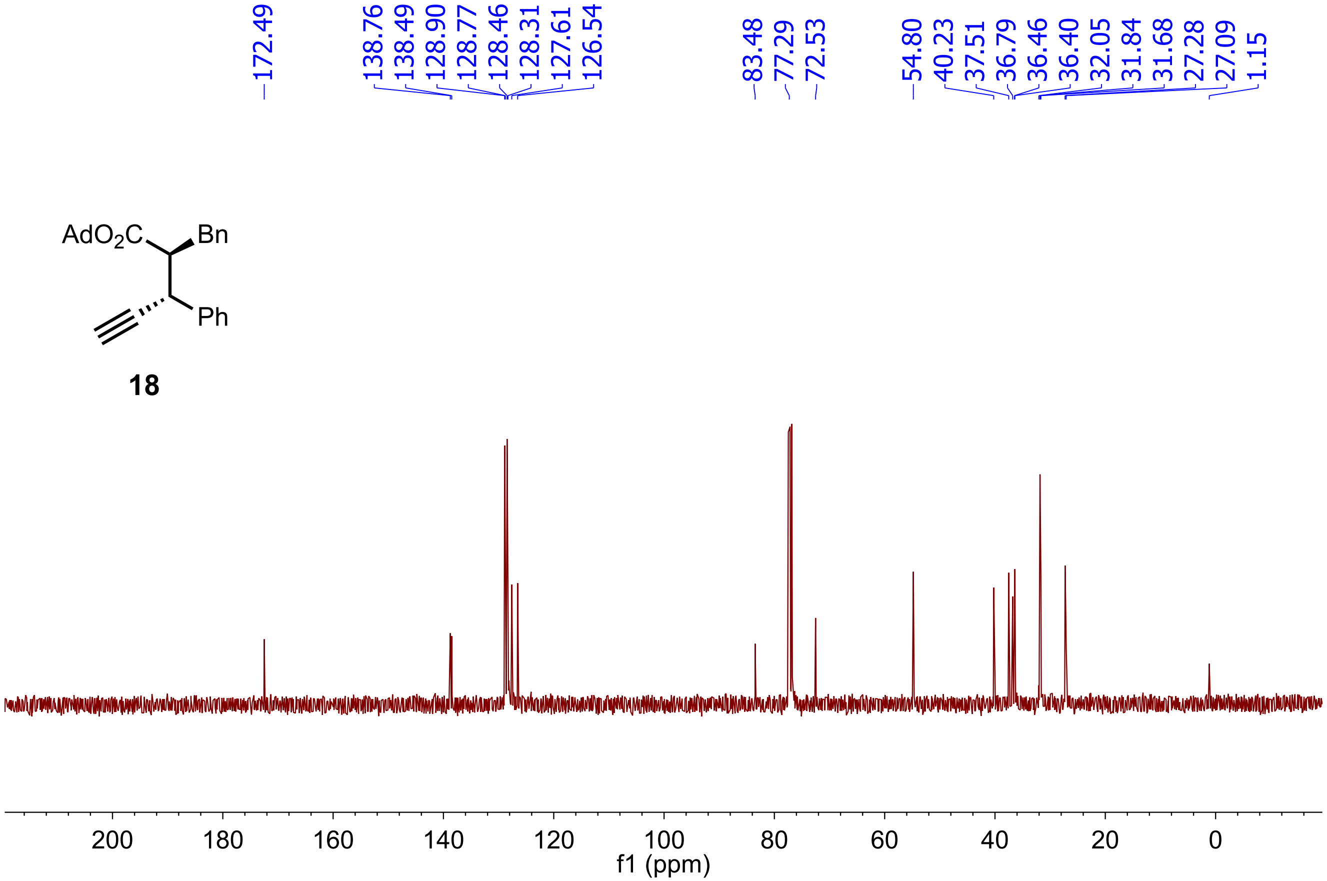

Supplementary Figure 236. ^{13}C NMR (101 MHz, $CDCl_3$) spectra for **18**

CDCl3, 400.13 MHz

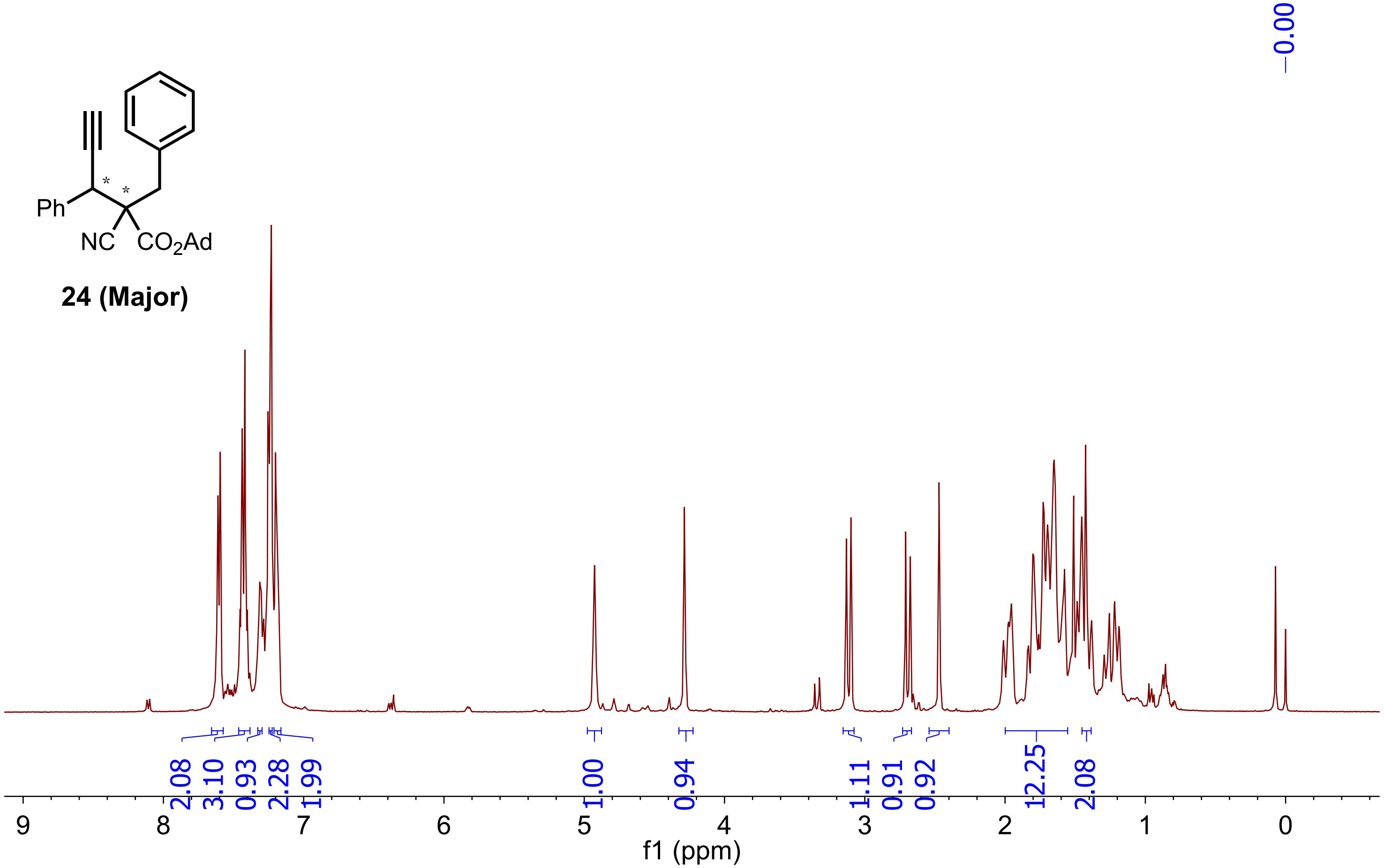

Supplementary Figure 237. ^{1}H NMR (400 MHz, $CDCl_3$) spectra for **24 (Major)**

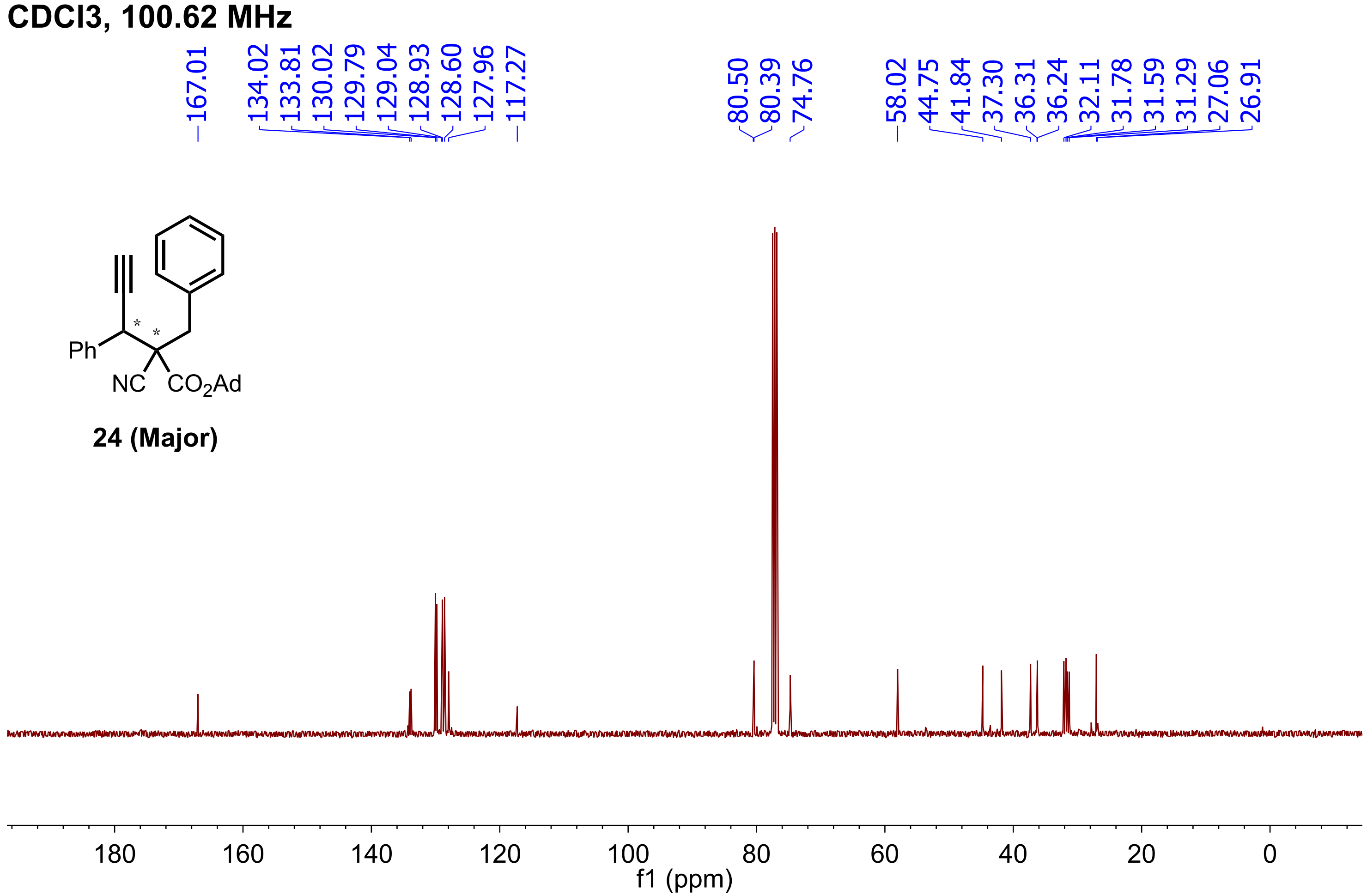

Supplementary Figure 238. ^{13}C NMR (101 MHz, $CDCl_3$) spectra for **24 (Major)**

CDCl3, 400.13 MHz

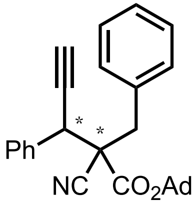

24 (Minor)

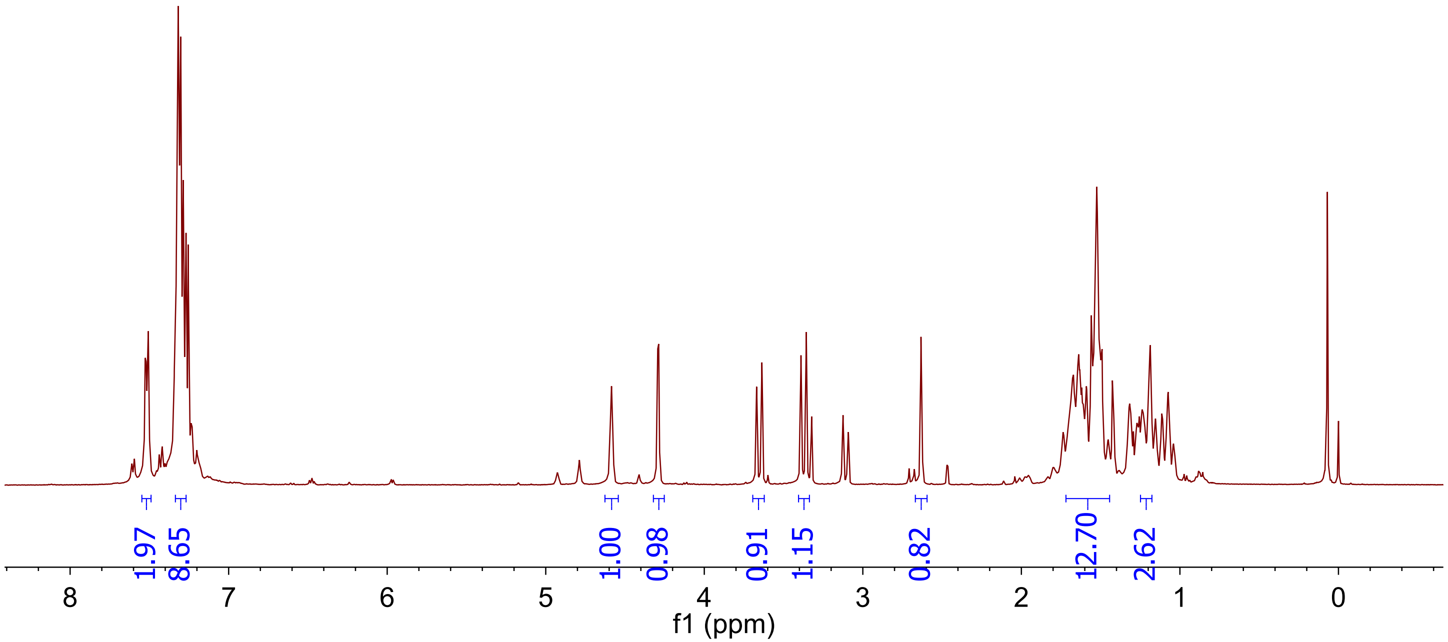

Supplementary Figure 239. ^{1}H NMR (400 MHz, $CDCl_3$) spectra for **24 (Minor)**

CDCl3, 100.62 MHz

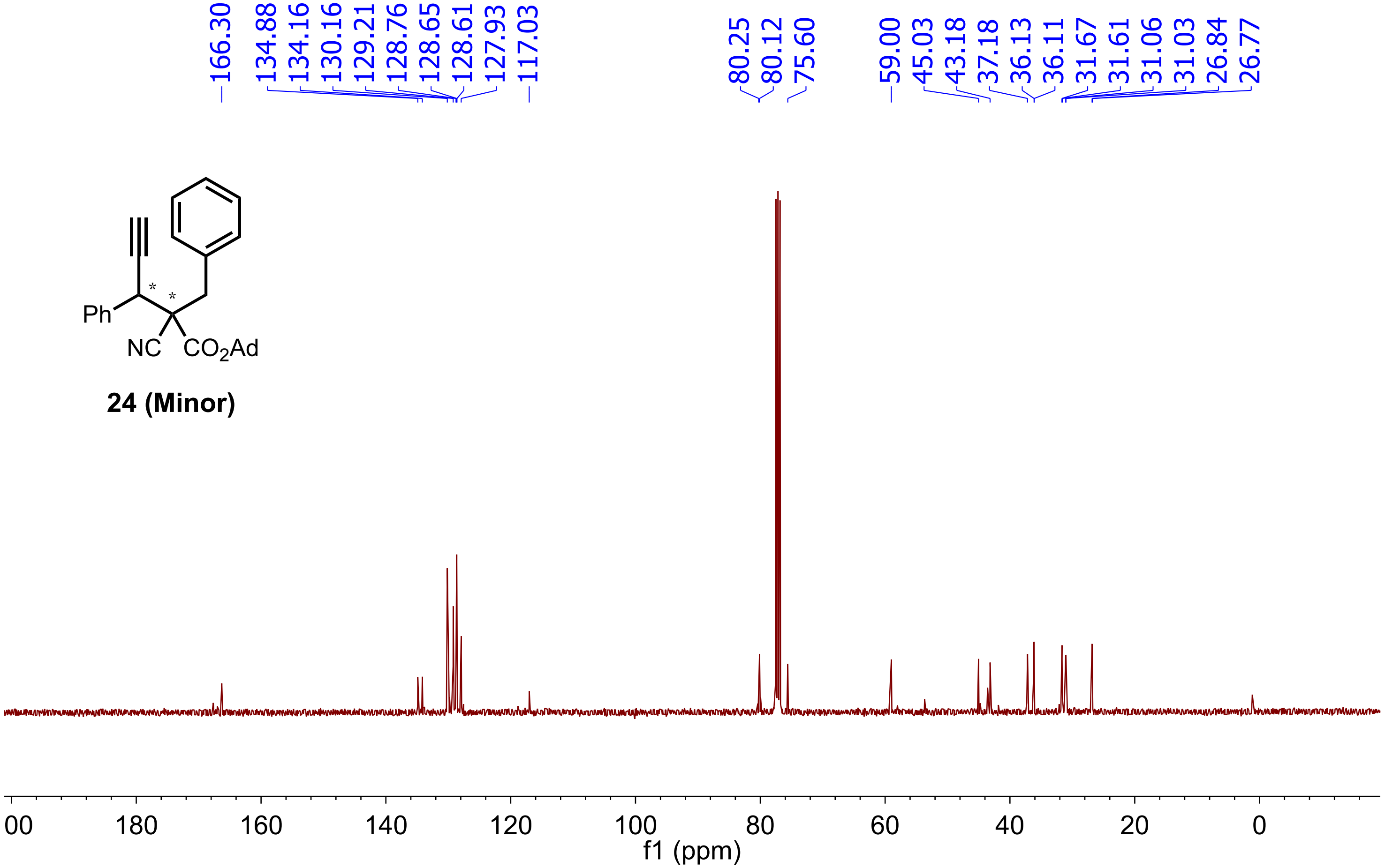

Supplementary Figure 240. ^{13}C NMR (101 MHz, $CDCl_3$) spectra for **24 (Minor)**

Supplementary References

[1] Bhor, S., Anilkumar, G., Tse, M. K., Klawonn, M., Döbler, C., Bitterlich, B., Grotevendt, A. & Beller, M. Synthesis of a New Chiral *N,N,N*-Tridentate Pyridinebisimidazoline Ligand Library and Its Application in Ru-Catalyzed Asymmetric Epoxidation. *Org. Lett,* **7**, 3393-3396 (2005).

[2] Gu, X., Zhang, Y., Xu, Z.-J. & Che, C.-M. Iron(III)-salan complexes catalysed highly enantioselective fluorination and hydroxylation of β-keto esters and N-Boc oxindoles. *Chem. Commun.*, **50**, 7870-7873 (2014).

[3] Weixler, R., Hehn, J. P. & Bach, T. On the Regioselectivity of the Intramolecular [2 + 2]-Photocycloaddition of Alk-3-enyl Tetronates. *J. Org. Chem.* **76**, 5924-5935 (2011).

[4] Vivier, D., Soussia, I. B., Rodrigues, N., Lolignier, S., Devilliers, M., Chatelain, F., Prival, L., Chapuy, E., Bourdier, G., Bennis, K., Lesage, F., Eschalier, A., Busserolles, J. & Ducki, S. Development of the First Two-Pore Domain Potassium Channel TWIK-Related K^+ Channel 1-Selective Agonist Possessing in Vivo Antinociceptive Activity. *J. Med. Chem,* **60**, 1076−1088 (2017).

[5] Navarro, I., Basset, J.-F., Hebbe, S., Major, S. M., Werner, T., Howsham, C., Bräckow, J. & Barrett, A. G. M. Biomimetic Synthesis of Resorcylate Natural Products Utilizing Late Stage Aromatization: Concise Total Syntheses of the Marine Antifungal Agents 15G256ι and 15G256β. *J. Am. Chem. Soc,* **130**, 10293-10298 (2008).

[6] Bradshaw, B., Parra, C. & Bonjoch, J. Organocatalyzed Asymmetric Synthesis of Morphans. *Org. Lett,* **15,** 2458-2461 (2013).

[7] Doan, N. N., Le, T. N., Nguyen, H. C., Hansen, P. E. & Duus, F. Ultrasound Assisted Synthesis of 5,9-Dimethylpentadecane and 5,9-Dimethylhexadecane – the Sex Pheromones of Leucoptera coffeella. *Molecules*, **12**, 2080-2088 (2007).

[8] Bétard, A., Wannapaiboon, S. & Fischer, R. A. Assessing the adsorption selectivity of linker functionalized, moisture-stable metal–organic framework thin films by means of an environment-controlled quartz crystal microbalance. *Chem. Commun.*, **48**, 10493-10495 (2012).
